# Co-expressed Pathways DataBase for Tomato: a database to predict pathways relevant to a query gene

**DOI:** 10.1186/s12864-017-3786-3

**Published:** 2017-06-05

**Authors:** Takafumi Narise, Nozomu Sakurai, Takeshi Obayashi, Hiroyuki Ohta, Daisuke Shibata

**Affiliations:** 1 Kazusa DNA Research Institute, 2-6-7 Kazusa-Kamatari, Kisarazu, Chiba, 292-0818 Japan; 2 Graduate School of Information Sciences, Tohoku University, 6-3-09 Aramaki-Aza-Aoba, Aoba-ku, Sendai, Miyagi, 980-8579 Japan; 3 Graduate School of Bioscience and Biotechnology, Tokyo Institute of Technology, 4259-B-65 Nagatsuta-cho, Midori-ku, Yokohama, Kanagawa, 226-8501 Japan

**Keywords:** Co-expression database, Pathway, Over-representation analysis, Gene set enrichment analysis, *Percentile*-score

## Abstract

**Background:**

Gene co-expression, the similarity of gene expression profiles under various experimental conditions, has been used as an indicator of functional relationships between genes, and many co-expression databases have been developed for predicting gene functions. These databases usually provide users with a co-expression network and a list of strongly co-expressed genes for a query gene. Several of these databases also provide functional information on a set of strongly co-expressed genes (i.e., provide biological processes and pathways that are enriched in these strongly co-expressed genes), which is generally analyzed via over-representation analysis (ORA). A limitation of this approach may be that users can predict gene functions only based on the strongly co-expressed genes.

**Results:**

In this study, we developed a new co-expression database that enables users to predict the function of tomato genes from the results of functional enrichment analyses of co-expressed genes while considering the genes that are not strongly co-expressed. To achieve this, we used the ORA approach with several thresholds to select co-expressed genes, and performed gene set enrichment analysis (GSEA) applied to a ranked list of genes ordered by the co-expression degree. We found that internal correlation in pathways affected the significance levels of the enrichment analyses. Therefore, we introduced a new measure for evaluating the relationship between the gene and pathway, termed the *percentile* (*p*)-score, which enables users to predict functionally relevant pathways without being affected by the internal correlation in pathways. In addition, we evaluated our approaches using receiver operating characteristic curves, which concluded that the *p*-score could improve the performance of the ORA.

**Conclusions:**

We developed a new database, named Co-expressed Pathways DataBase for Tomato, which is available at http://cox-path-db.kazusa.or.jp/tomato. The database allows users to predict pathways that are relevant to a query gene, which would help to infer gene functions.

**Electronic supplementary material:**

The online version of this article (doi:10.1186/s12864-017-3786-3) contains supplementary material, which is available to authorized users.

## Background

Gene co-expression, the similarity of gene expression profiles under various experimental conditions, has been used as an indicator of functional relationships between genes [1], and many databases using co-expression analysis have been developed for plant research, e.g., ATTED-II [2], ALCOdb [3], AraNet v2 [4], RiceNet v2 [5], PlaNet [6], PODC [7], CoP [8], VTCdb [9], and TFGD [10]. These databases provide users with a co-expression network and a list of strongly co-expressed genes for a query gene, which has successfully contributed to the characterization of many genes [11–14].

To further facilitate the prediction of gene functions, several of these databases have also provided functional information on a set of strongly co-expressed genes with a query gene, i.e., providing biological processes and pathways that are enriched in strongly co-expressed genes [2, 4, 8, 9]. These genes are usually analyzed by over-representation analysis (ORA), which can identify biological processes and pathways enriched in the set of selected genes of interest and help to extract biological meanings, and therefore, has been used to facilitate interpretation of gene expression data [15, 16]. However, a limitation of ORA is that the results are highly dependent on the cutoff used in selecting a set of genes of interest and ignore the effect of the remaining genes [15, 16].

Gene set enrichment analysis (GSEA) has been developed to overcome the limitation of ORA [17]. Unlike ORA, GSEA can assess, without selecting genes of interest, whether biological processes and pathways are enriched at the top of a ranked list of genes ordered by the degree of differential expression [17, 18]. This enables GSEA to be performed without being dependent on the cutoff used to select differentially expressed genes. GSEA may also be effective in the case of the co-expression analysis. Namely, GSEA may be applied to a ranked list of genes ordered by the degree of co-expression, which would enable the examination of gene–pathway relationships without being dependent on the threshold used to determine strongly co-expressed genes. However, there is no co-expression database that uses GSEA in this way. Currently, only strongly co-expressed genes are considered, and therefore, users cannot predict gene functions from other co-expressed genes.

*Solanum lycopersicum* (tomato) is a major crop worldwide and a model system for fruit development [19]. Elucidating the metabolic functions of individual tomato genes will facilitate rational design of metabolic engineering and breeding. Tomato fruit metabolites have been intensively studied [20]. For example, the biosynthesis mechanism of lycopene, the red pigment in tomato fruits, has been well-characterized both in vitro and in vivo [21], and its consumption is reported to be associated with lowered risks of cancer and cardiovascular disease [22].

In this study, we developed a new database that allows users to predict the function of tomato genes from the results of functional enrichment analyses of co-expressed genes. Our developed database provides, for each tomato gene, a ranked list of pathways in which higher-ranked pathways are more likely related to each gene. To create the ranked pathway list, we performed ORA with several thresholds to select co-expressed genes, and applied GSEA to a ranked list of genes ordered by the co-expression degree. This approach enables users to predict pathways that are relevant to the gene of interest while considering the genes that are not strongly co-expressed. In addition, we introduced a new measure for evaluating the relationship between the gene and pathway, which improved the prediction of functionally relevant pathways.

## Construction and content

We constructed a database, named Co-expressed Pathways DataBase for Tomato (CoxPathDB) [23], which aims to help users infer relevant pathways to a query gene and assist to predict its gene functions. In this section, we describe the procedural steps taken to construct the database and to evaluate our approach.

### Creation of the gene–gene correlation matrix

RNA-Seq data from tomato plants generated on the Illumina HiSeq or MiSeq platforms were downloaded from the DDBJ Sequence Read Archive (SRA) database [24]. The 1,234 downloaded SRA files were converted to FASTQ format using the fastq-dump utility of the SRA toolkit [25].

To remove low-quality reads and adapter sequences, the reads were trimmed using Trimmomatic version 0.36 [26] with the following parameters: ILLUMINACLIP:2:30:10 LEADING:3 TRAILING:3 SLIDINGWINDOW:20:20 MINLEN:50. Then, the reads were used to estimate gene expression levels by using kallisto version 0.43.0 [27] and the tomato cDNA sequences obtained from the RefSeq database [28]. In the case of single-end reads, the average fragment length was set to 200 bp. NCBI Entrez Gene IDs were converted to Ensembl Gene IDs by using BioMart [29] and the Kyoto Encyclopedia of Genes and Genomes (KEGG) database [30] (Additional file 1), and the genes whose IDs could not be converted were removed from the analysis. We filtered out low-quality SRA data (total estimated counts < 1 million), and then performed manual curation (e.g., removed small RNA-Seq data annotated as RNA-Seq data). Consequently, 790 SRA Runs were selected for further analysis (Additional file 1).

The expression values (transcripts per million) were quantile-normalized using the preprocessCore package in the R statistical software [31], and were log2-transformed after adding pseudo-count of 4. The 790 SRA Runs were clustered based on their gene expression profiles by the unweighted pair-group method using arithmetic averages (Additional file 2). They were clustered largely according to the sample tissues, suggesting the validity of the gene expression matrix. Then, the gene–gene correlation matrix was calculated with the gene expression matrix; correlations between gene expression profiles were calculated using the Pearson’s correlation coefficient. The gene expression matrix and the correlation matrix can be downloaded from the CoxPathDB webpage [23].

### Creation of the ranked gene lists

For each tomato gene, we created a ranked list of genes based on the values of correlation coefficients in the correlation matrix; all genes except for each target gene were ordered in decreasing order of correlation with the target gene. Consequently, 13,183 ranked gene lists were created.

### ORA of co-expressed genes

For each ranked gene list, we selected the top 100, 500, 1000, 1500, 2000, 2500, and 3000 ranked genes and performed ORA via the Fisher’s exact test implemented in the SciPy Python library. In ORA and GSEA (described later), we used the KEGG pathways downloaded from the KEGG database [30], because they cover a wide variety of metabolic pathways and are less redundant than Gene Ontology terms. We omitted pathways containing more than 500 genes or less than 15 genes because they might be too general or meaningless.

### GSEA of the ranked gene lists

We performed GSEA [17] for all 13,183 ranked gene lists using KEGG pathways. The sample permutation approach is not applicable for this analysis, and therefore, the gene permutation approach was used to obtain significance levels. To calculate the exact *p*-values, we used unweighted GSEA via dynamic programming [32], which is described as follows.

Given that a target gene is *t* and the ranked gene list for the target gene is *L*_*t*_, the overall ranked gene list is represented as

1 $$ L_{t}=\left\{g_{1}, \cdots, g_{n}\right\}\ (r_{1} \geq \cdots \geq r_{n}),  $$

where *n* is the total number of genes in the gene list and *r*∗ is the correlation coefficient of gene *g*∗ in the ranked gene list. We also assumed that the pathway to be tested is *S*, the number of genes in the pathway is *m*, and *V* is a vector where *V*(*j*) is the component corresponding to gene *g*_*j*_ in the ranked gene list *L*_*t*_. *V*(*j*) takes the value 1/*m* for the gene in pathway *S* and −1/(*n*−*m*) for the gene not in pathway *S*. The enrichment score (*ES*), the test statistic of GSEA, for pathway *S* is calculated as

2 $$ ES(S)=f(S) \max_{k=1, \cdots,n}\left| \sum\limits_{j=1}^{k}V(j)\right|,  $$

3 $$ f(S) = \left\{ \begin{array}{ll} +1 & \left(\sum_{j=1}^{k'}V(j) \geq 0\right), \\ -1 & \left(\sum_{j=1}^{k'}V(j) < 0\right), \end{array} \right.  $$

4 $$ k' = {\underset{k=1, \cdots,n}{\arg\max}}\;\left|\sum\limits_{j=1}^{k}V(j)\right|.  $$

Namely, *ES* is the maximum deviation of the running sum statistic, ${\sum \nolimits }_{j=1}^{k}V(j)$, from zero. The significance level is calculated depending on whether *E* *S*(*S*) is positive or negative. In the case of positive *E* *S*(*S*), the *p*-value for whether pathway *S* is enriched at the top of the ranked gene list *L*_*t*_ is computed by

5 $$ p\text{-value} = Pr \left\{ES_{+null} \geq ES_{+}(S) \right\},  $$

where *Pr* means probability, *E* *S*_+_ represents positive *ES*, and *E* *S*_+*null*_ is *E* *S*_+_ for a randomly generated pathway in which *m* genes are randomly distributed in the ranked gene list *L*_*t*_. This probability can be calculated exactly by using dynamic programming [32]. If the *p*-value is small (e.g., *p* < 0.05), pathway *S* is significant. In the case of negative *E* *S*(*S*), the *p*-value for whether pathway *S* is enriched at the bottom of the ranked gene list can be computed similarly. However, in this study, the *p*-values of pathways with negative *ES* were set to 1 because we focused on detecting pathways enriched at the top.

### Calculation of the *percentile* (*p*)-scores

In addition to the *p*-value, we calculated the *p*-score from the GSEA results, which is defined as follows. Additional file 3 shows the observed *ES* distribution of each pathway, which were obtained from the GSEA of 13,183 ranked gene lists. The *p*-score of each pathway for a gene of interest was calculated using the observed *ES* distribution. To estimate the probability density function of the observed *ES*, we used kernel density estimation, implemented in R version 3.3.1 [31]. Based on the estimated probability density function, the *p*-score of each pathway for a gene of interest was calculated as

6 $$ p\text{-score}=Pr \left\{ES \geq ES_{e} \right\},  $$

where *E* *S*_*e*_ is the *ES* for the gene of interest. As described in Eq. (5), the *p*-value is derived from the *ES* distribution of randomly generated pathways, whereas the *p*-score is derived from the observed *ES* distribution that was obtained from the GSEA of all 13,183 ranked gene lists.

### Evaluation of the ORA and GSEA results

We evaluated the ORA and GSEA results using receiver operating characteristic (ROC) curves. We classified gene–pathway pairs as “condition positive” or “condition negative” by using the relationship between genes and pathways in the KEGG database. If a gene was a member of a KEGG pathway, the gene–pathway pair was classified as condition positive; otherwise the gene–pathway pair was classified as condition negative. We calculated the true positive rate and false positive rate for each approach, drew ROC curves, and calculated the area under the curves (AUCs).

## Results

### Comparative analysis of ORA and GSEA

**Fig. 1:**
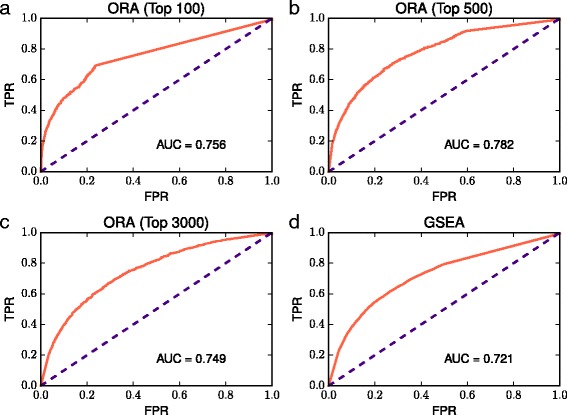
Evaluation of the ORA and GSEA. ROC curves drawn from the ORA of the *top* (**a**) 100, (**b**) 500, and (**c**) 3000 ranked genes and from (**d**) the GSEA of the ranked gene list

We evaluated the ORA of the top 100, 500, 1000, 1500, 2000, 2500, and 3000 ranked genes and the GSEA of the ranked lists of genes ordered by the co-expression degree (Fig. 1 and Additional file 4). We expected that if the *p*-value was small, the gene–pathway pair would be related to each other. Fig. 1 shows the ROC curves drawn from the ORA of the top 100, 500, and 3000 ranked genes and the GSEA of the ranked gene lists (see Additional file 4 for the ROC curves generated from the ORA of the top 1000–2500 ranked genes). These results demonstrated that the ORA of the top 500 ranked genes performed best, with the largest AUC value of 0.782. The AUC of GSEA was smaller than that of ORA, although GSEA is a threshold free approach.

### Effect of internal correlation in pathways

**Fig. 2:**
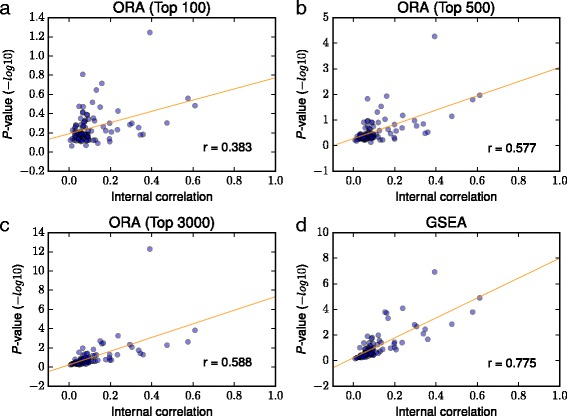
Effect of internal correlation in pathways. The average of −*l* *o* *g*10*p*-values (*y-axis*) was plotted against the internal correlation (*x-axis*), and the correlation coefficient between x and y, represented as r, was calculated. The ORA of the *top* (**a**) 100, (**b**) 500, and (**c**) 3000 ranked genes and (**d**) the GSEA of the ranked gene list are presented

We examined the effect of internal correlation in pathways (Fig. 2 and Additional file 5), which is reported to lead to an overestimation of the statistical significance of GSEA [18]. To calculate the internal correlation in each pathway, we averaged the correlation coefficients between all gene pairs in each pathway. Then, to examine the effect of the internal correlation, we averaged, for each pathway and approach, the −*l* *o* *g*10*p*-values of gene–pathway pairs where the gene was not a member of the pathway tested. We plotted the *p*-value averages against the internal correlation (Fig. 2 and Additional file 5).

Figure 2 and Additional file 5 indicate that the internal correlation in pathways affected the significance levels of ORA and GSEA, i.e., even when a gene is not a member of a pathway tested, the higher the internal correlation in the pathway, the smaller the *p*-value tended to become. The effect of internal correlation was larger in GSEA than in ORA (for GSEA, *r*= 0.775, and for ORA of the top 100–3000 ranked genes, *r*= 0.383, 0.577, 0.630, 0.628, 0.613, 0.598, and 0.588), which explains the lower performance of GSEA.

### Evaluation of the ranked lists of KEGG pathways

**Fig. 3:**
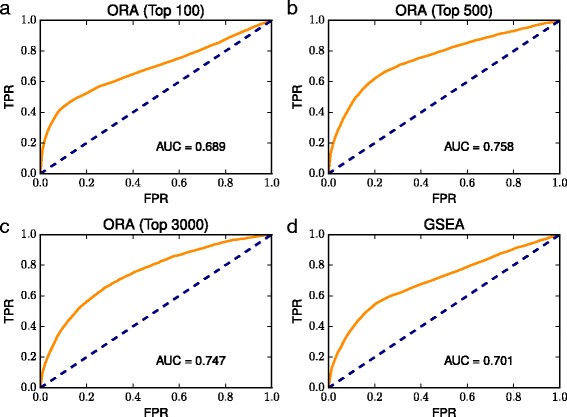
Evaluation of the ranked lists of KEGG pathways. ROC curves drawn from the ORA of the *top* (**a**) 100, (**b**) 500, and (**c**) 3000 ranked genes and from (**d**) the GSEA of the ranked gene list. The evaluation is based on the rank order of KEGG pathways for each gene

CoxPathDB provides, for each tomato gene, a ranked list of KEGG pathways, where higher ranked pathways are more likely related to each gene. Therefore, we evaluated whether the highly ranked pathways are actually related to each gene (Fig. 3; see Additional file 6 for the ROC curves generated from the ORA of the top 1000–2500 ranked genes). Figure 3 and Additional file 6 show that in addition to the evaluation of the *p*-value (Fig. 1 and Additional file 4), the ORA of the top 500 ranked genes had the second largest AUC value of 0.758.

### Improvement in the ranked lists of KEGG pathways

The findings highlighted in Fig. 2 may be biologically meaningful; the pathways with high internal correlation may tend to be related to many genes, and therefore, had small *p*-value averages (Fig. 2). However, if all pathways tested are ordered by increasing *p*-values, the pathway with high internal correlation tends to be ranked higher than that with low internal correlation, which prevents users from exploring a wide variety of gene–pathway relationships. For example, the “Photosynthesis” pathway had a high internal correlation (0.577) and small *p*-value averages (the −*l* *o* *g*10*p*-values were 3.82 for GSEA and 0.560, 1.80, 2.68, 2.92, 2.89, 2.80, and 2.68 for the ORA of the top 100–3000 ranked genes). This is consistent with a previous report stating that in the Arabidopsis gene co-expression network, genes involved in photosynthesis are strongly co-expressed and over-represented in the largest co-expression module [33]. This centrality of photosynthesis genes indicates that many genes are related to photosynthesis, and therefore, the Photosynthesis pathway is often ranked high, which may hinder the discovery of relationships among other pathways and genes.

Therefore, to compare pathways without being affected by the difference in their internal correlation, we calculated *p*-scores from the GSEA results, as described in the “Construction and content” section. The distribution of *p*-scores was similar among all pathways (Additional file 7), indicating that *p*-scores are not affected by internal correlation and suitable for examining diverse gene–pathway relationships.

**Fig. 4:**
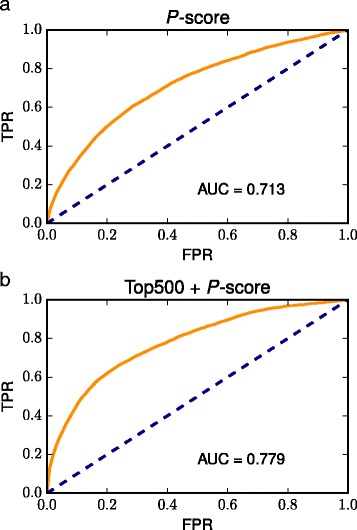
Evaluation of the GSEA using the *p*-score. ROC curves drawn from (**a**) the GSEA using the *p*-score and from (**b**) the analysis combining the ORA of the top 500 ranked genes and the GSEA using the *p*-score. The evaluation is based on the rank order of KEGG pathways for each gene

Another merit of the *p*-score is that it can be calculated with respect to all gene–pathway pairs. Although the ORA of the top 500 ranked genes performed well overall, these genes often do not contain any genes from the pathways to be tested. In such cases, the significance levels of the pathways cannot be compared, which decreases the performance of ORA. The *p*-scores can be used to order such pathways. The performance of GSEA using the *p*-score itself is smaller than those of ORA except for the ORA of the top 100 genes (Fig. 4 a). However, the combination of the ORA of the top 500 ranked genes and the GSEA using the *p*-score had the largest AUC value among the approaches we used (Fig. 4 b).

## Utility and discussion

**Fig. 5:**
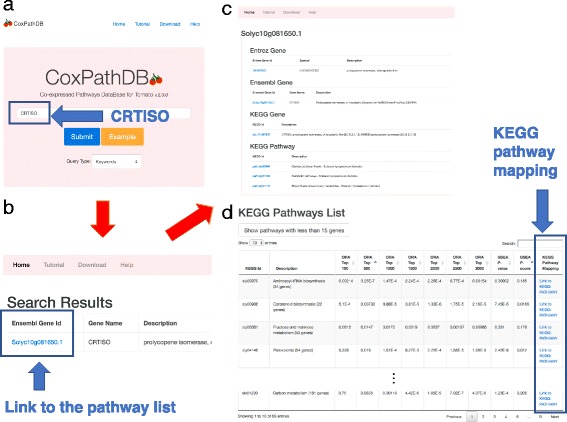
Example of the usage of CoxPathDB. **a** The search box on CoxPathDB. The word “CRTISO” is entered. **b** The search results page for the query, CRTISO. **c**, **d** The page for the CRTISO gene displaying (**c**) the brief gene information and (**d**) the ranked list of KEGG pathways

In this section, we give an example of the usage of CoxPathDB (Fig. 5). The search box on the CoxPathDB website is shown in Fig. 5 a. In this example, the word “CRTISO” is entered. The CRTISO enzyme catalyzes the isomerization of prolycopene to lycopene [21]. After clicking the “Submit” button, the search results are displayed on the search results page (Fig. 5 b). The “Ensembl Gene Id” column provides the links for the ranked list of KEGG pathways for the query gene. In this example, the blue link, “Solyc10g081650.1,” provides the link for the ranked list of the CRTISO gene (Fig. 5 b).

The webpage for each gene (Figs. 5c and d) also provides brief gene information from the Entrez database [34], Ensembl Plants [29] and the KEGG database [30], and the KEGG pathways that the gene belongs to (Fig. 5 c). Each id is a link to the external database where the detailed information is available. The KEGG pathways list is displayed just below the gene information (Fig. 5 d). In the default setting, it provides pathways that were ordered primarily by the increasing *p*-values obtained from the ORA of the top 500 ranked genes, and then, by *p*-scores calculated from GSEA, because this performed best among the approaches we used (Figs. 3 and 4 and Additional file 6). The results for pathways with less than 15 genes are available by clicking the “Show pathways with less than 15 genes” button.

The “KEGG Pathway Mapping” column provides links to the KEGG database (Fig. 5 d). In this example, the CRTISO gene is highly correlated with “Carotenoid biosynthesis,” the pathway to which the CRTISO gene belongs (the *p*-value [ORA Top 500] = 0.00732 and the *p*-score = 0.0166). When the corresponding link in the column is clicked, the genes in the Carotenoid biosynthesis pathway are mapped to the KEGG pathway database and colored in red or blue (Additional file 8); the CRTISO gene itself is colored in purple. The intensity of red and blue colors reflects the degree of positive and negative correlations, respectively. Green color means that the corresponding gene is present in the KEGG database but is not present in CoxPathDB, whereas white color means that the corresponding gene is not present in the KEGG database.

Figure 5 d shows that the CRTISO gene is also highly correlated with the “Aminoacyl-tRNA biosynthesis” pathway (the *p*-value [ORA Top 500] = 3.25E −7 and the *p*-score = 0.185). Although the *p*-value from the ORA of the top 500 ranked genes is small, the *p*-score is not very low. The *p*-score can compare pathways without being affected by the internal correlation in pathways (Additional file 7), and therefore, the reason for the small *p*-value may be the high internal correlation. On the other hand, the *p*-score of the Carotenoid biosynthesis pathway is relatively low (0.0166), indicating that its small *p*-value is not caused by high internal correlation. By checking the *p*-score, users can examine gene–pathway relationships while considering the context of pathways.

The “Peroxisome” pathway also exhibits a low *p*-value for the ORA of the top 500 ranked genes (0.0160) and a low *p*-score (0.0120). It has been proposed that antioxidative enzymes in peroxisomes may act as modulators of reactive oxygen species (ROS) signaling during pepper fruit maturation [35]. Solyc07g063430.2 (Entrez Gene ID: 101247444), encoding an MPV17 protein which may be involved in ROS metabolism, is strongly co-expressed with the CRTISO gene (Additional file 9), which may suggest that peroxisomal ROS generated via this protein modulates the lycopene biosynthesis during tomato fruit maturation.

## Conclusions

In this study, we developed a database named Co-expressed Pathways DataBase for Tomato [23]. The database provides, for each tomato gene, a ranked list of KEGG pathways, where the higher-ranked pathways are more likely related to each gene. The *p*-score enables users to predict functionally relevant pathways without being affected by internal correlation in pathways.

## Additional files

Additional file 1 ID correspondence table and information on the SRA Runs. The correspondences among Entrez Gene ID, Kegg Gene ID, and Ensemble Gene ID are shown in Table 1. Information (Run ID, Experiment ID, Study ID, sample tissue and cultivar) on the SRA Runs used to construct the gene expression matrix is shown in Table 2. (XLSX 1015 kb)

Additional file 2 Dendrogram of the RNA-Seq samples. The dendrogram of the clustering analysis of the RNA-Seq samples. The first color bar indicates the sample tissue, and the second one indicates the cultivar. (PDF 65 kb)

Additional file 3 Distribution of the observed *ES*. The frequency distribution of *ES* for each KEGG pathway, which was obtained from the GSEA of all ranked gene lists. (PDF 372 kb)

Additional file 4 Evaluation of the ORA of the top 1000–2500 ranked genes. ROC curves drawn from the ORA of the top (A) 1000, (B) 1500, (C) 2000, and (D) 2500 ranked genes. (PDF 132 kb)

Additional file 5 Effect of internal correlation in pathways. The average of −*l* *o* *g*10*p*-values (y-axis) was plotted against the internal correlation (x-axis), and the correlation coefficient between x and y, represented as r, was calculated. The ORA of the top (A) 1000, (B) 1500, (C) 2000, and (D) 2500 ranked genes. (PDF 205 kb)

Additional file 6 Evaluation of the ranked lists of KEGG pathways. ROC curves drawn from the ORA of the top (A) 1000, (B) 1500, (C) 2000, and (D) 2500 ranked genes. The evaluation is based on the rank order of KEGG pathways for each gene. (PDF 119 kb)

Additional file 7 Distribution of the *p*-score. The frequency distribution of *p*-score for each KEGG pathway. (PDF 352 kb)

Additional file 8 KEGG pathway mapping of the “Carotenoid biosynthesis” pathway genes. The query gene (CRTISO gene) itself is colored purple. The intensity of the red and blue colors reflects the degree of positive and negative correlations, respectively. (PNG 61 kb)

Additional file 9 KEGG pathway mapping of the “Peroxisome” pathway genes. The intensity of the red and blue colors reflects the degree of positive and negative correlations with the query gene (CRTISO gene), respectively. (PNG 33 kb)

## Abbreviations

AUC: Area under the curve
CoxPathDB: Co-expressed Pathways DataBase for Tomato
GSEA: Gene set enrichment analysis
KEGG: Kyoto Encyclopedia of Genes and Genomes
ORA: Over-representation analysis
*p*-score: *percentile*-score
ROC: Receiver operating characteristic
ROS: Reactive oxygen species
SRA: Sequence Read Archive
