# Supplementary figures and images for: Co-expressed Pathways DataBase for Tomato: a database to predict pathways relevant to a query gene
Source: BMC Genomics. 2017 Jun 5;18:437. doi: 10.1186/s12864-017-3786-3 (PMC5460524; doi:10.1186/s12864-017-3786-3)

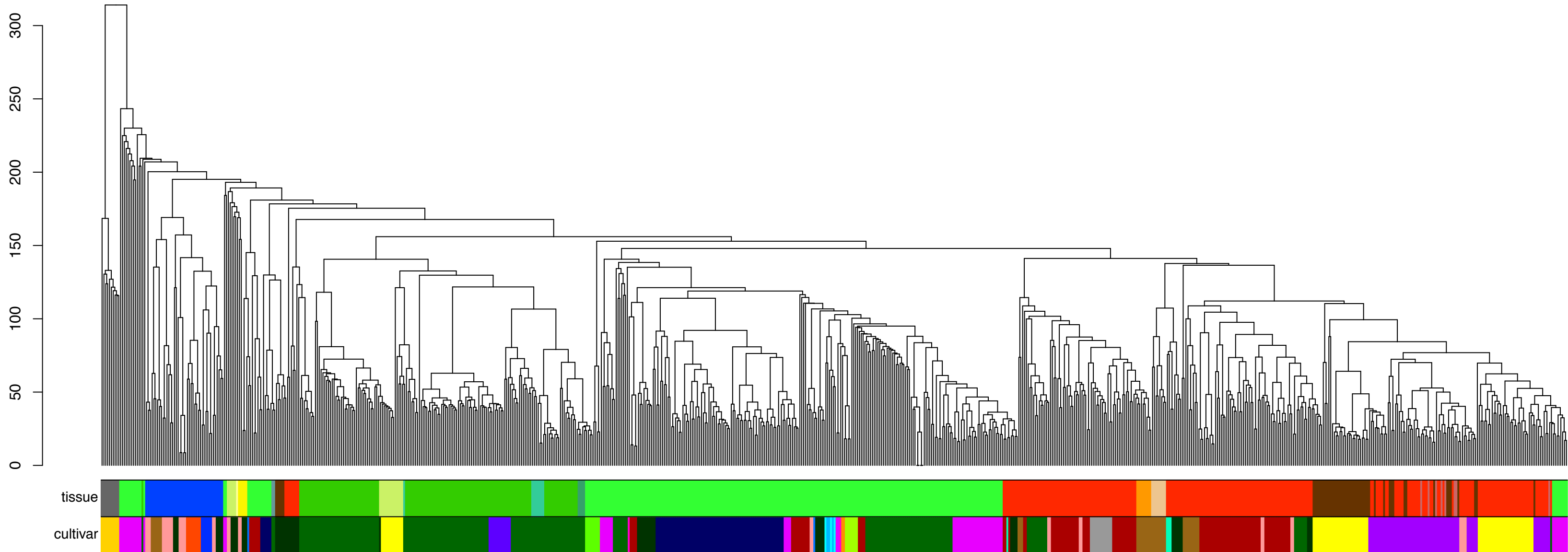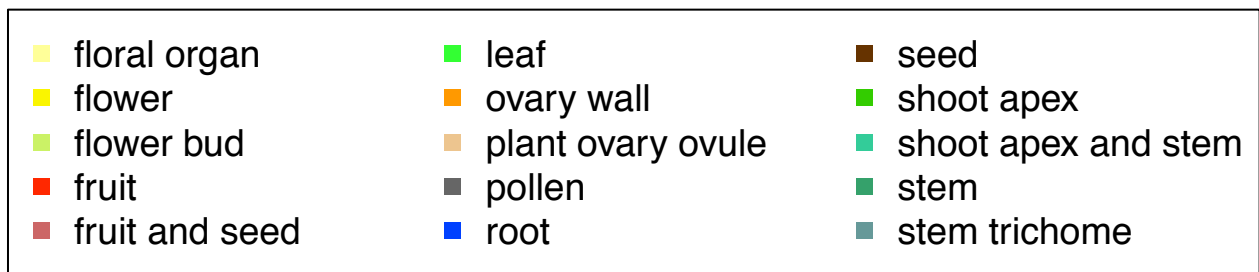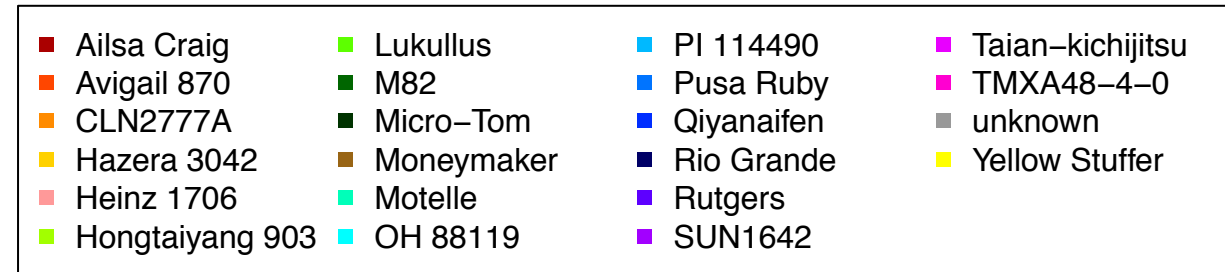

Supplement: Supplementary file 2 — Dendrogram of the RNA-Seq samples. The dendrogram of the clustering analysis of the RNA-Seq samples. The first color bar indicates the sample tissue, and the second one indicates the cultivar. (PDF 65 kb) [file 12864_2017_3786_MOESM2_ESM.pdf]

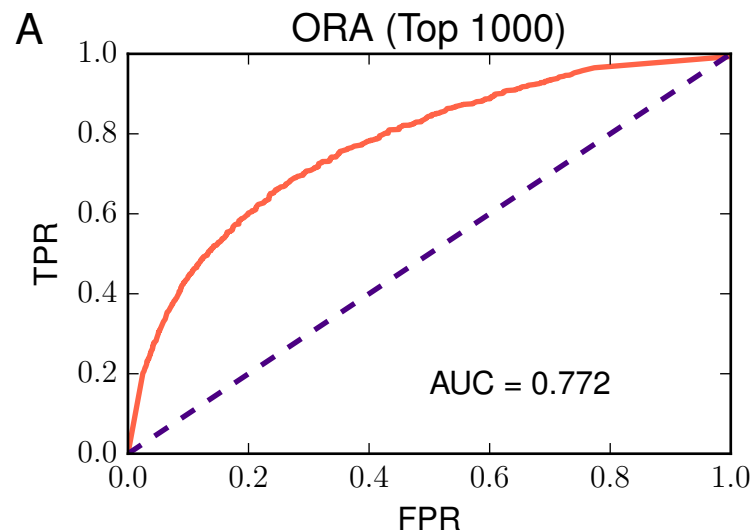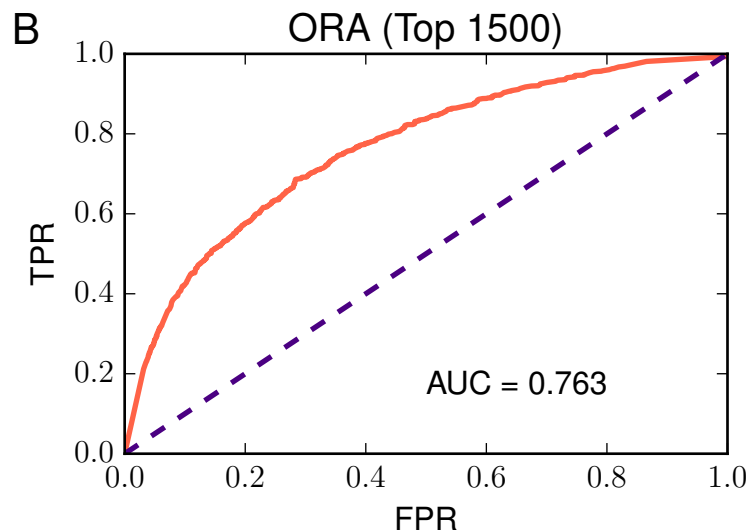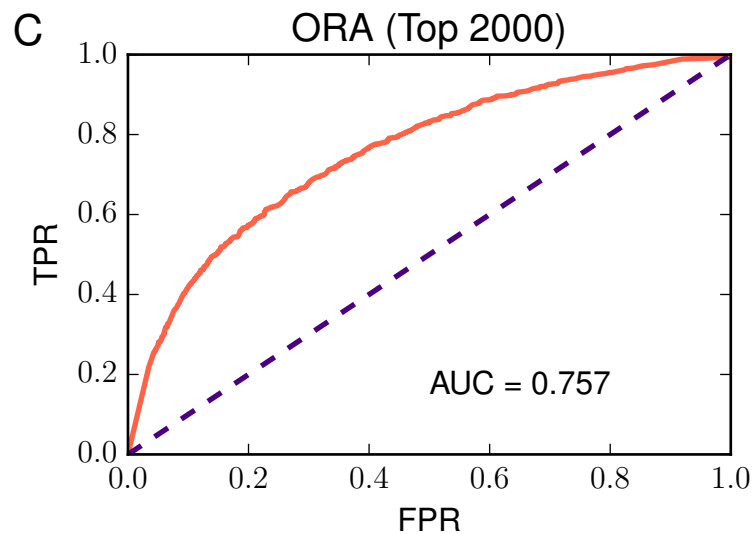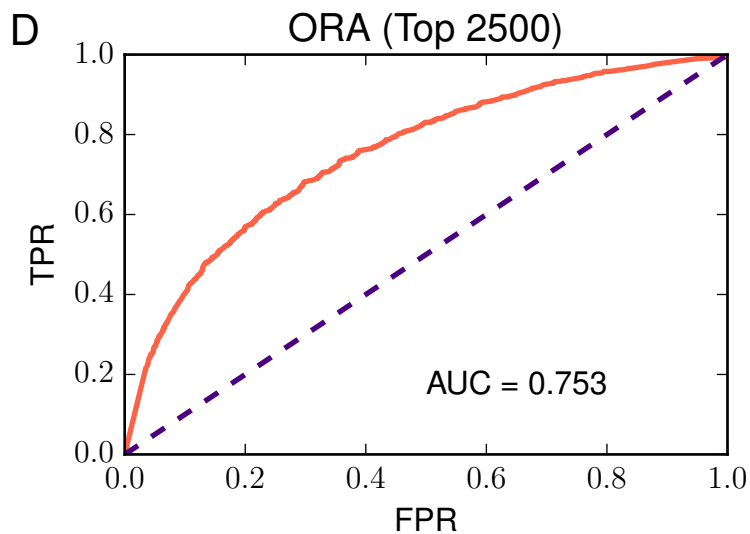

Supplement: Supplementary file 4 — Evaluation of the ORA of the top 1000–2500 ranked genes. ROC curves drawn from the ORA of the top (A) 1000, (B) 1500, (C) 2000, and (D) 2500 ranked genes. (PDF 132 kb) [file 12864_2017_3786_MOESM4_ESM.pdf]

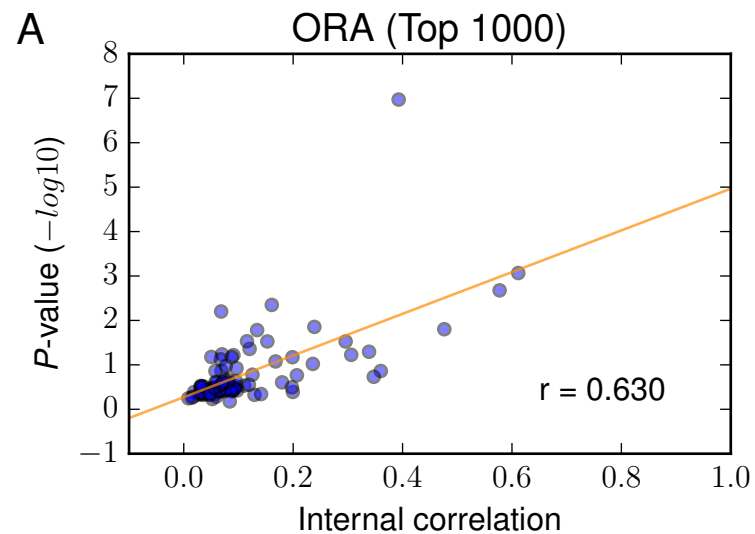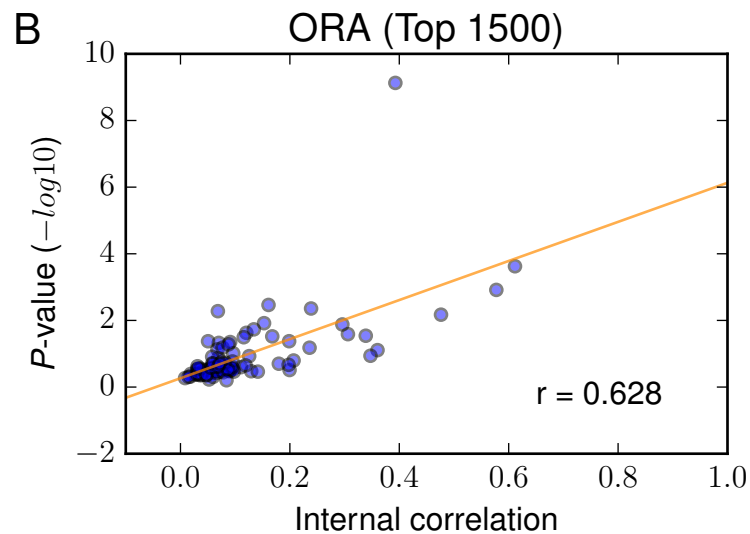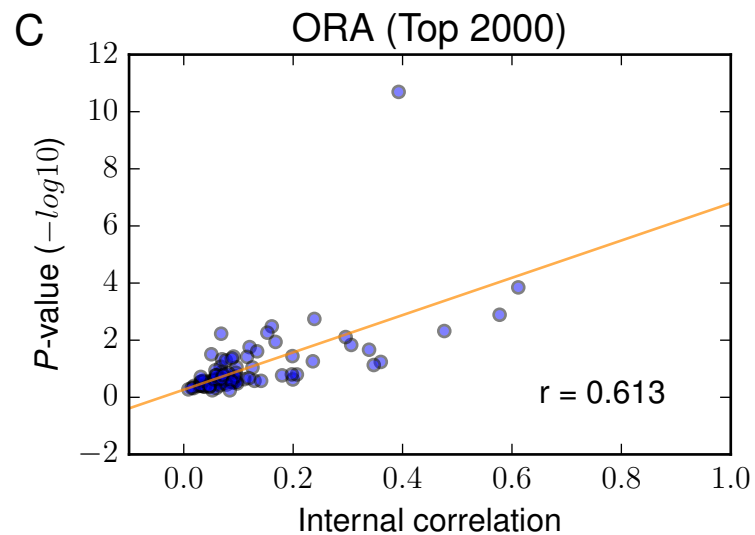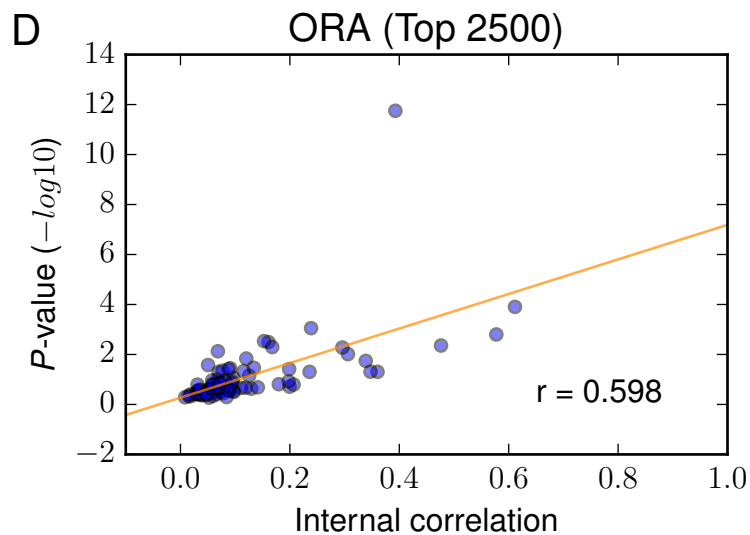

Supplement: Supplementary file 5 — Effect of internal correlation in pathways. The average of −l o g10p-values (y-axis) was plotted against the internal correlation (x-axis), and the correlation coefficient between x and y, represented as r, was calculated. The ORA of the top (A) 1000, (B) 1500, (C) 2000, and (D) 2500 ranked genes. (PDF 205 kb) [file 12864_2017_3786_MOESM5_ESM.pdf]

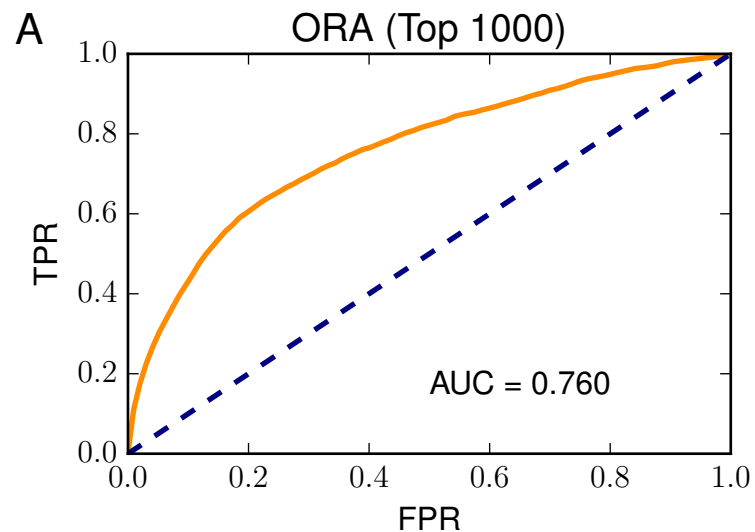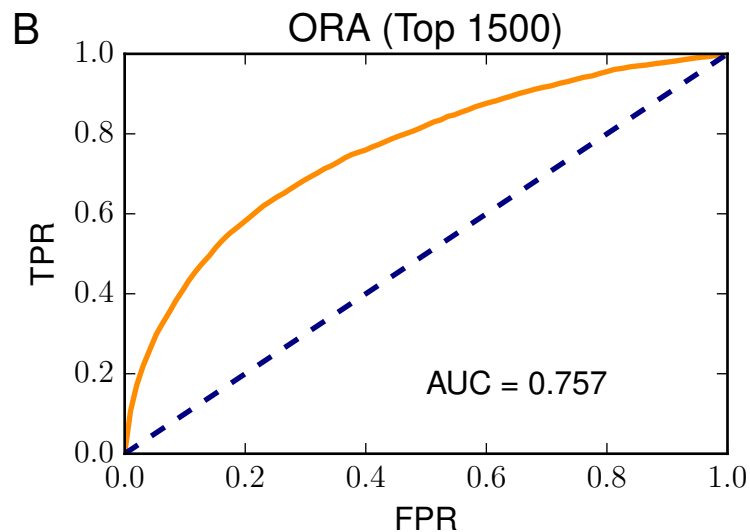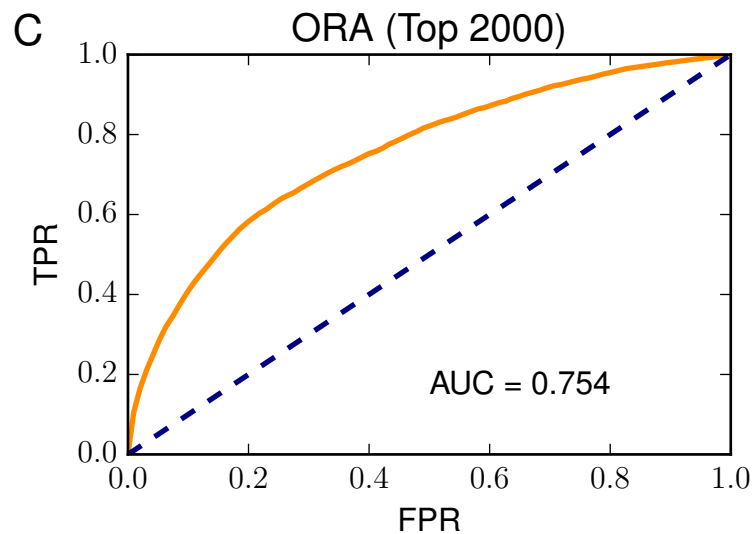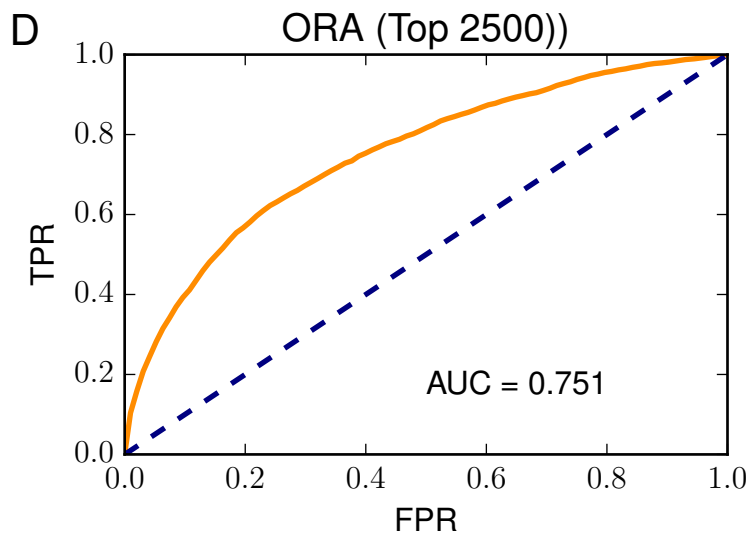

Supplement: Supplementary file 6 — Evaluation of the ranked lists of KEGG pathways. ROC curves drawn from the ORA of the top (A) 1000, (B) 1500, (C) 2000, and (D) 2500 ranked genes. The evaluation is based on the rank order of KEGG pathways for each gene. (PDF 119 kb) [file 12864_2017_3786_MOESM6_ESM.pdf]

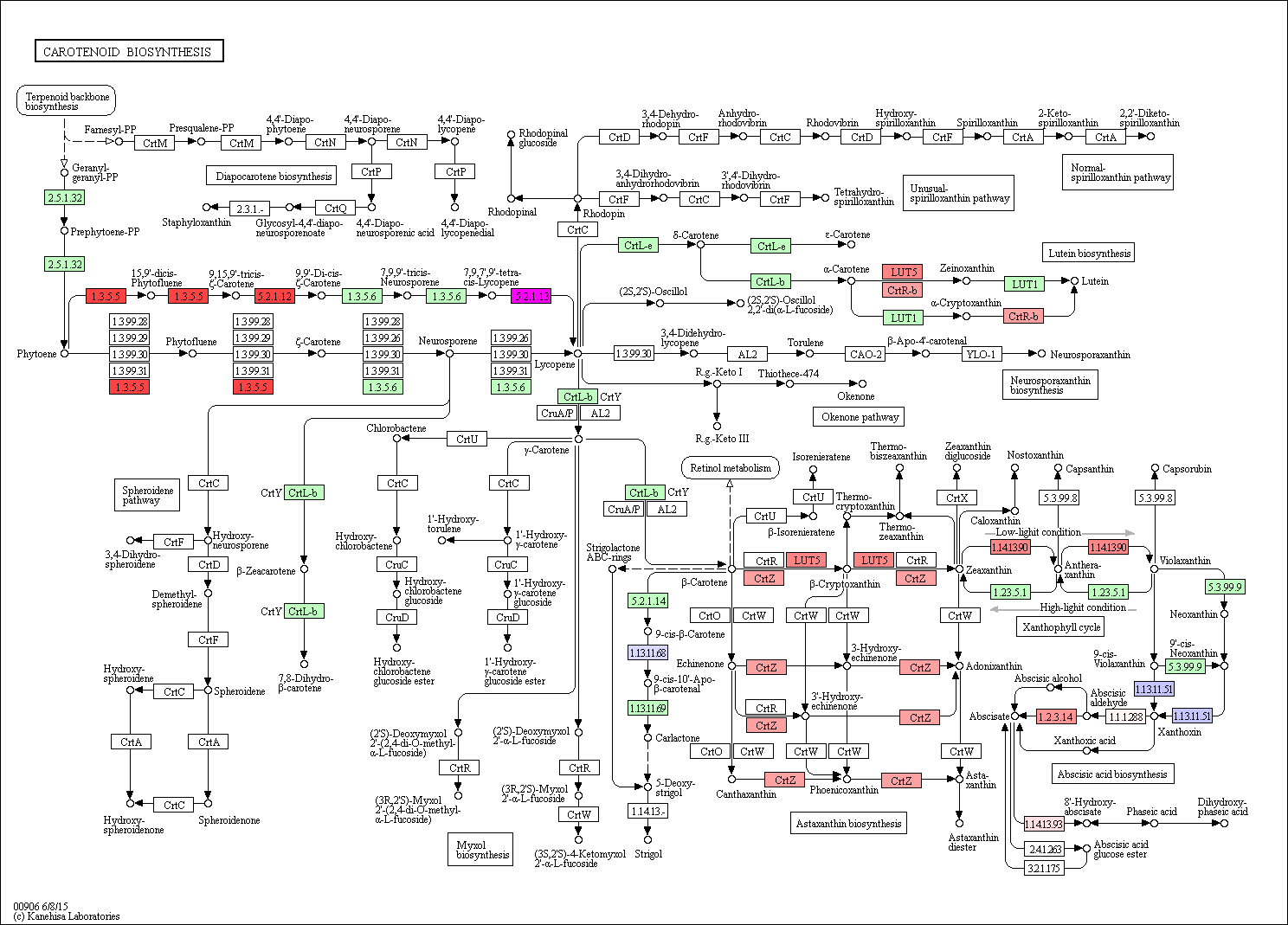

Supplement: Supplementary file 8 — KEGG pathway mapping of the “Carotenoid biosynthesis” pathway genes. The query gene (CRTISO gene) itself is colored purple. The intensity of the red and blue colors reflects the degree of positive and negative correlations, respectively. (PNG 61 kb) [file 12864_2017_3786_MOESM8_ESM.png]

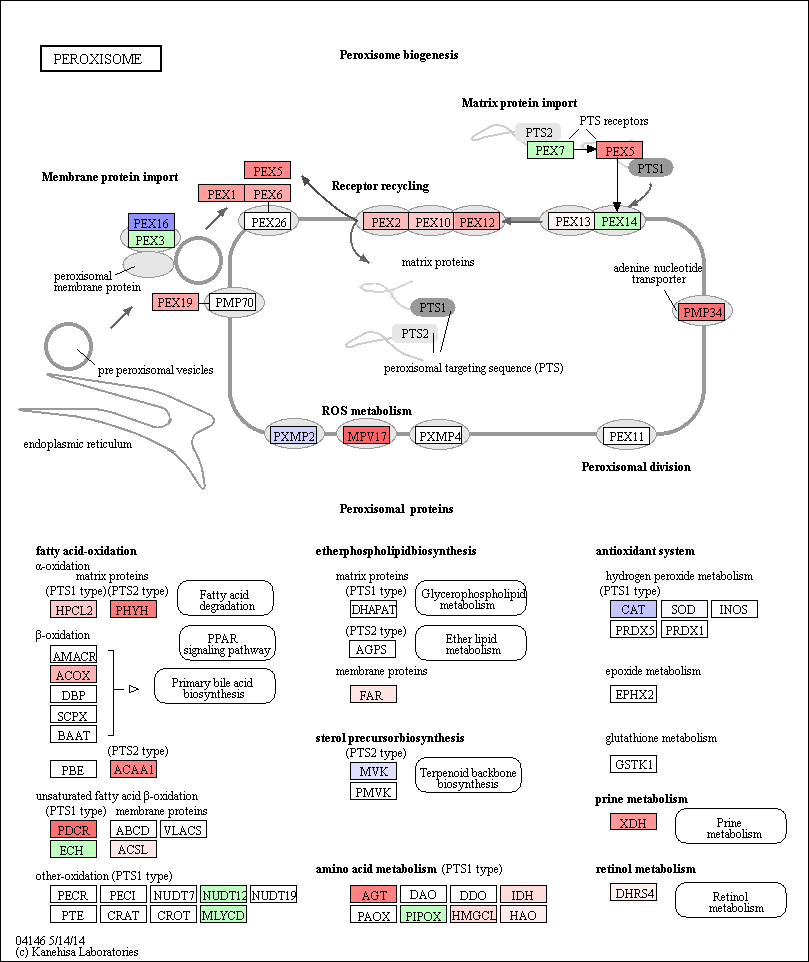

Supplement: Supplementary file 9 — KEGG pathway mapping of the “Peroxisome” pathway genes. The intensity of the red and blue colors reflects the degree of positive and negative correlations with the query gene (CRTISO gene), respectively. (PNG 33 kb) [file 12864_2017_3786_MOESM9_ESM.png]
