# Supplementary material for: Co-expressed Pathways DataBase for Tomato: a database to predict pathways relevant to a query gene
Source: BMC Genomics. 2017 Jun 5;18:437. doi: 10.1186/s12864-017-3786-3 (PMC5460524; doi:10.1186/s12864-017-3786-3)
Supplement: Supplementary file 3 — Distribution of the observed ES. The frequency distribution of ES for each KEGG pathway, which was obtained from the GSEA of all ranked gene lists. (PDF 372 kb) [file 12864_2017_3786_MOESM3_ESM.pdf]

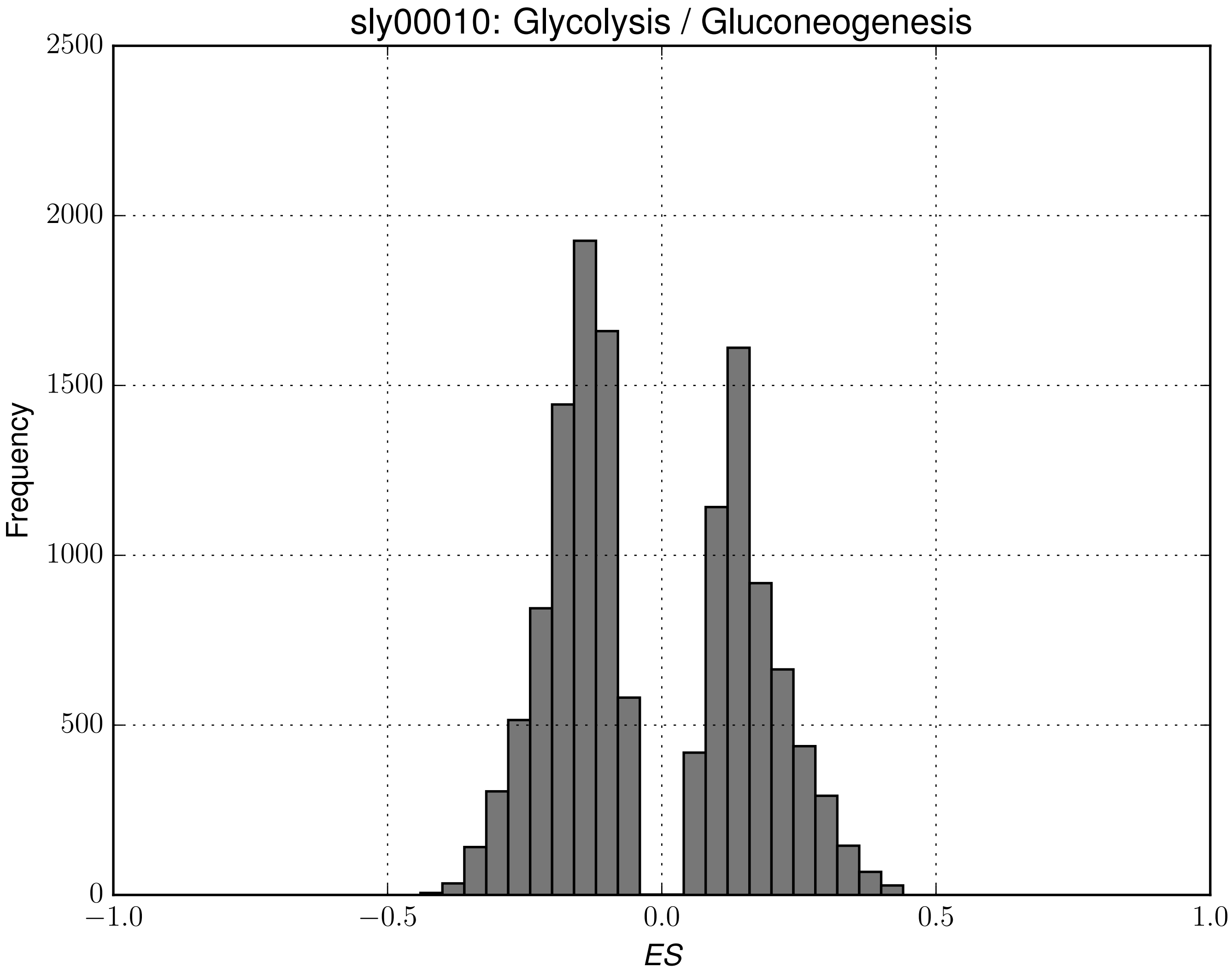
sly00010: Glycolysis / Gluconeogenesis
Frequency
ES
2500
2000
1500
1000
500
0
−1.0
−0.5
0.0
0.5
1.0

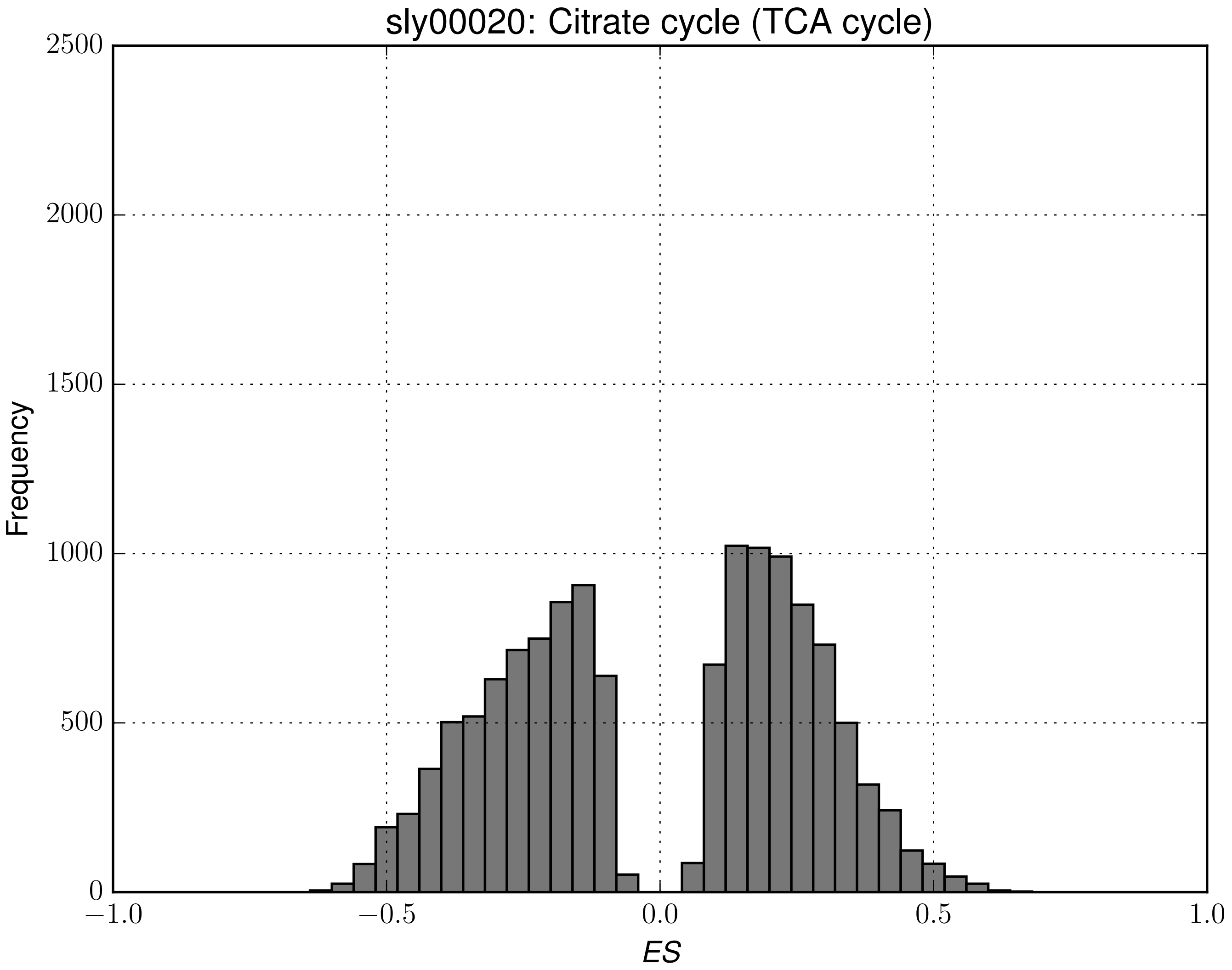
sly00020: Citrate cycle (TCA cycle)
Frequency
ES
2500
2000
1500
1000
500
0
−1.0
−0.5
0.0
0.5
1.0

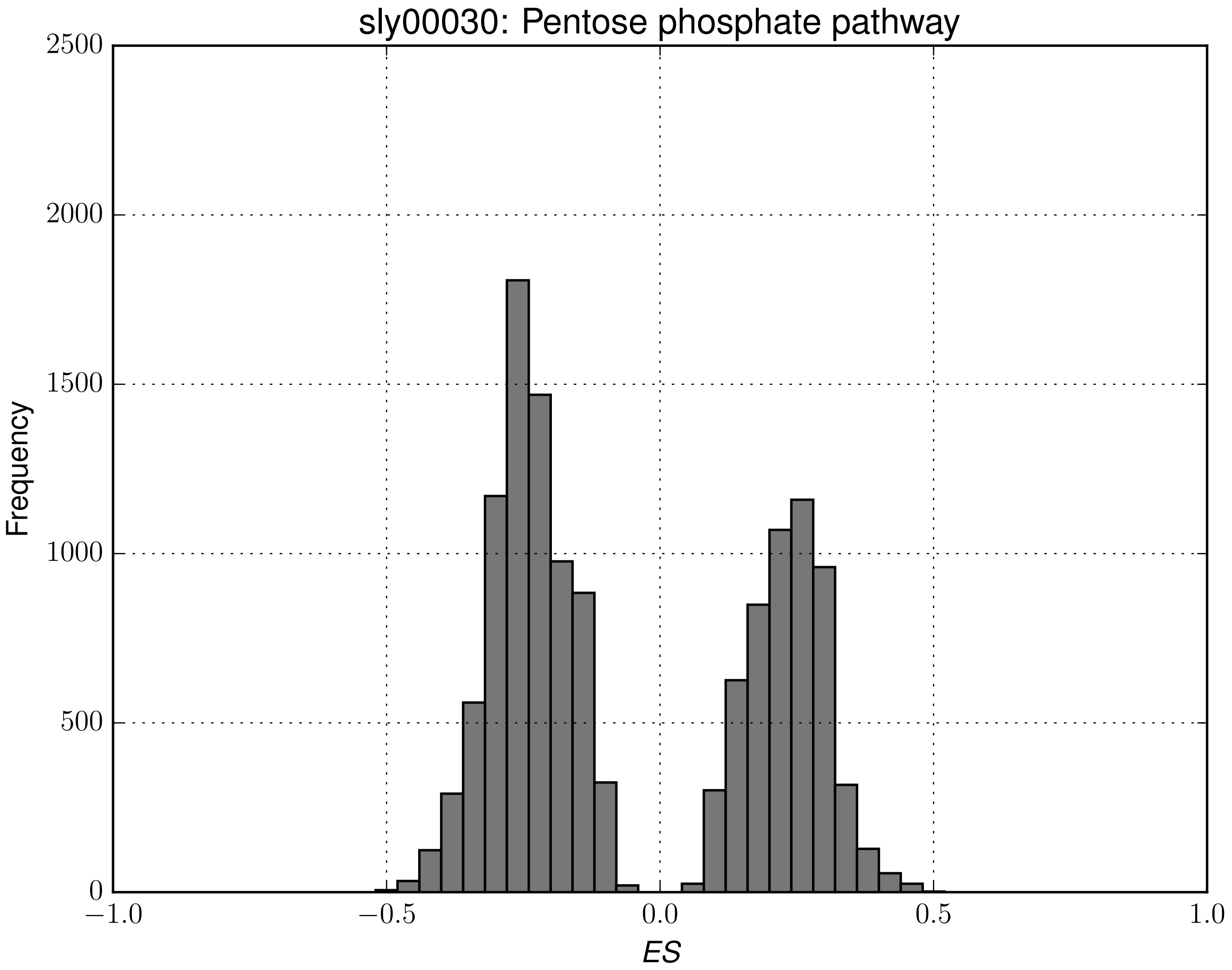
sly00030: Pentose phosphate pathway
Frequency
ES
2500
2000
1500
1000
500
0
−1.0
−0.5
0.0
0.5
1.0

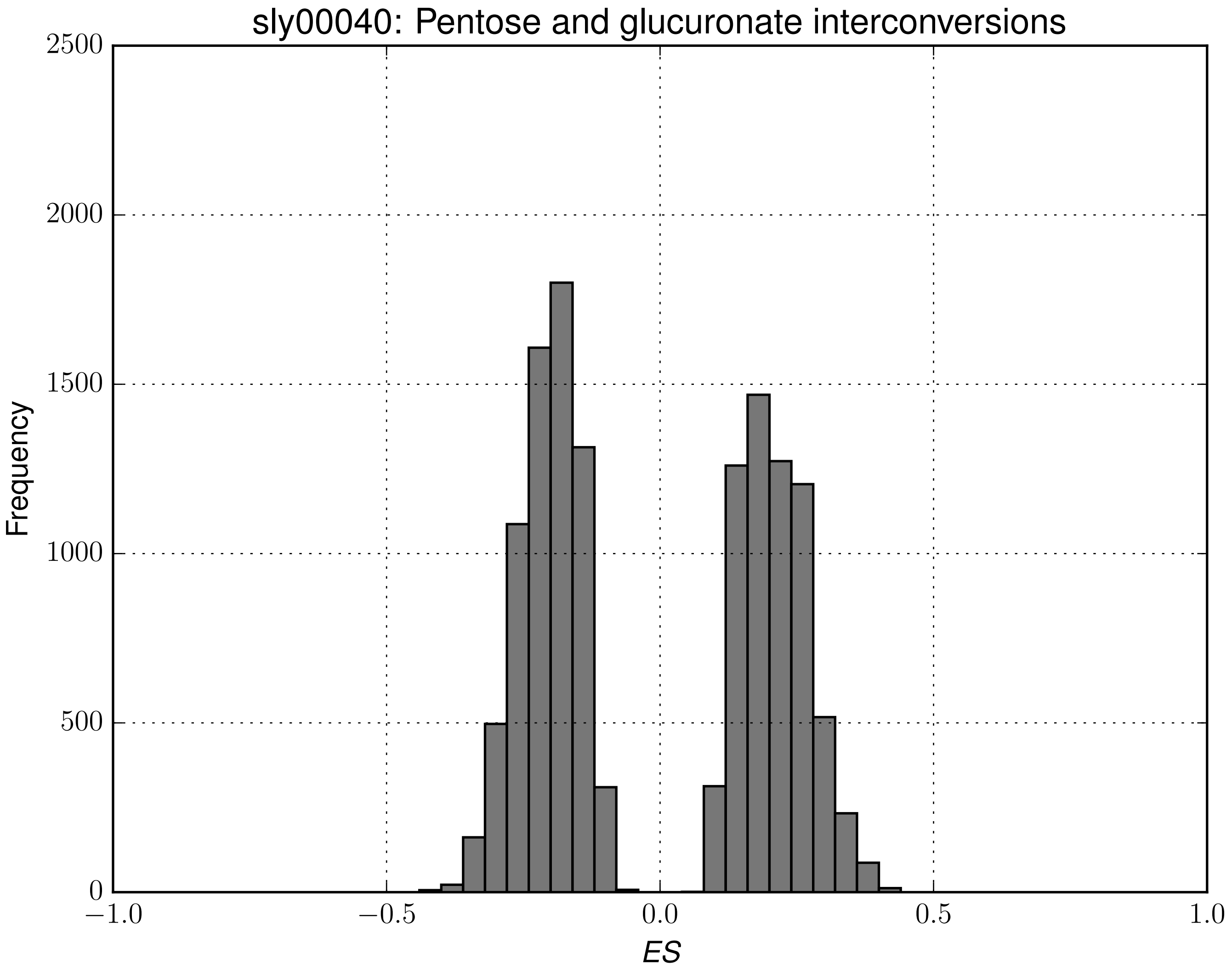

sly00040: Pentose and glucuronate interconversions
Frequency
ES
2500
2000
1500
1000
500
0
−1.0
−0.5
0.0
0.5
1.0

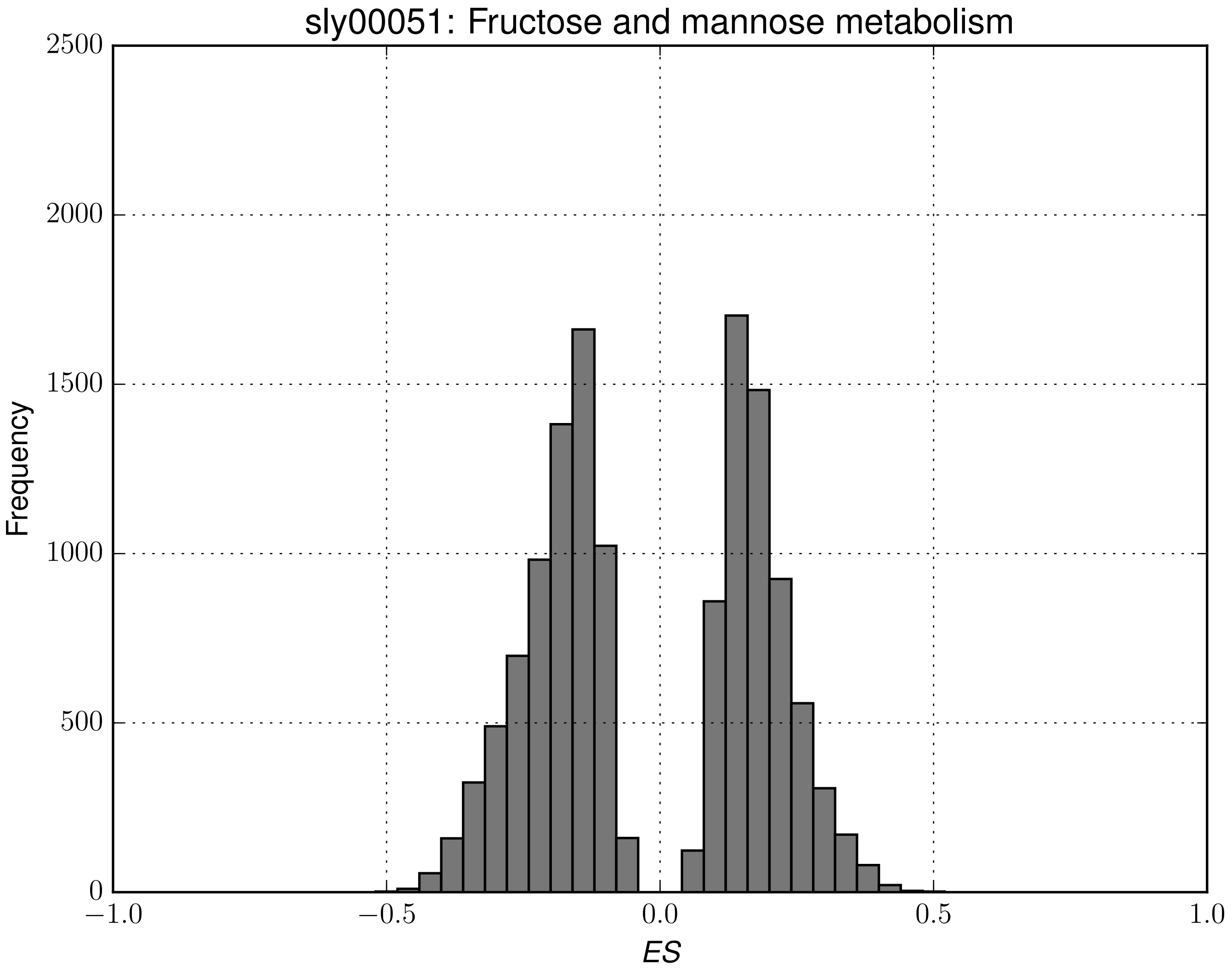

sly00051: Fructose and mannose metabolism
2500
2000
1500
1000
500
0
Frequency
−1.0
−0.5
0.0
0.5
1.0
ES

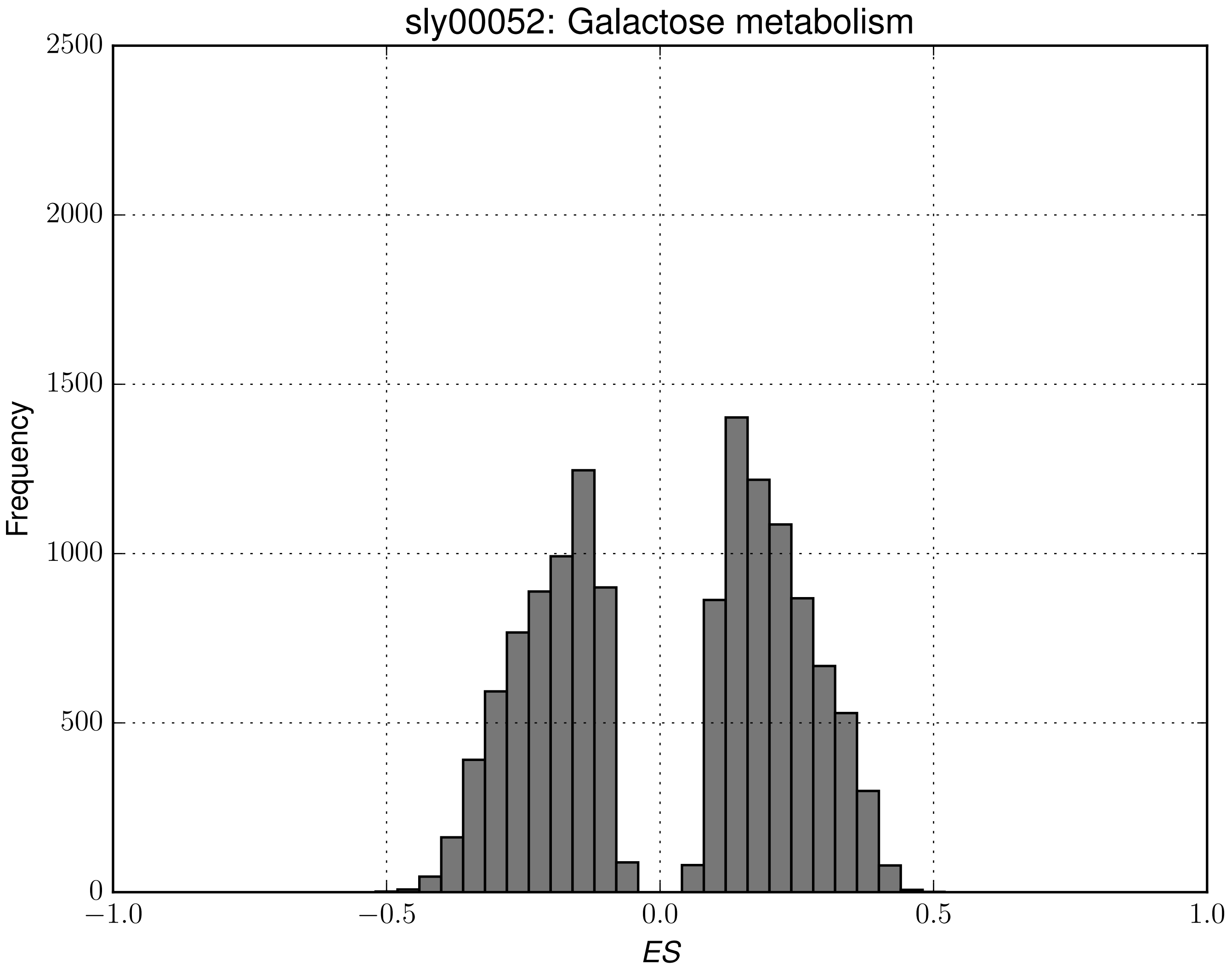

sly00052: Galactose metabolism
Frequency
ES
2500
2000
1500
1000
500
0
−1.0
−0.5
0.0
0.5
1.0

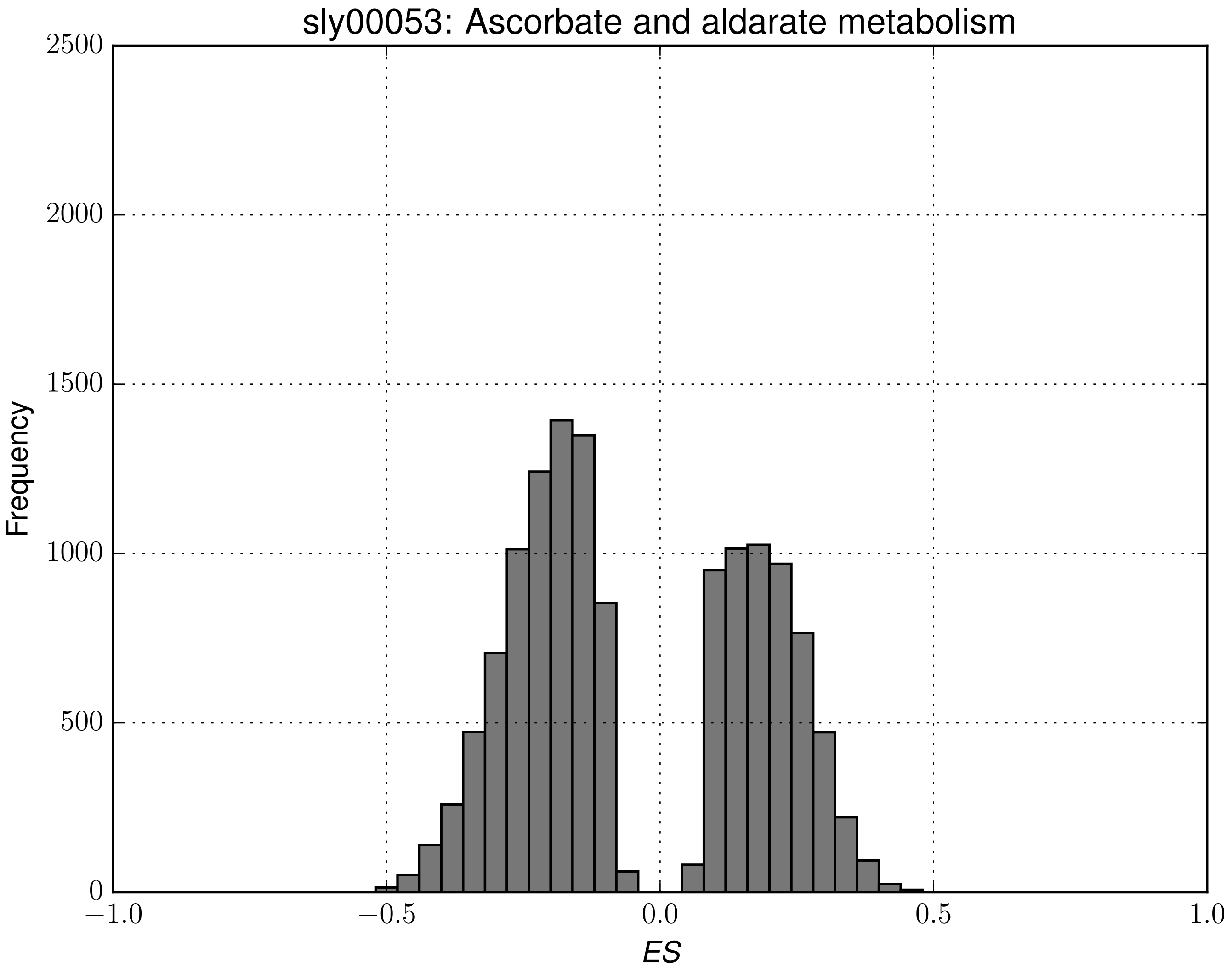

sly00053: Ascorbate and aldarate metabolism
Frequency
ES
2500
2000
1500
1000
500
0
−1.0
−0.5
0.0
0.5
1.0

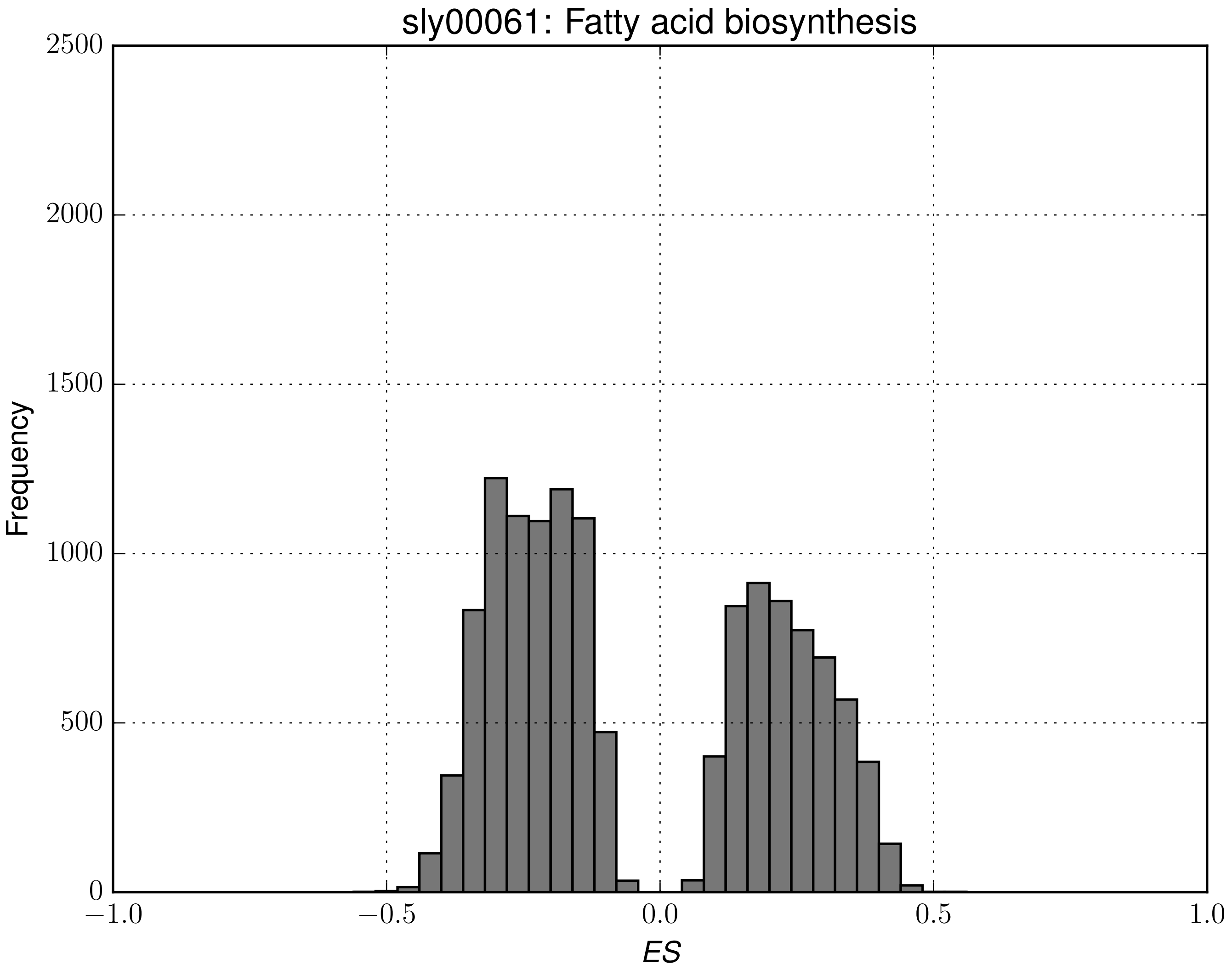
sly00061: Fatty acid biosynthesis
Frequency
ES
2500
2000
1500
1000
500
0
−1.0
−0.5
0.0
0.5
1.0

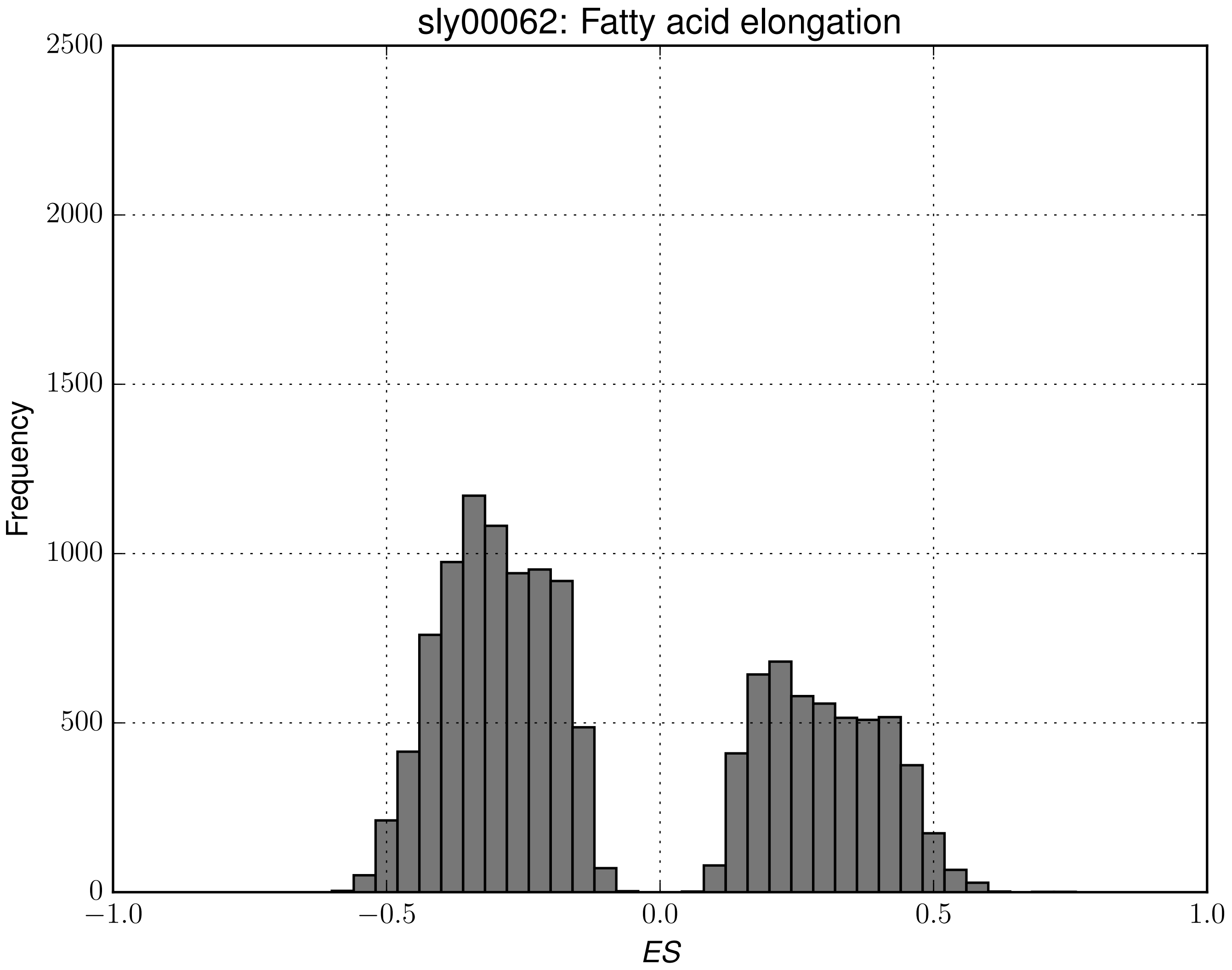
sly00062: Fatty acid elongation
Frequency
2500
2000
1500
1000
500
0
−1.0
−0.5
0.0
0.5
1.0
ES

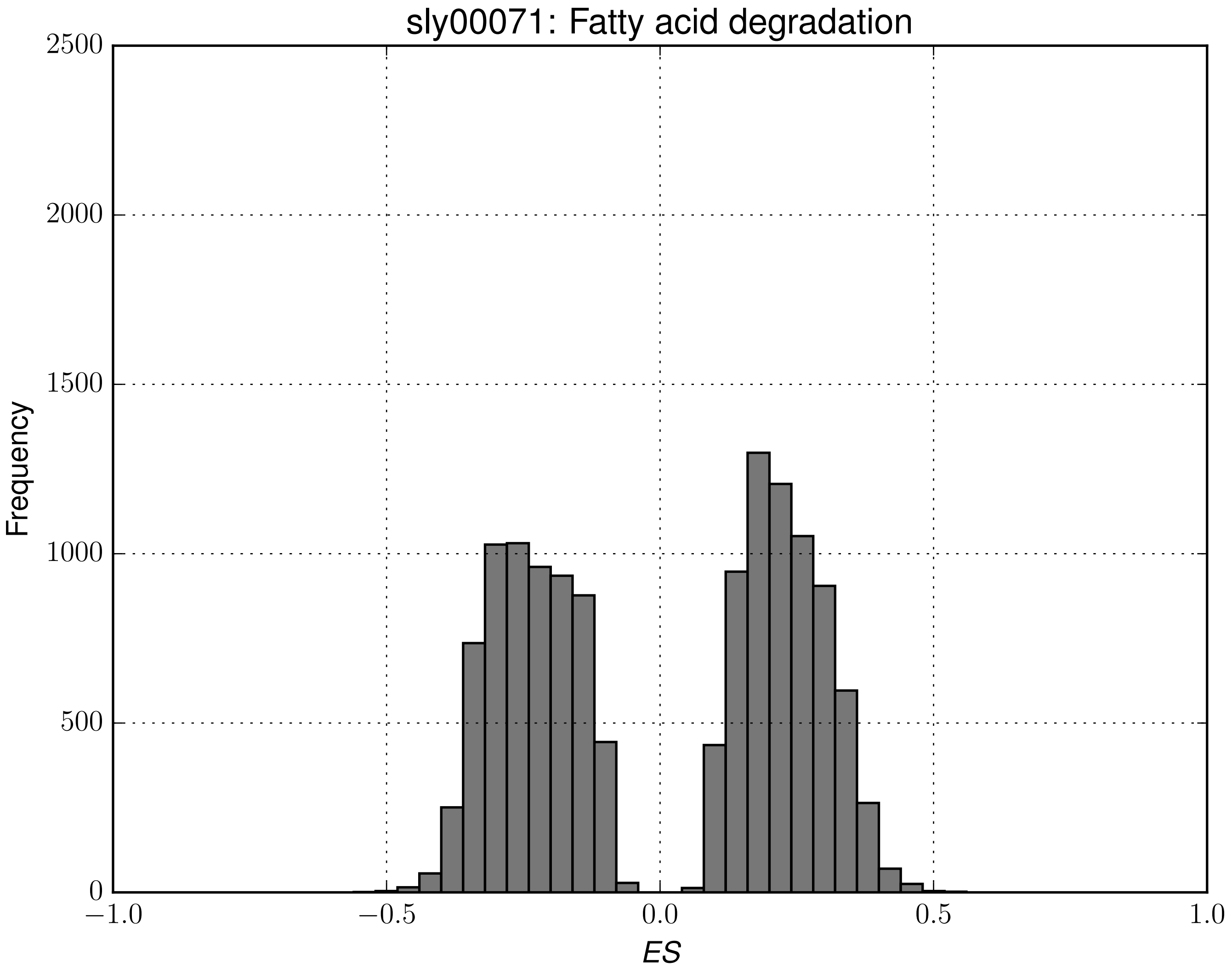

sly00071: Fatty acid degradation
Frequency
ES
0
500
1000
1500
2000
2500
−1.0
−0.5
0.0
0.5
1.0

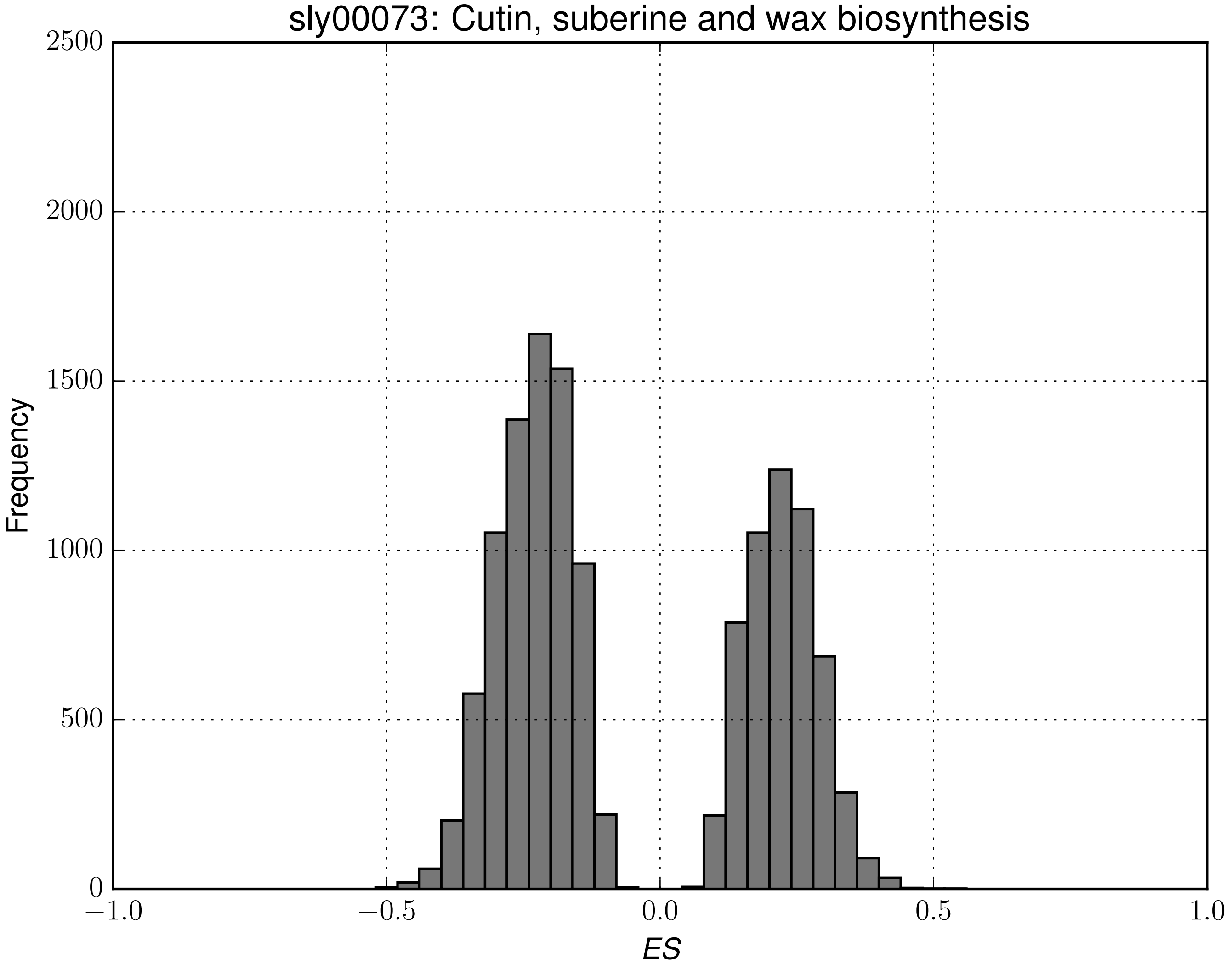
sly00073: Cutin, suberine and wax biosynthesis
Frequency
2500
2000
1500
1000
500
0
−1.0
−0.5
0.0
0.5
1.0
ES

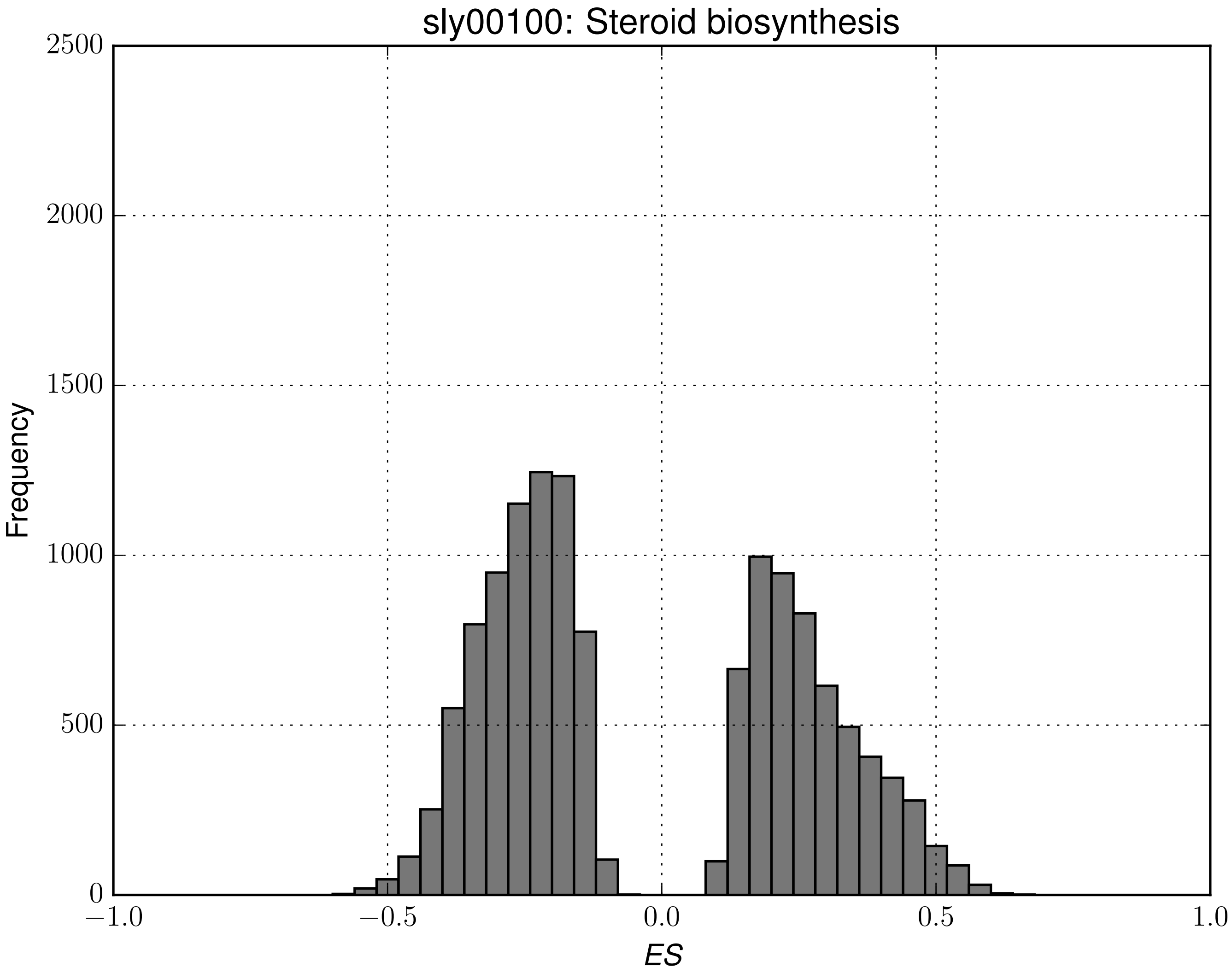
sly00100: Steroid biosynthesis
Frequency
ES
2500
2000
1500
1000
500
0
−1.0
−0.5
0.0
0.5
1.0

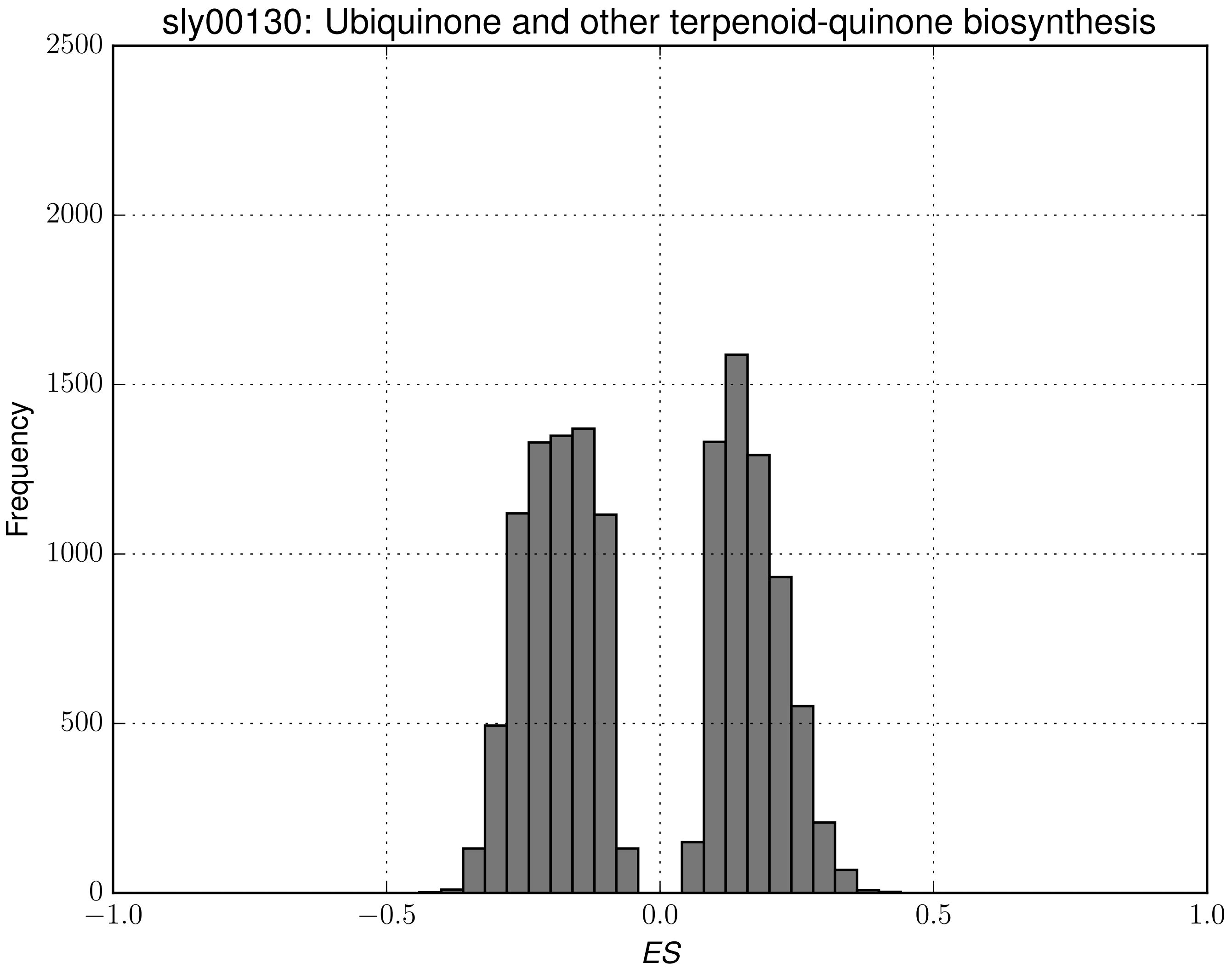
sly00130: Ubiquinone and other terpenoid-quinone biosynthesis
Frequency
ES
0
500
1000
1500
2000
2500
−1.0
−0.5
0.0
0.5
1.0

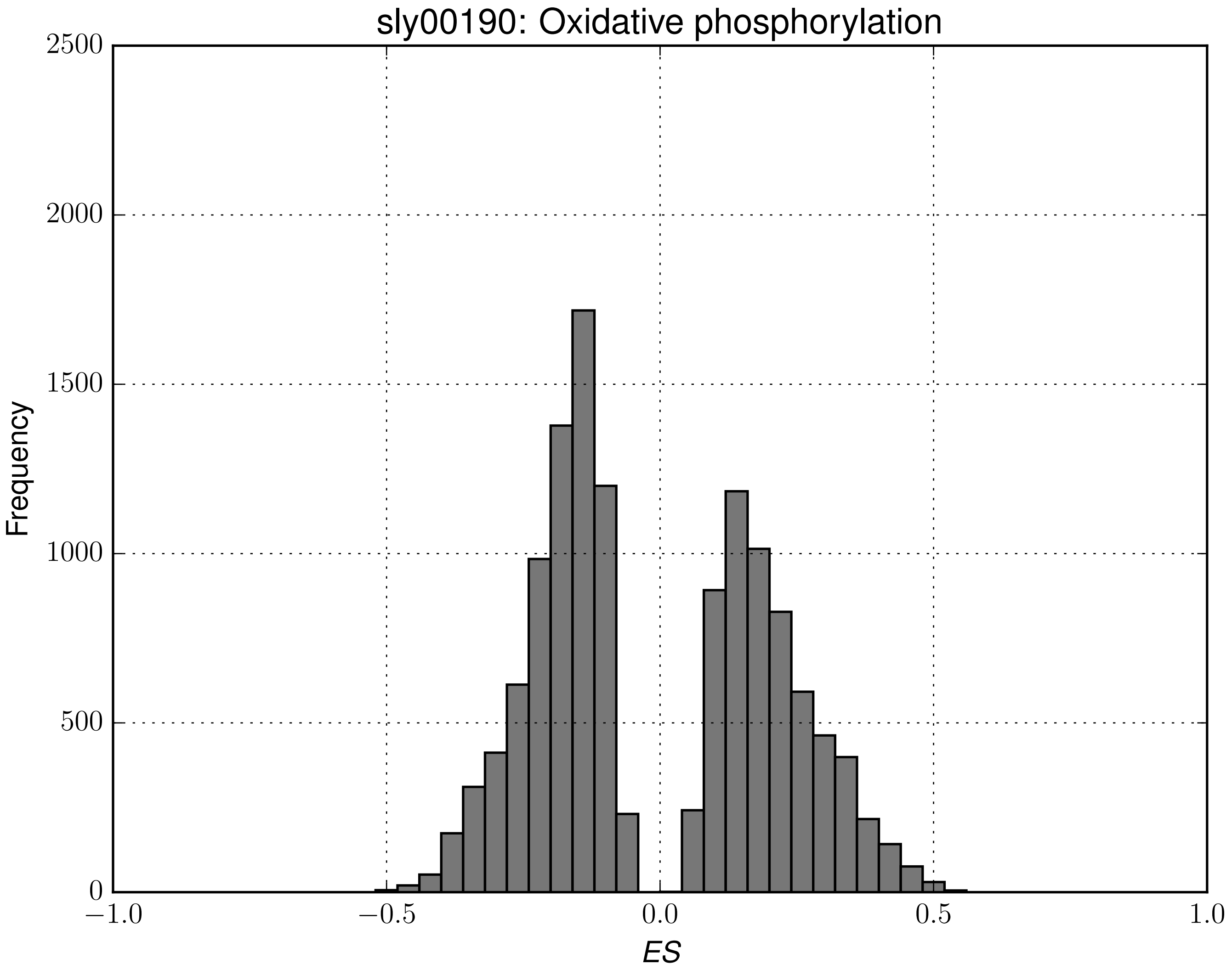

sly00190: Oxidative phosphorylation
2500
2000
1500
1000
500
0
Frequency
−1.0
−0.5
0.0
0.5
1.0
ES

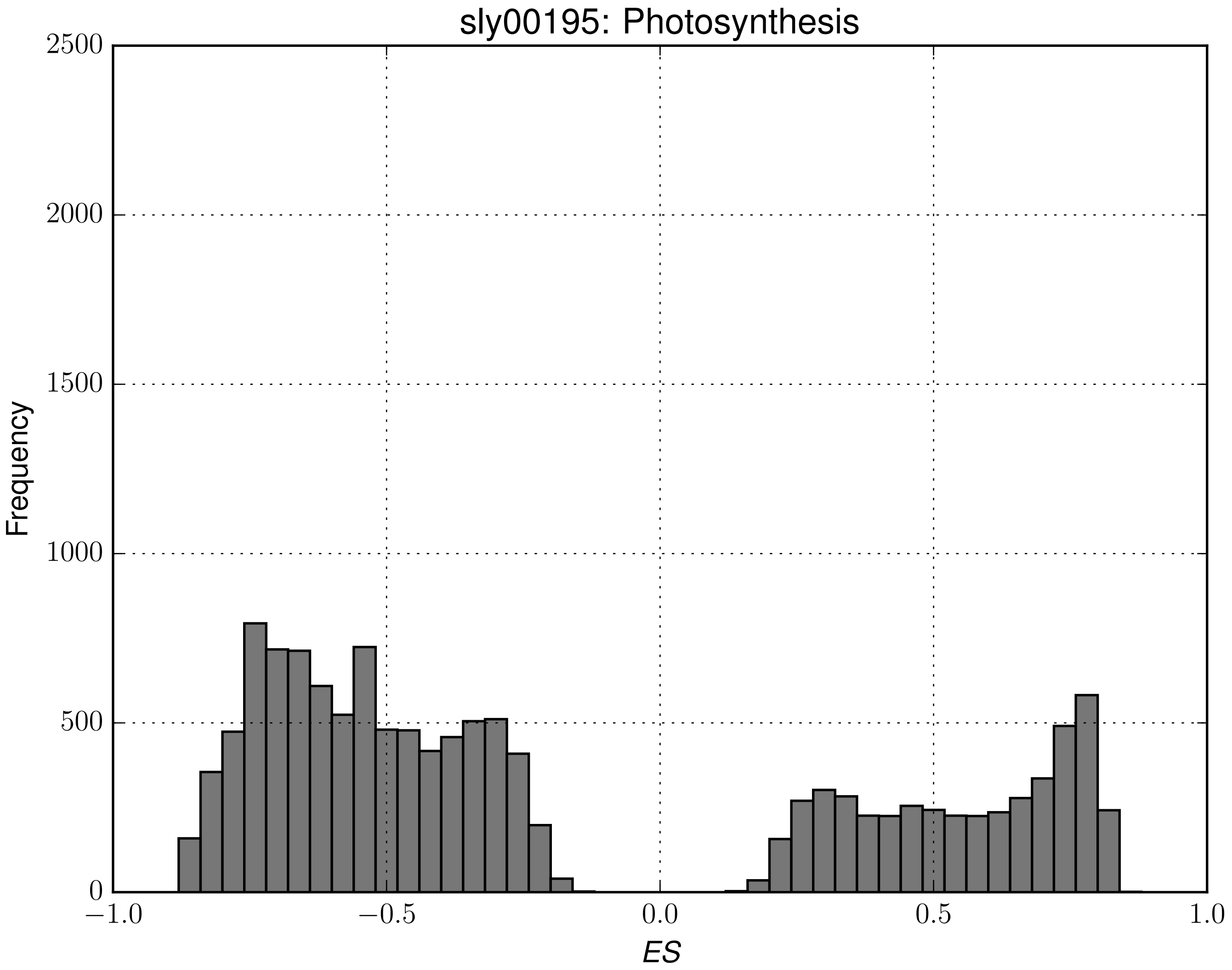
sly00195: Photosynthesis
Frequency
ES
2500
2000
1500
1000
500
0
−1.0
−0.5
0.0
0.5
1.0

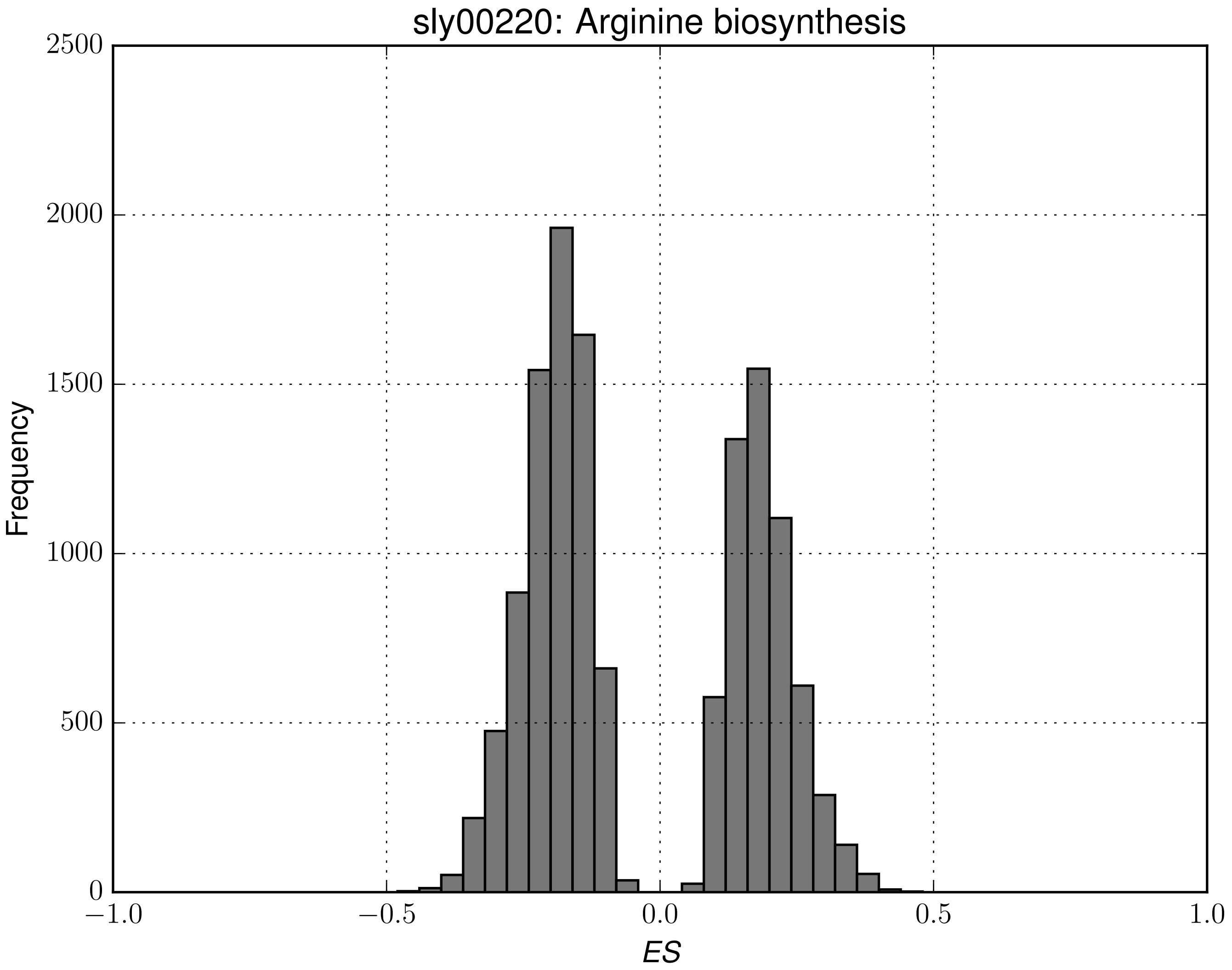
sly00220: Arginine biosynthesis
Frequency
ES
2500
2000
1500
1000
500
0
−1.0
−0.5
0.0
0.5
1.0

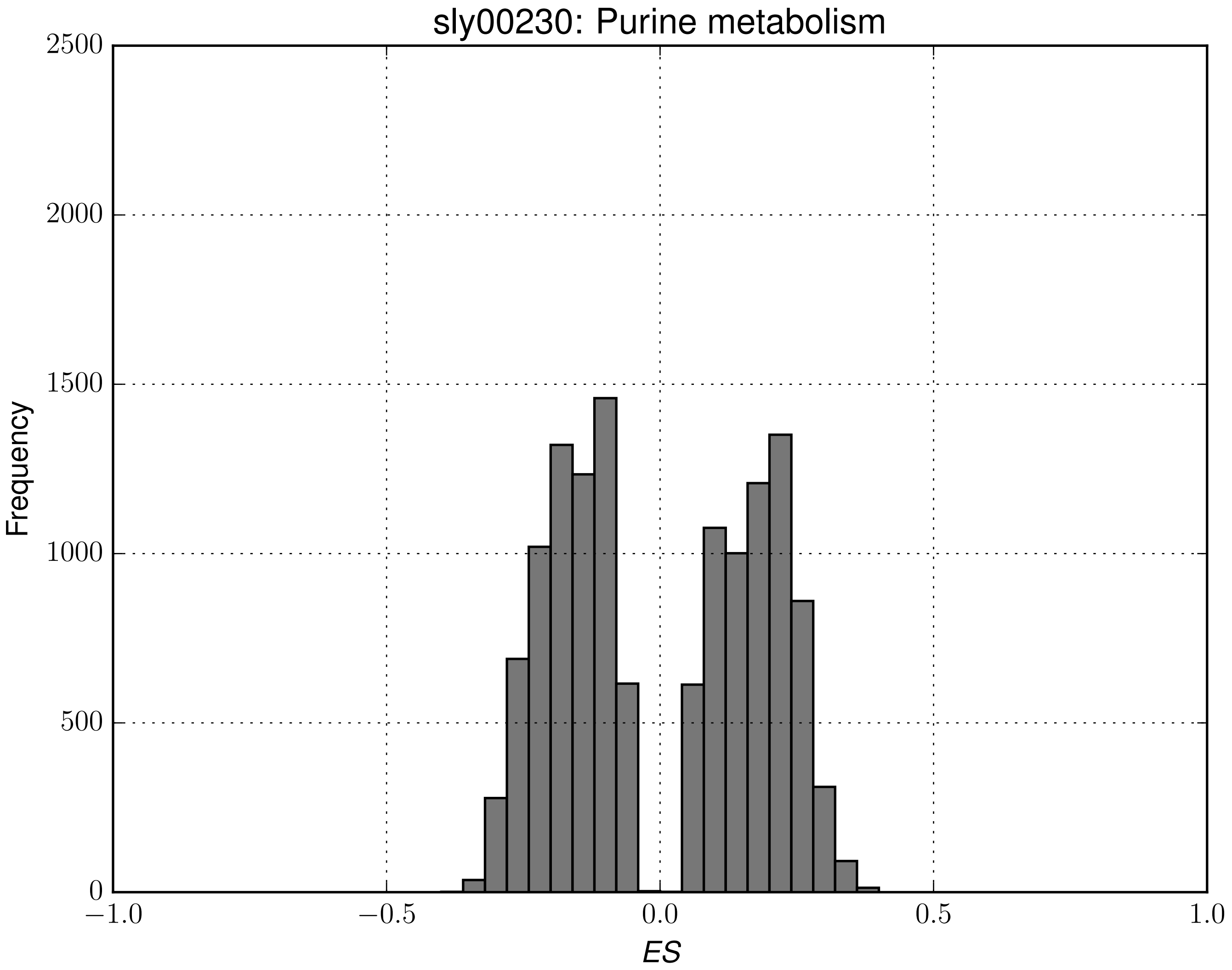
sly00230: Purine metabolism
2500
2000
1500
1000
500
0
Frequency
−1.0
−0.5
0.0
0.5
1.0
ES

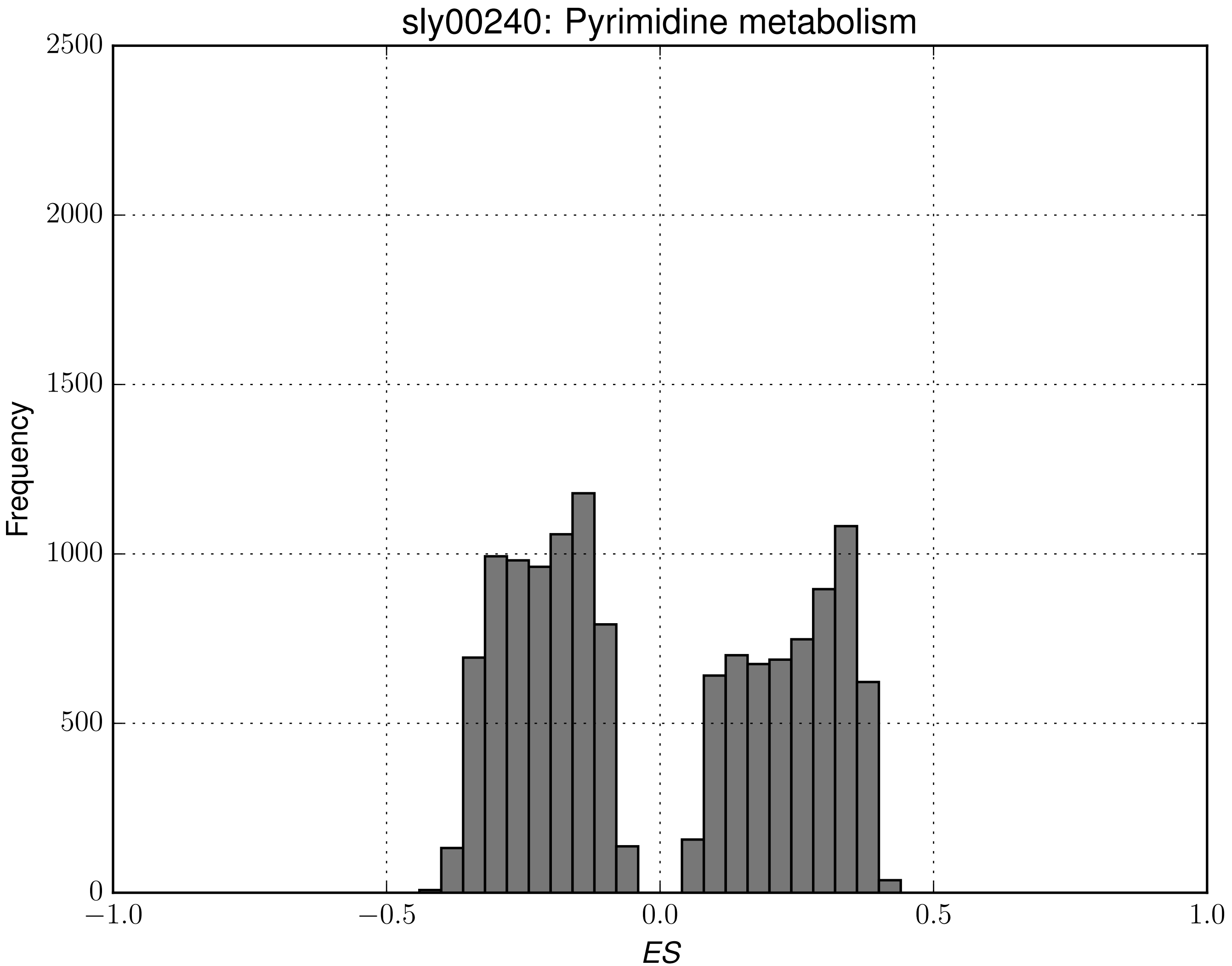
sly00240: Pyrimidine metabolism
2500
2000
1500
1000
500
0
Frequency
−1.0
−0.5
0.0
0.5
1.0
ES

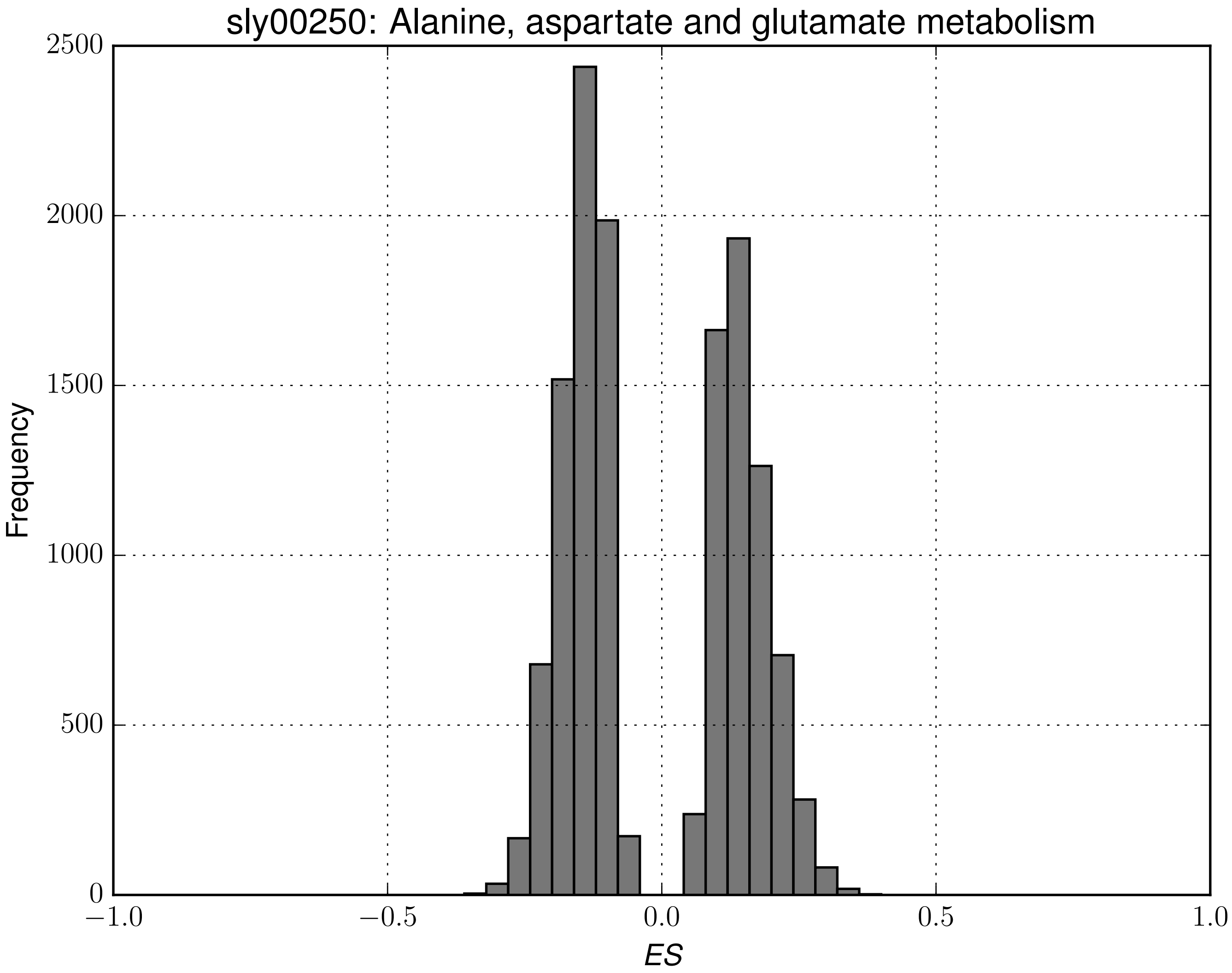
sly00250: Alanine, aspartate and glutamate metabolism
Frequency
ES
2500
2000
1500
1000
500
0
−1.0
−0.5
0.0
0.5
1.0

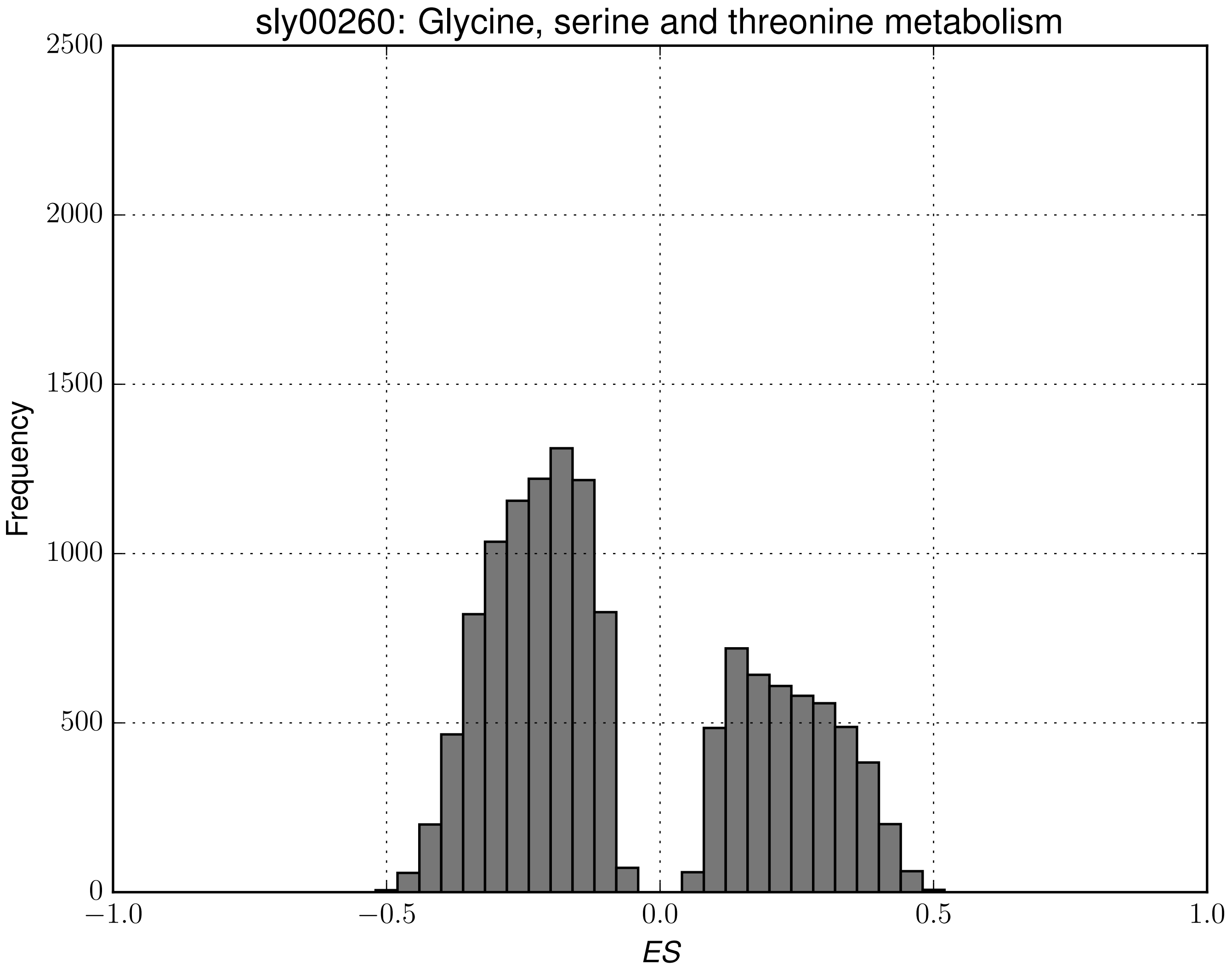
sly00260: Glycine, serine and threonine metabolism
Frequency
ES
2500
2000
1500
1000
500
0
−1.0
−0.5
0.0
0.5
1.0

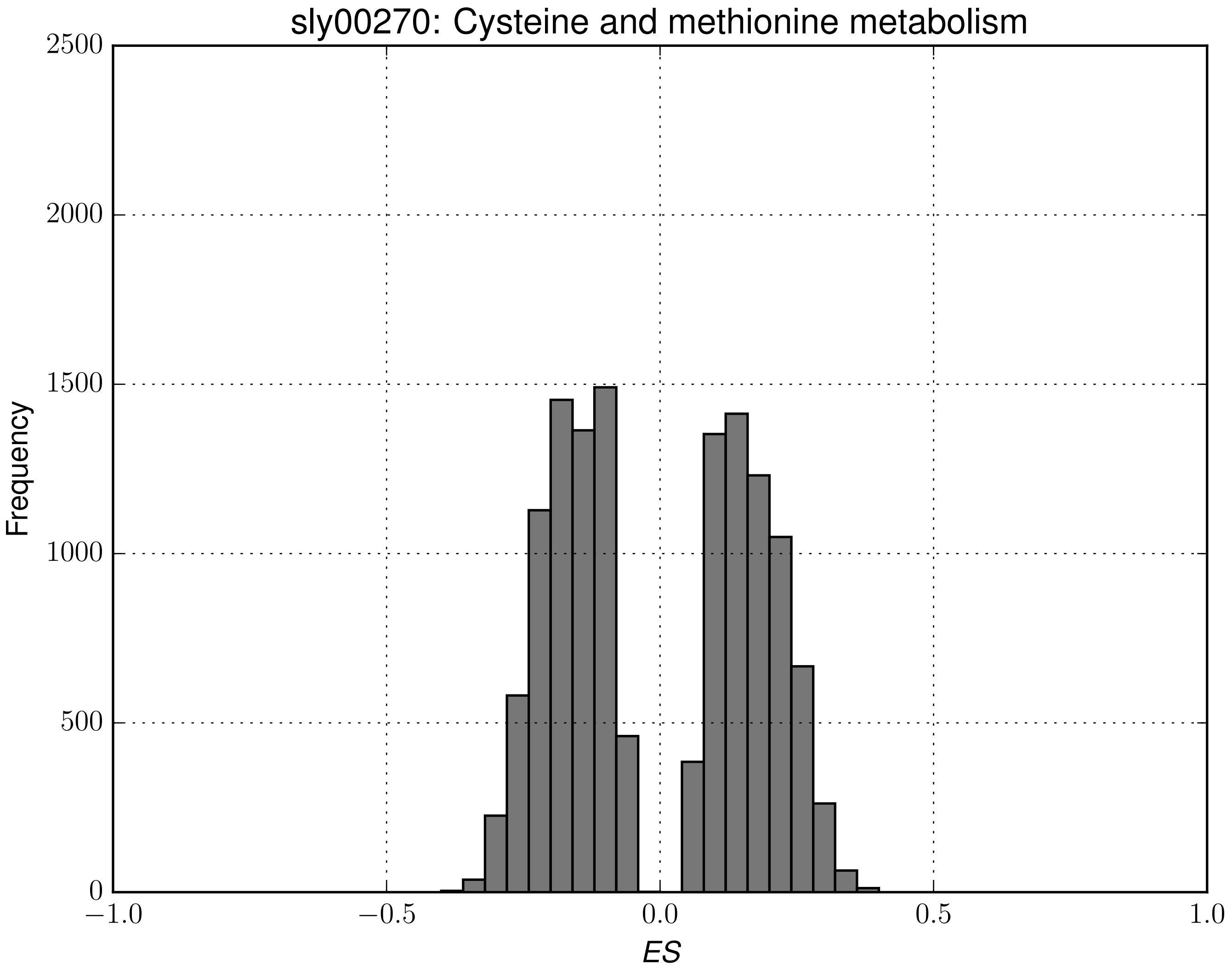

sly00270: Cysteine and methionine metabolism
Frequency
ES
2500
2000
1500
1000
500
0
−1.0
−0.5
0.0
0.5
1.0

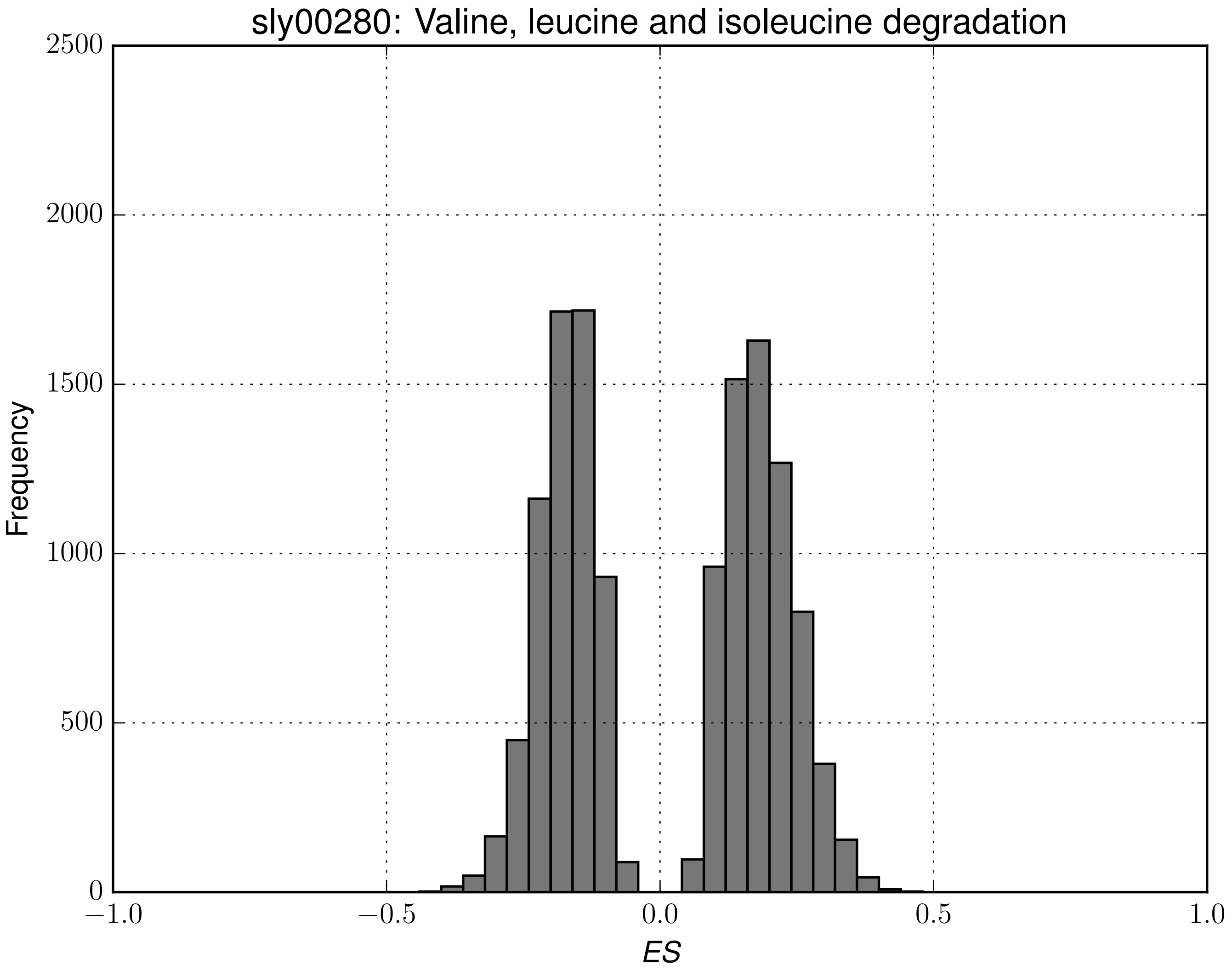
sly00280: Valine, leucine and isoleucine degradation
Frequency
ES
2500
2000
1500
1000
500
0
−1.0
−0.5
0.0
0.5
1.0

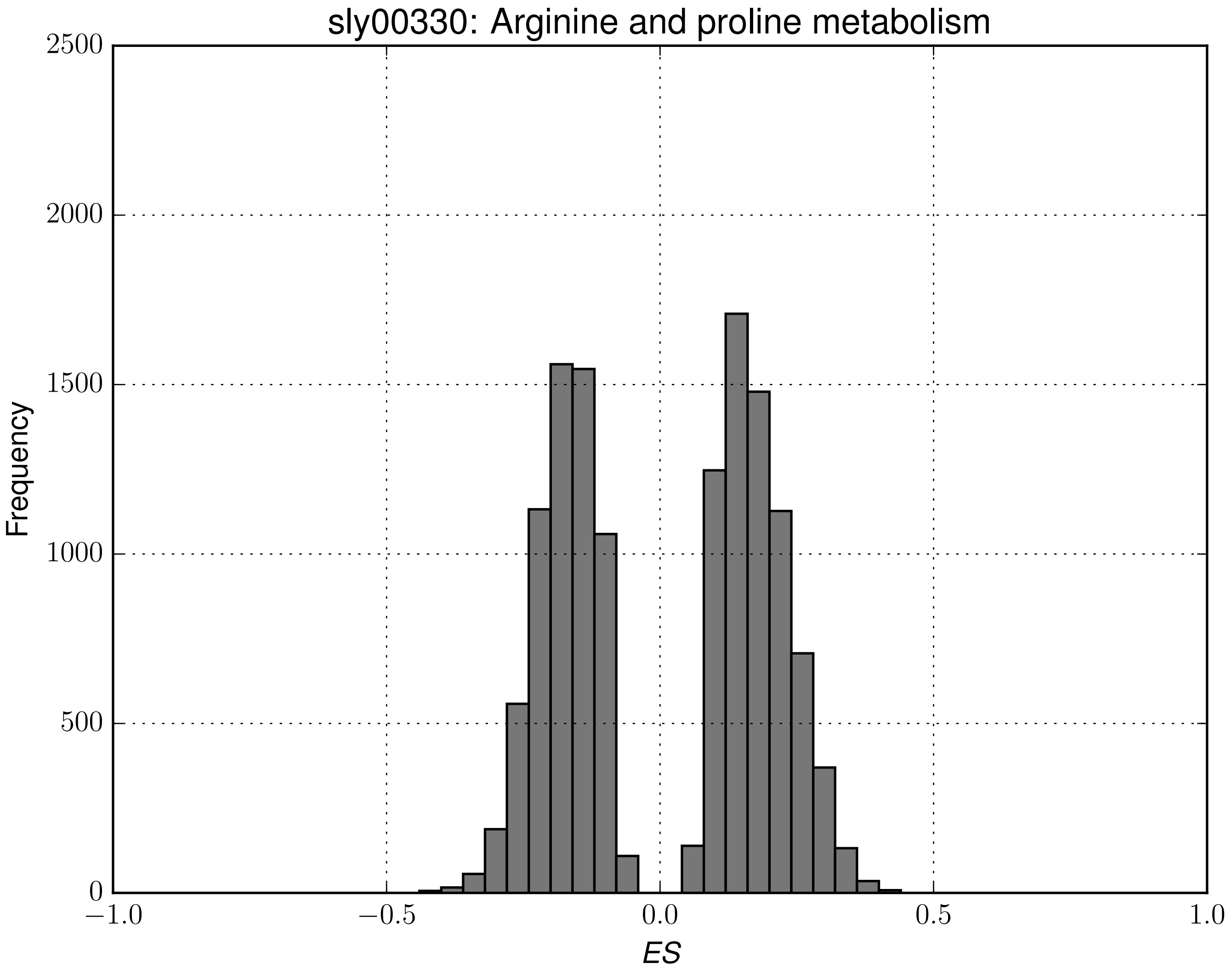
sly00330: Arginine and proline metabolism
2500
2000
1500
1000
500
0
Frequency
−1.0
−0.5
0.0
0.5
1.0
ES

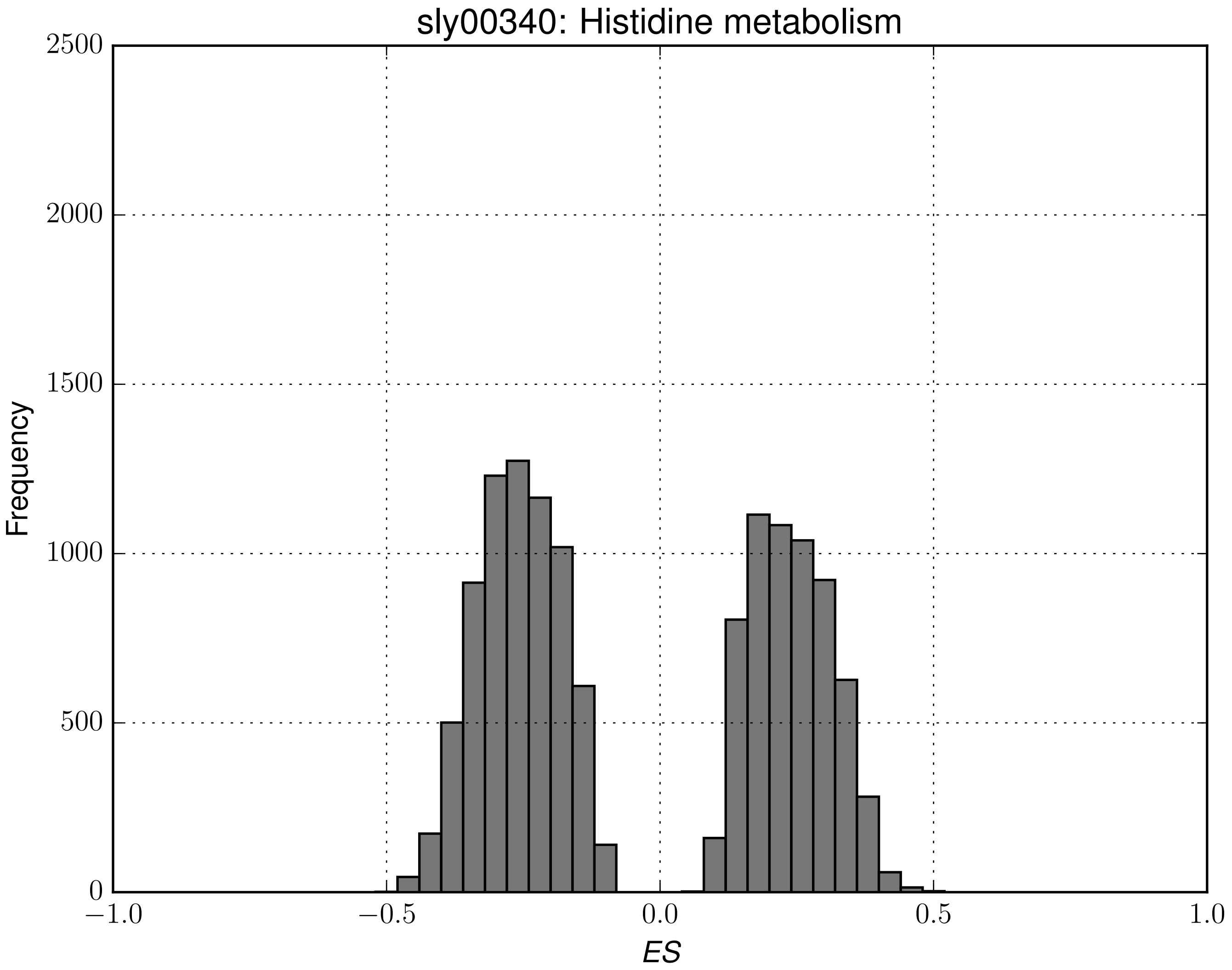

sly00340: Histidine metabolism
Frequency
ES
2500
2000
1500
1000
500
0
−1.0
−0.5
0.0
0.5
1.0

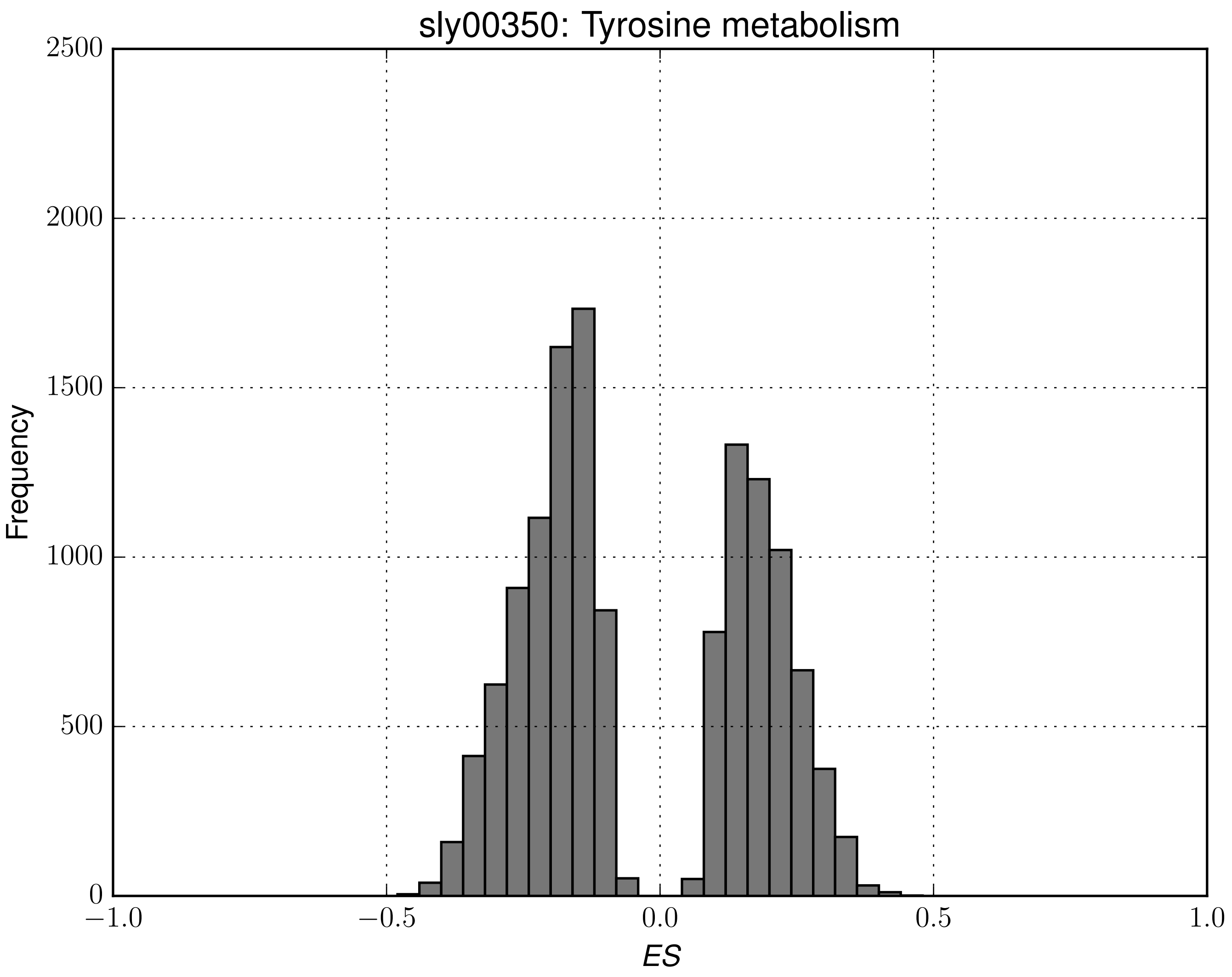

sly00350: Tyrosine metabolism
Frequency
ES
2500
2000
1500
1000
500
0
−1.0
−0.5
0.0
0.5
1.0

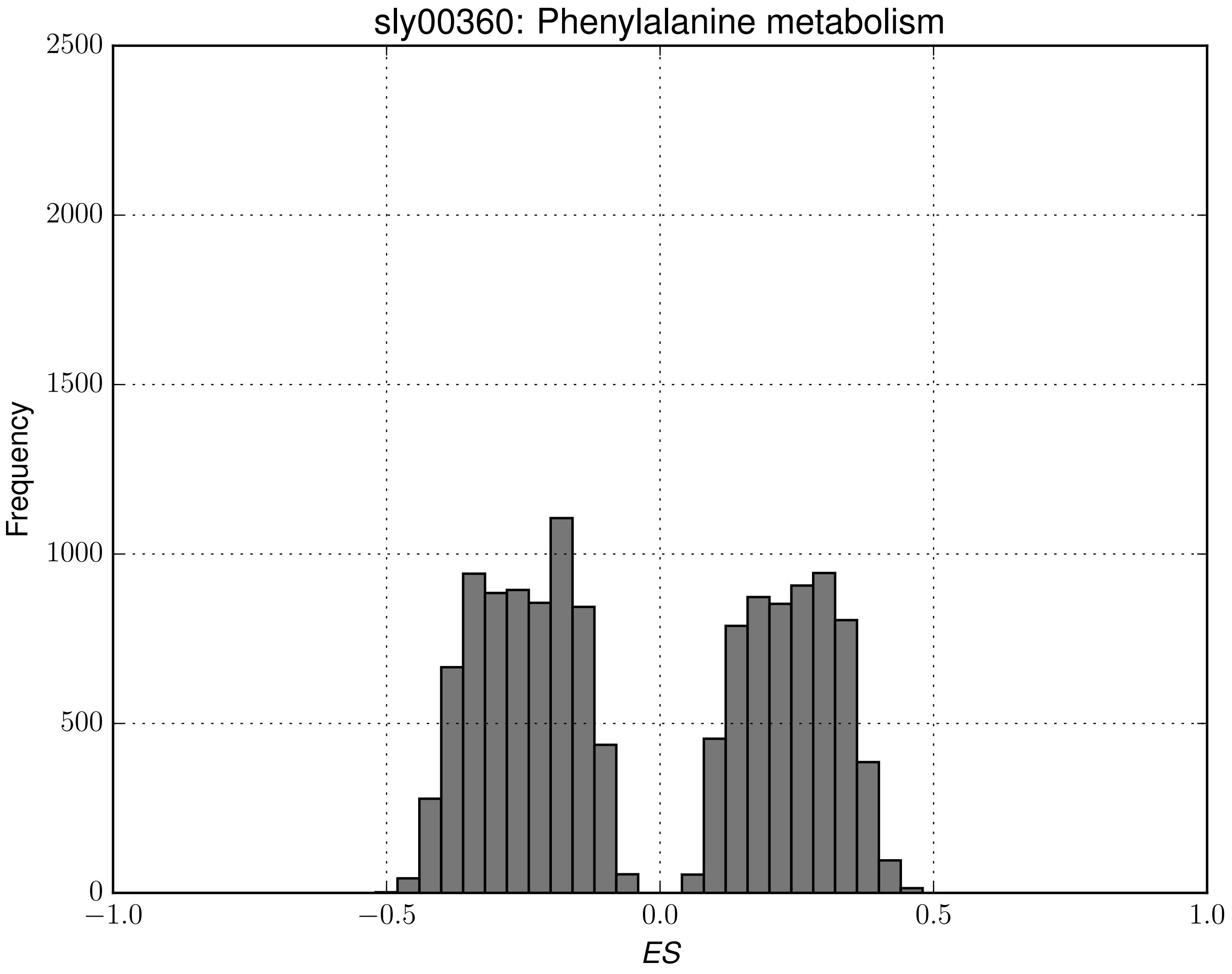

sly00360: Phenylalanine metabolism
Frequency
ES
2500
2000
1500
1000
500
0
−1.0
−0.5
0.0
0.5
1.0

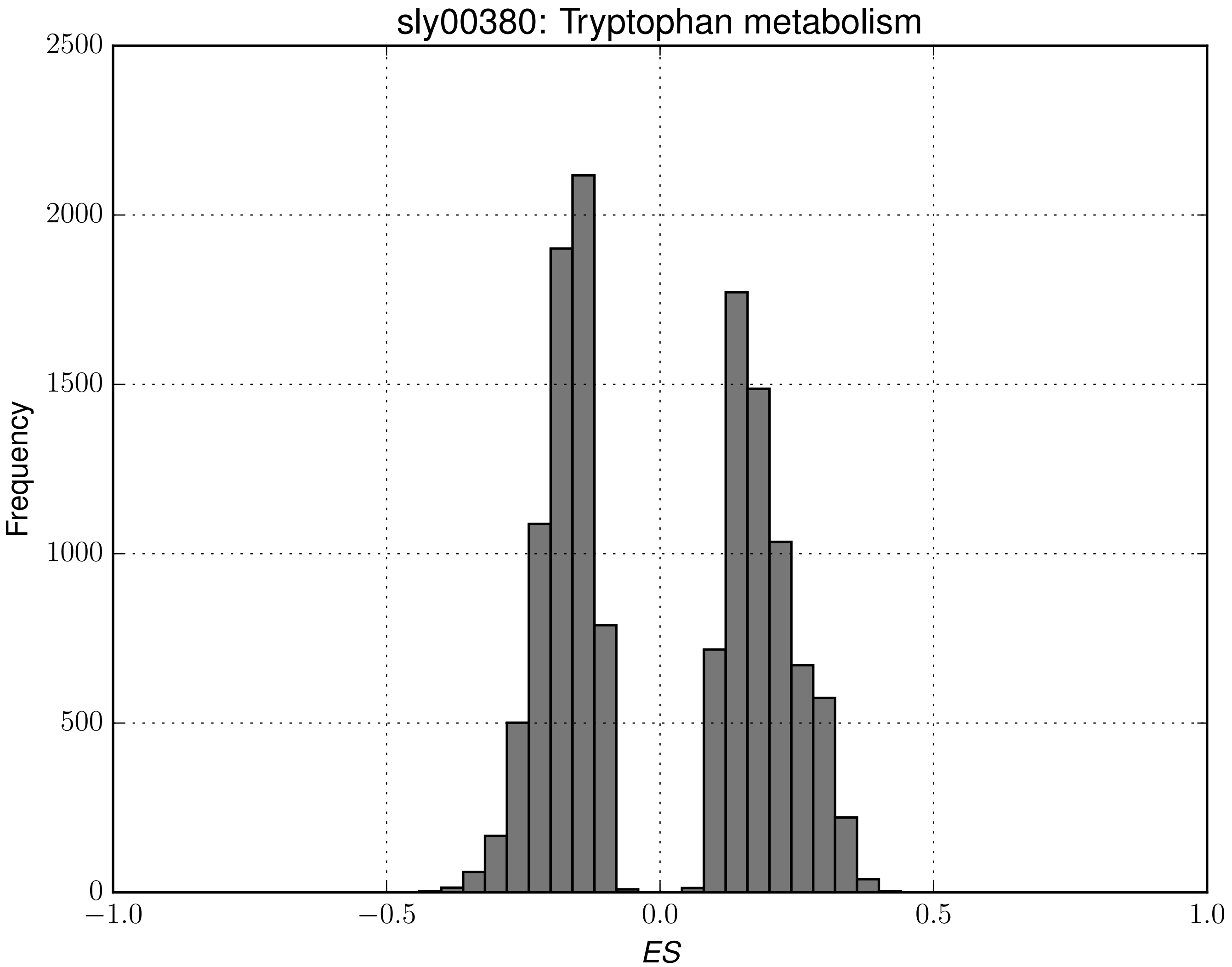

sly00380: Tryptophan metabolism
2500
2000
1500
1000
500
0
Frequency
−1.0
−0.5
0.0
0.5
1.0
ES

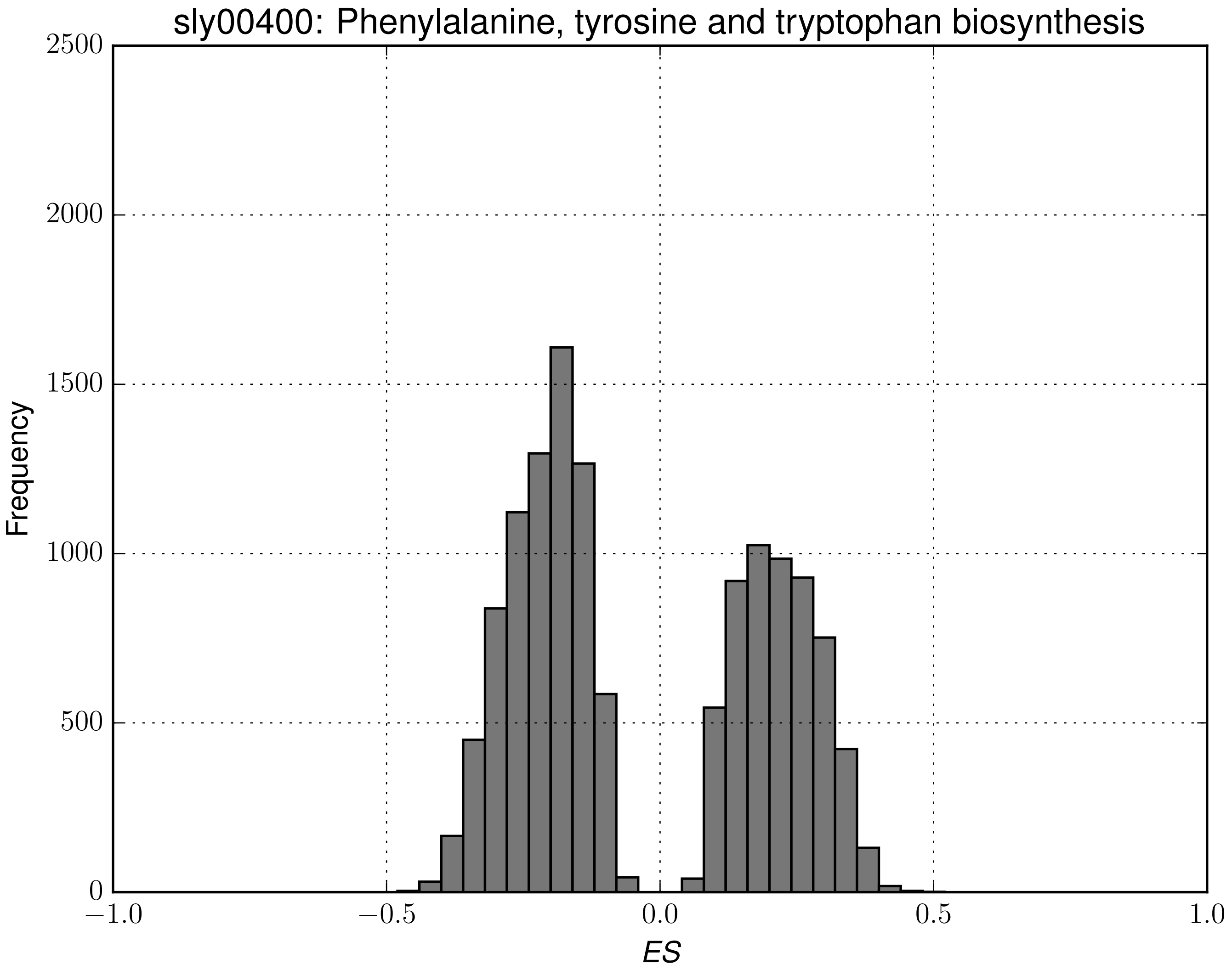

sly00400: Phenylalanine, tyrosine and tryptophan biosynthesis
2500
2000
1500
1000
500
0
Frequency
−1.0
−0.5
0.0
0.5
1.0
ES

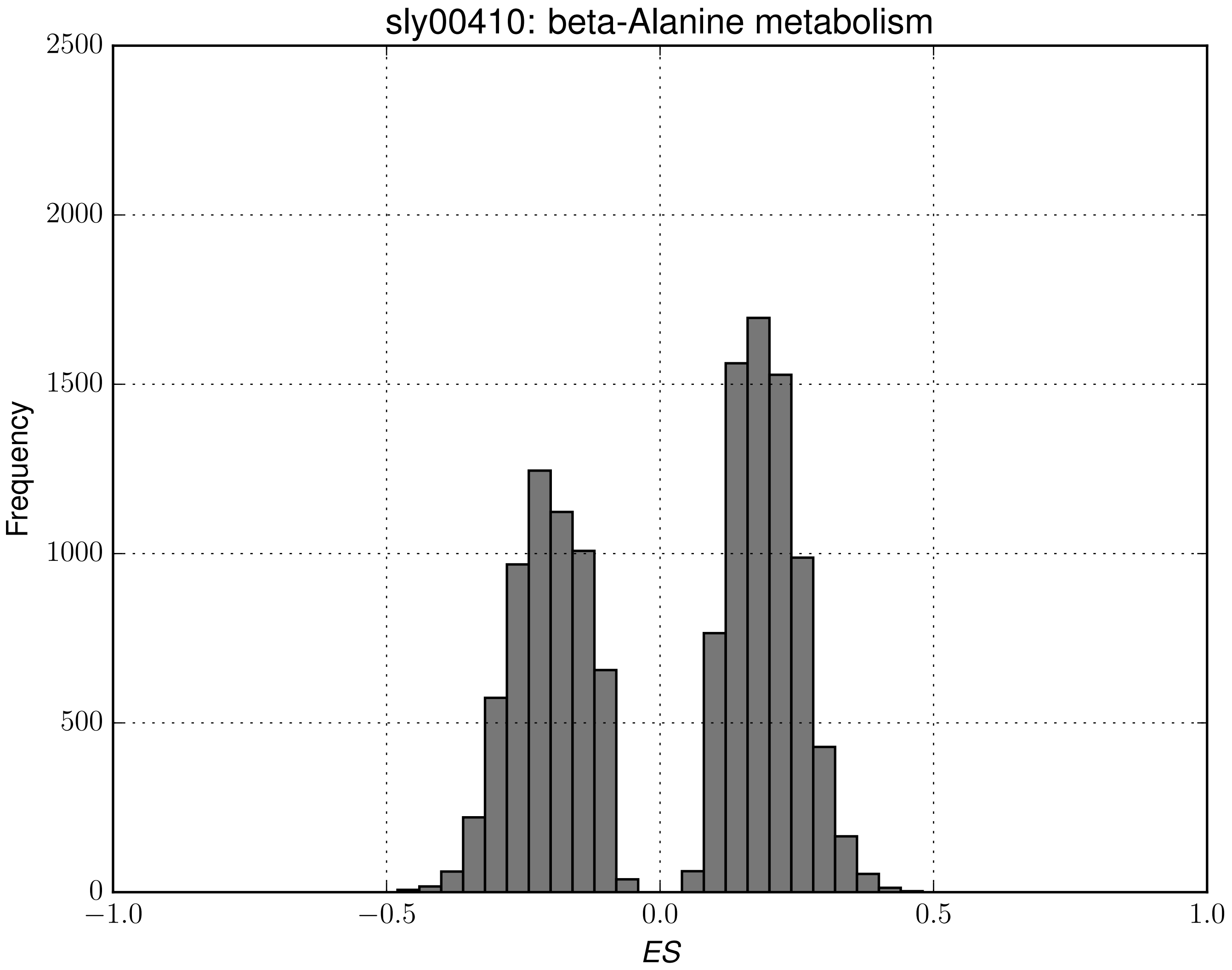

sly00410: beta-Alanine metabolism
Frequency
ES
2500
2000
1500
1000
500
0
−1.0
−0.5
0.0
0.5
1.0

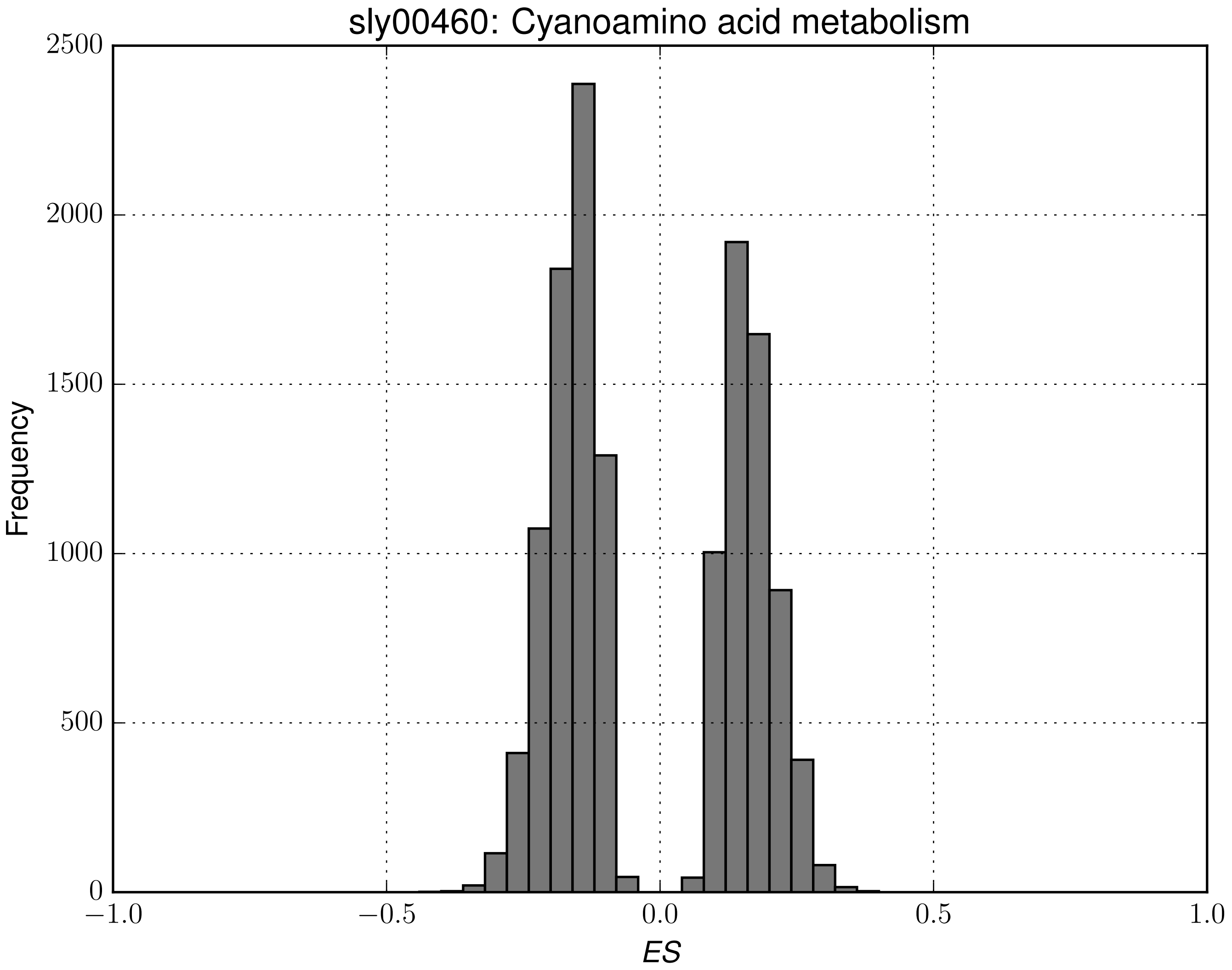

sly00460: Cyanoamino acid metabolism
Frequency
ES
2500
2000
1500
1000
500
0
−1.0
−0.5
0.0
0.5
1.0

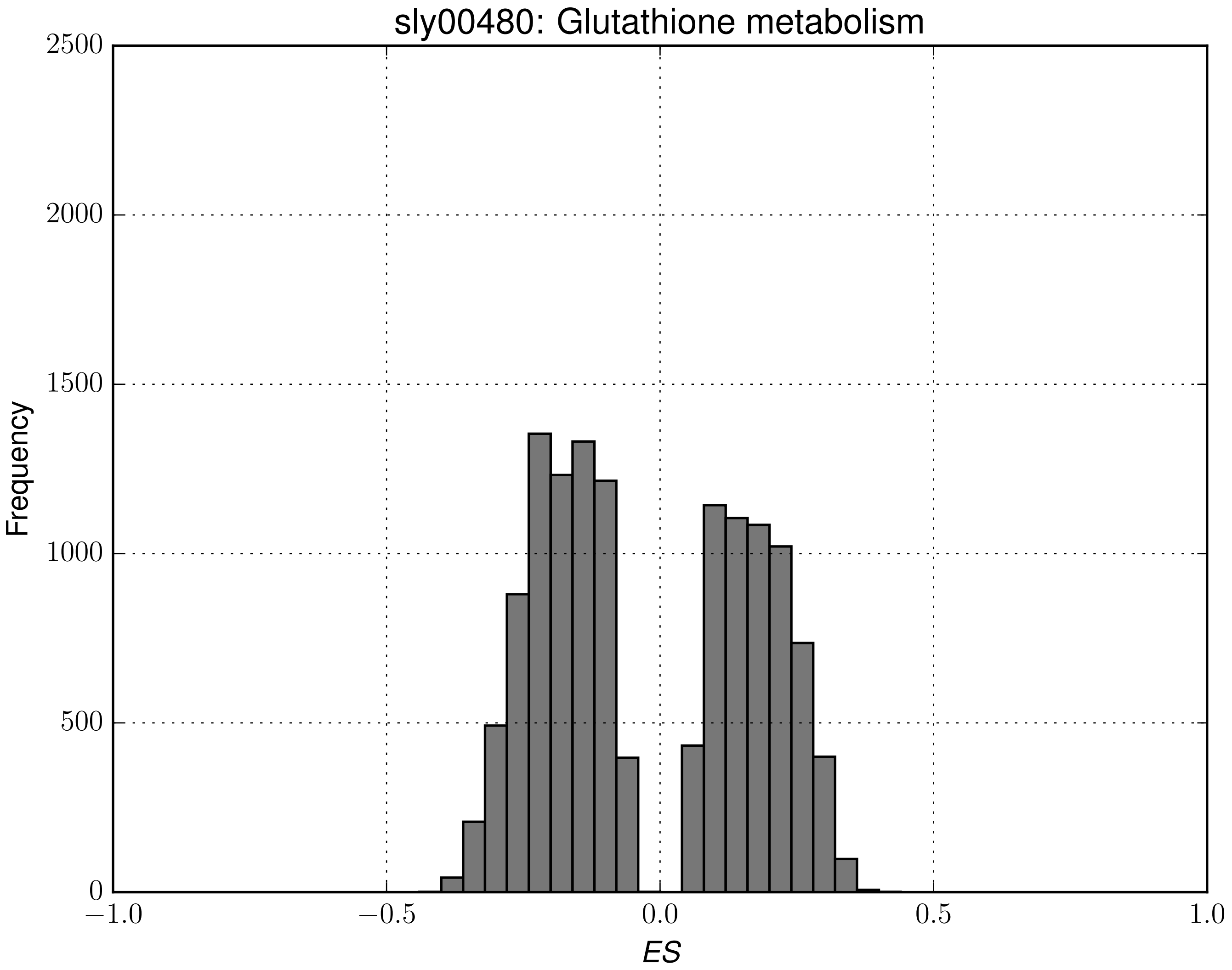

sly00480: Glutathione metabolism
Frequency
ES
2500
2000
1500
1000
500
0
−1.0
−0.5
0.0
0.5
1.0

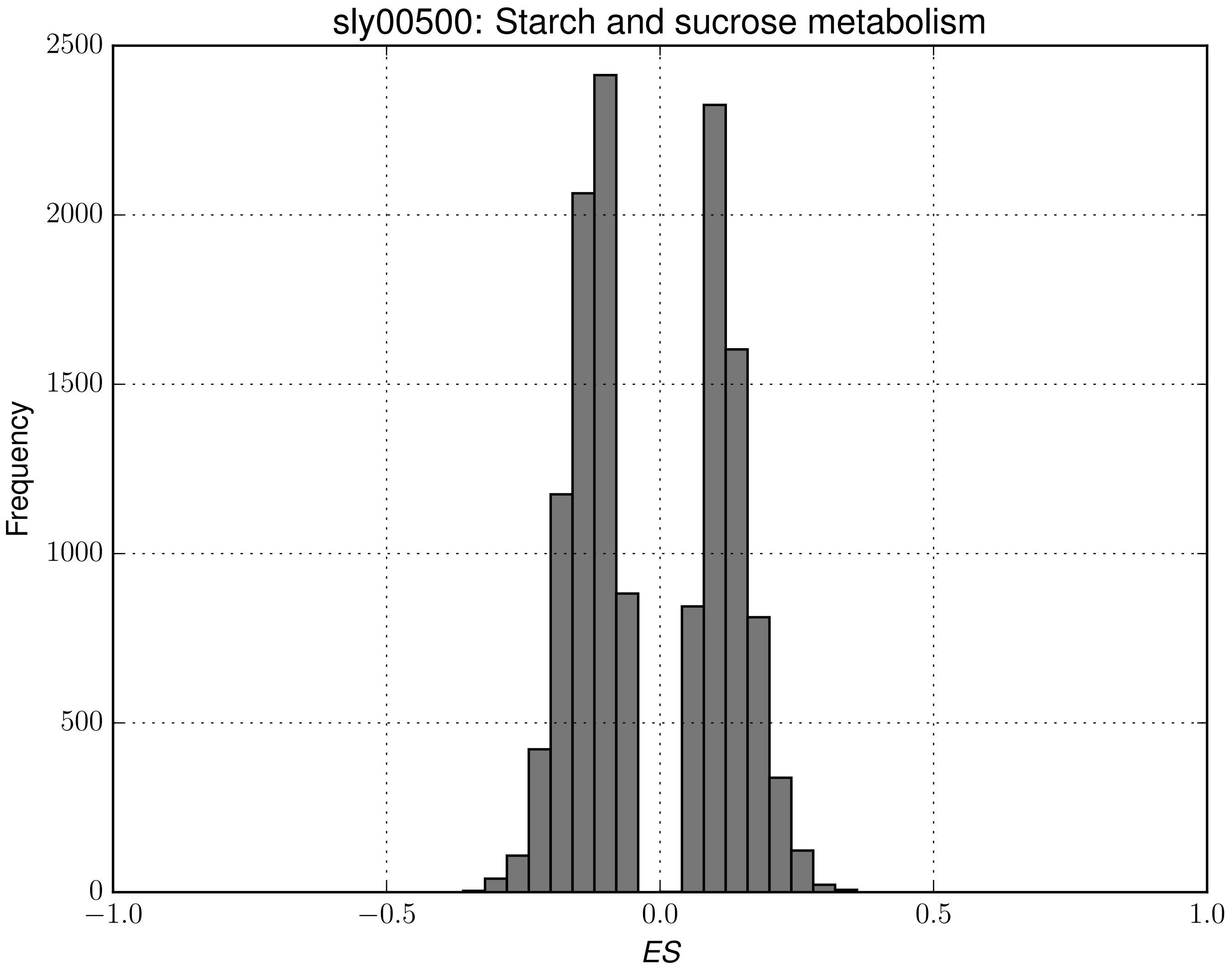
sly00500: Starch and sucrose metabolism
Frequency
ES
0
500
1000
1500
2000
2500
−1.0
−0.5
0.0
0.5
1.0

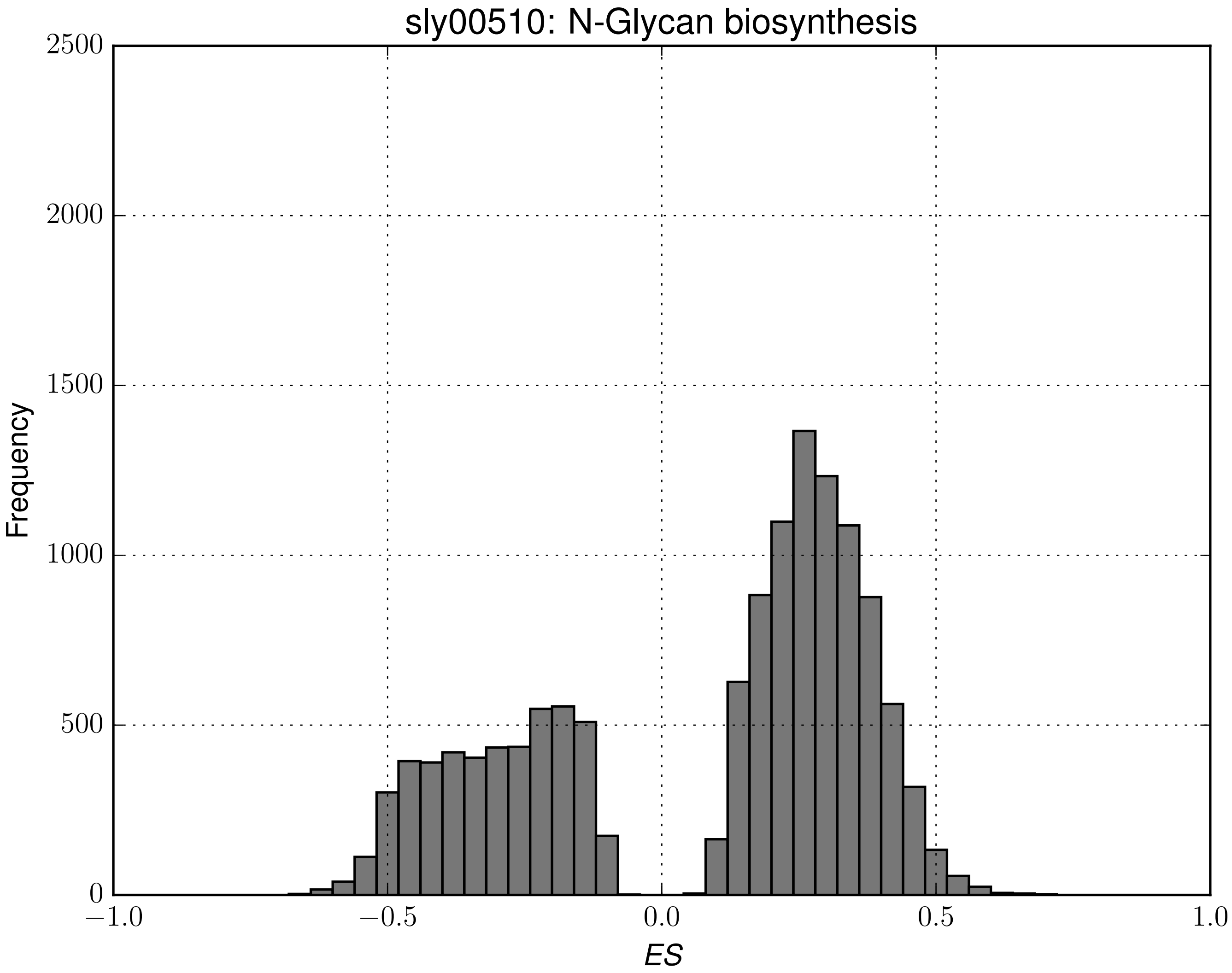

sly00510: N-Glycan biosynthesis
Frequency
ES
2500
2000
1500
1000
500
0
−1.0
−0.5
0.0
0.5
1.0

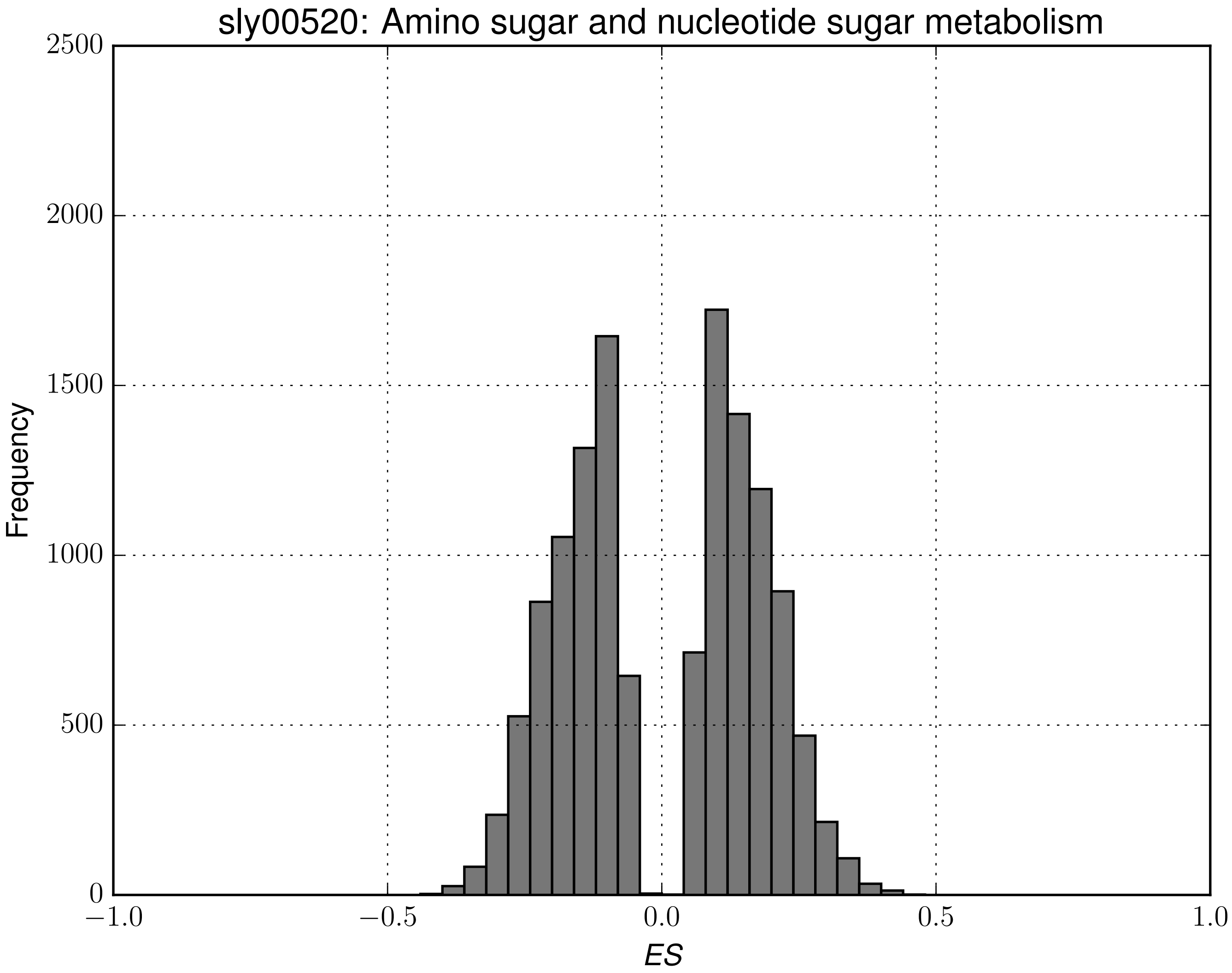

sly00520: Amino sugar and nucleotide sugar metabolism
Frequency
2500
2000
1500
1000
500
0
−1.0
−0.5
0.0
0.5
1.0
ES

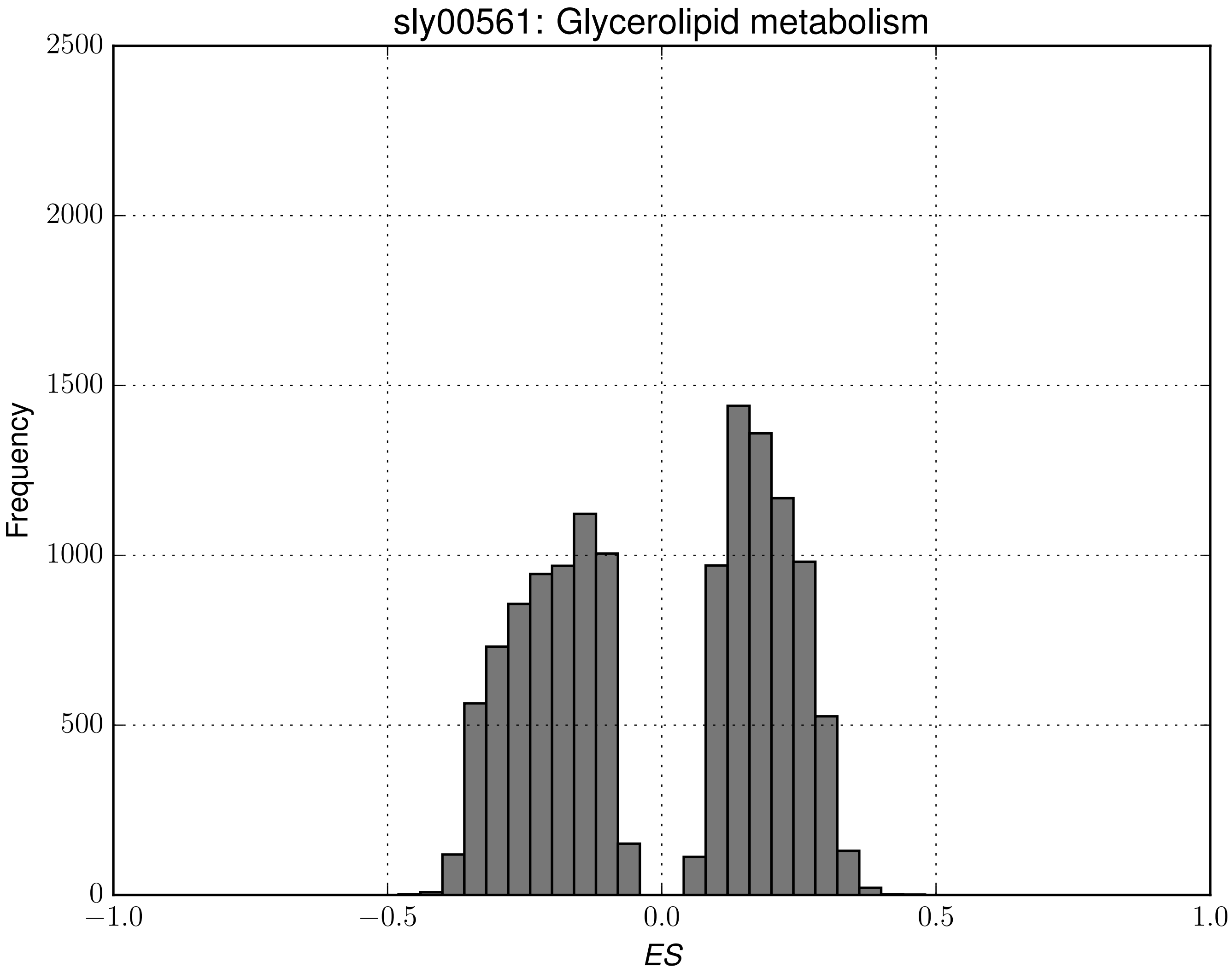
sly00561: Glycerolipid metabolism
Frequency
ES
2500
2000
1500
1000
500
0
−1.0
−0.5
0.0
0.5
1.0

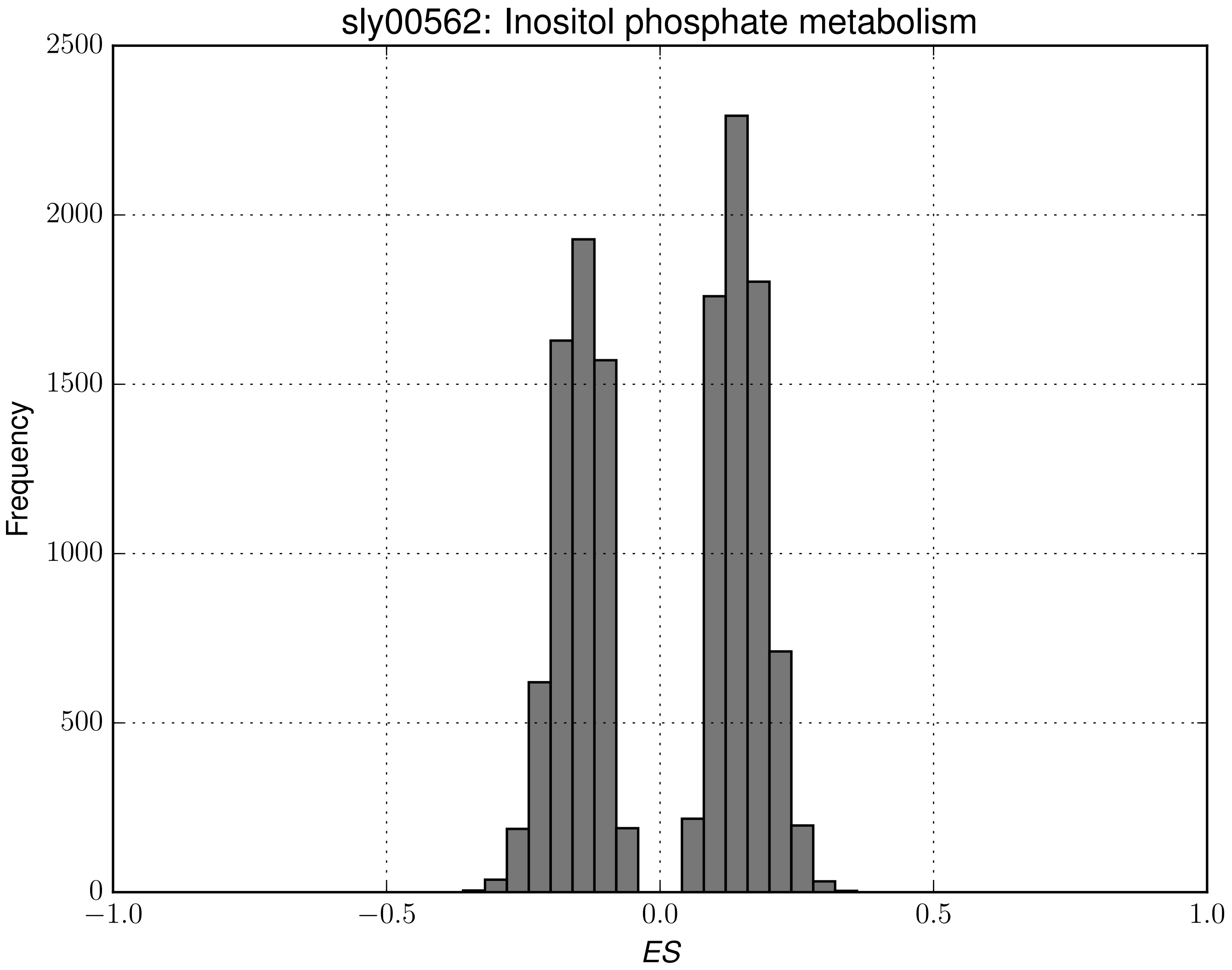
sly00562: Inositol phosphate metabolism
Frequency
ES
2500
2000
1500
1000
500
0
−1.0
−0.5
0.0
0.5
1.0

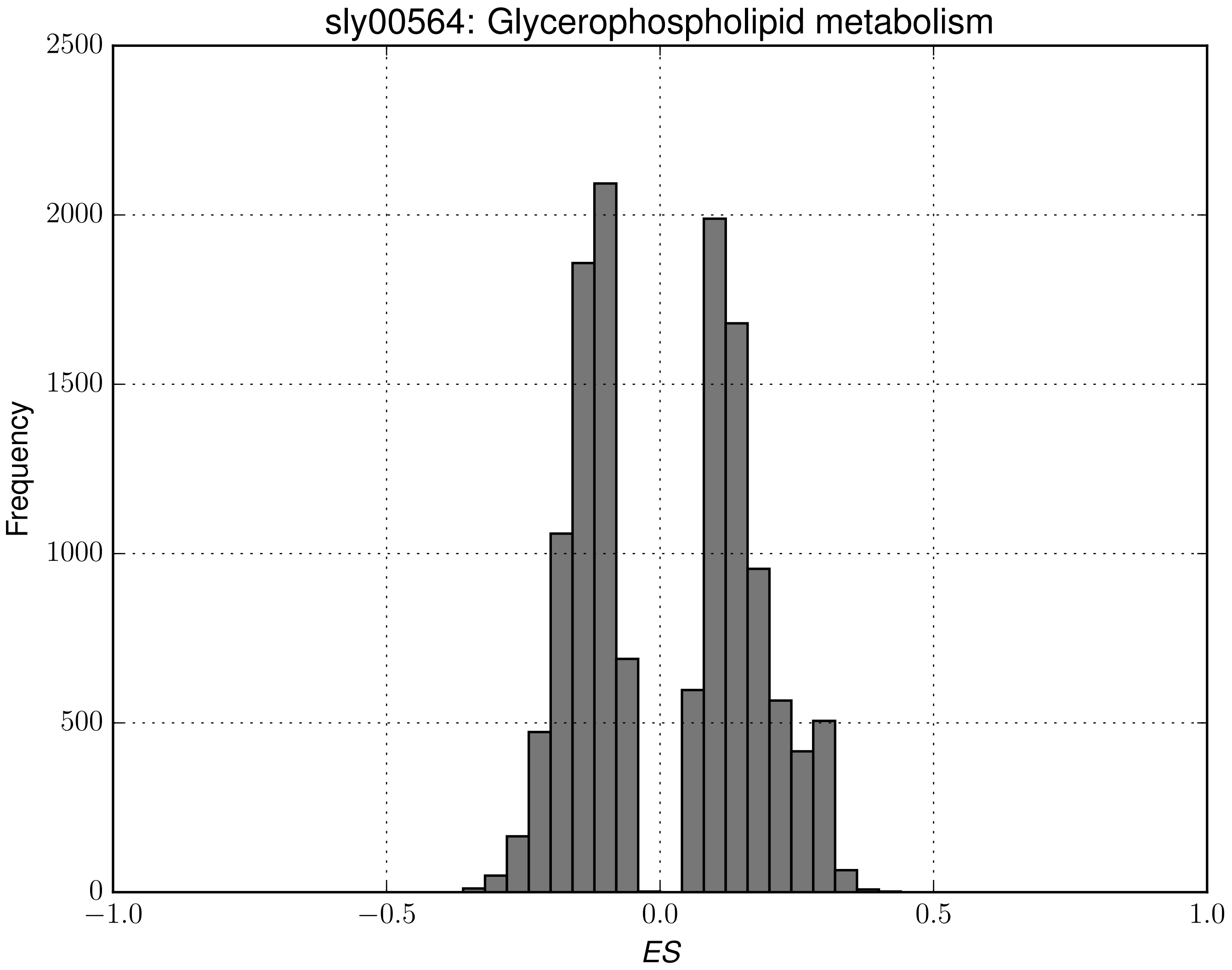

sly00564: Glycerophospholipid metabolism
Frequency
ES
2500
2000
1500
1000
500
0
−1.0
−0.5
0.0
0.5
1.0

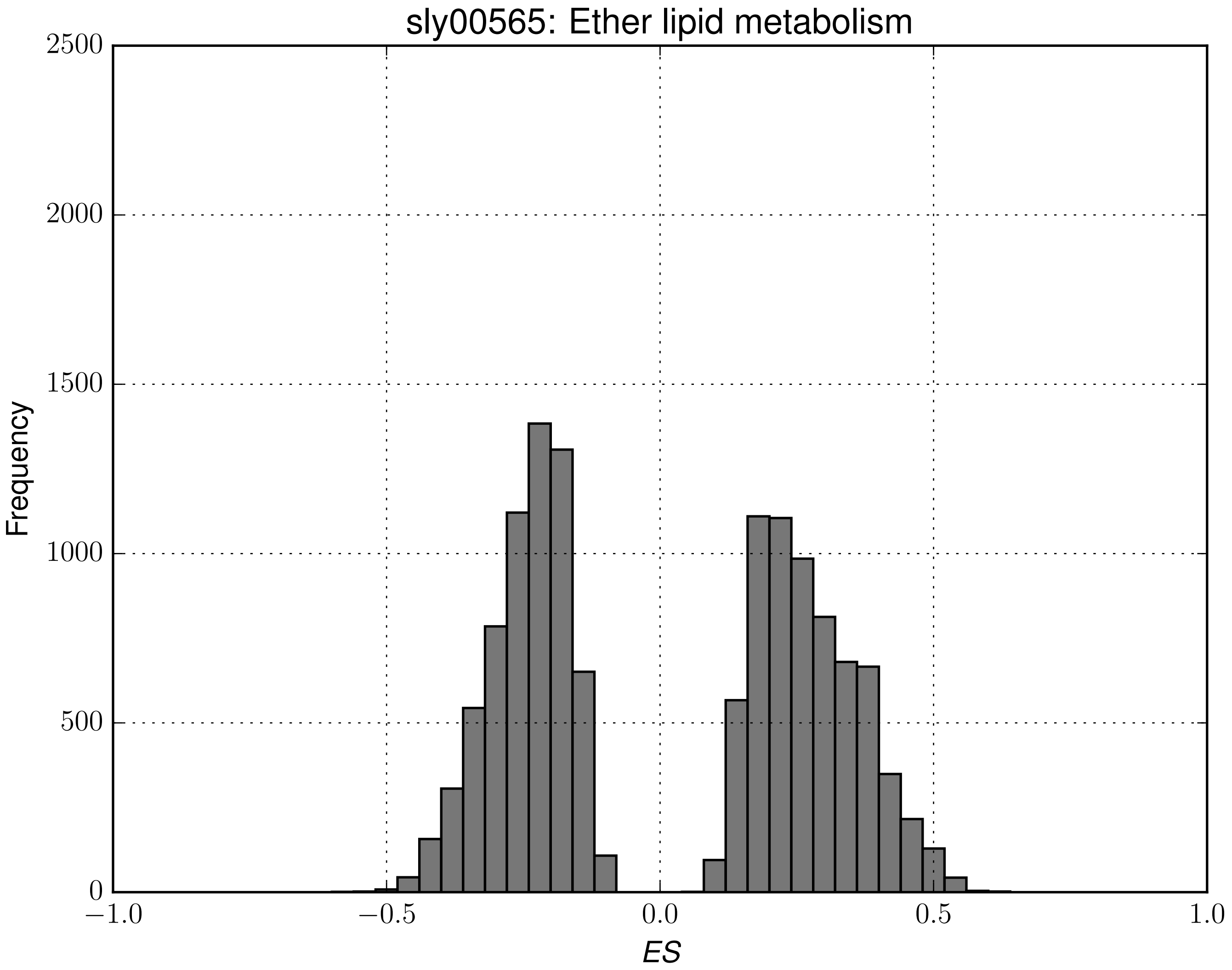
sly00565: Ether lipid metabolism
Frequency
ES
2500
2000
1500
1000
500
0
−1.0
−0.5
0.0
0.5
1.0

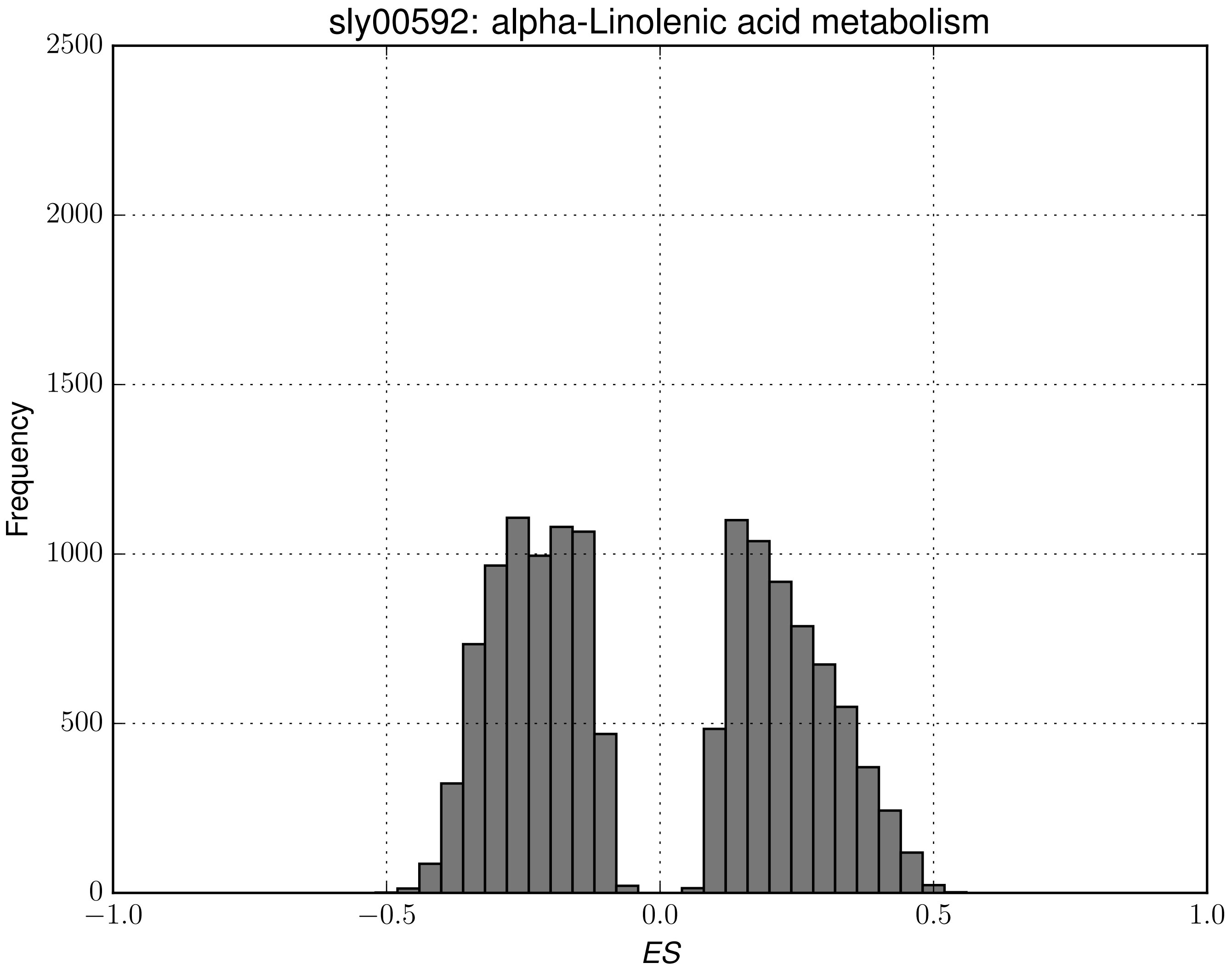

sly00592: alpha-Linolenic acid metabolism
Frequency
ES
2500
2000
1500
1000
500
0
−1.0
−0.5
0.0
0.5
1.0

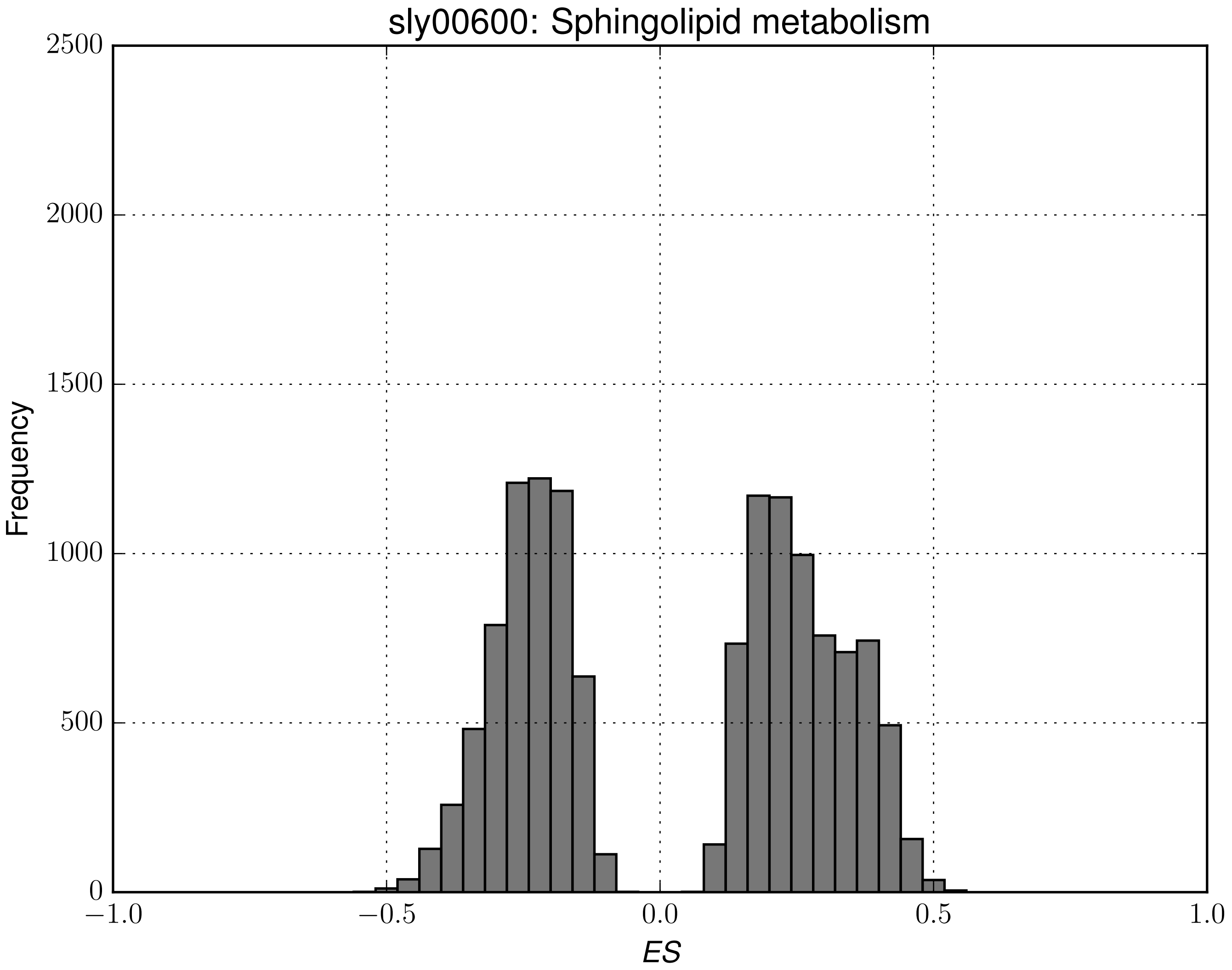

sly00600: Sphingolipid metabolism
Frequency
ES
2500
2000
1500
1000
500
0
−1.0
−0.5
0.0
0.5
1.0

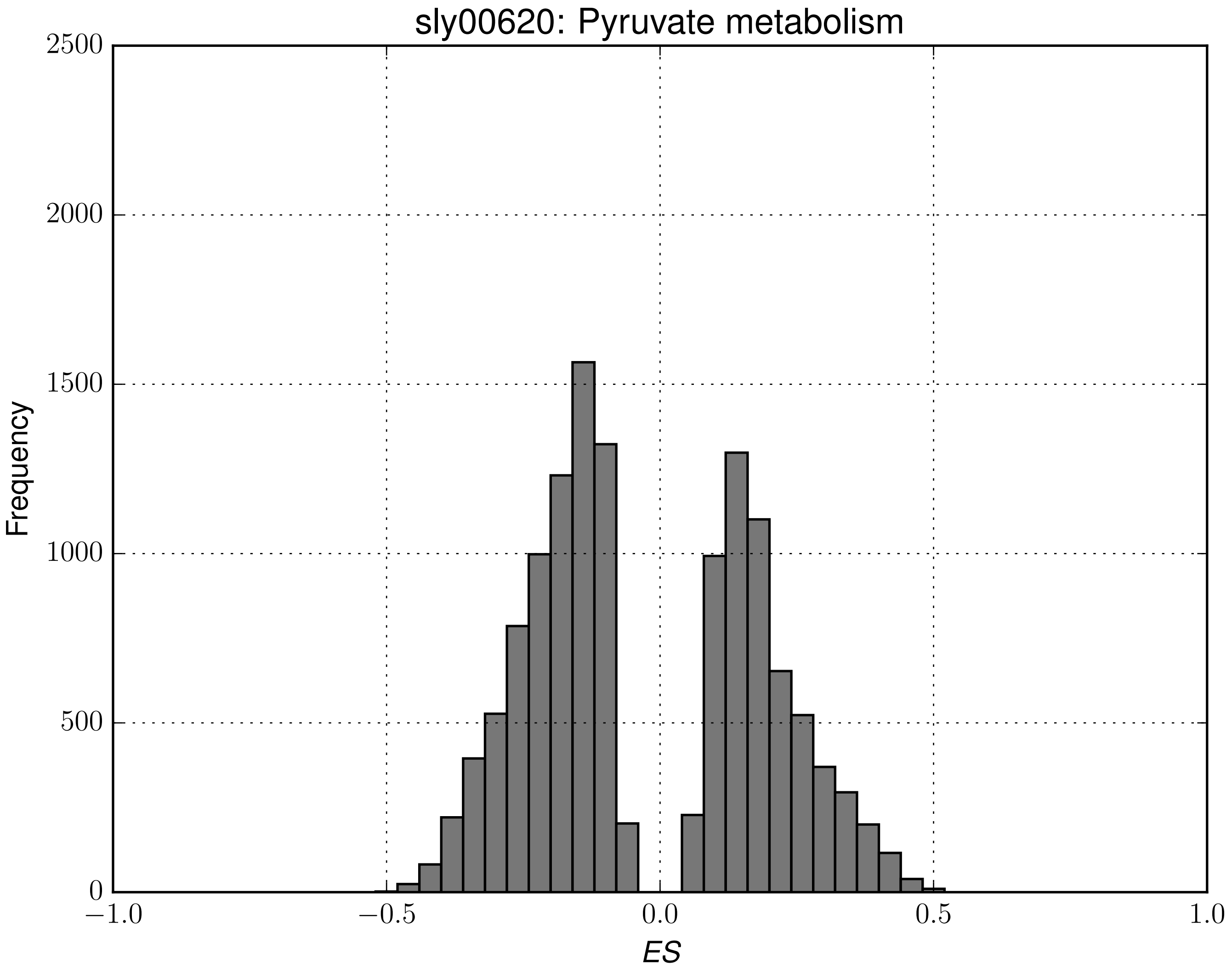

sly00620: Pyruvate metabolism
2500
2000
1500
1000
500
0
Frequency
−1.0
−0.5
0.0
0.5
1.0
ES

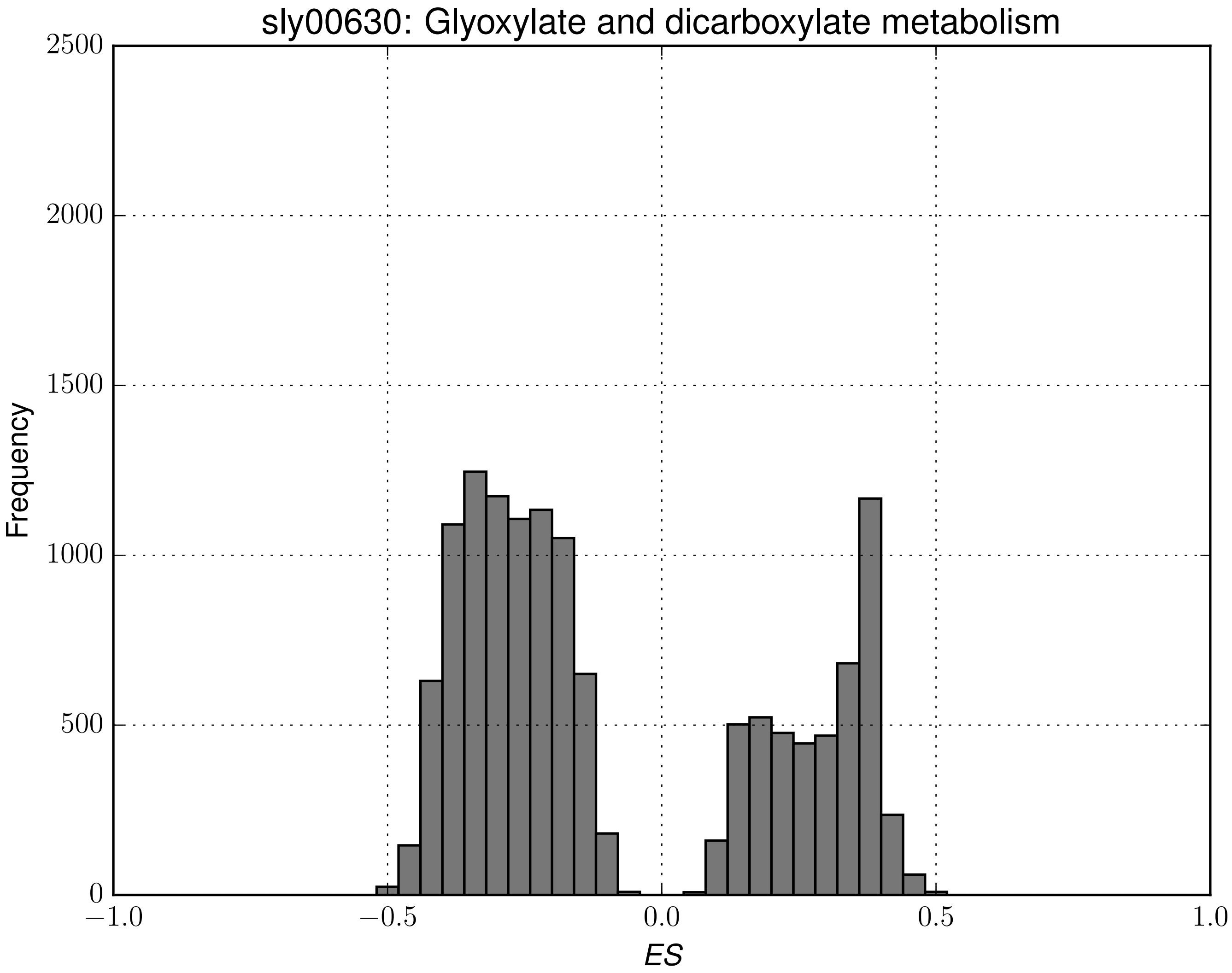

sly00630: Glyoxylate and dicarboxylate metabolism
Frequency
ES
0
500
1000
1500
2000
2500
−1.0
−0.5
0.0
0.5
1.0

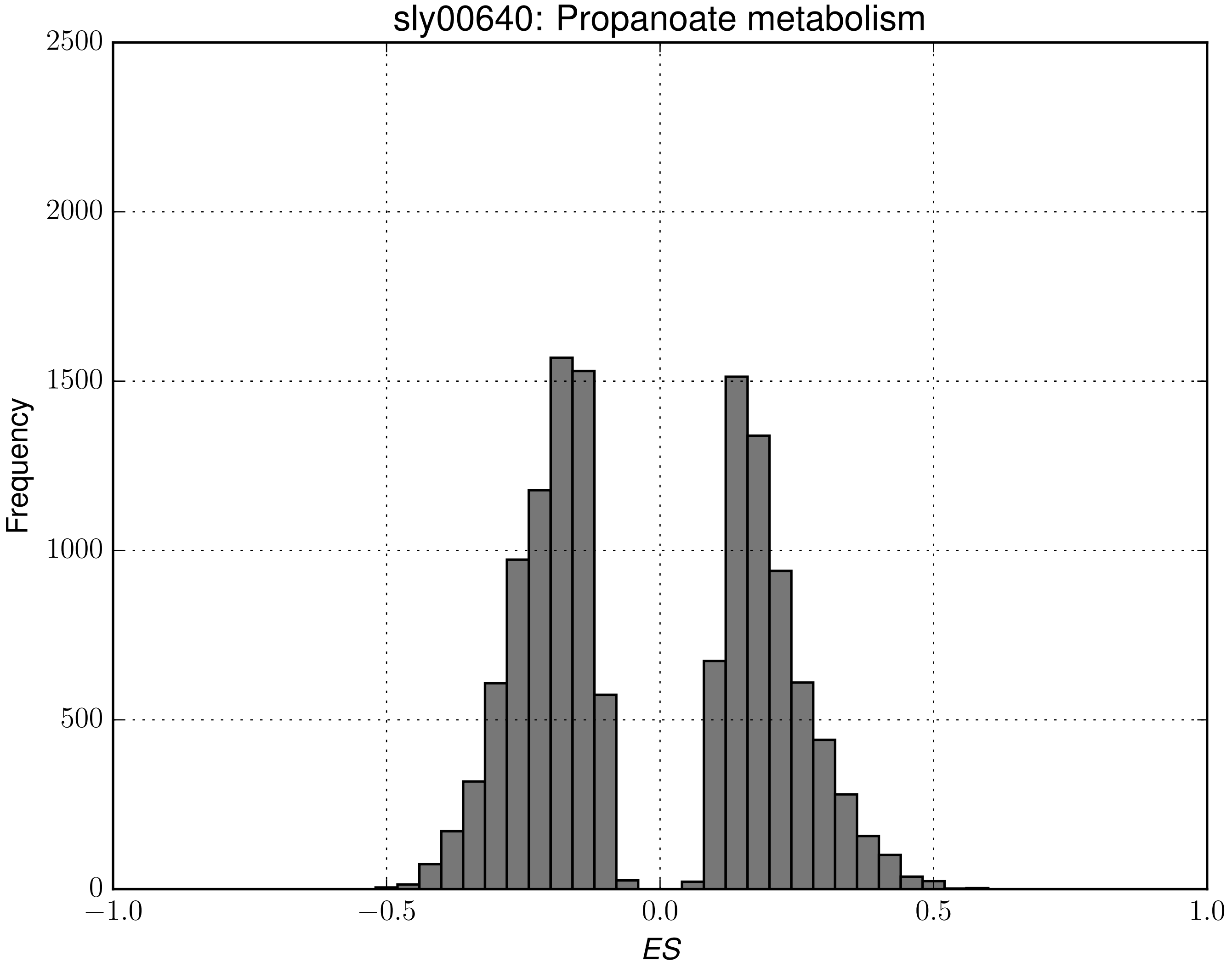
sly00640: Propanoate metabolism
Frequency
ES
2500
2000
1500
1000
500
0
−1.0
−0.5
0.0
0.5
1.0

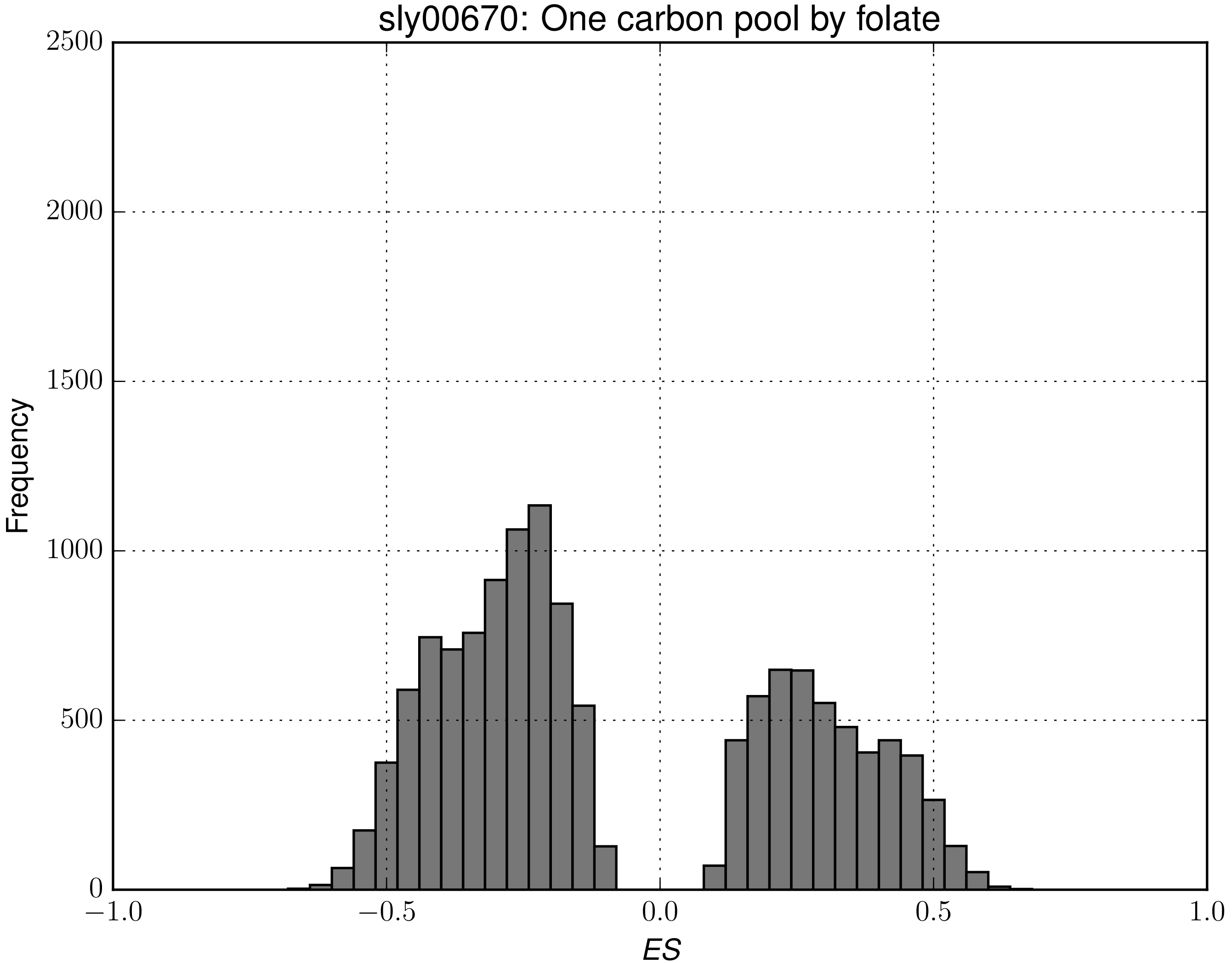

sly00670: One carbon pool by folate
Frequency
ES
0
500
1000
1500
2000
2500
−1.0
−0.5
0.0
0.5
1.0

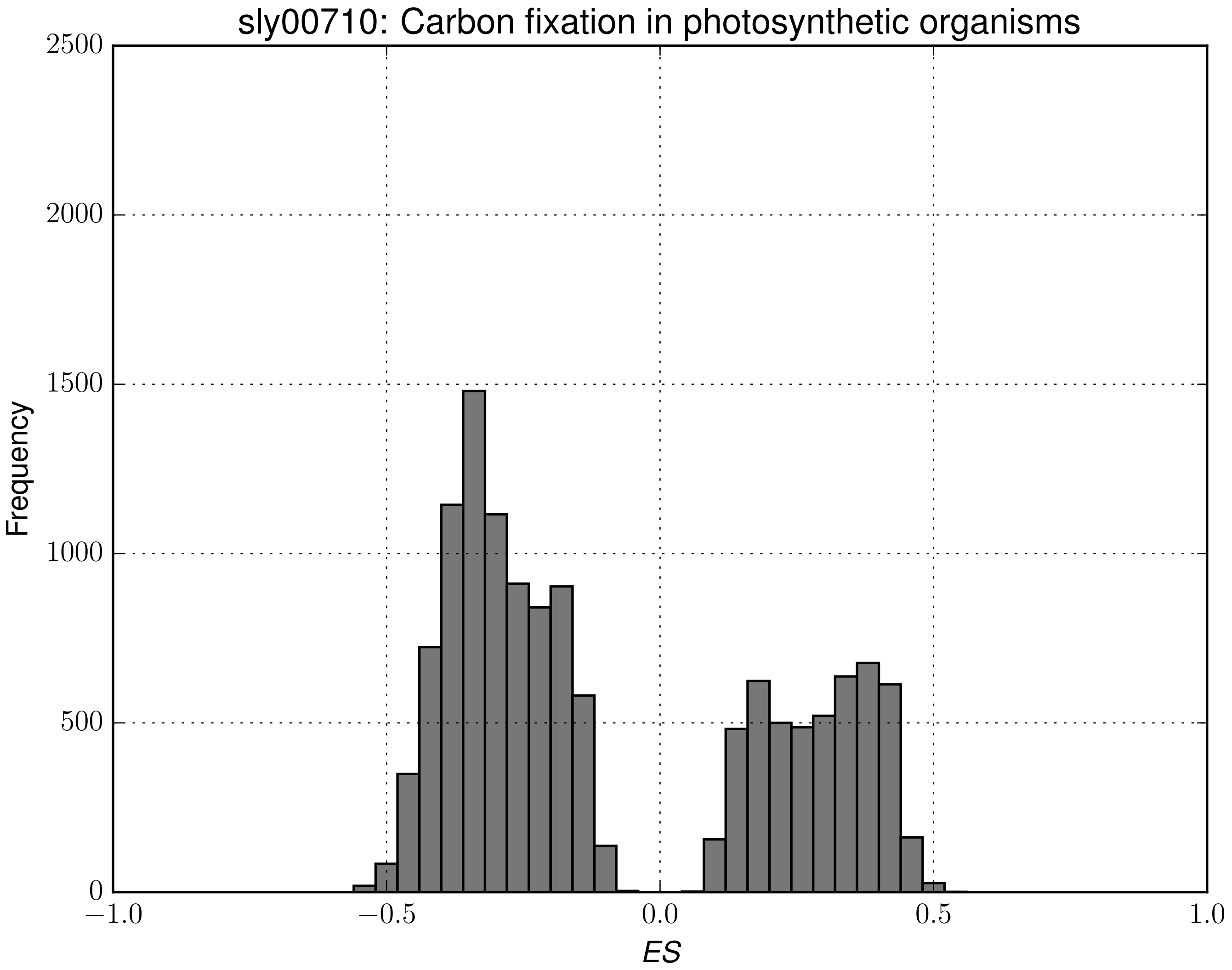
sly00710: Carbon fixation in photosynthetic organisms
Frequency
ES
2500
2000
1500
1000
500
0
−1.0
−0.5
0.0
0.5
1.0

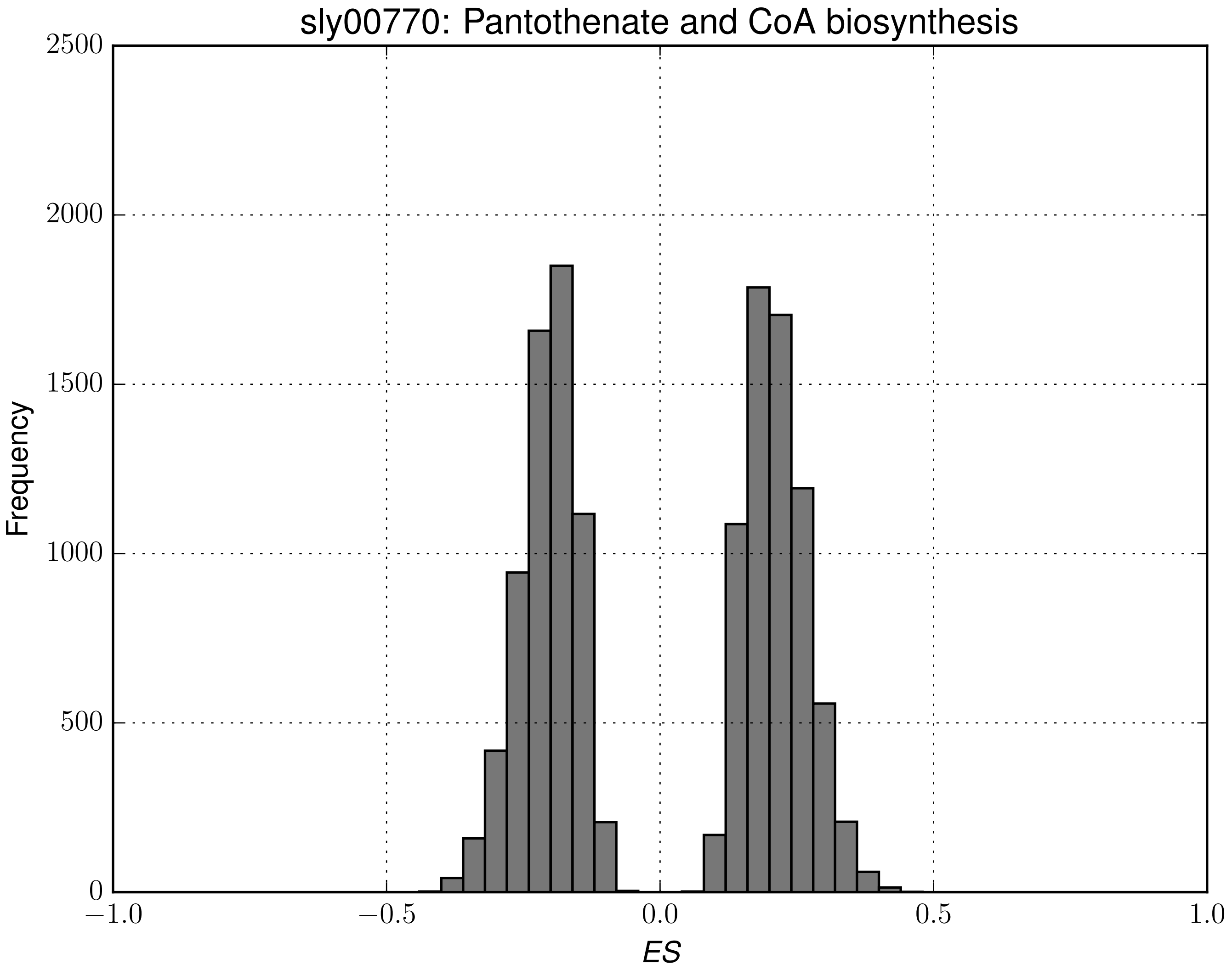
sly00770: Pantothenate and CoA biosynthesis
Frequency
ES
2500
2000
1500
1000
500
0
−1.0
−0.5
0.0
0.5
1.0

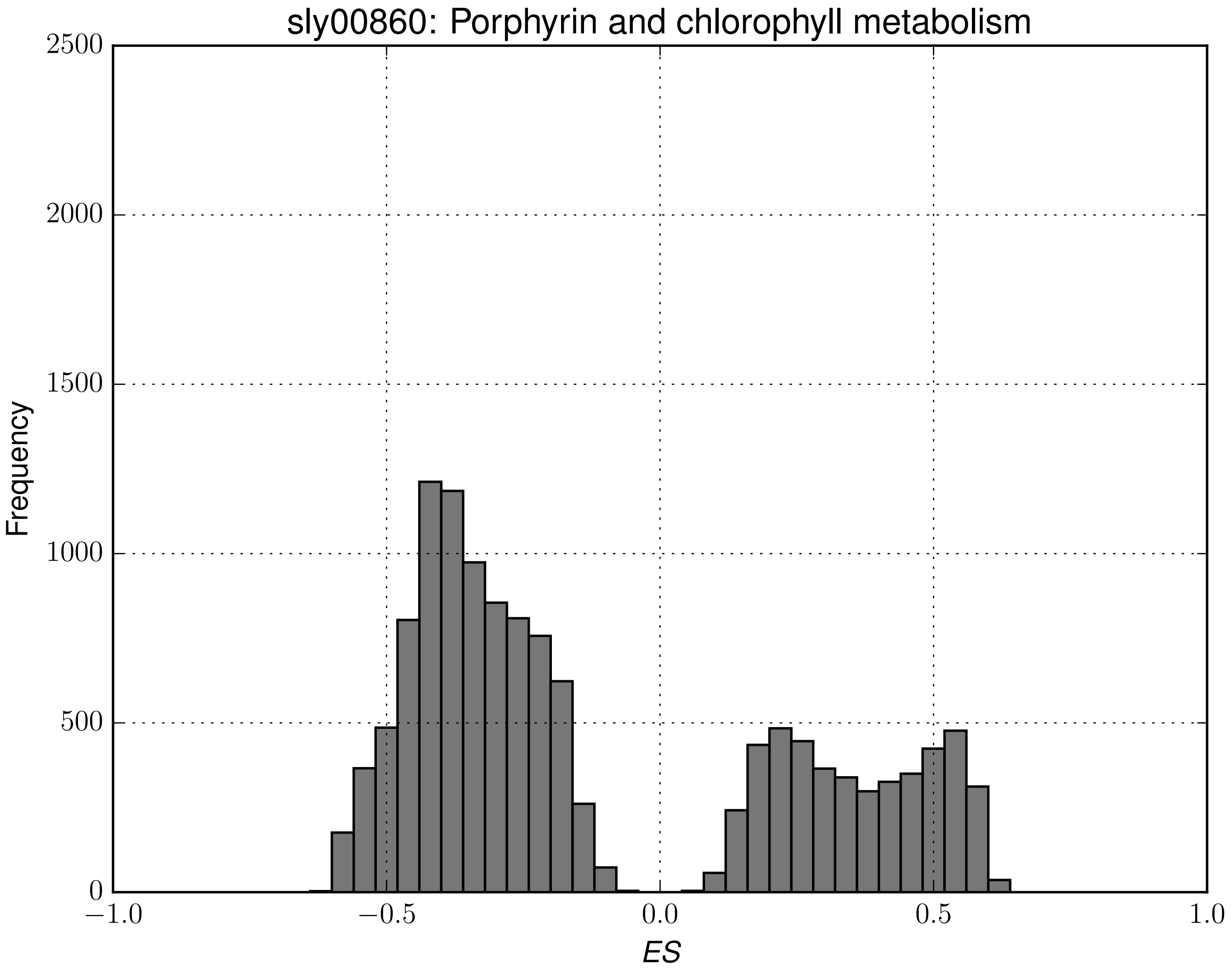

sly00860: Porphyrin and chlorophyll metabolism
2500
2000
1500
1000
500
0
Frequency
−1.0
−0.5
0.0
0.5
1.0
ES

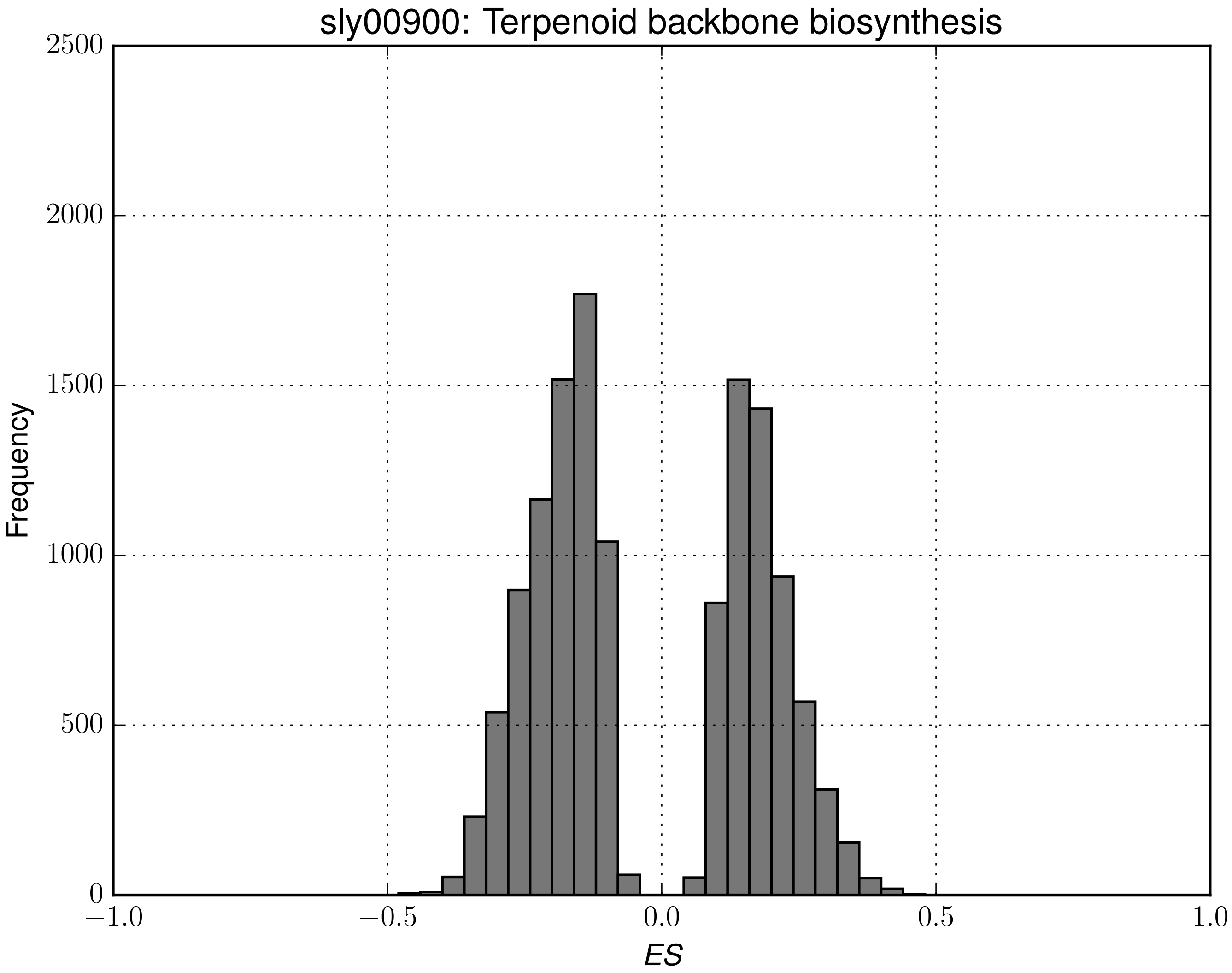

sly00900: Terpenoid backbone biosynthesis
Frequency
ES
0
500
1000
1500
2000
2500
−1.0
−0.5
0.0
0.5
1.0

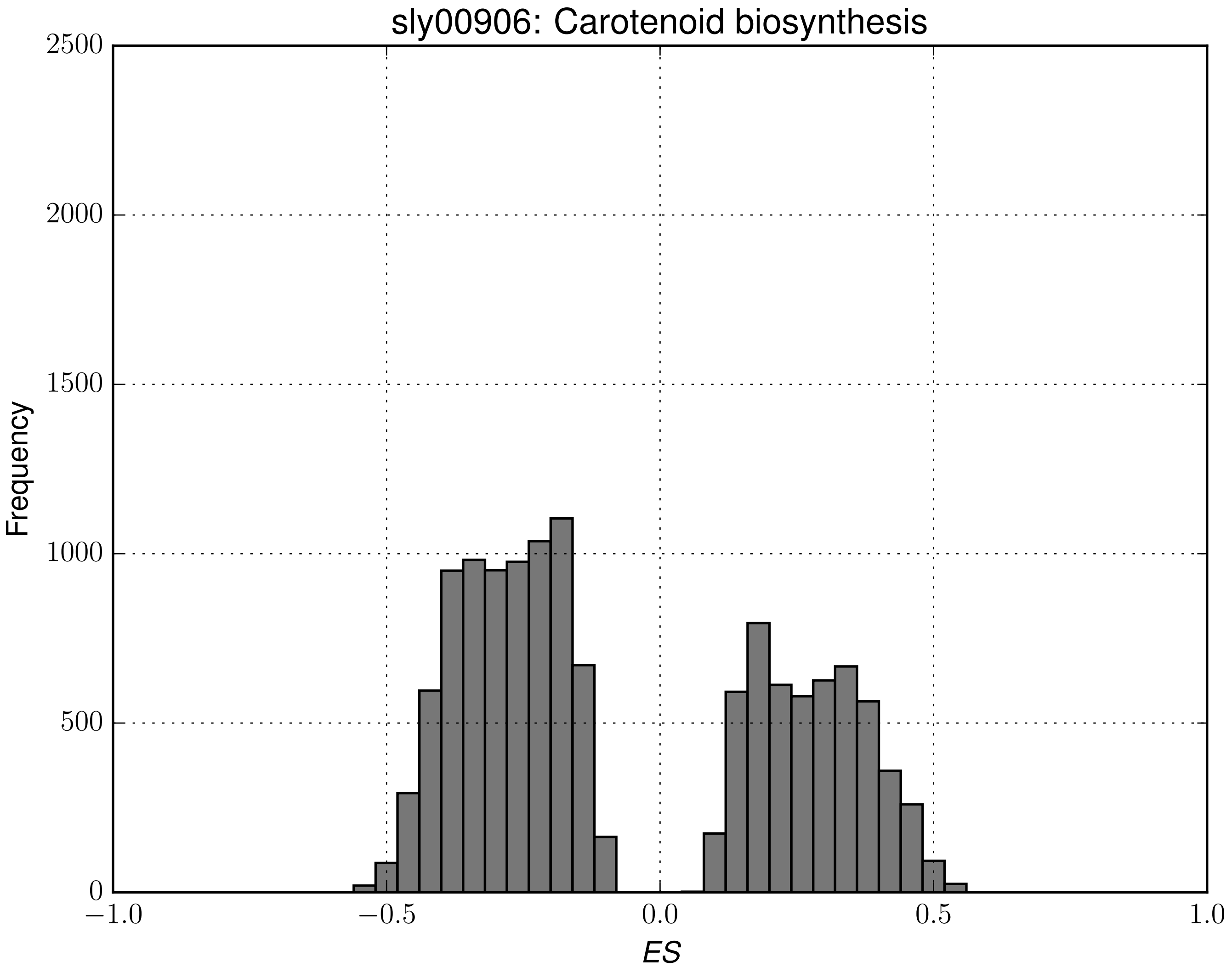
sly00906: Carotenoid biosynthesis
2500
2000
1500
1000
500
0
Frequency
−1.0
−0.5
0.0
0.5
1.0
ES

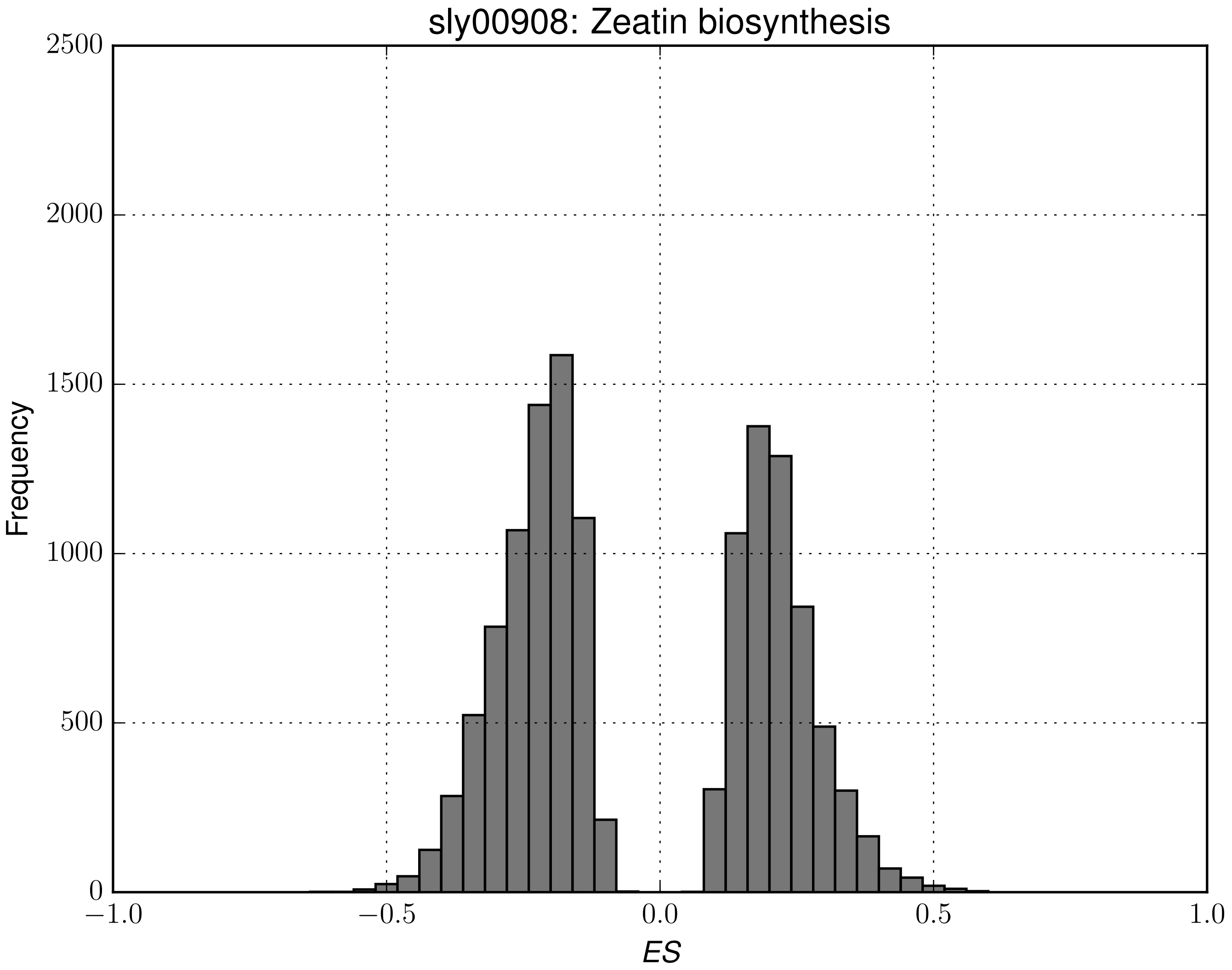

sly00908: Zeatin biosynthesis
Frequency
ES
2500
2000
1500
1000
500
0
−1.0
−0.5
0.0
0.5
1.0

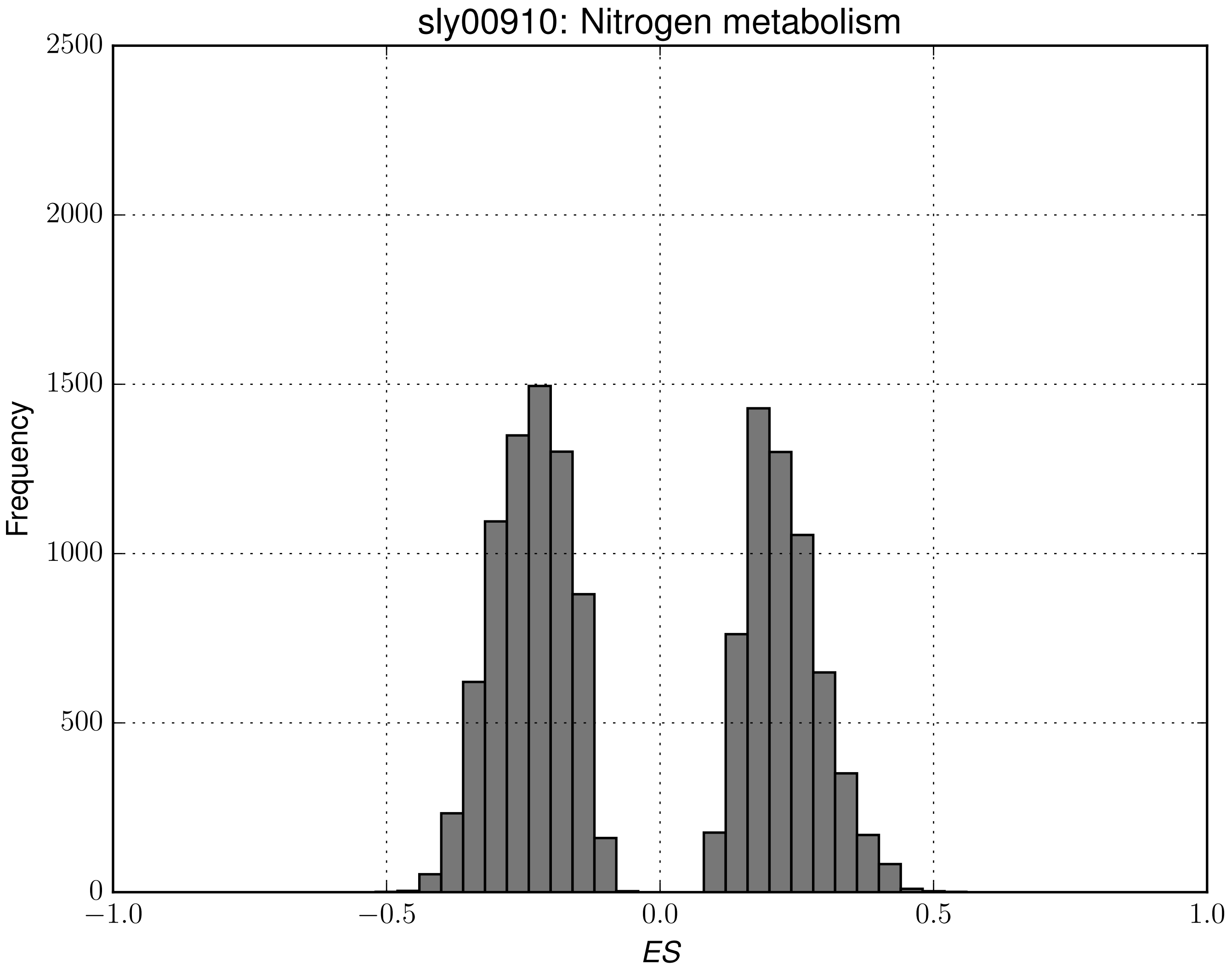

sly00910: Nitrogen metabolism
Frequency
ES
2500
2000
1500
1000
500
0
−1.0
−0.5
0.0
0.5
1.0

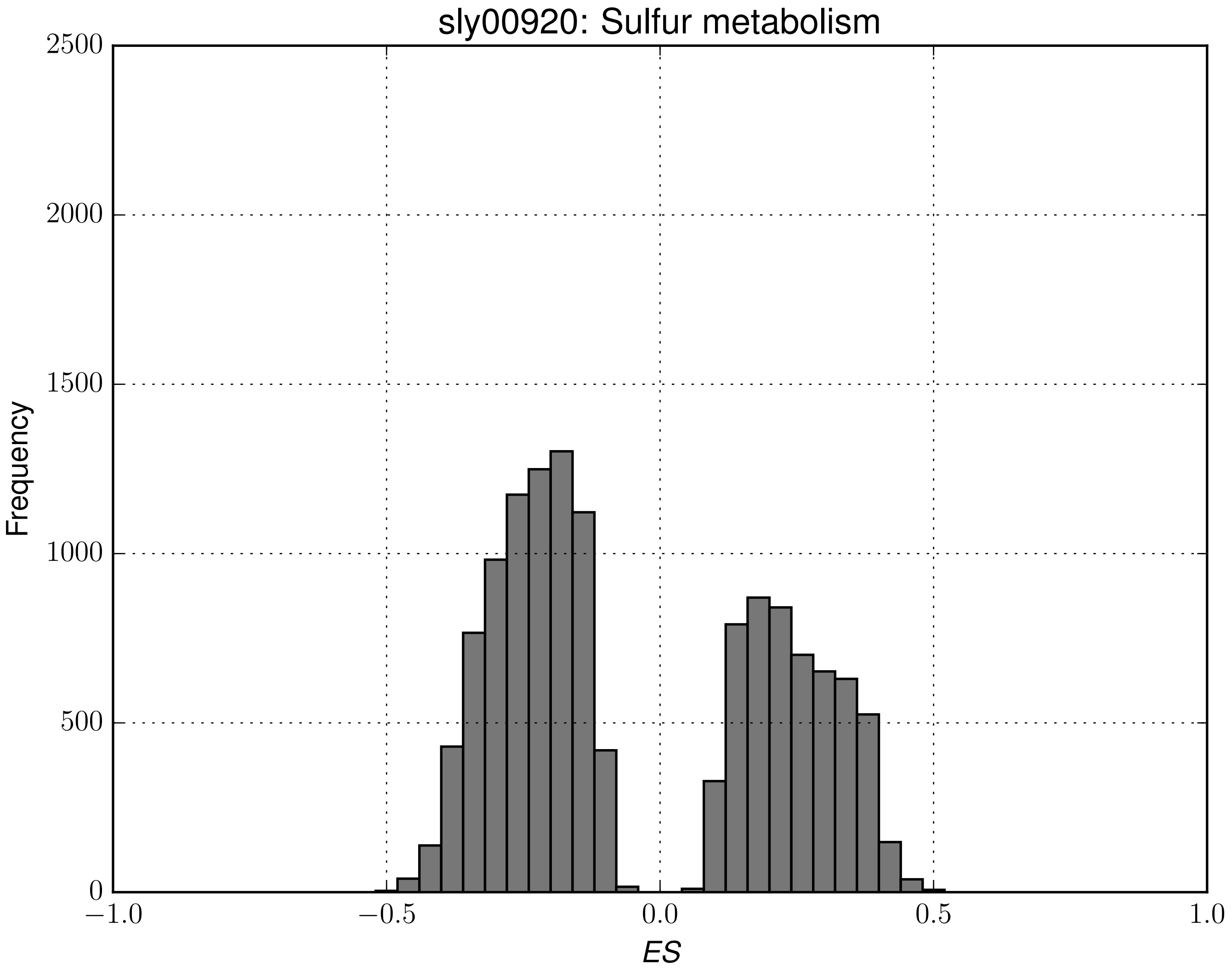
sly00920: Sulfur metabolism
Frequency
ES
2500
2000
1500
1000
500
0
−1.0
−0.5
0.0
0.5
1.0

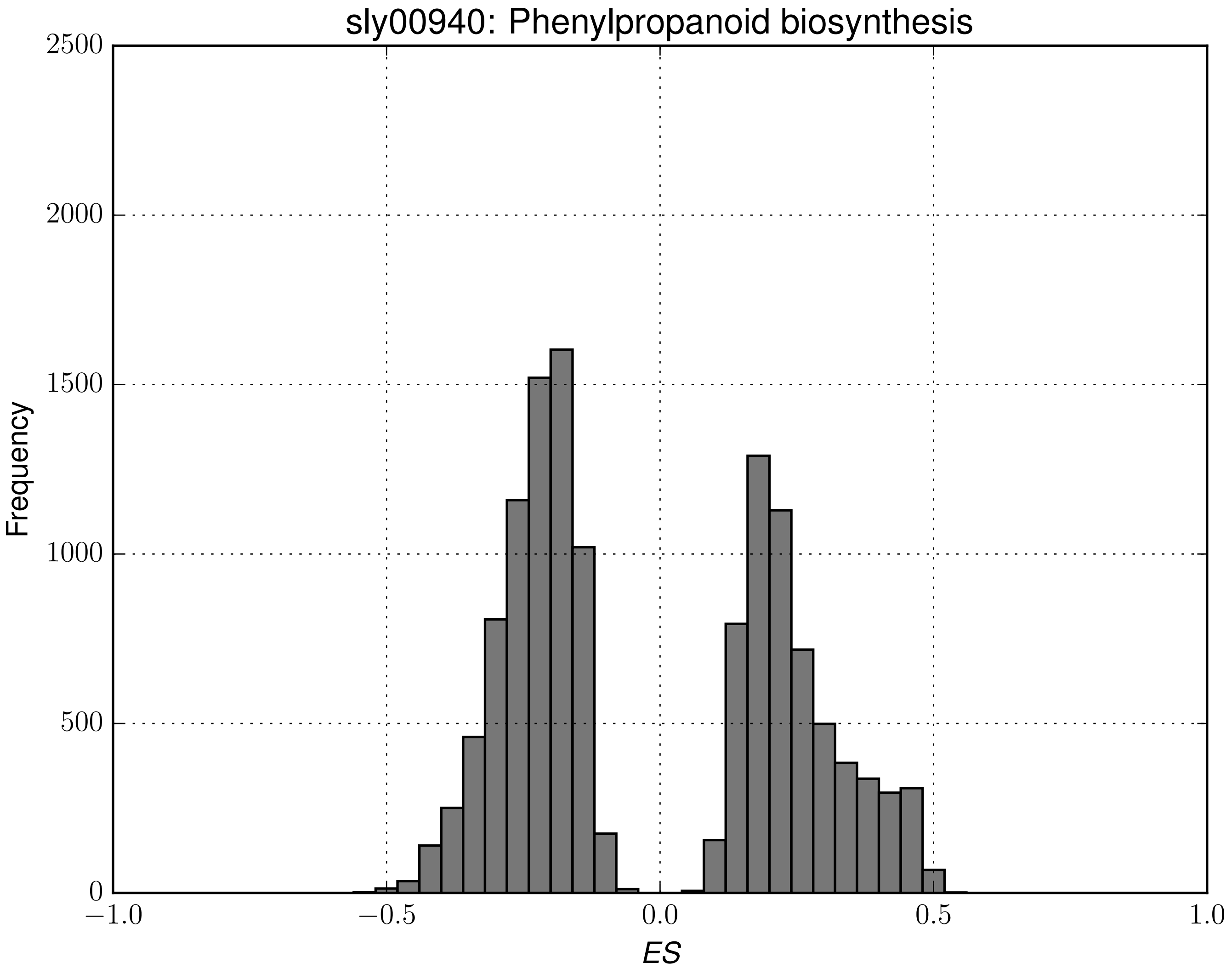
sly00940: Phenylpropanoid biosynthesis
Frequency
ES
0
500
1000
1500
2000
2500
−1.0
−0.5
0.0
0.5
1.0

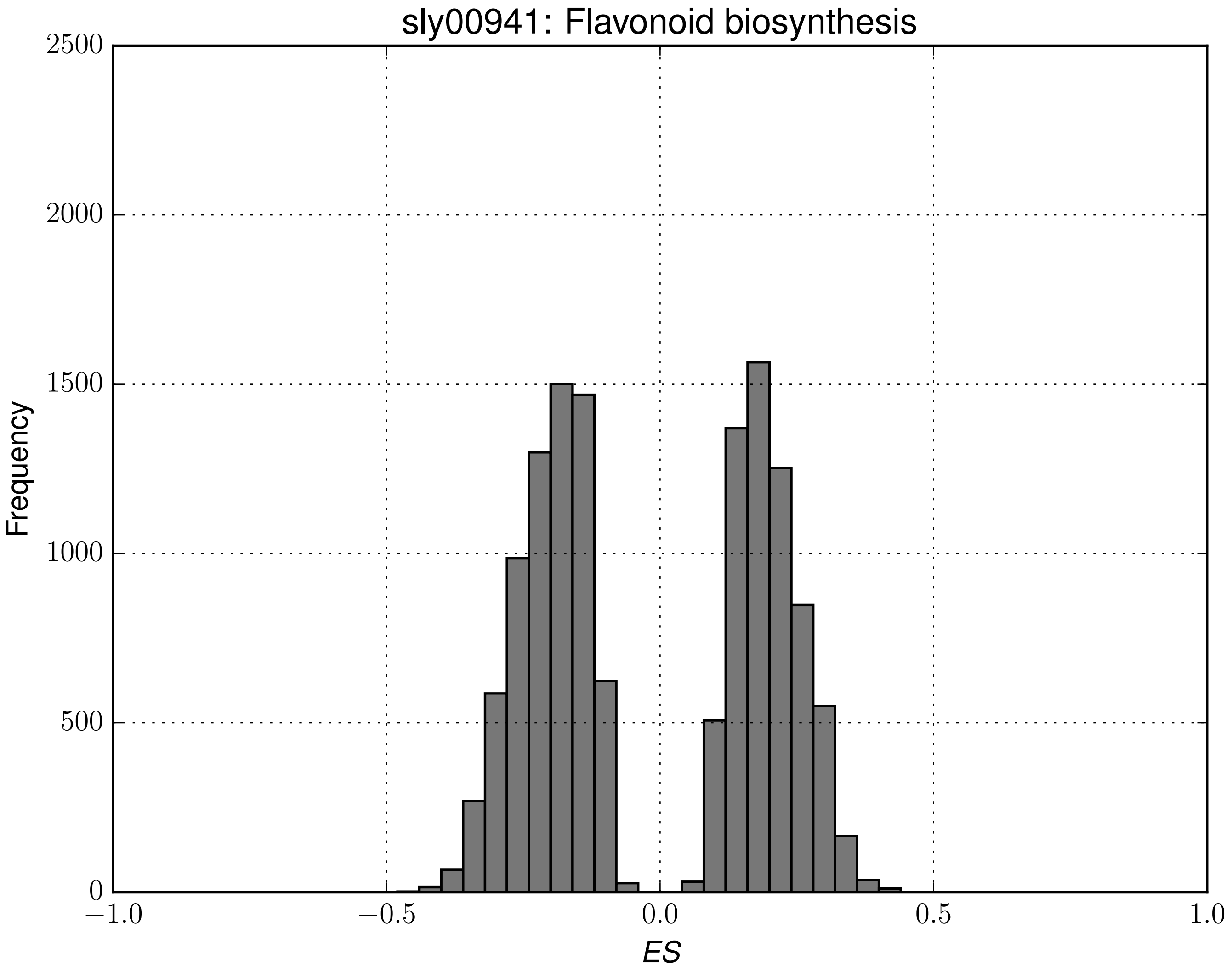
sly00941: Flavonoid biosynthesis
Frequency
ES
2500
2000
1500
1000
500
0
−1.0
−0.5
0.0
0.5
1.0

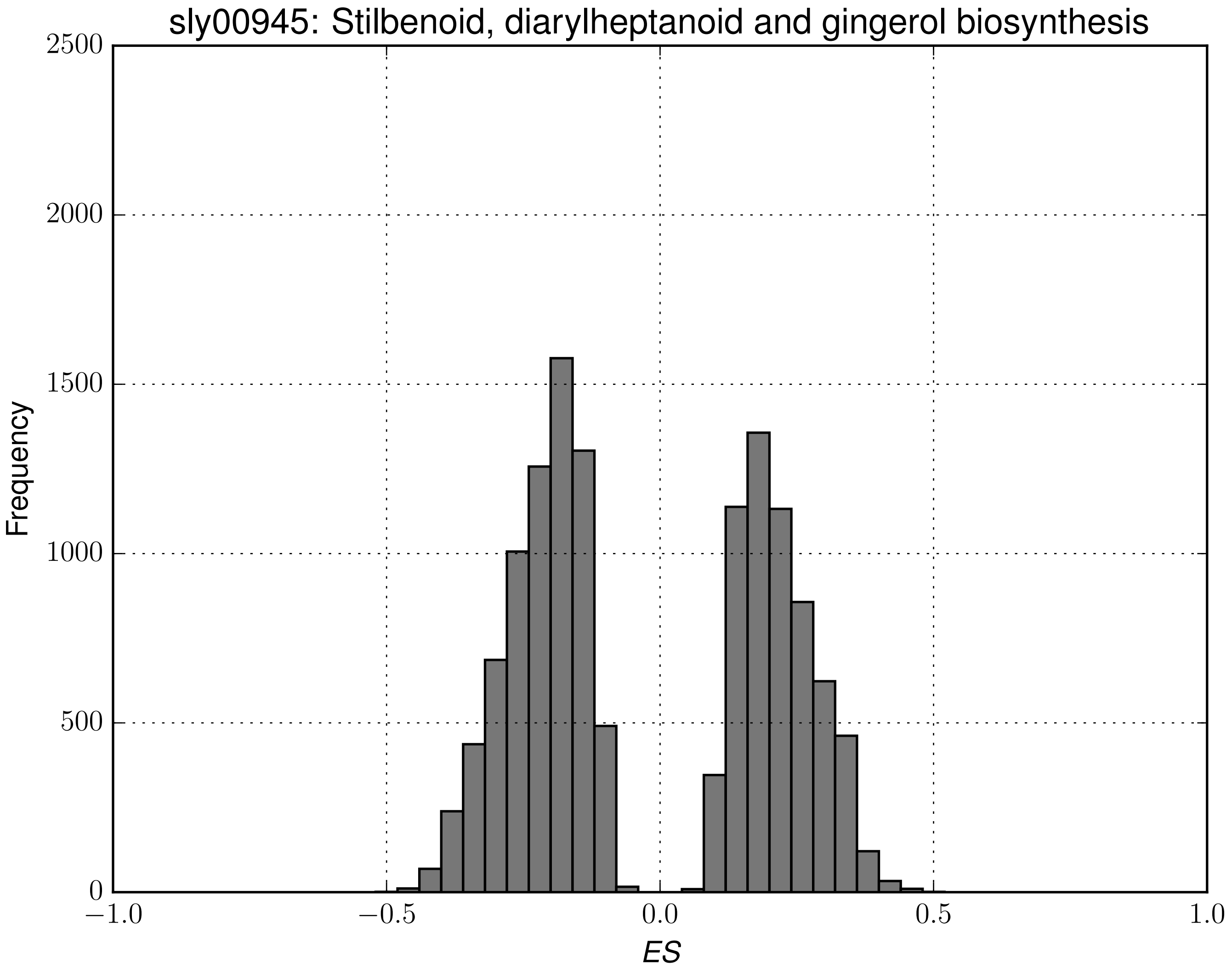
sly00945: Stilbenoid, diarylheptanoid and gingerol biosynthesis
Frequency
ES
2500
2000
1500
1000
500
0
−1.0
−0.5
0.0
0.5
1.0

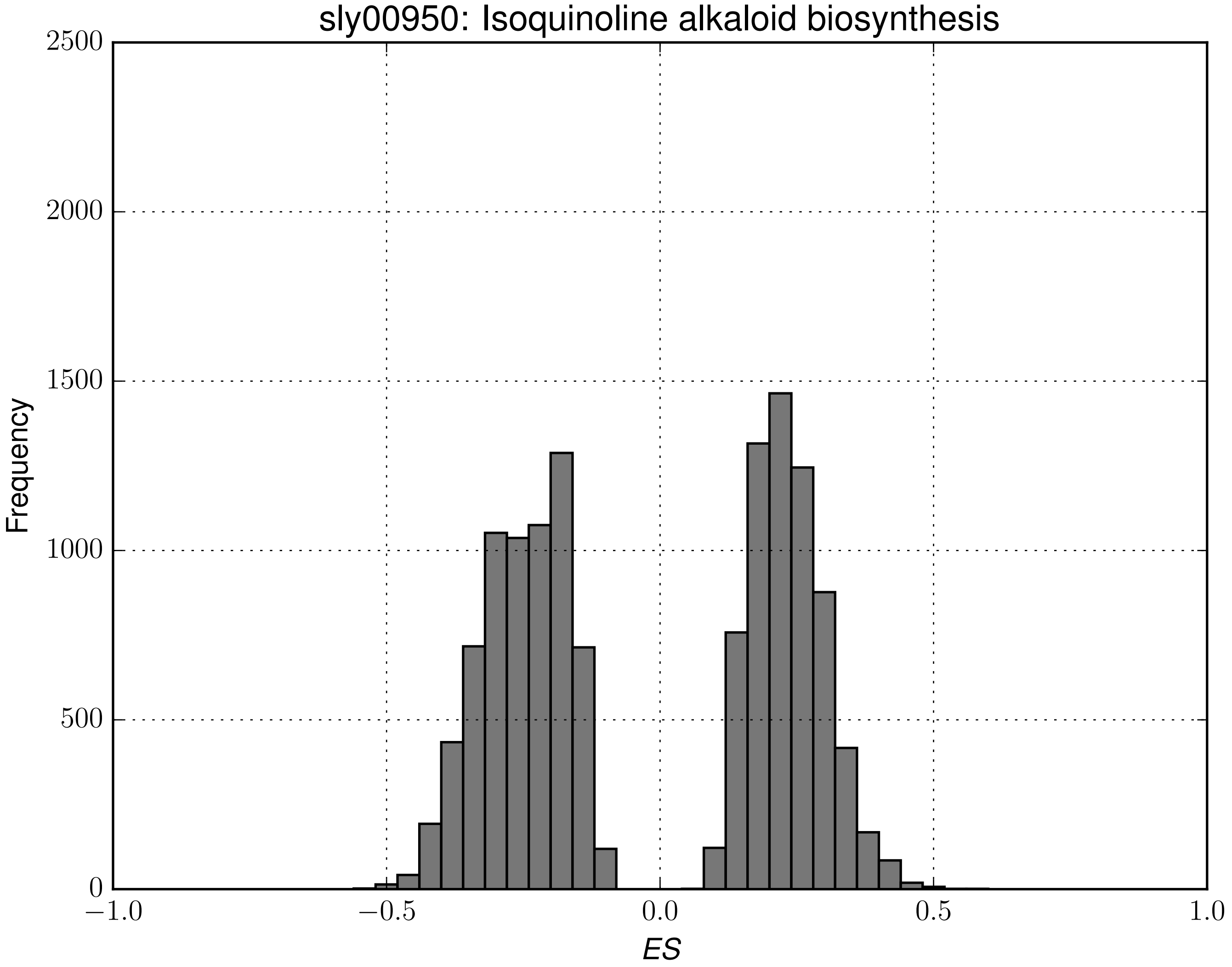

sly00950: Isoquinoline alkaloid biosynthesis
Frequency
ES
2500
2000
1500
1000
500
0
−1.0
−0.5
0.0
0.5
1.0

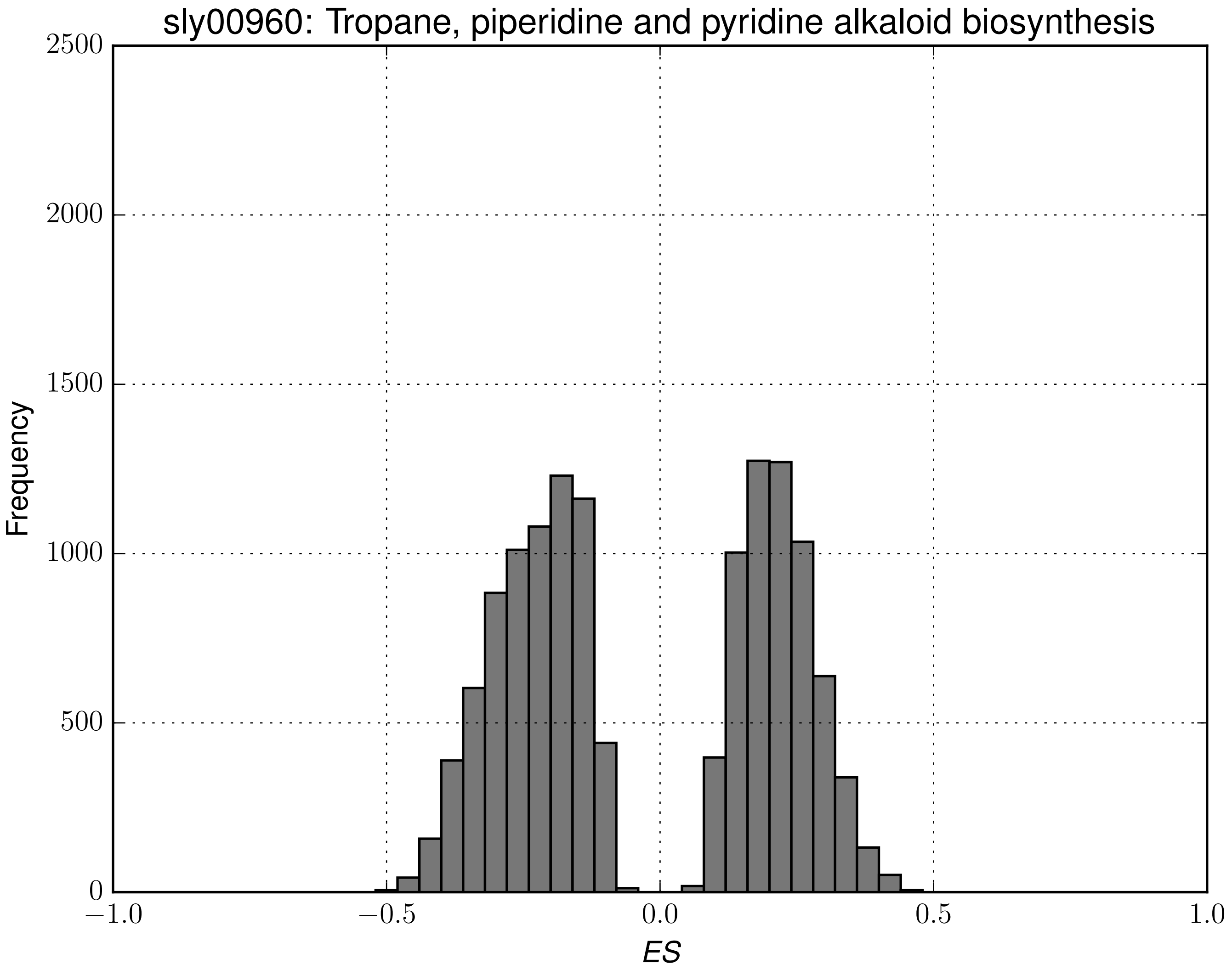
sly00960: Tropane, piperidine and pyridine alkaloid biosynthesis
Frequency
ES
2500
2000
1500
1000
500
0
−1.0
−0.5
0.0
0.5
1.0

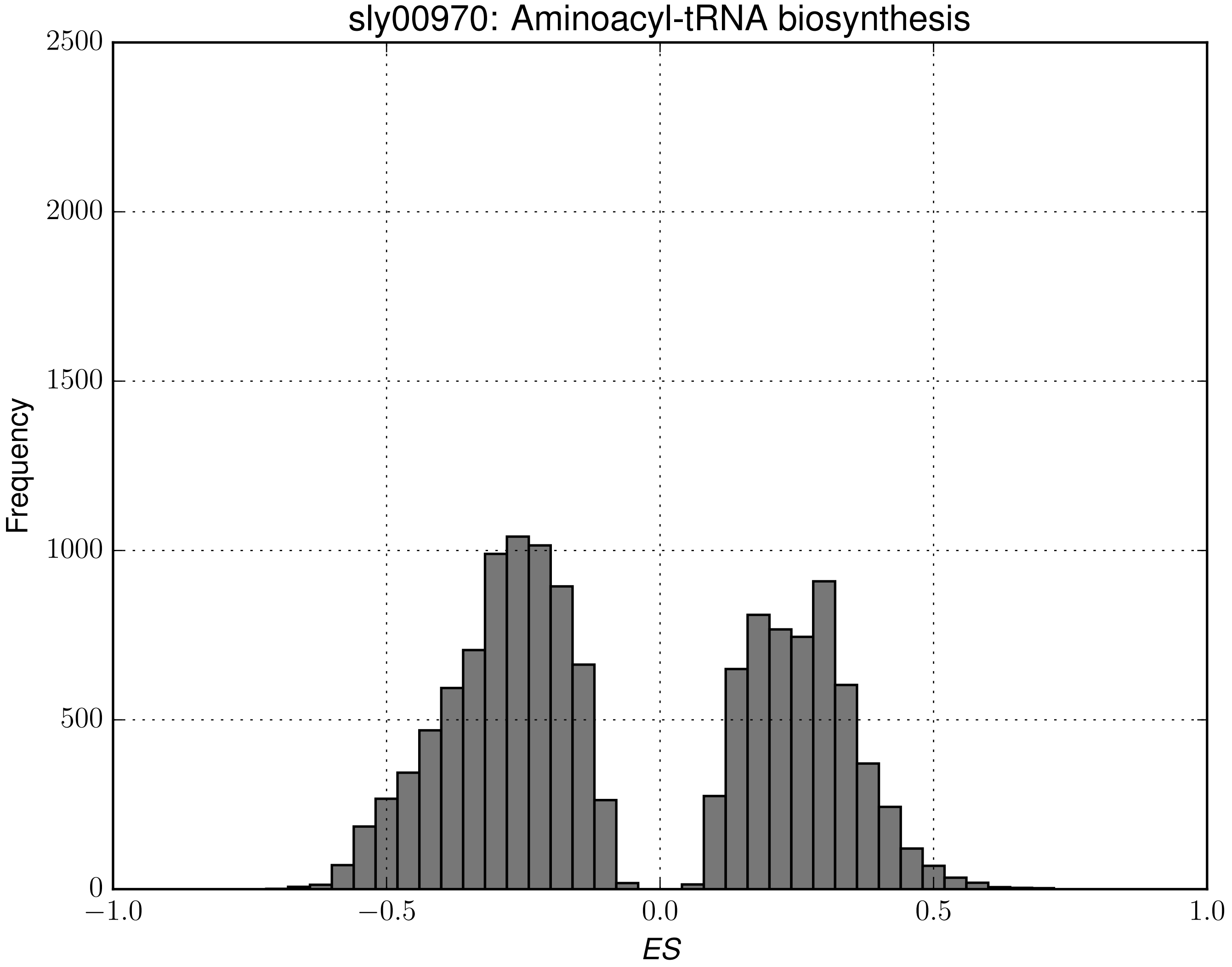

sly00970: Aminoacyl-tRNA biosynthesis
Frequency
ES
2500
2000
1500
1000
500
0
−1.0
−0.5
0.0
0.5
1.0

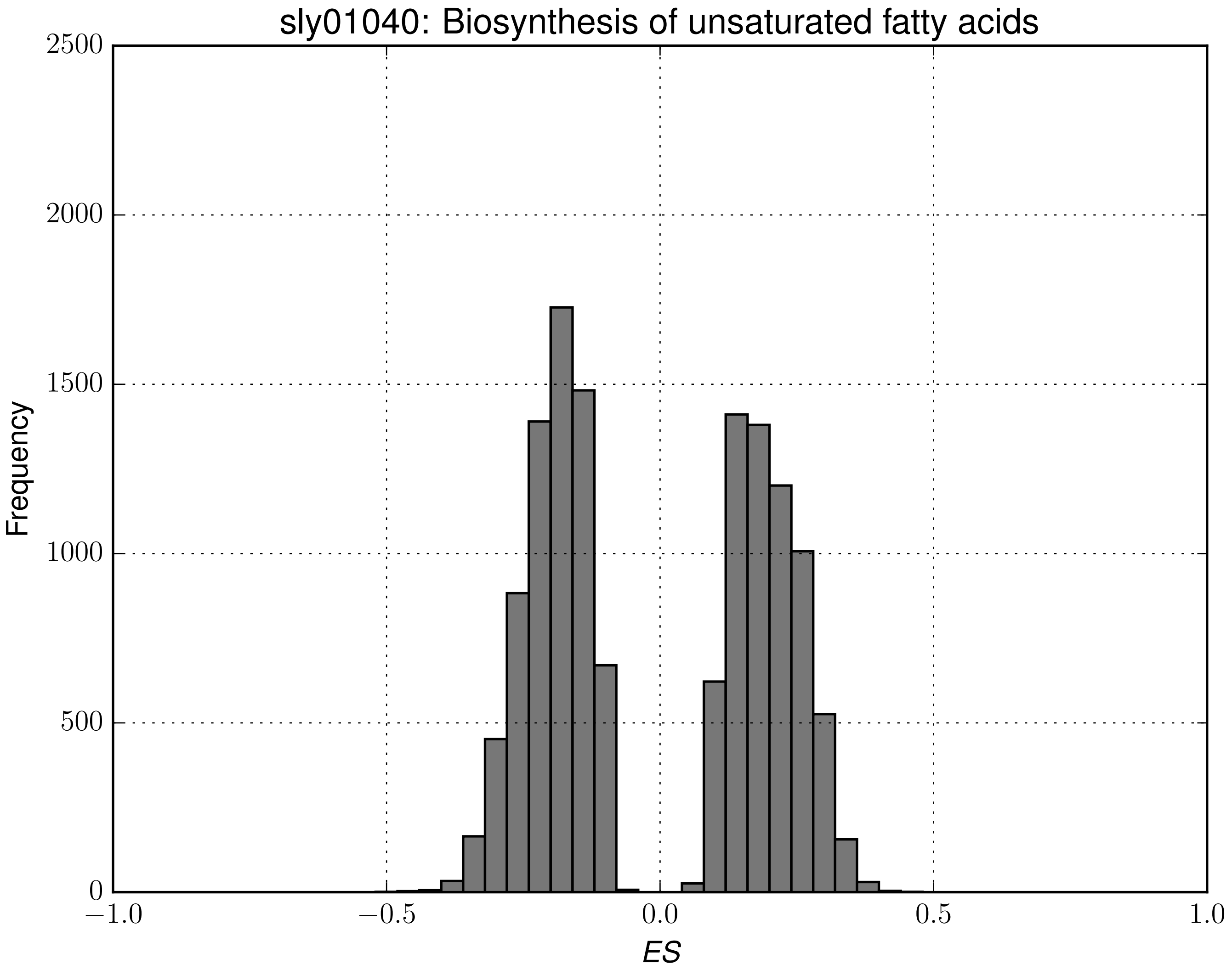

sly01040: Biosynthesis of unsaturated fatty acids
Frequency
ES
2500
2000
1500
1000
500
0
−1.0
−0.5
0.0
0.5
1.0

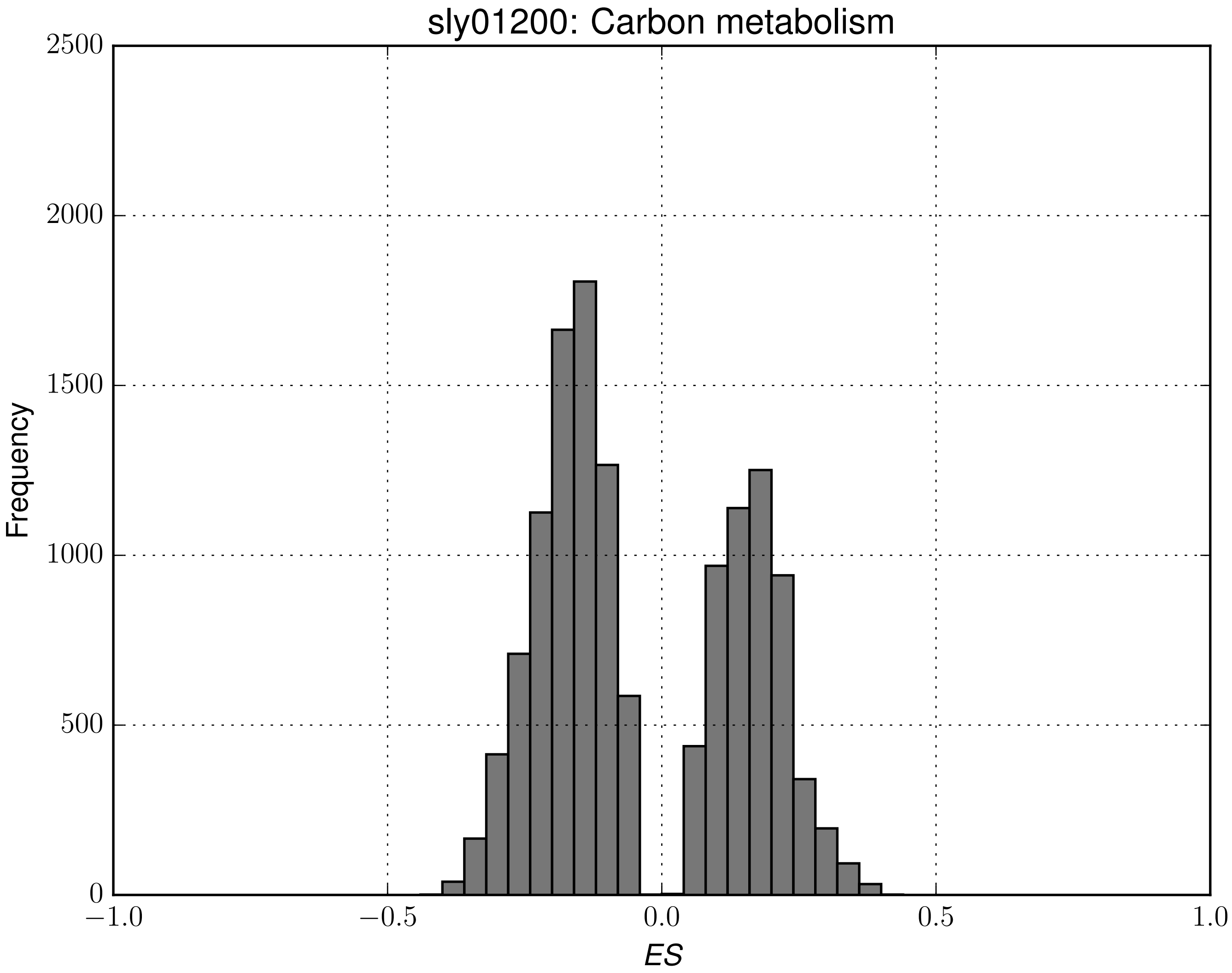

sly01200: Carbon metabolism
Frequency
ES
2500
2000
1500
1000
500
0
−1.0
−0.5
0.0
0.5
1.0

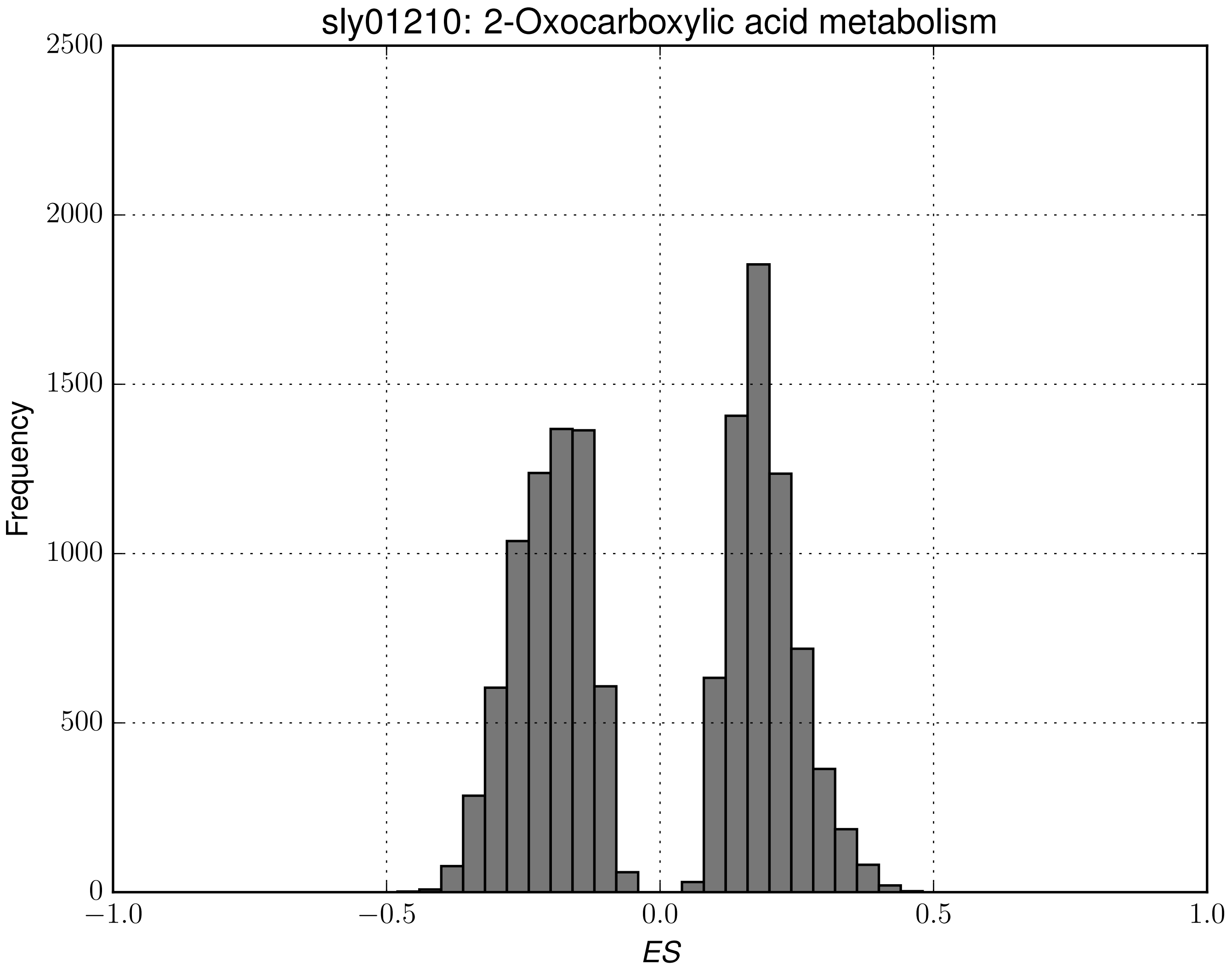

sly01210: 2-Oxocarboxylic acid metabolism
2500
2000
1500
1000
500
0
Frequency
−1.0
−0.5
0.0
0.5
1.0
ES

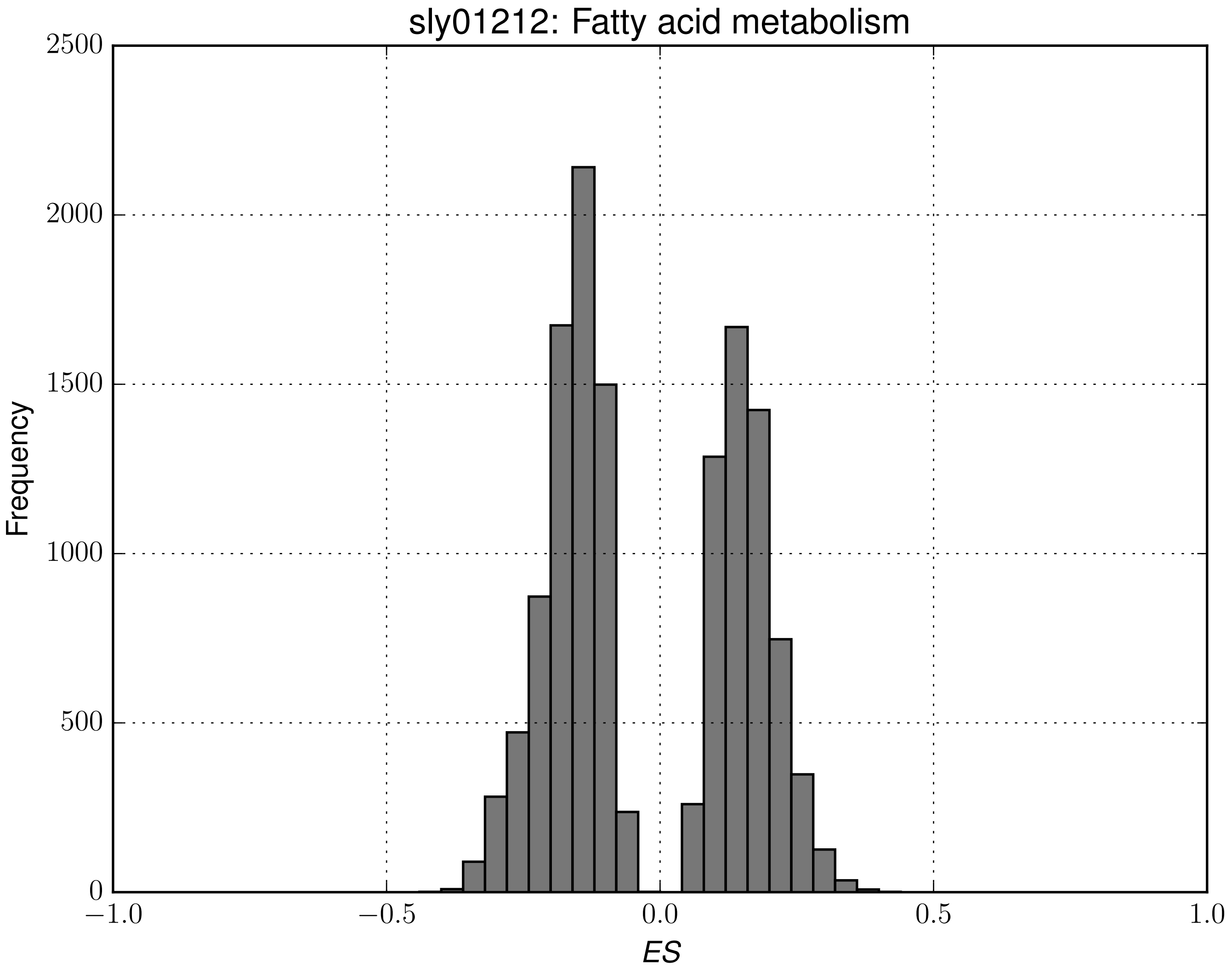
sly01212: Fatty acid metabolism
2500
2000
1500
1000
500
0
Frequency
−1.0
−0.5
0.0
0.5
1.0
ES

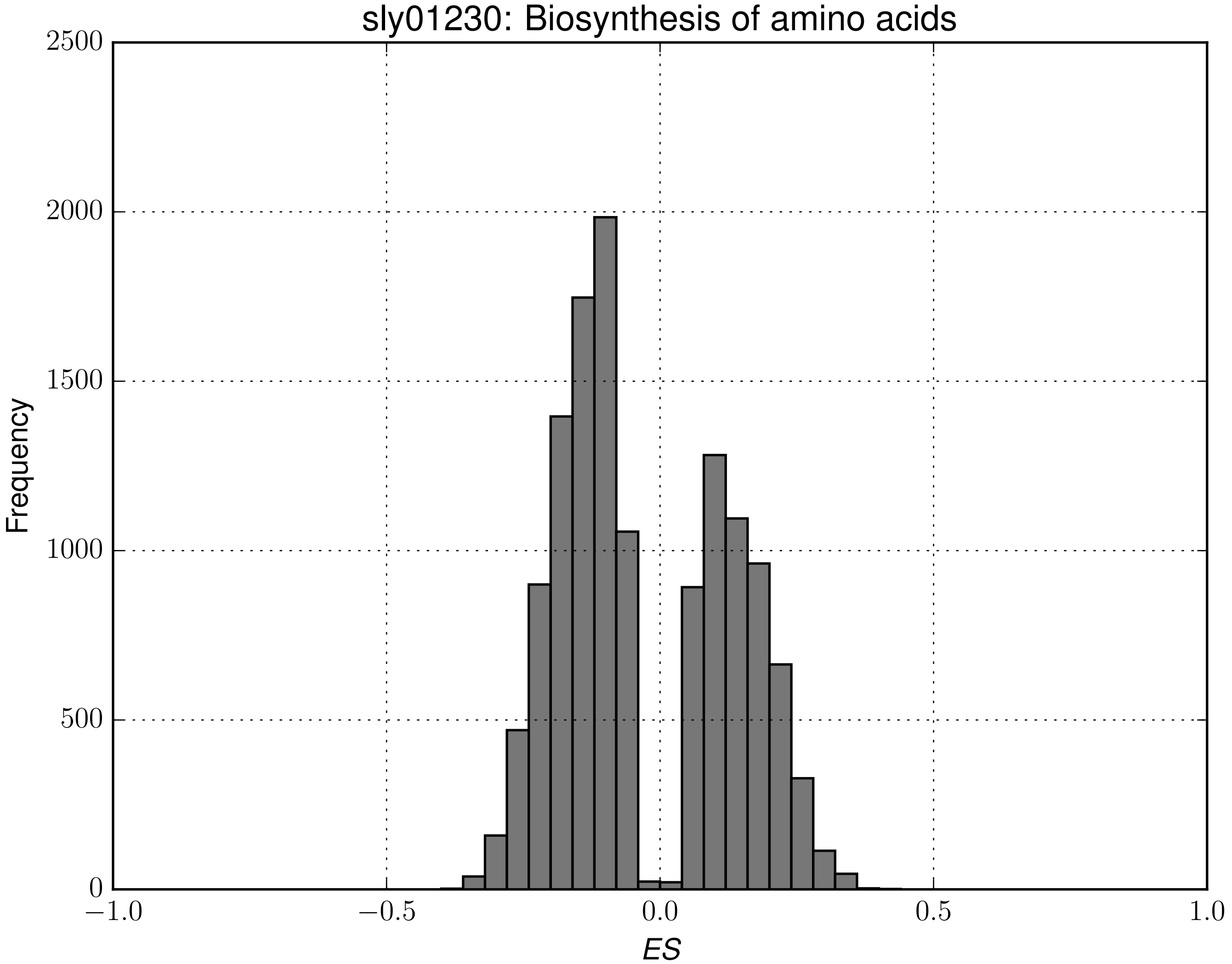
sly01230: Biosynthesis of amino acids
Frequency
ES
2500
2000
1500
1000
500
0
−1.0
−0.5
0.0
0.5
1.0

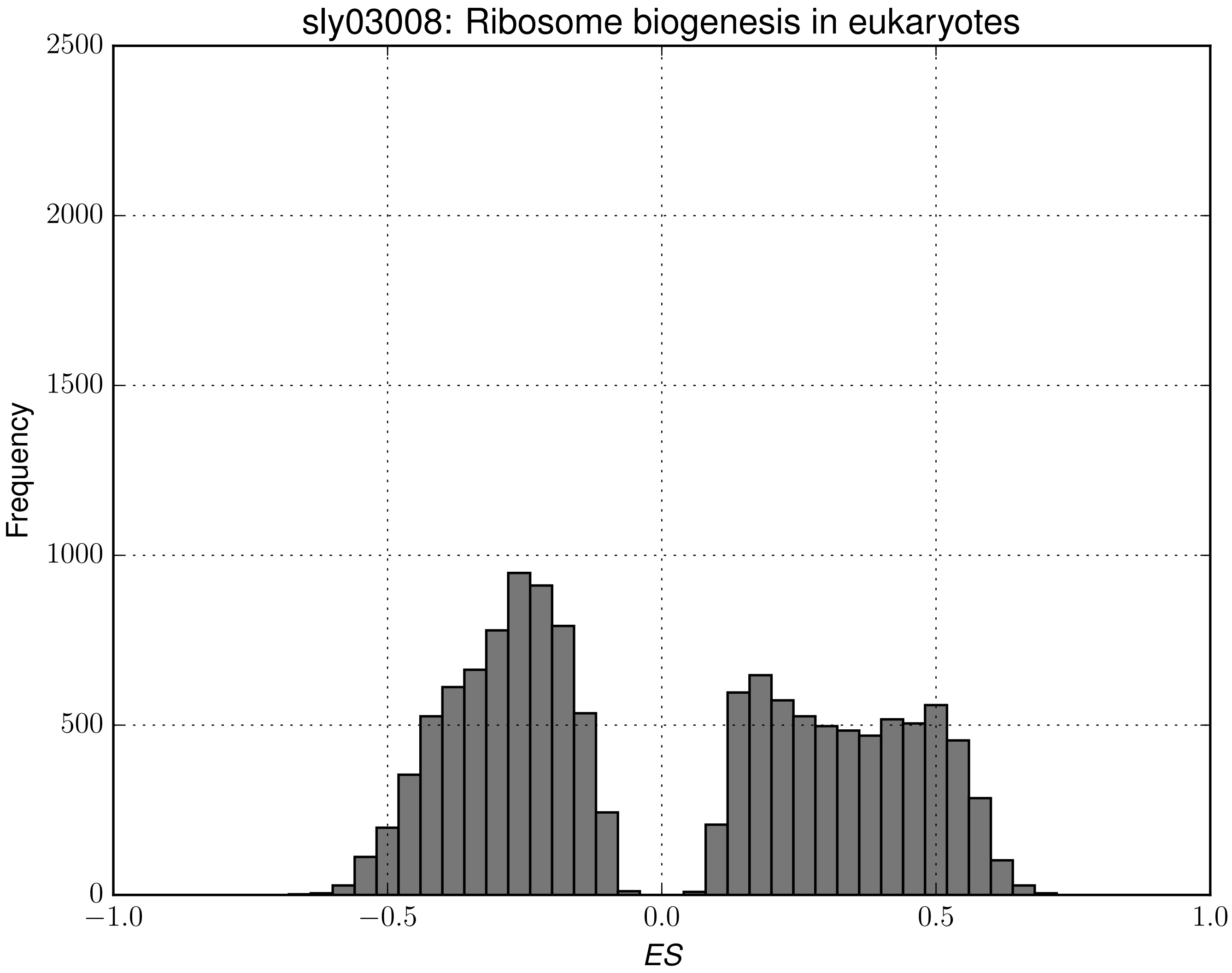

sly03008: Ribosome biogenesis in eukaryotes
Frequency
ES
2500
2000
1500
1000
500
0
−1.0
−0.5
0.0
0.5
1.0

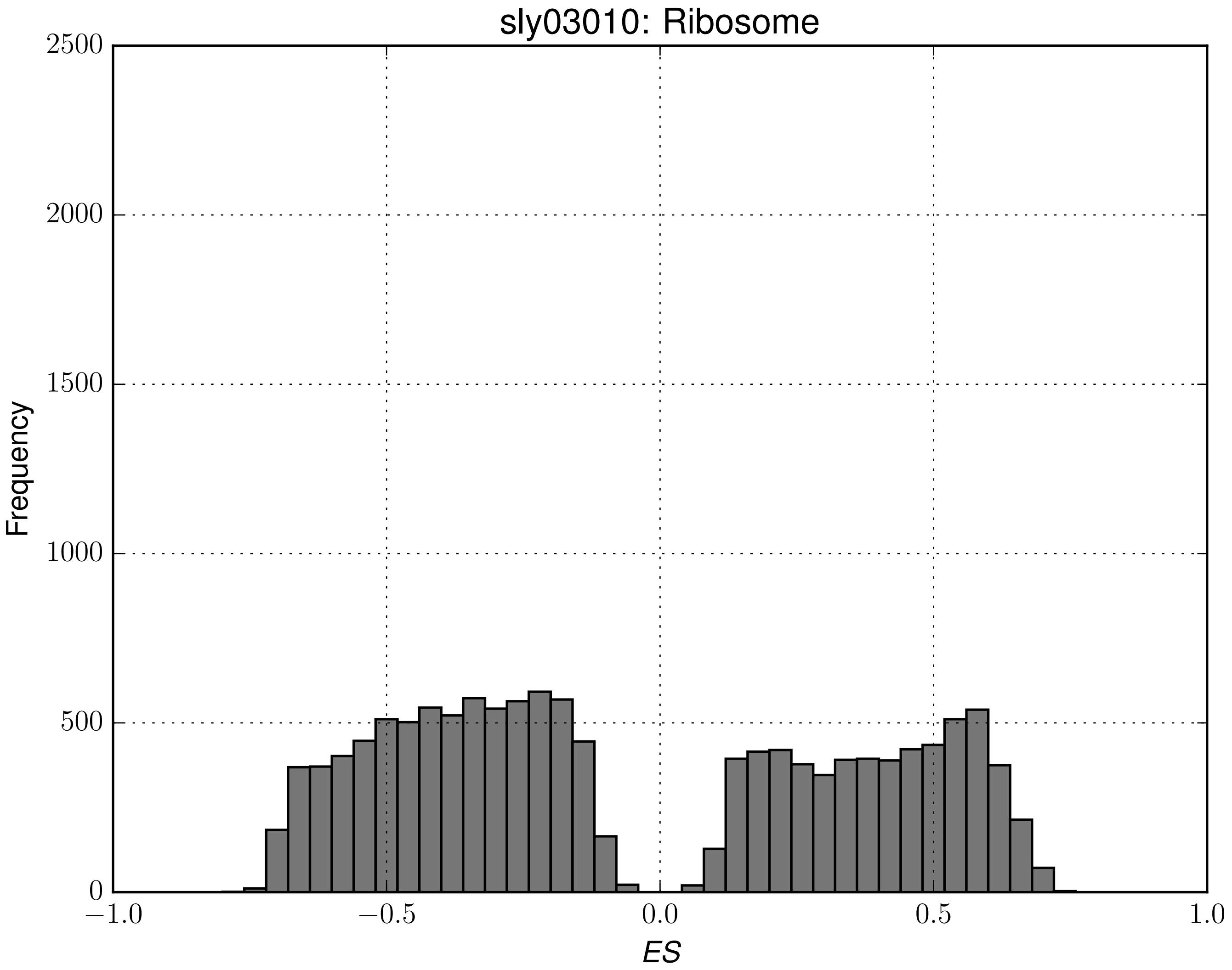
sly03010: Ribosome
Frequency
ES
2500
2000
1500
1000
500
0
−1.0
−0.5
0.0
0.5
1.0

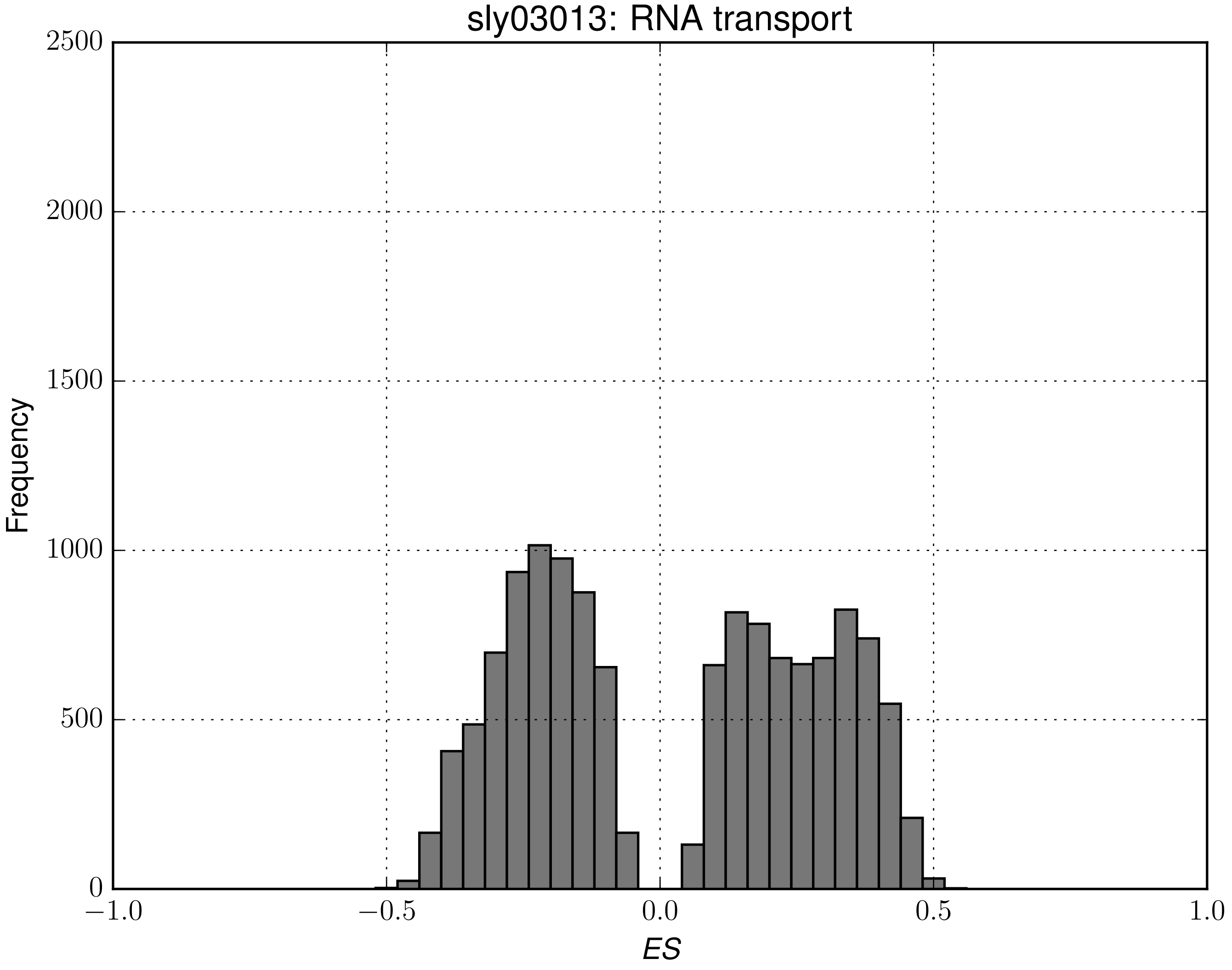

sly03013: RNA transport
Frequency
ES
2500
2000
1500
1000
500
0
−1.0
−0.5
0.0
0.5
1.0

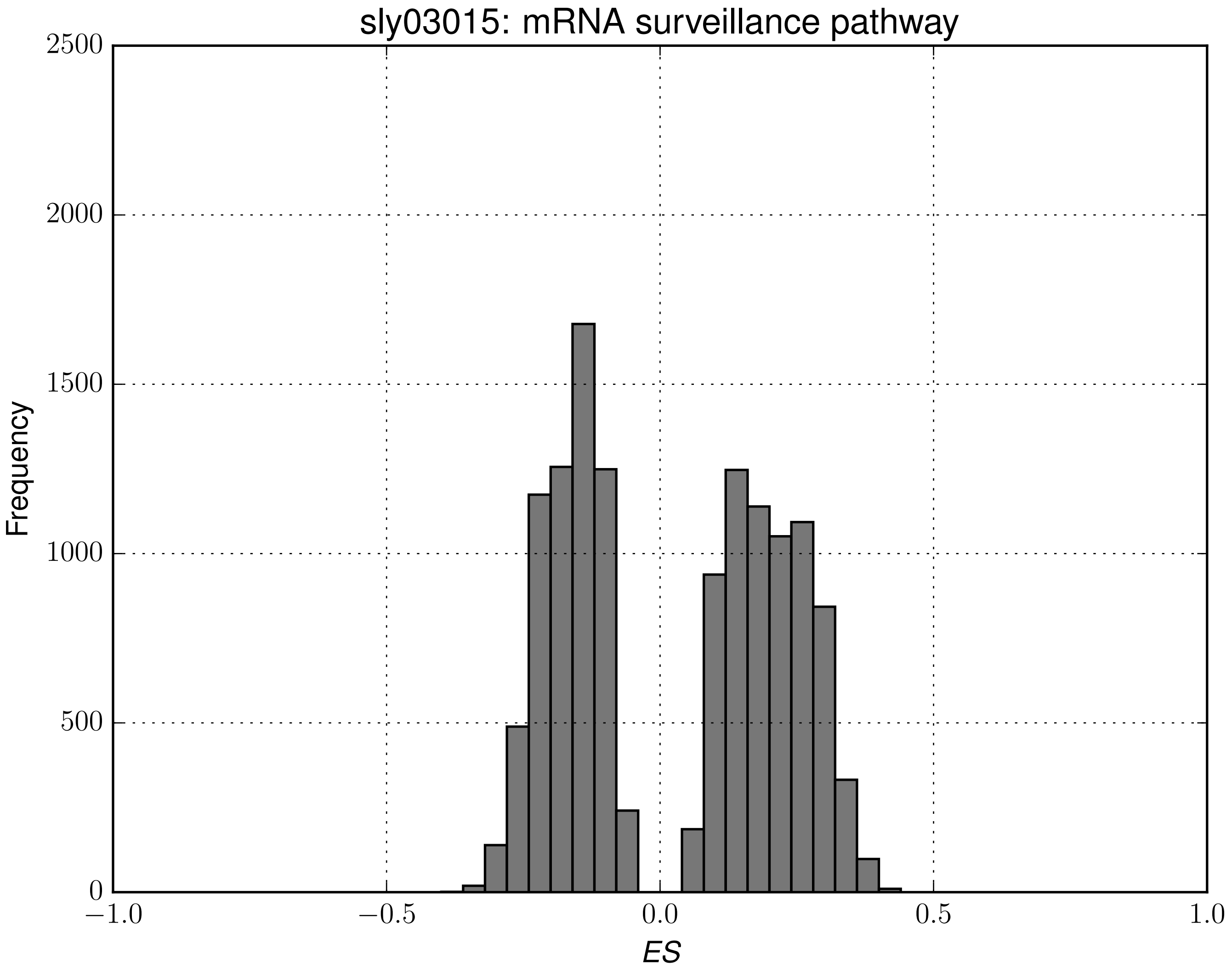
sly03015: mRNA surveillance pathway
Frequency
ES
2500
2000
1500
1000
500
0
−1.0
−0.5
0.0
0.5
1.0

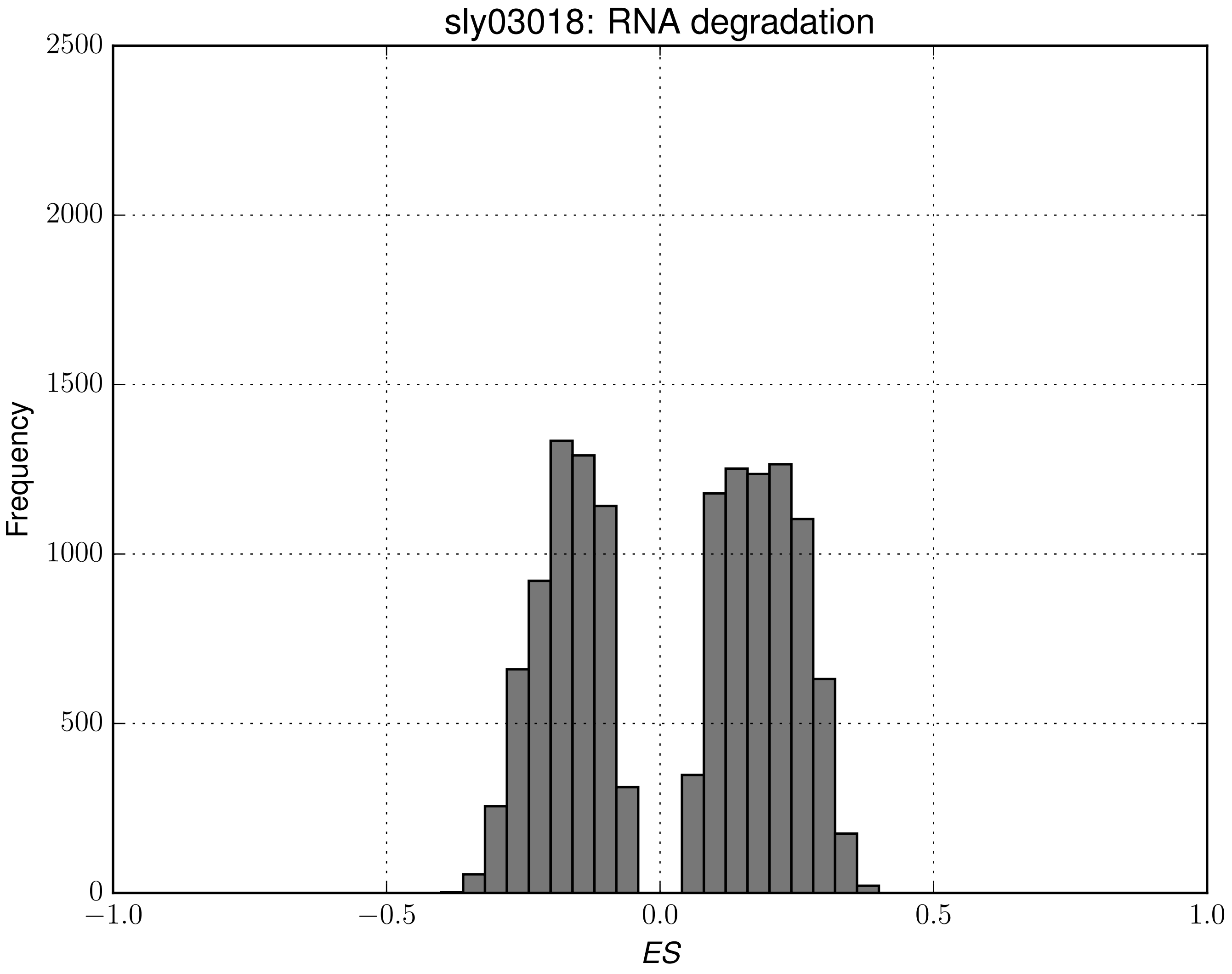
sly03018: RNA degradation
Frequency
ES
2500
2000
1500
1000
500
0
−1.0
−0.5
0.0
0.5
1.0

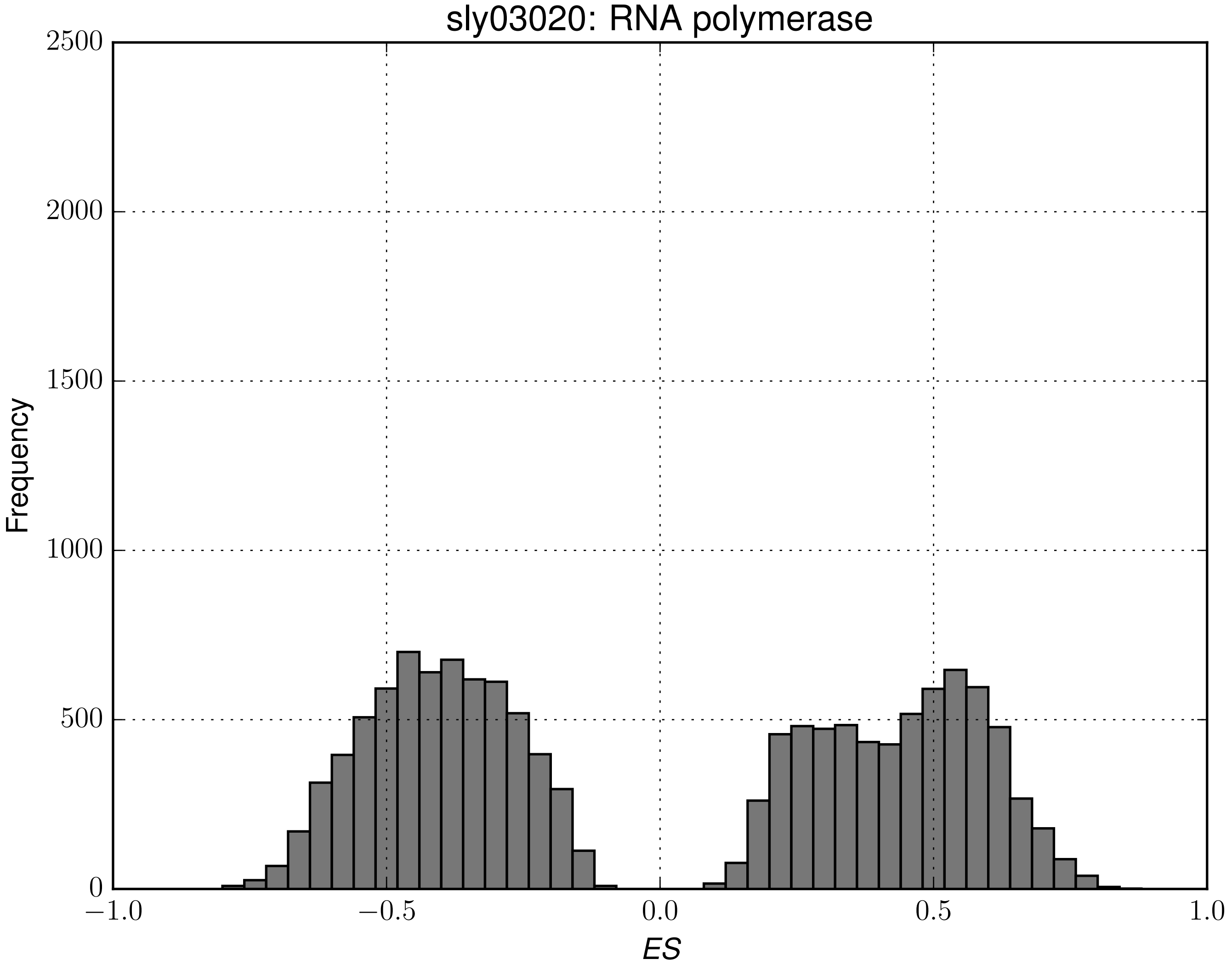

sly03020: RNA polymerase
Frequency
ES
2500
2000
1500
1000
500
0
−1.0
−0.5
0.0
0.5
1.0

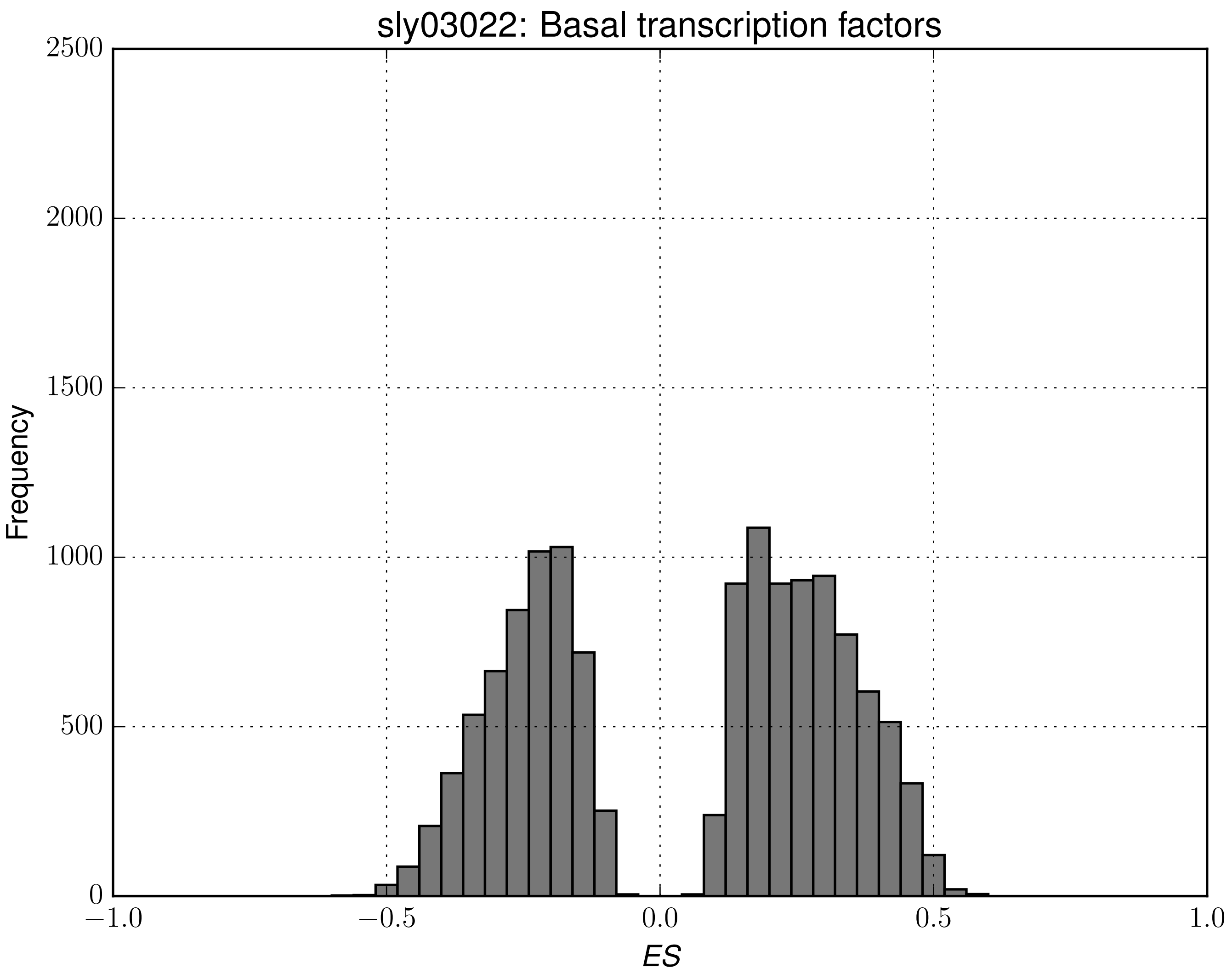
sly03022: Basal transcription factors
Frequency
ES
2500
2000
1500
1000
500
0
−1.0
−0.5
0.0
0.5
1.0

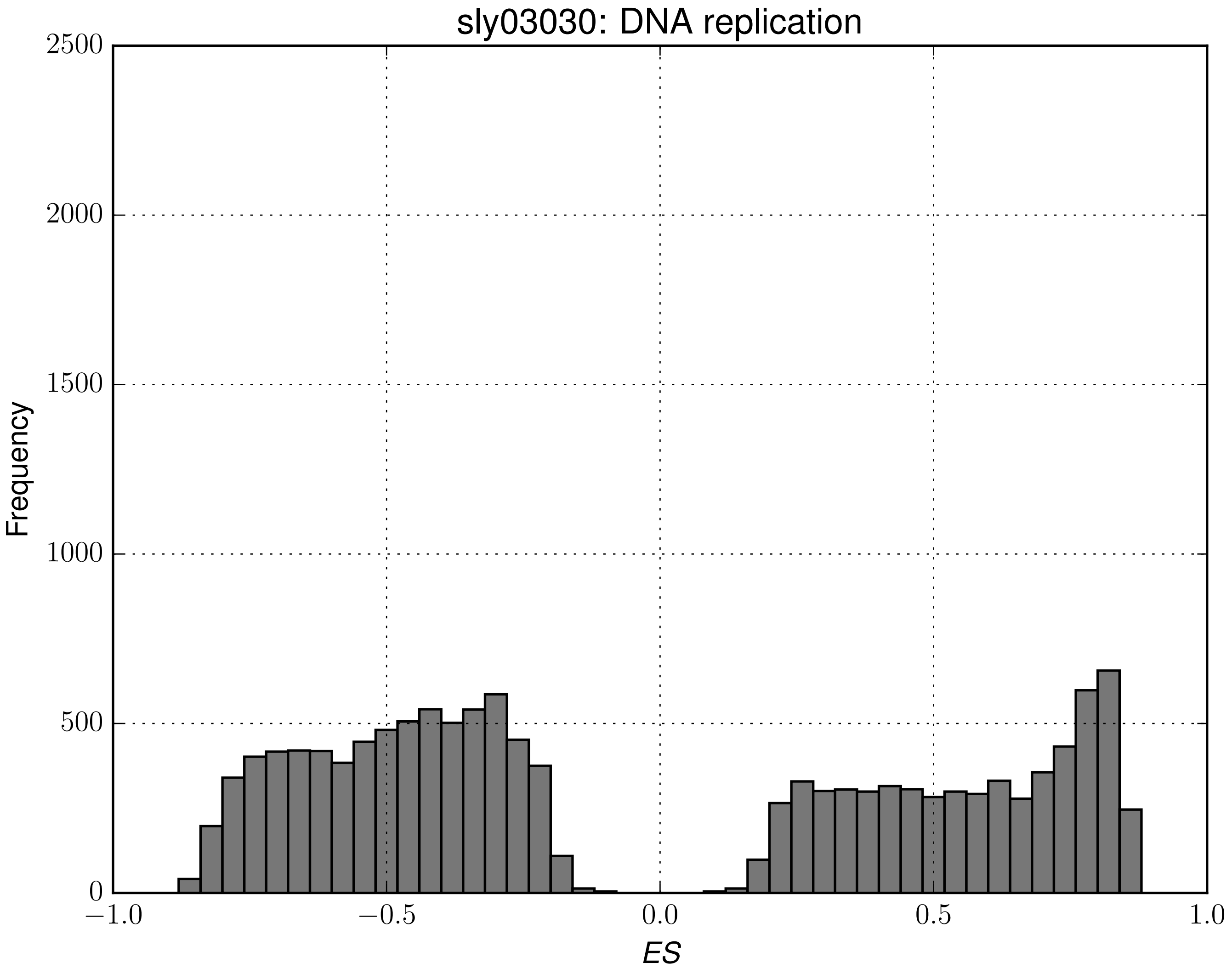

sly03030: DNA replication
Frequency
ES
2500
2000
1500
1000
500
0
−1.0
−0.5
0.0
0.5
1.0

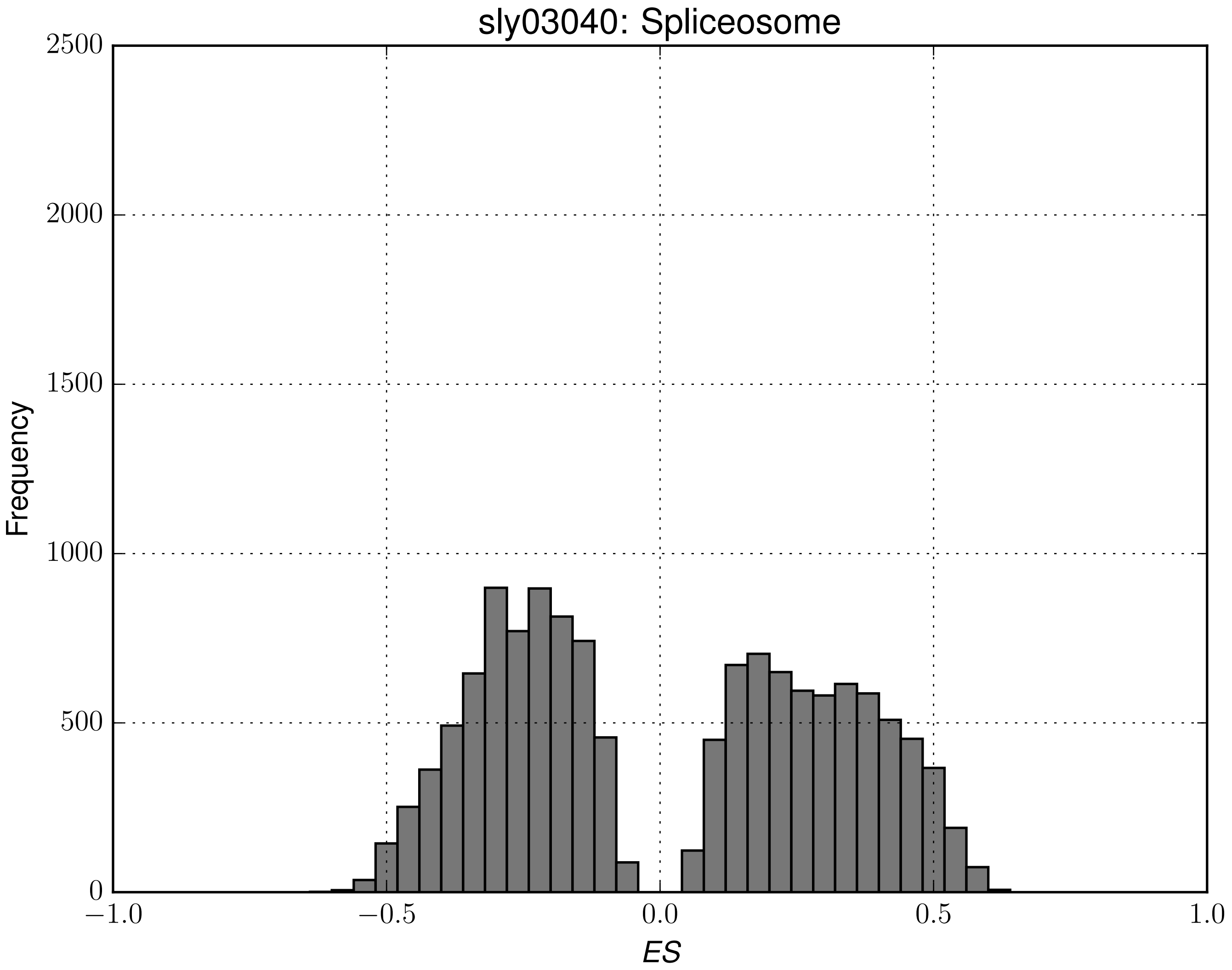

sly03040: Spliceosome
Frequency
ES
2500
2000
1500
1000
500
0
−1.0
−0.5
0.0
0.5
1.0

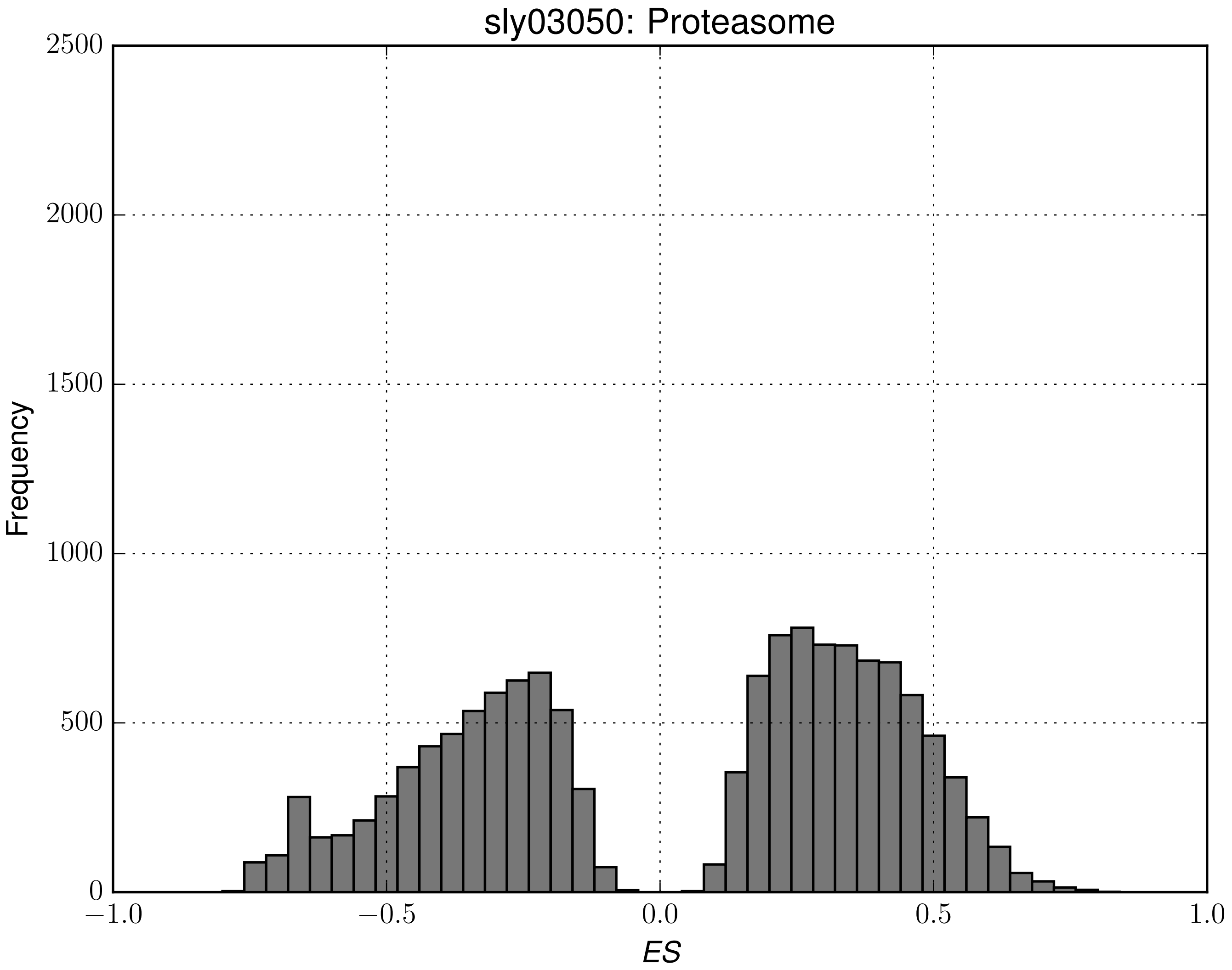

sly03050: Proteasome
Frequency
ES
2500
2000
1500
1000
500
0
−1.0
−0.5
0.0
0.5
1.0

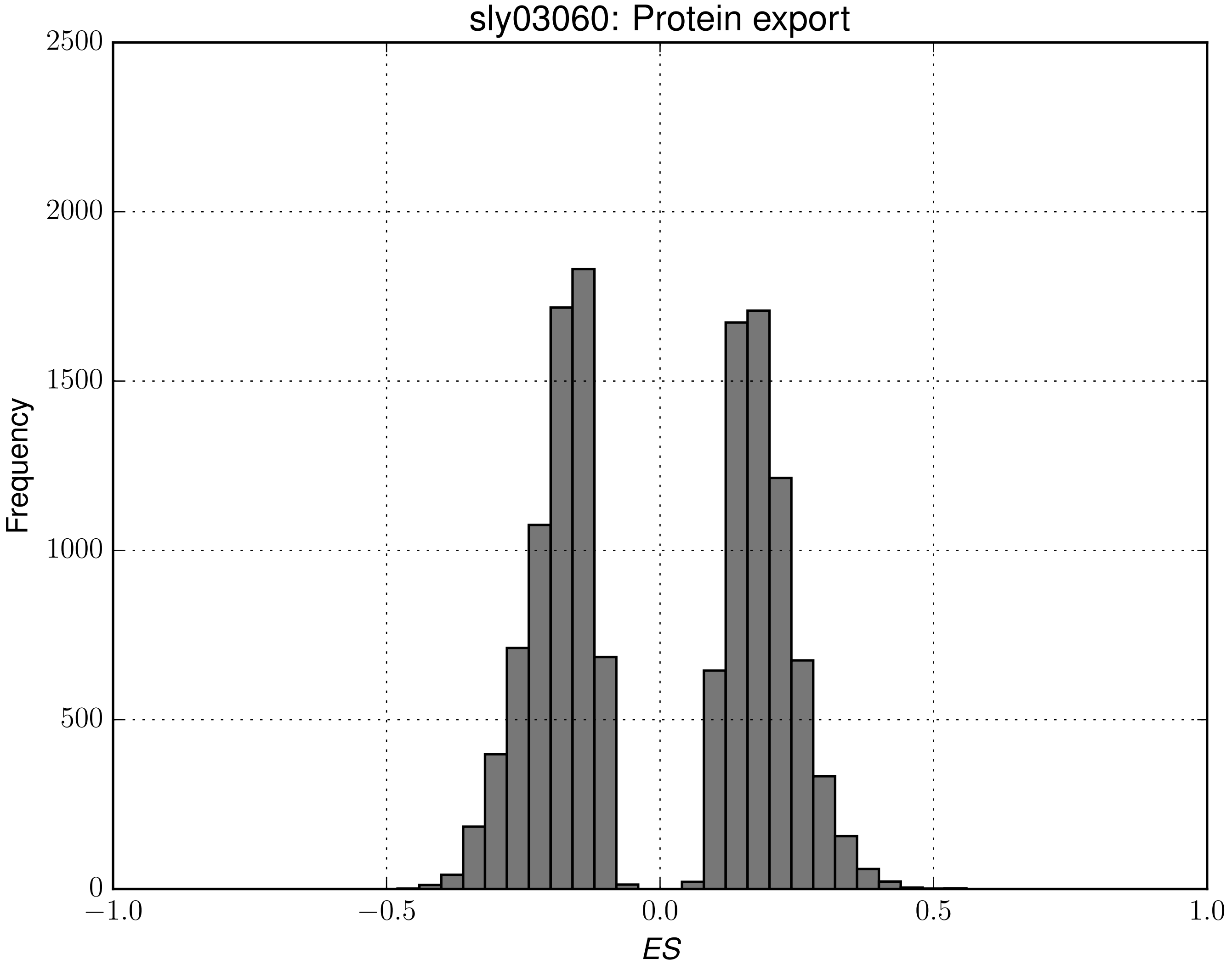

sly03060: Protein export
Frequency
ES
2500
2000
1500
1000
500
0
−1.0
−0.5
0.0
0.5
1.0

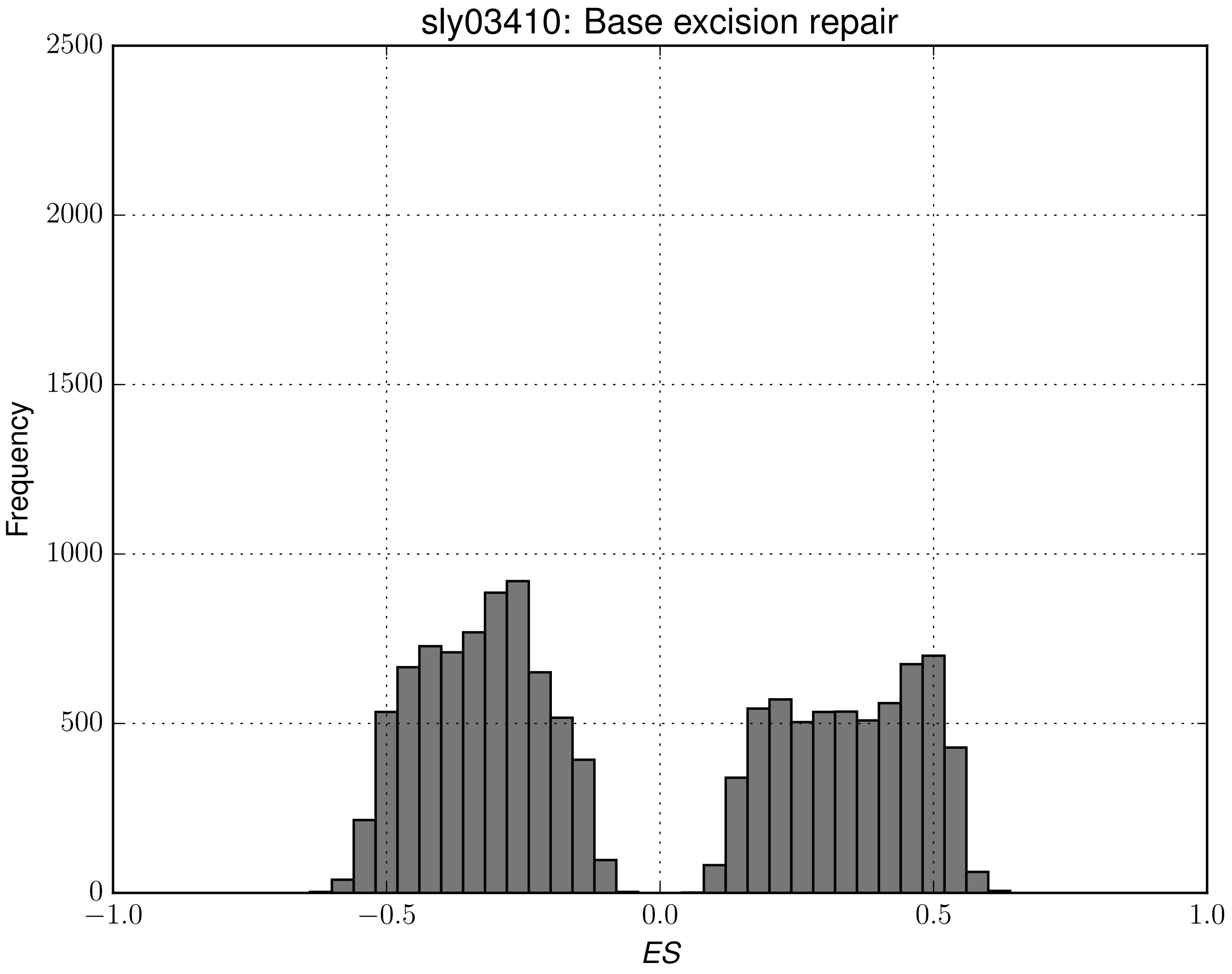
sly03410: Base excision repair
Frequency
ES
2500
2000
1500
1000
500
0
−1.0
−0.5
0.0
0.5
1.0

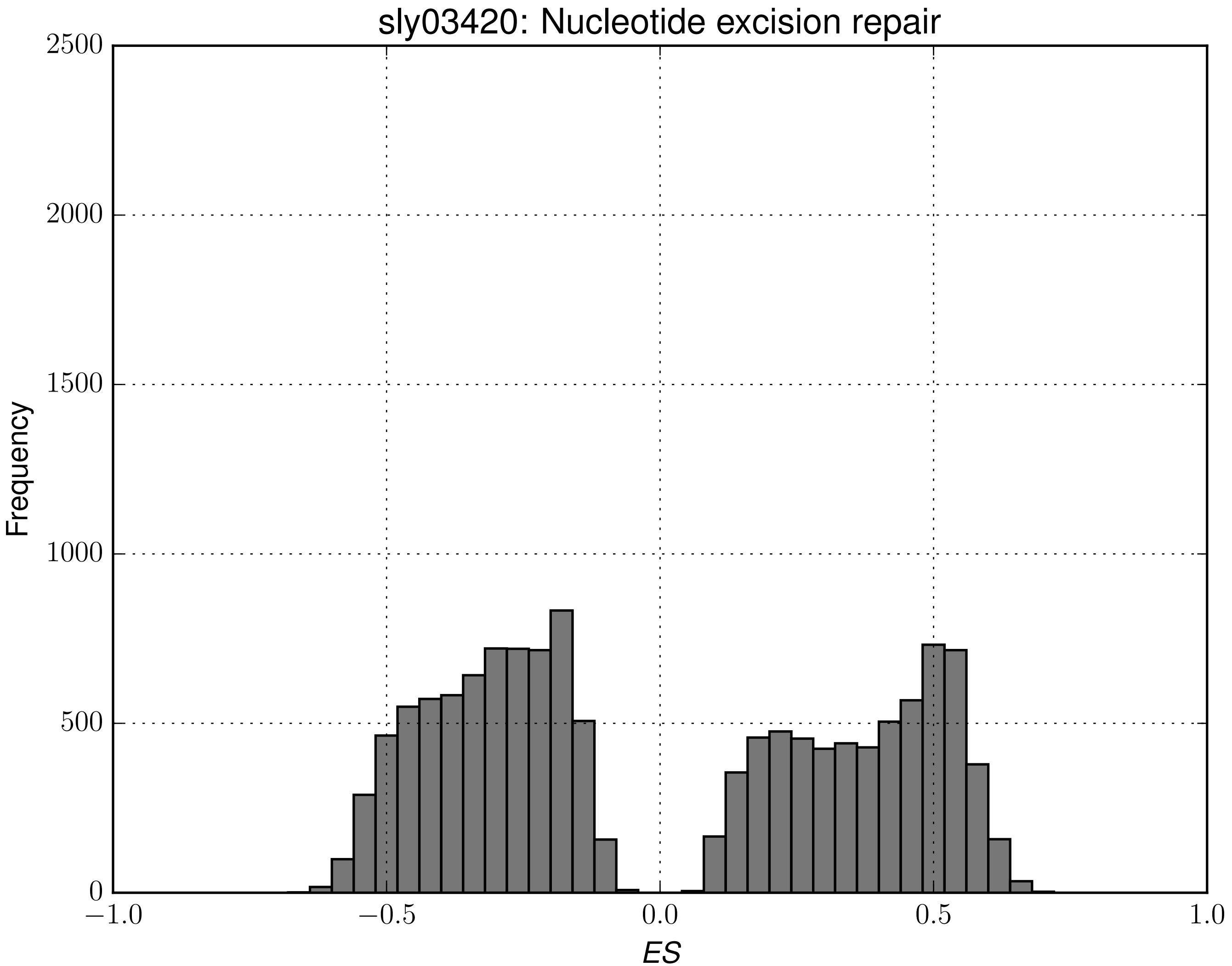

sly03420: Nucleotide excision repair
Frequency
ES
2500
2000
1500
1000
500
0
−1.0
−0.5
0.0
0.5
1.0

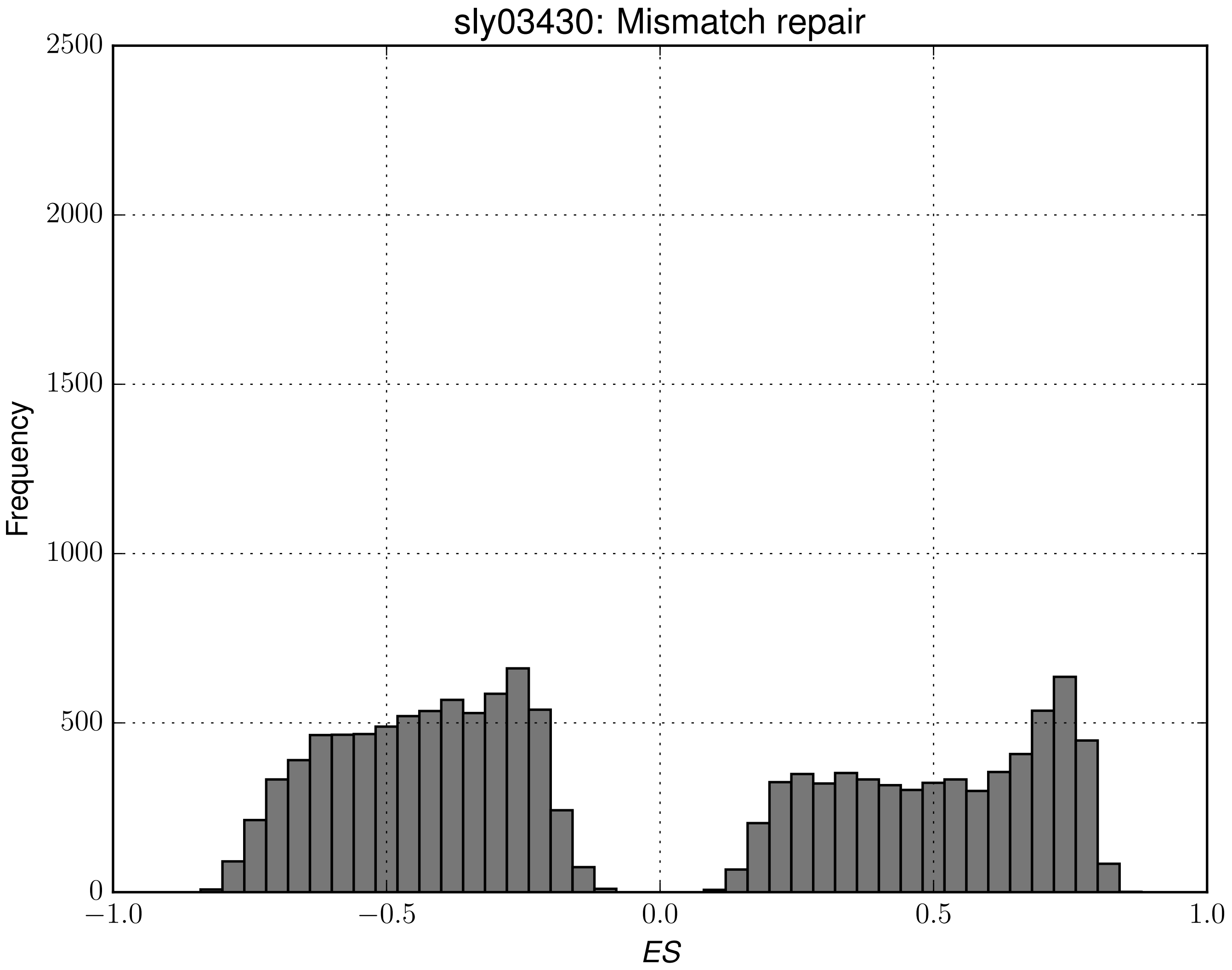
sly03430: Mismatch repair
Frequency
ES
2500
2000
1500
1000
500
0
−1.0
−0.5
0.0
0.5
1.0

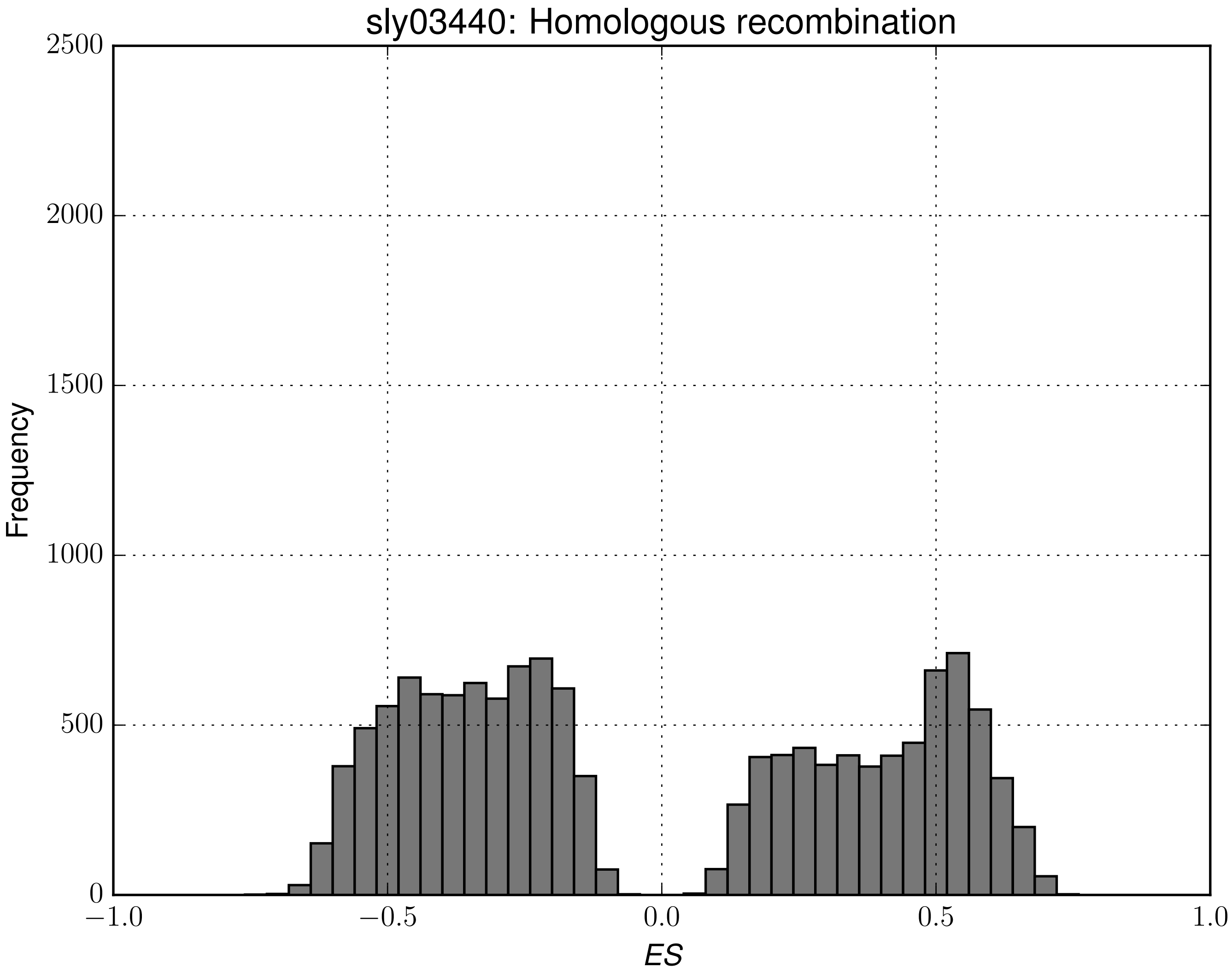
sly03440: Homologous recombination
Frequency
ES
2500
2000
1500
1000
500
0
−1.0
−0.5
0.0
0.5
1.0

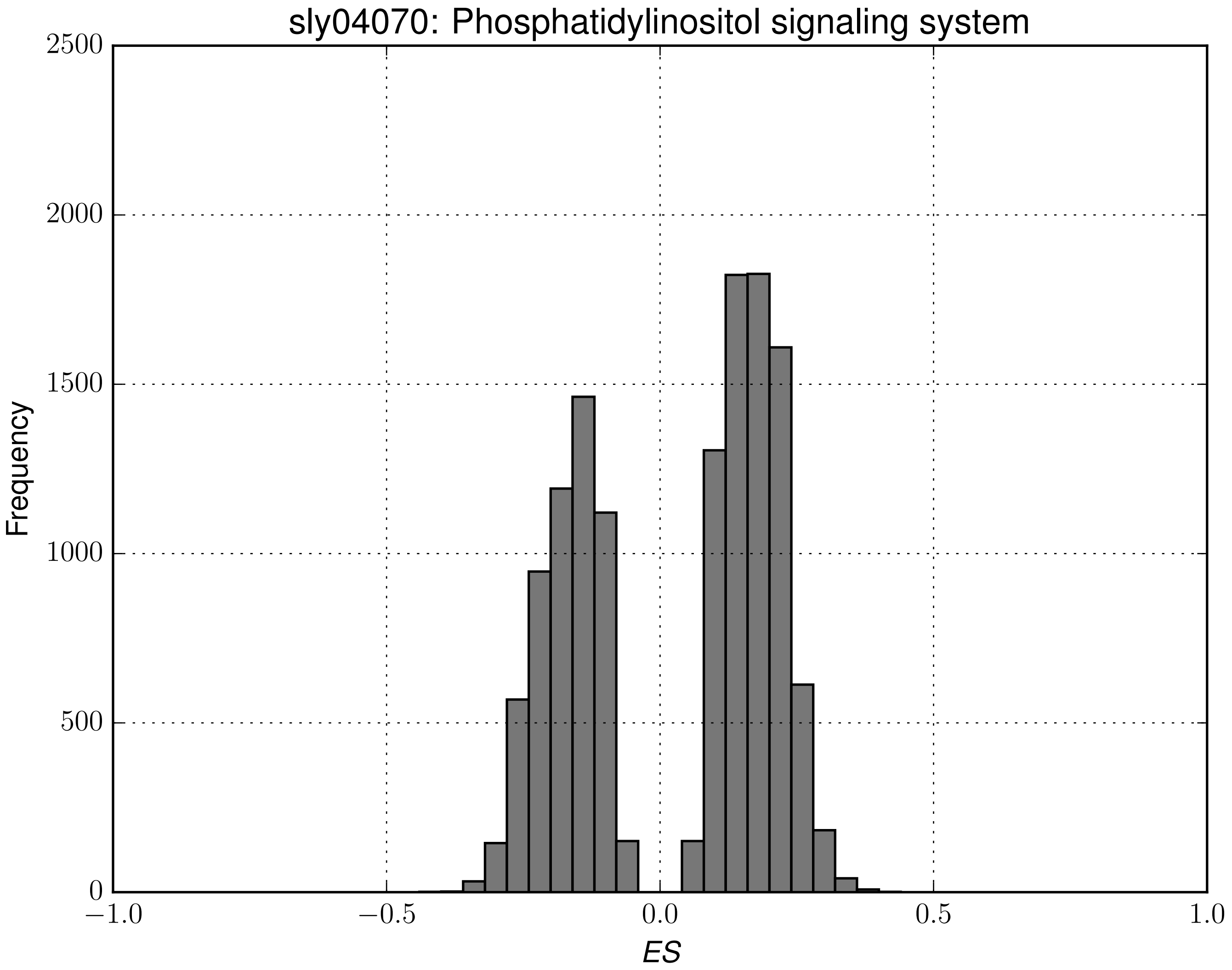
sly04070: Phosphatidylinositol signaling system
Frequency
ES
2500
2000
1500
1000
500
0
−1.0
−0.5
0.0
0.5
1.0

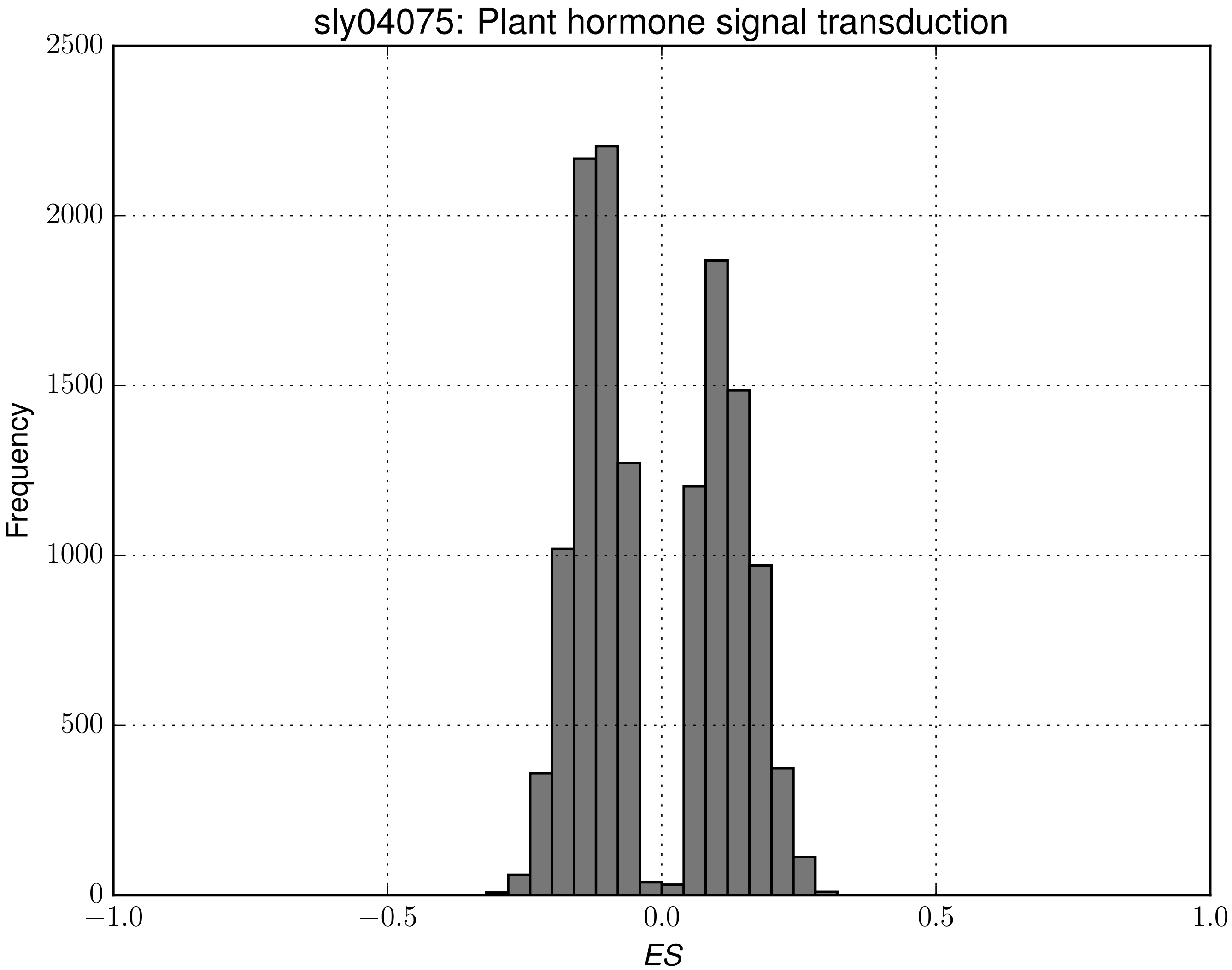

sly04075: Plant hormone signal transduction
Frequency
2500
2000
1500
1000
500
0
−1.0
−0.5
0.0
0.5
1.0
ES

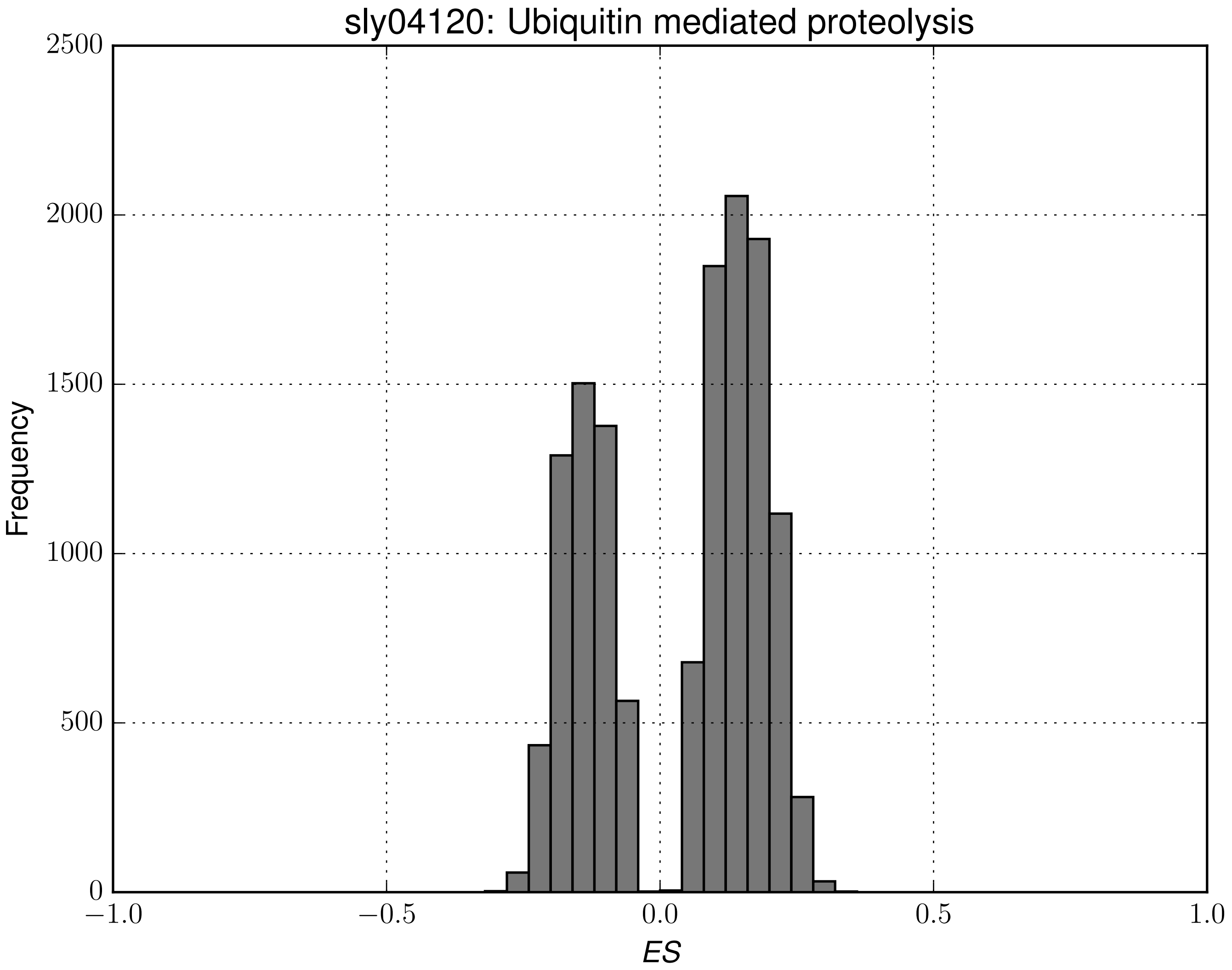

sly04120: Ubiquitin mediated proteolysis
Frequency
ES
2500
2000
1500
1000
500
0
−1.0
−0.5
0.0
0.5
1.0

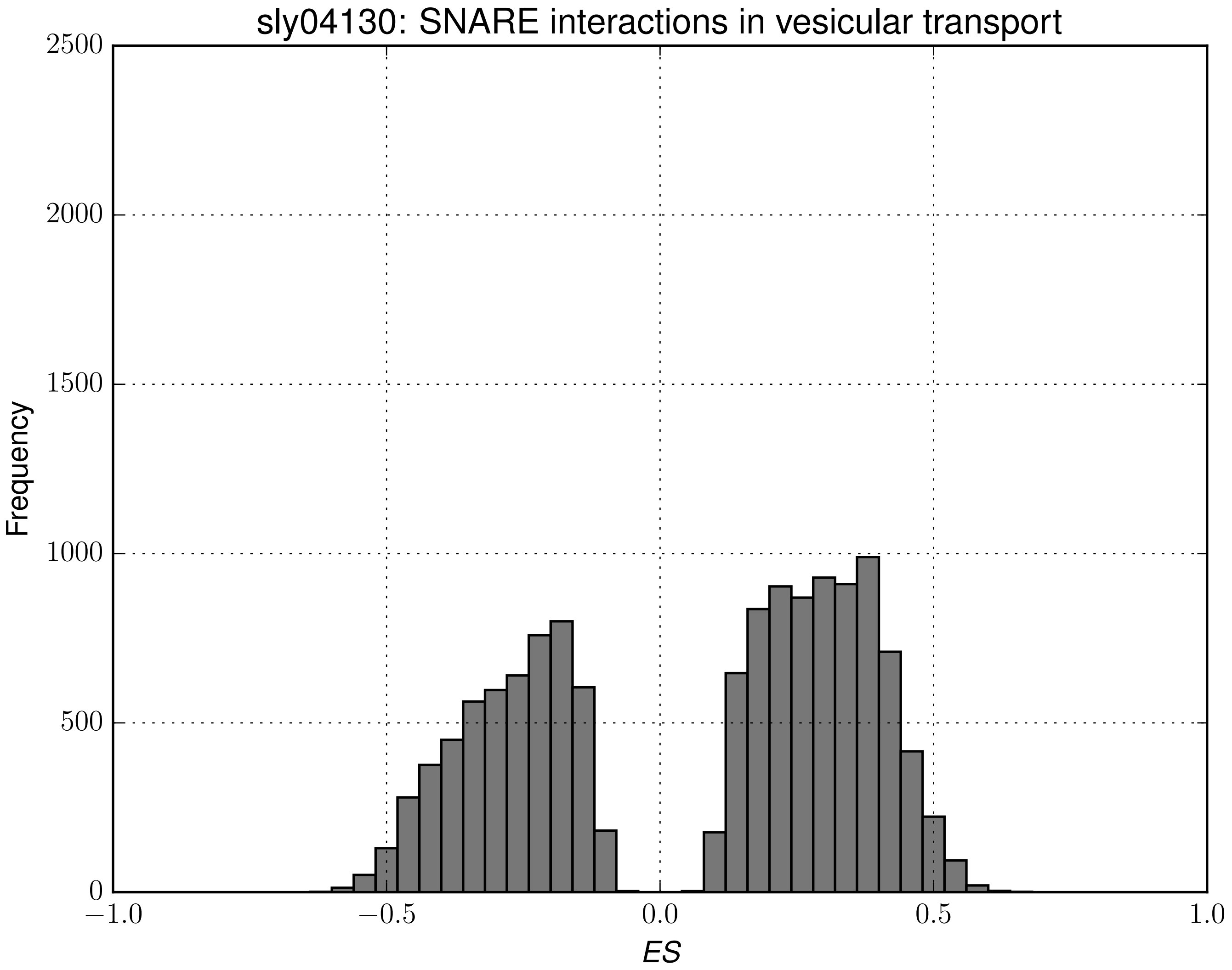

sly04130: SNARE interactions in vesicular transport
Frequency
ES
0
500
1000
1500
2000
2500
−1.0
−0.5
0.0
0.5
1.0

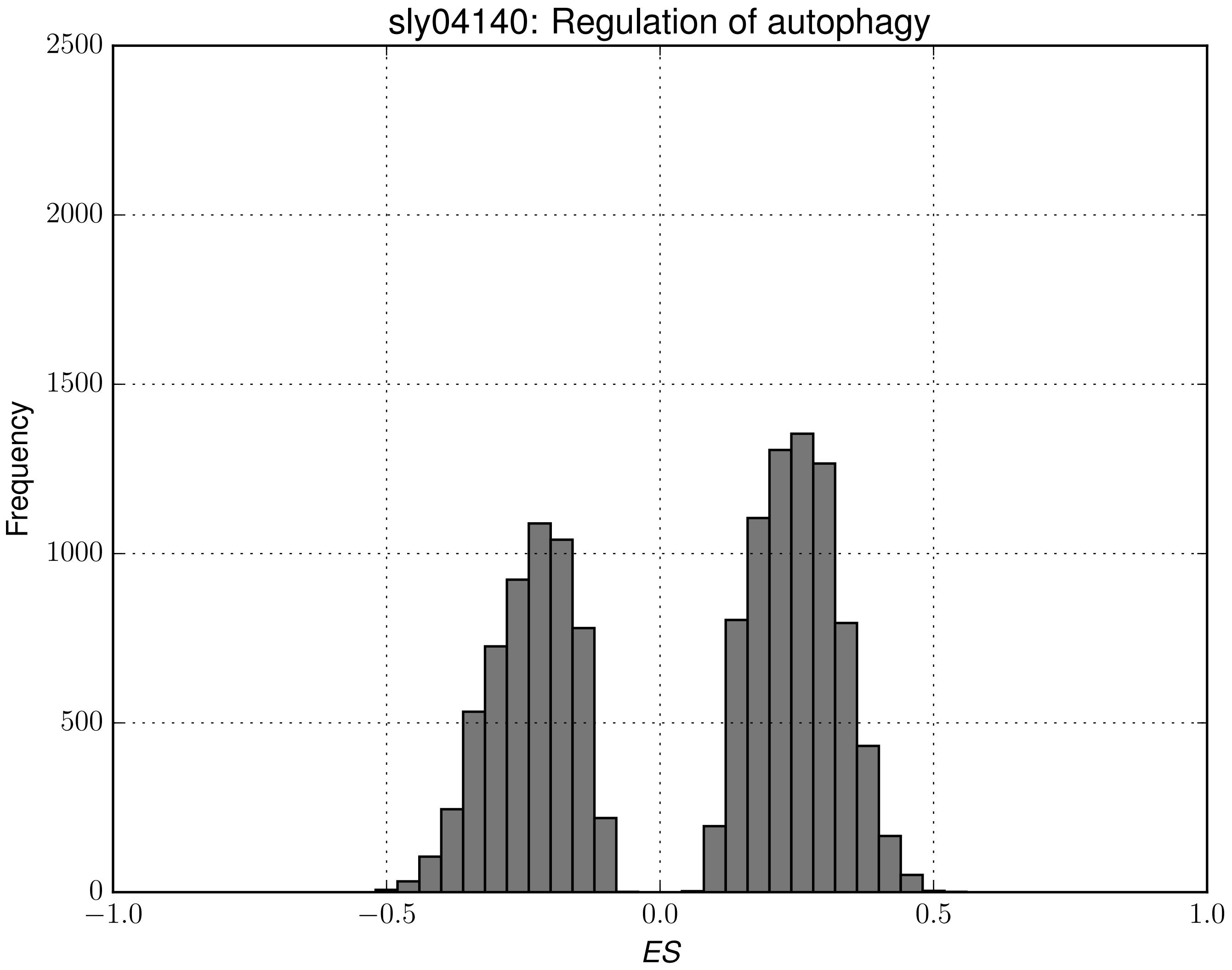
sly04140: Regulation of autophagy
Frequency
2500
2000
1500
1000
500
0
−1.0
−0.5
0.0
0.5
1.0
ES

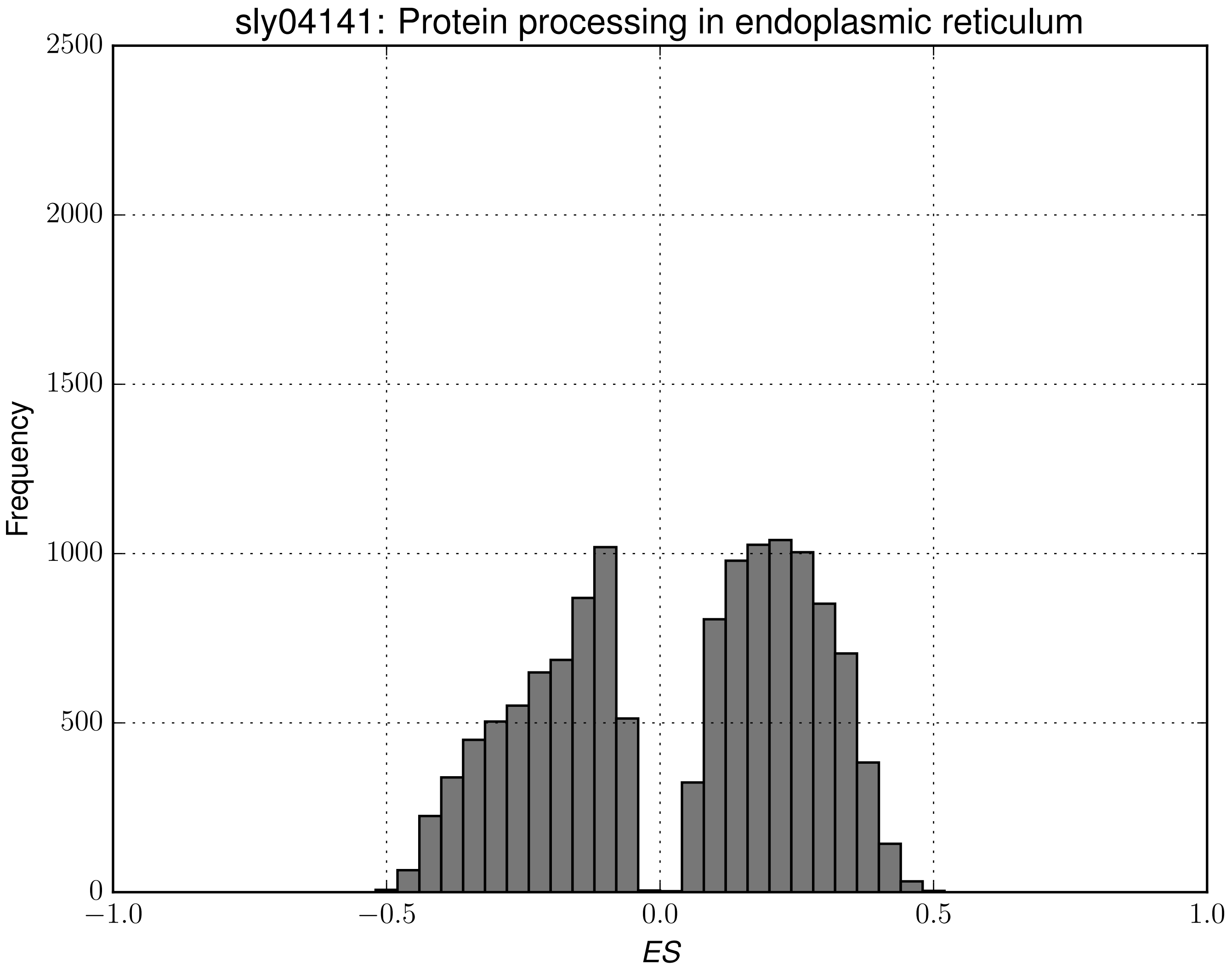
sly04141: Protein processing in endoplasmic reticulum
Frequency
ES
0
500
1000
1500
2000
2500
−1.0
−0.5
0.0
0.5
1.0

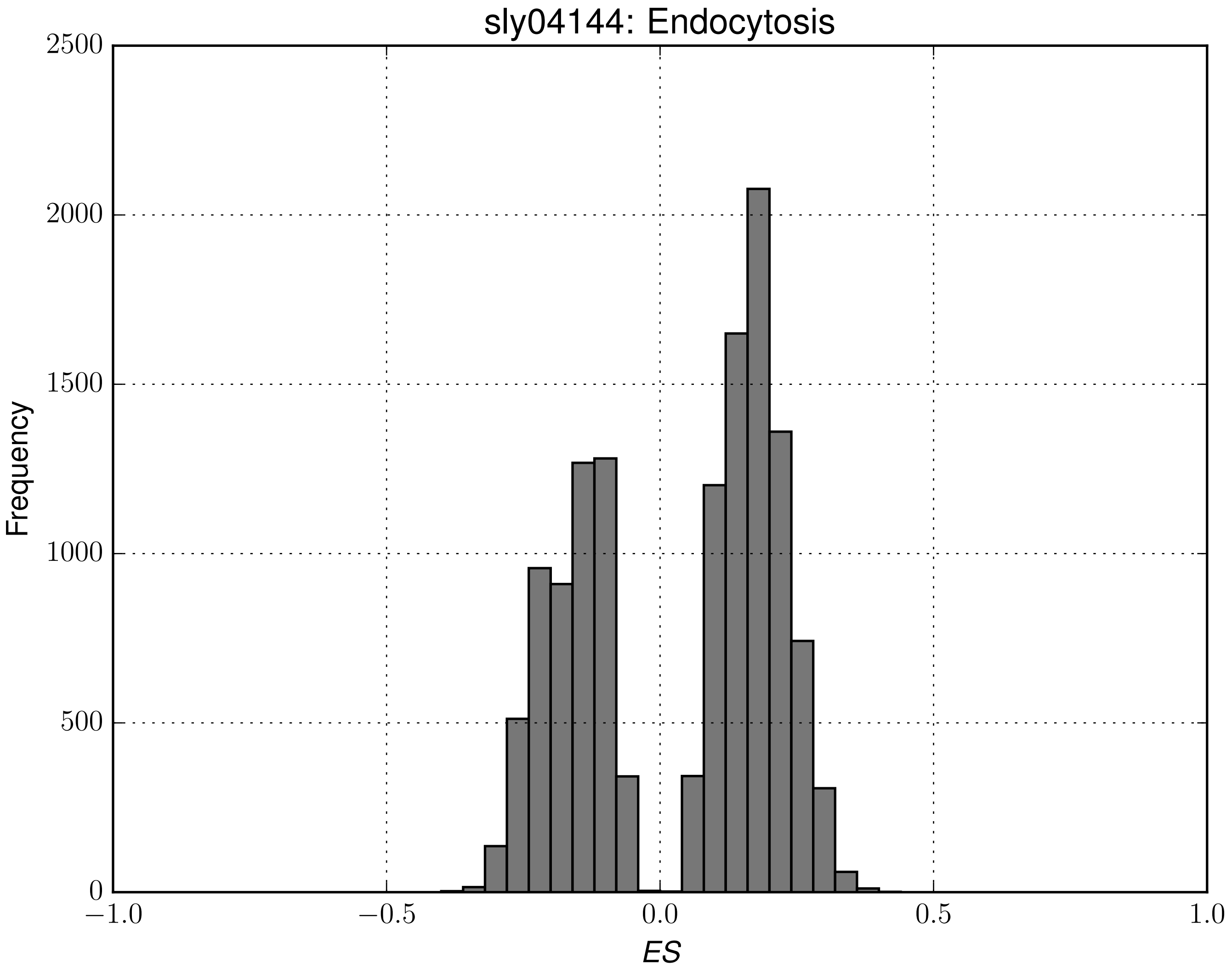

sly04144: Endocytosis
2500
2000
1500
1000
500
0
Frequency
−1.0
−0.5
0.0
0.5
1.0
ES

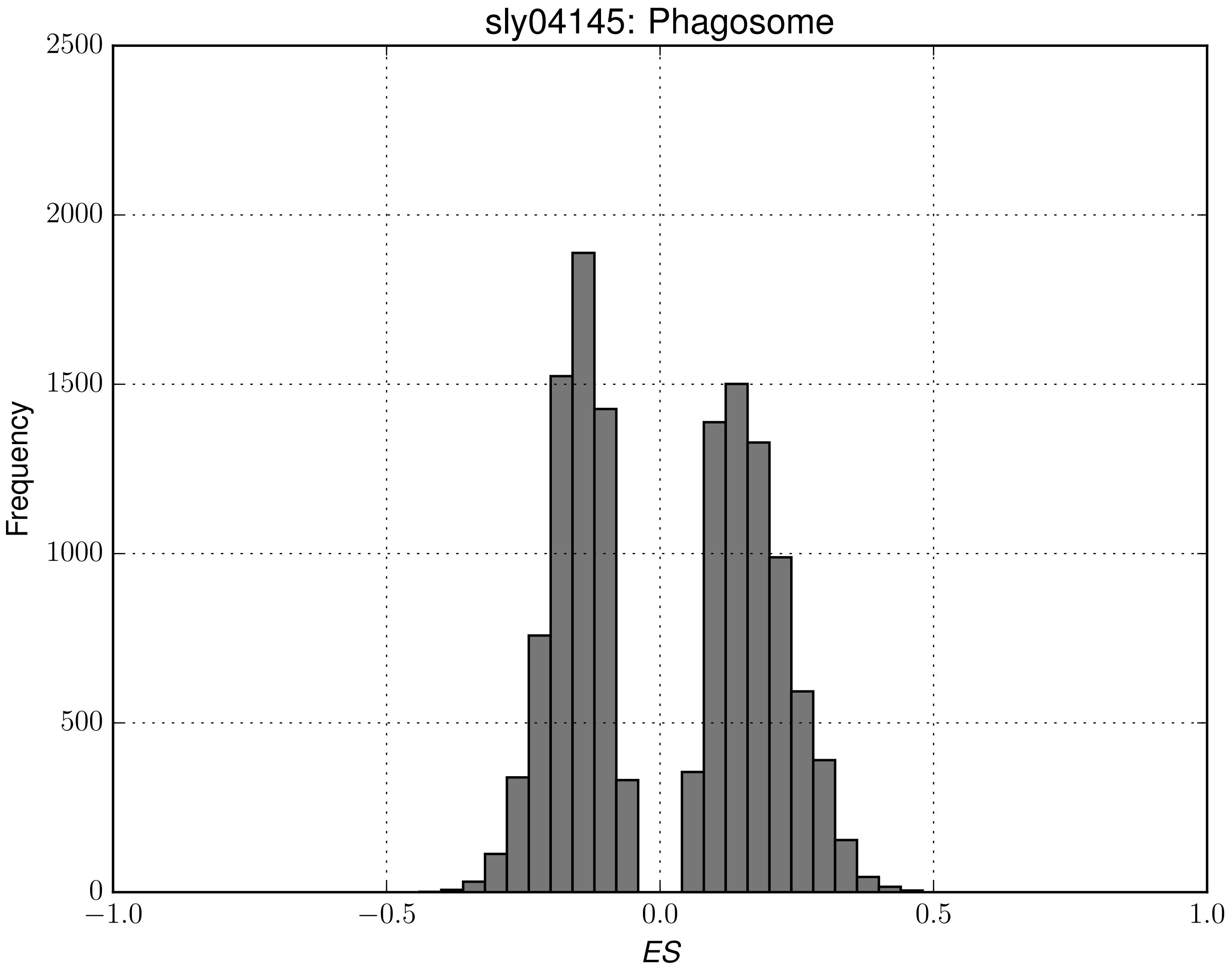
sly04145: Phagosome
Frequency
ES
2500
2000
1500
1000
500
0
−1.0
−0.5
0.0
0.5
1.0

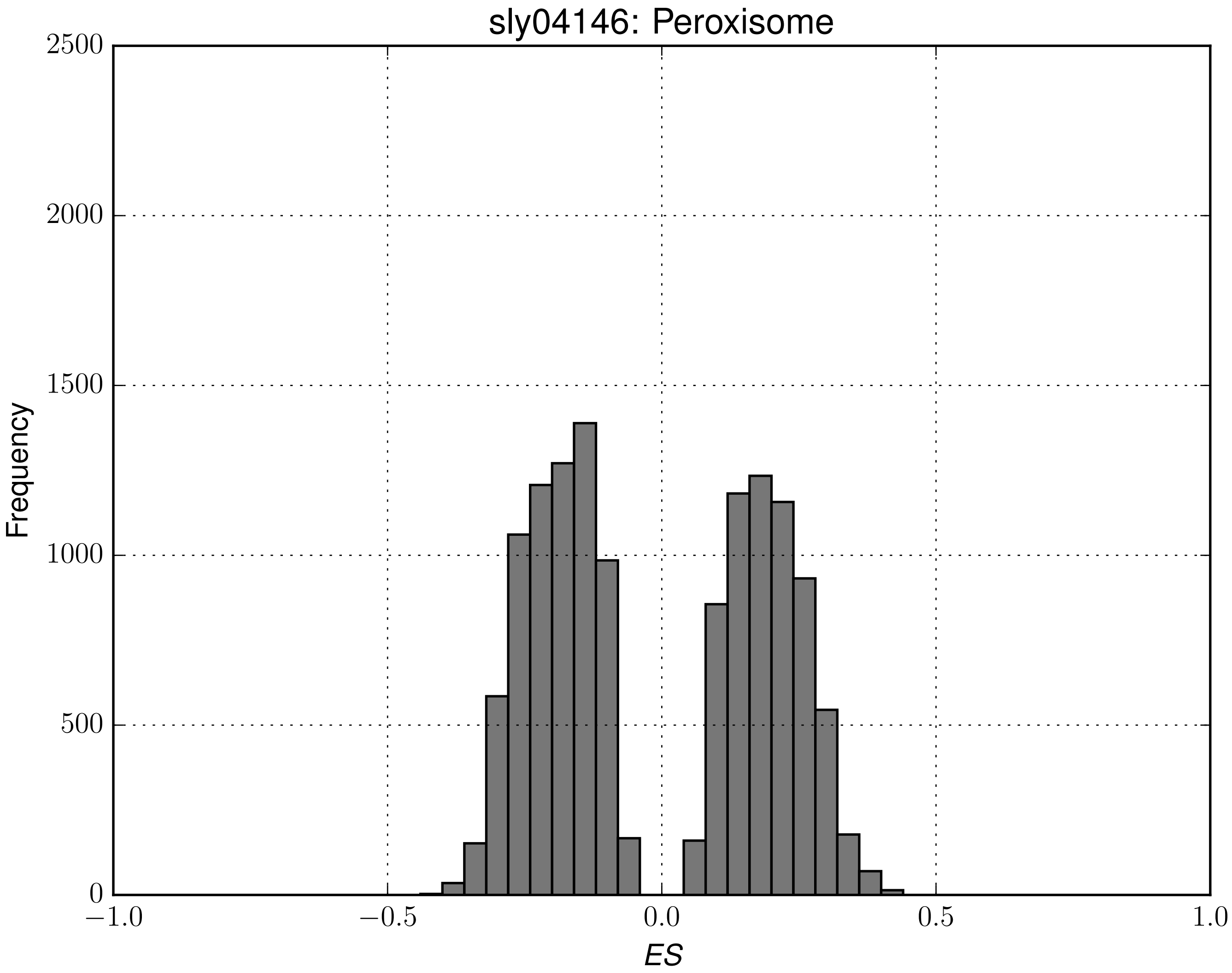

sly04146: Peroxisome
Frequency
ES
2500
2000
1500
1000
500
0
−1.0
−0.5
0.0
0.5
1.0

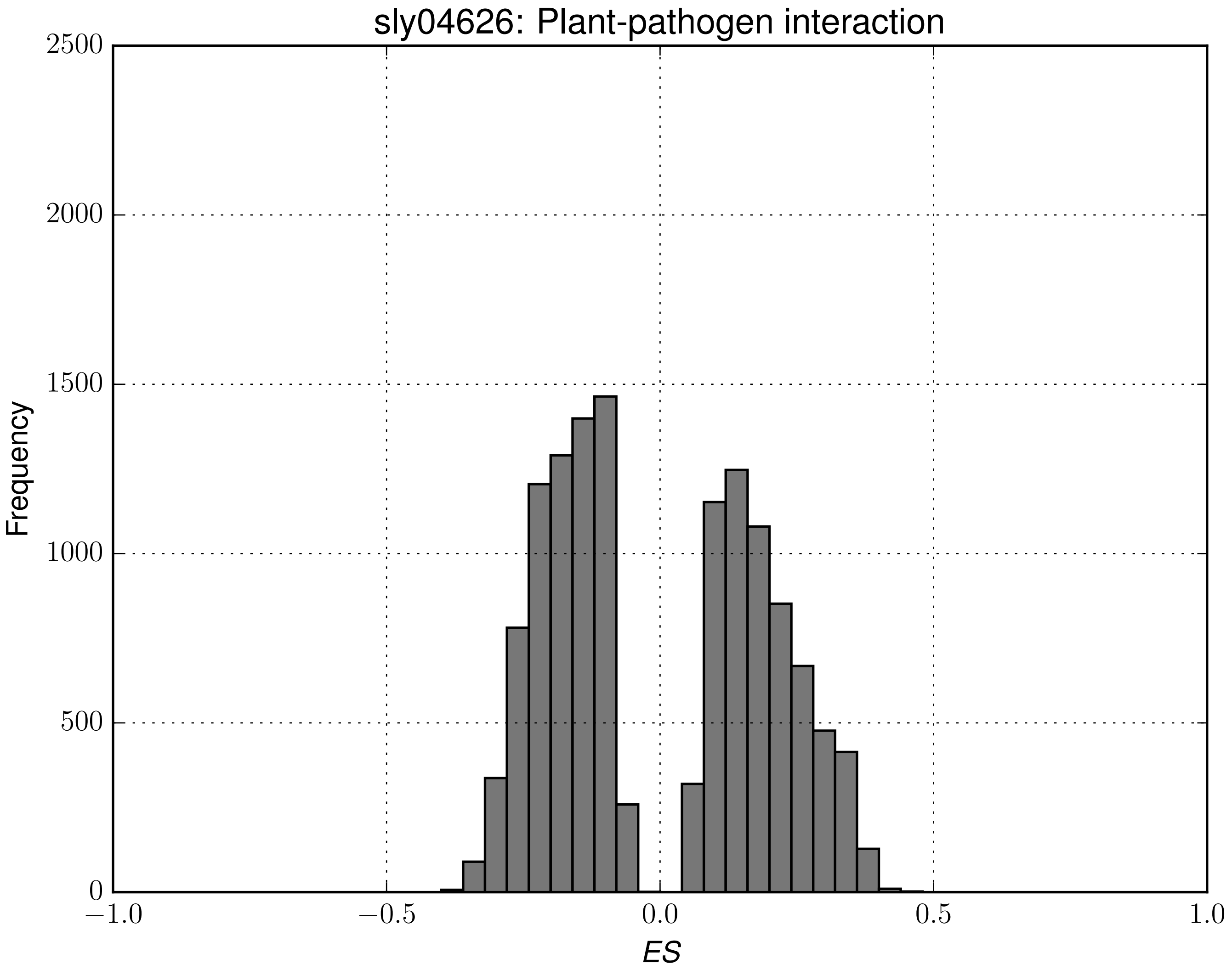

sly04626: Plant-pathogen interaction
Frequency
ES
2500
2000
1500
1000
500
0
−1.0
−0.5
0.0
0.5
1.0

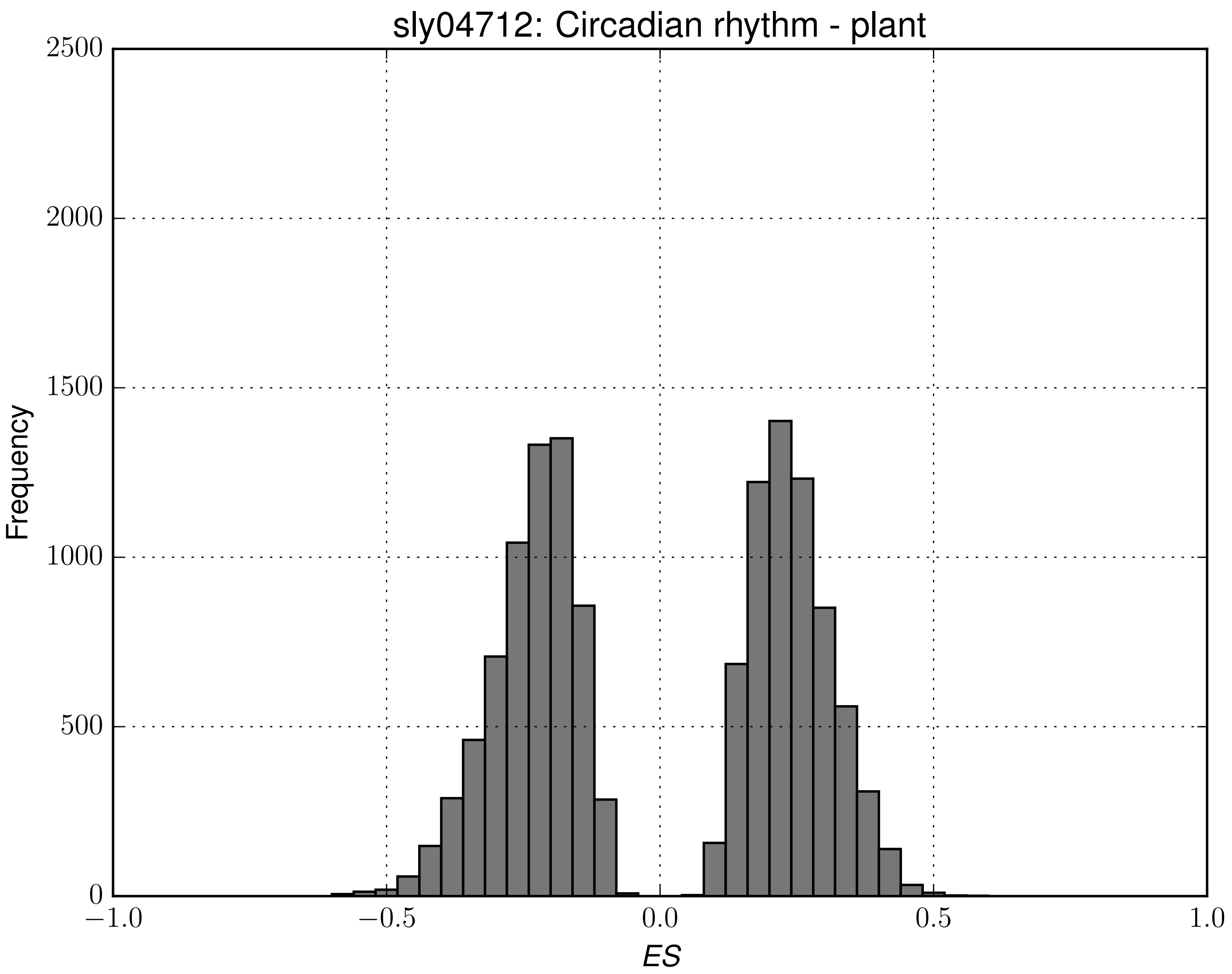
sly04712: Circadian rhythm - plant
Frequency
2500
2000
1500
1000
500
0
−1.0
−0.5
0.0
0.5
1.0
ES
