# Supplementary material for: Co-expressed Pathways DataBase for Tomato: a database to predict pathways relevant to a query gene
Source: BMC Genomics. 2017 Jun 5;18:437. doi: 10.1186/s12864-017-3786-3 (PMC5460524; doi:10.1186/s12864-017-3786-3)
Supplement: Supplementary file 7 — Distribution of the p-score. The frequency distribution of p-score for each KEGG pathway. (PDF 352 kb) [file 12864_2017_3786_MOESM7_ESM.pdf]

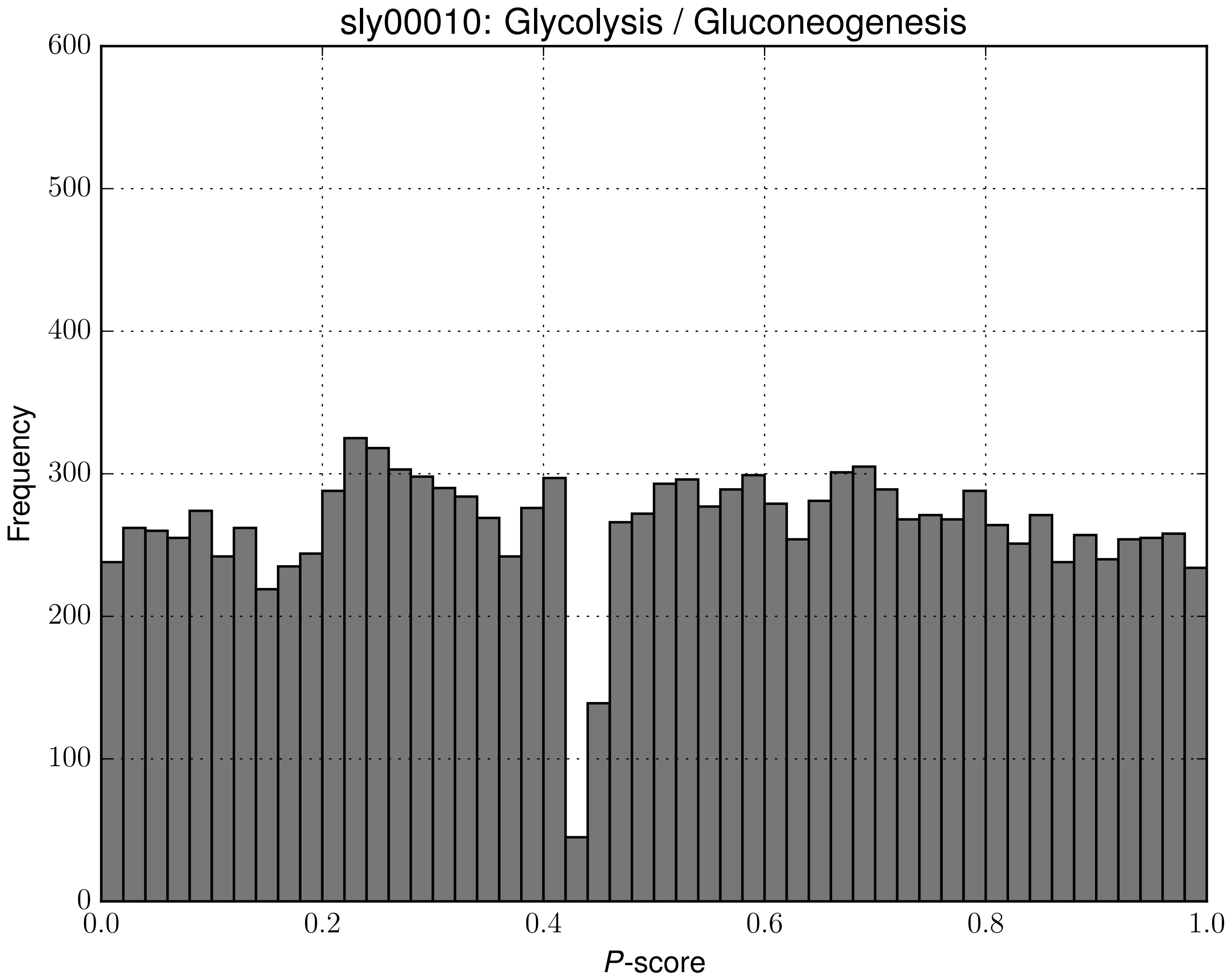
sly00010: Glycolysis / Gluconeogenesis
Frequency
P-score
0
100
200
300
400
500
600
0.0
0.2
0.4
0.6
0.8
1.0

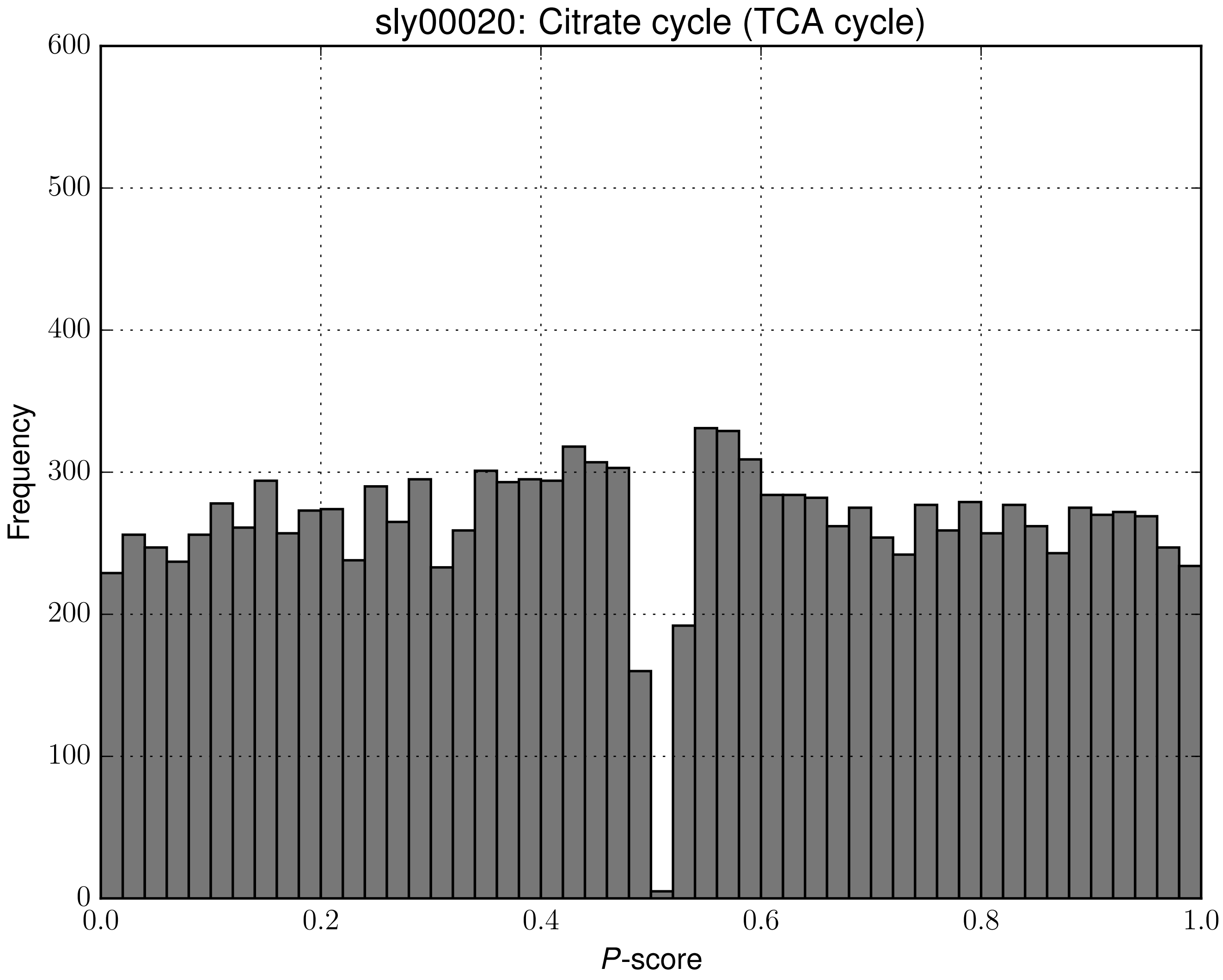
sly00020: Citrate cycle (TCA cycle)
Frequency
P-score
0
100
200
300
400
500
600
0.0
0.2
0.4
0.6
0.8
1.0

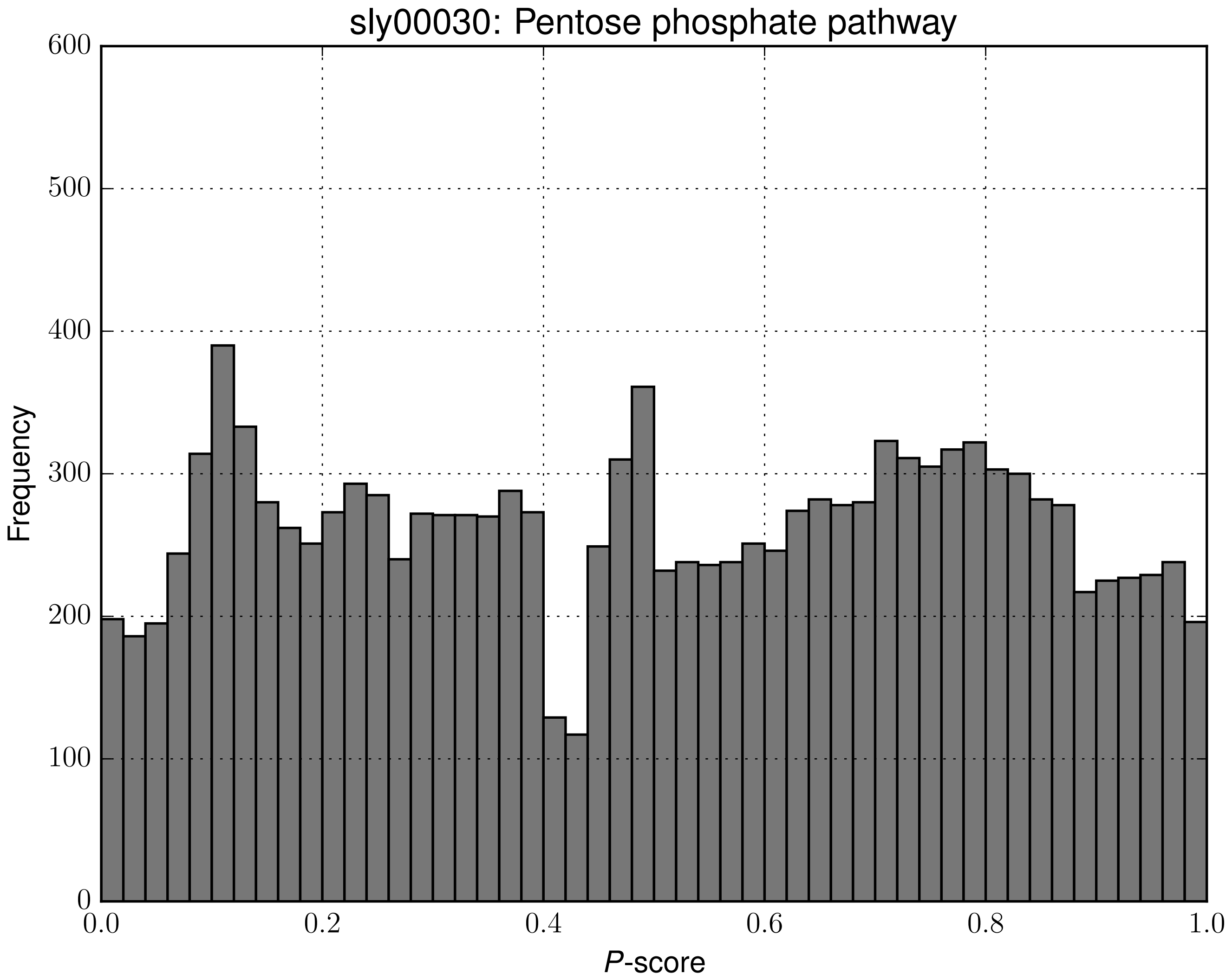

sly00030: Pentose phosphate pathway
600
500
400
300
200
100
0
Frequency
0.0
0.2
0.4
0.6
0.8
1.0
P-score

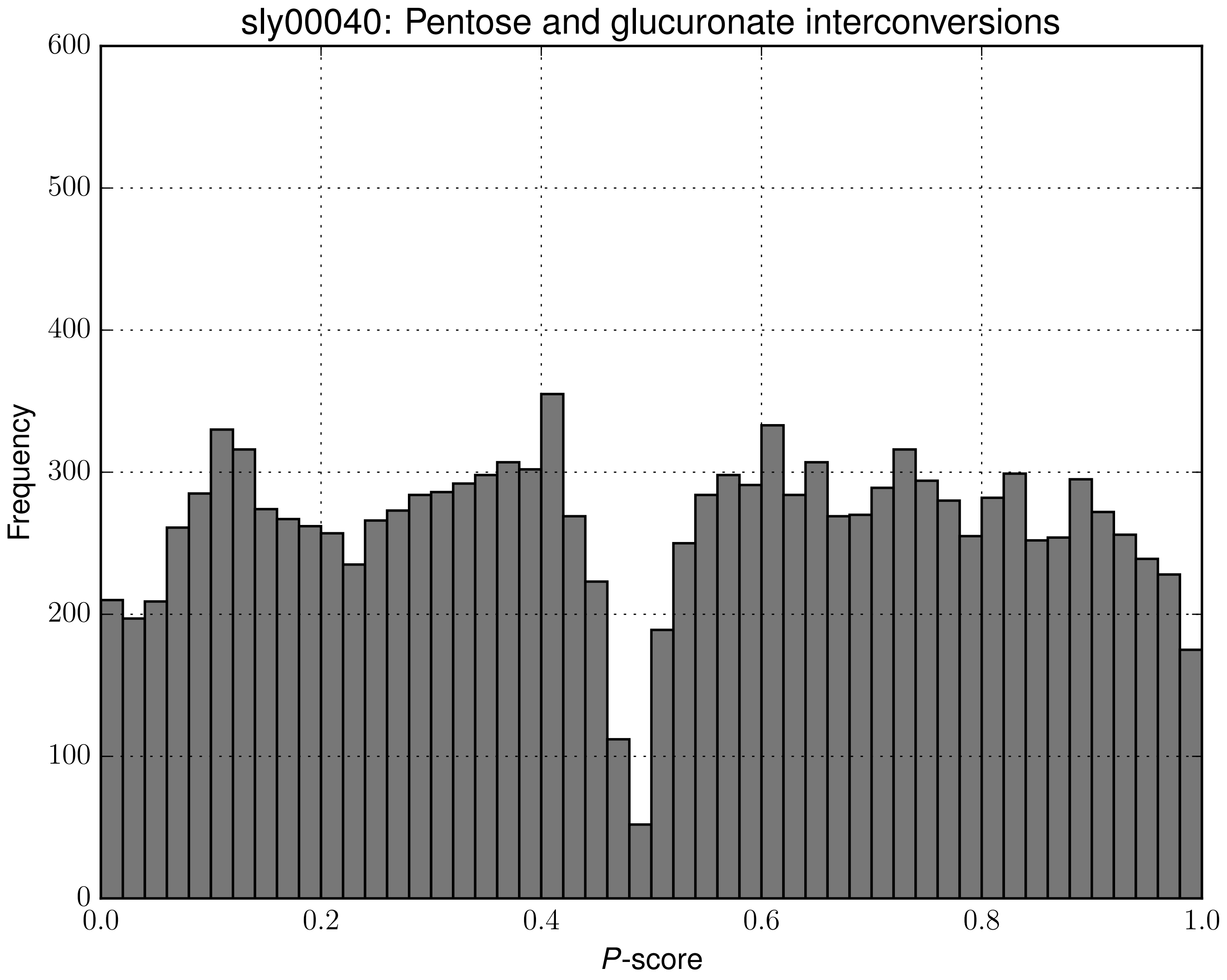
sly00040: Pentose and glucuronate interconversions
Frequency
P-score
0
100
200
300
400
500
600
0.0
0.2
0.4
0.6
0.8
1.0

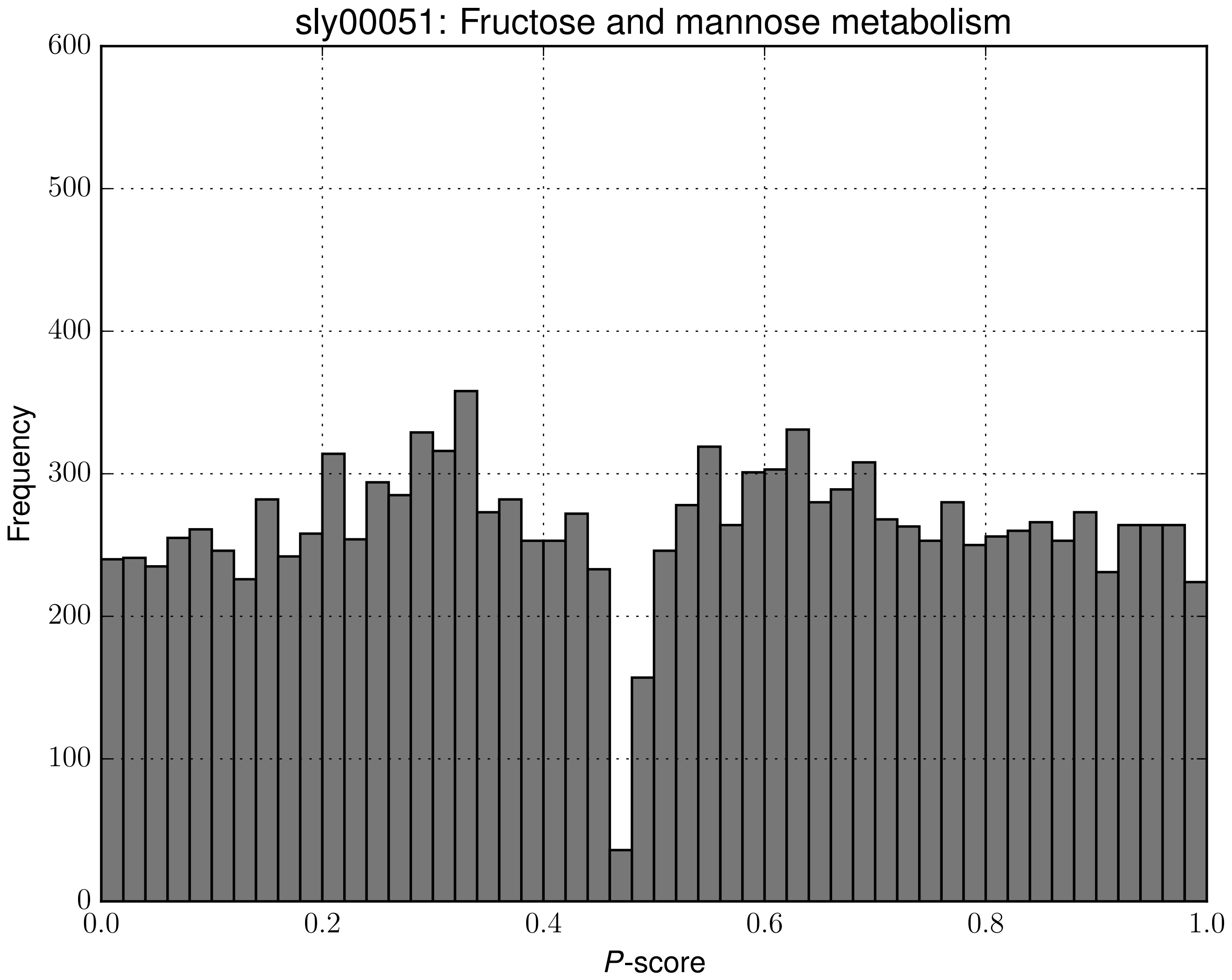
sly00051: Fructose and mannose metabolism
600
500
400
300
200
100
0
0.0
0.2
0.4
0.6
0.8
1.0
Frequency
P-score

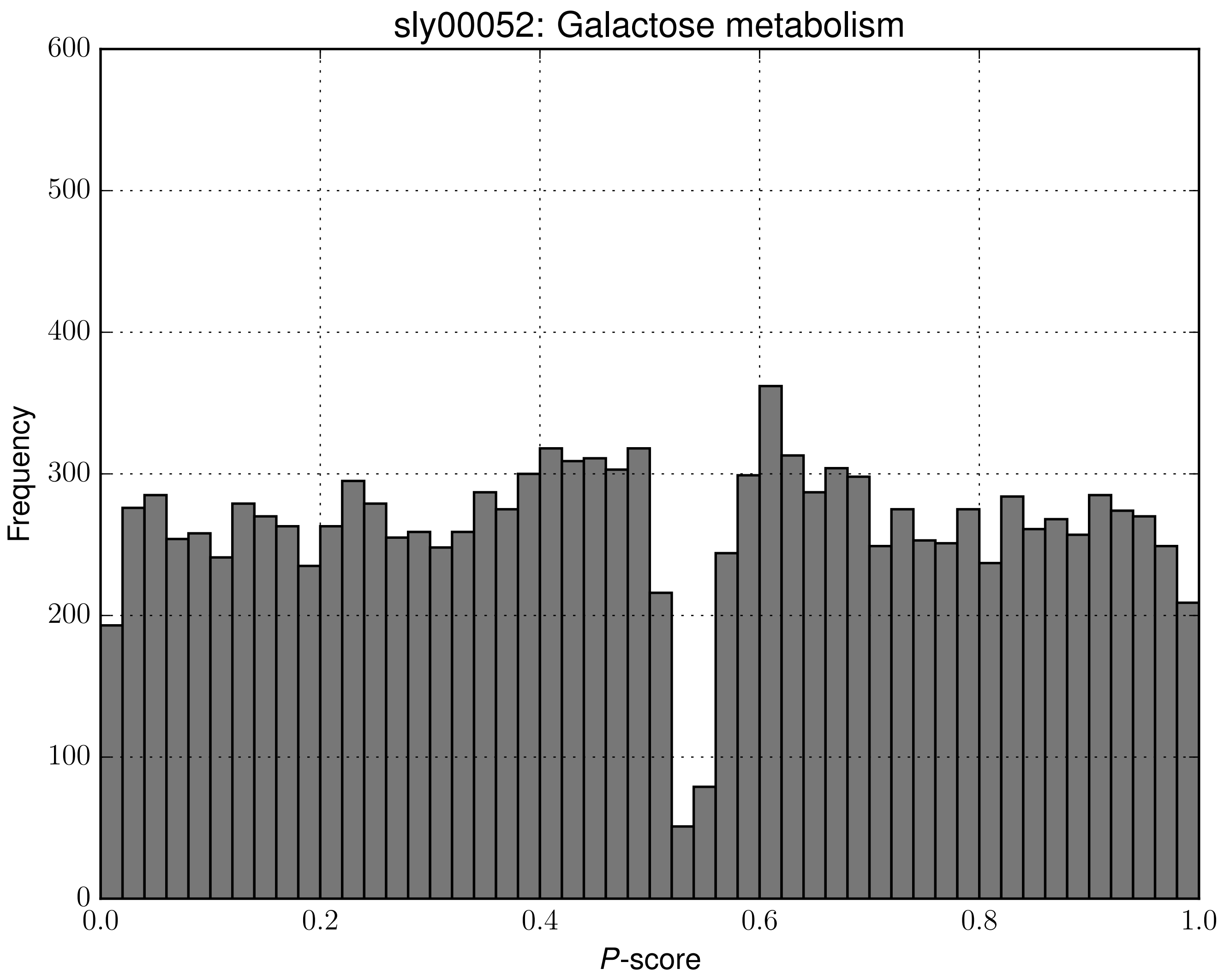
sly00052: Galactose metabolism
Frequency
P-score
0
100
200
300
400
500
600
0.0
0.2
0.4
0.6
0.8
1.0

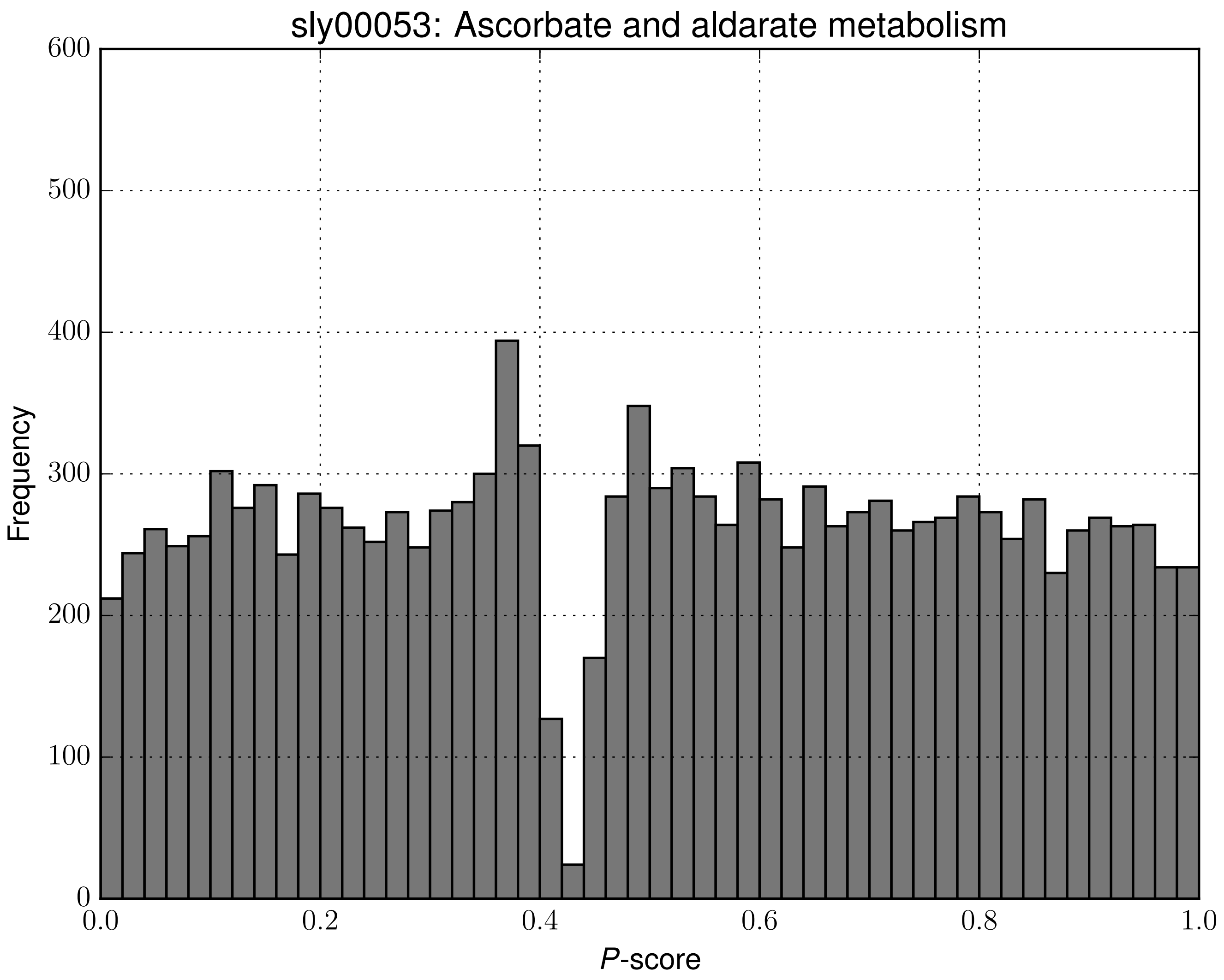
sly00053: Ascorbate and aldarate metabolism
Frequency
P-score
0
100
200
300
400
500
600
0.0
0.2
0.4
0.6
0.8
1.0

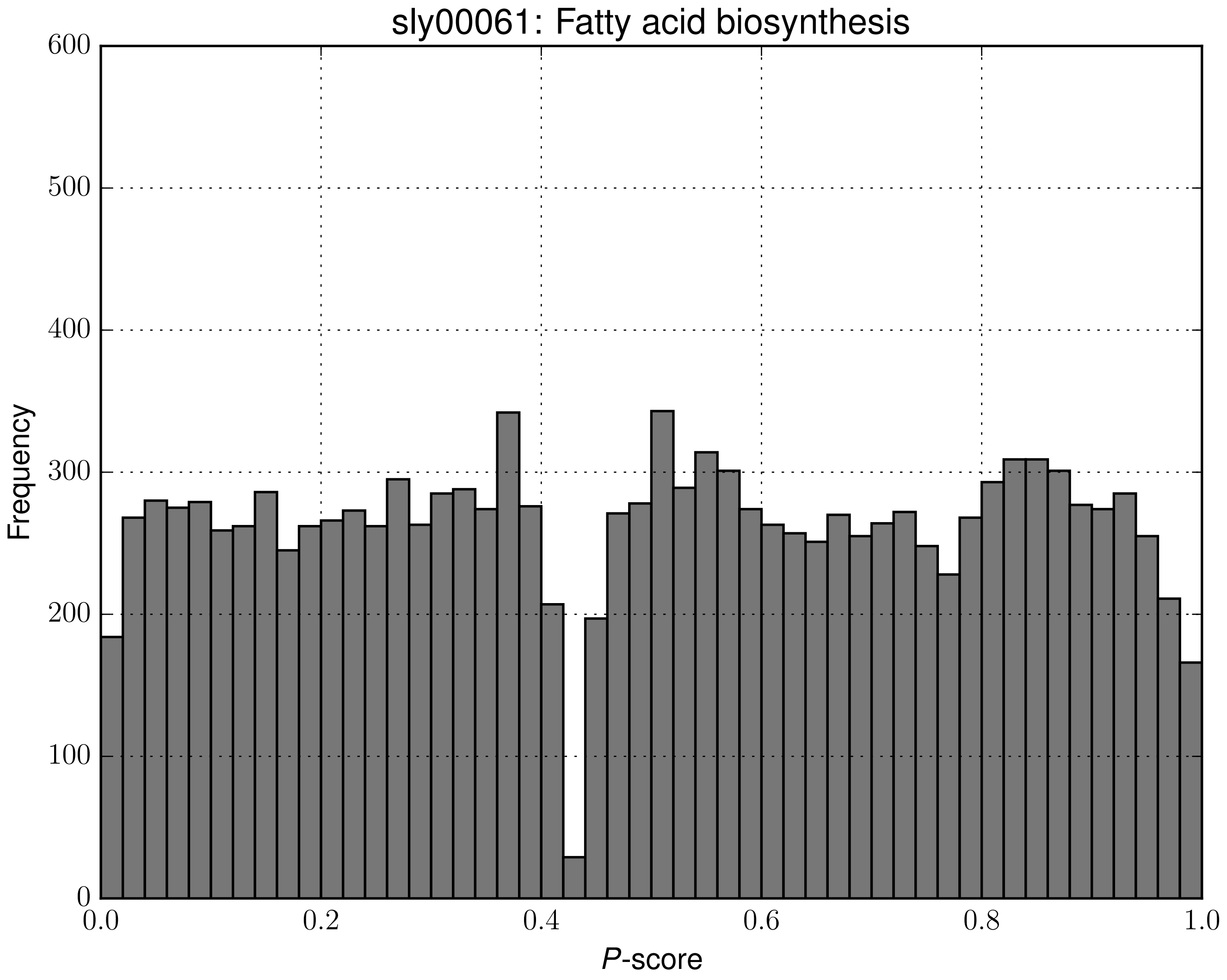
sly00061: Fatty acid biosynthesis
Frequency
P-score
0
100
200
300
400
500
600
0.0
0.2
0.4
0.6
0.8
1.0

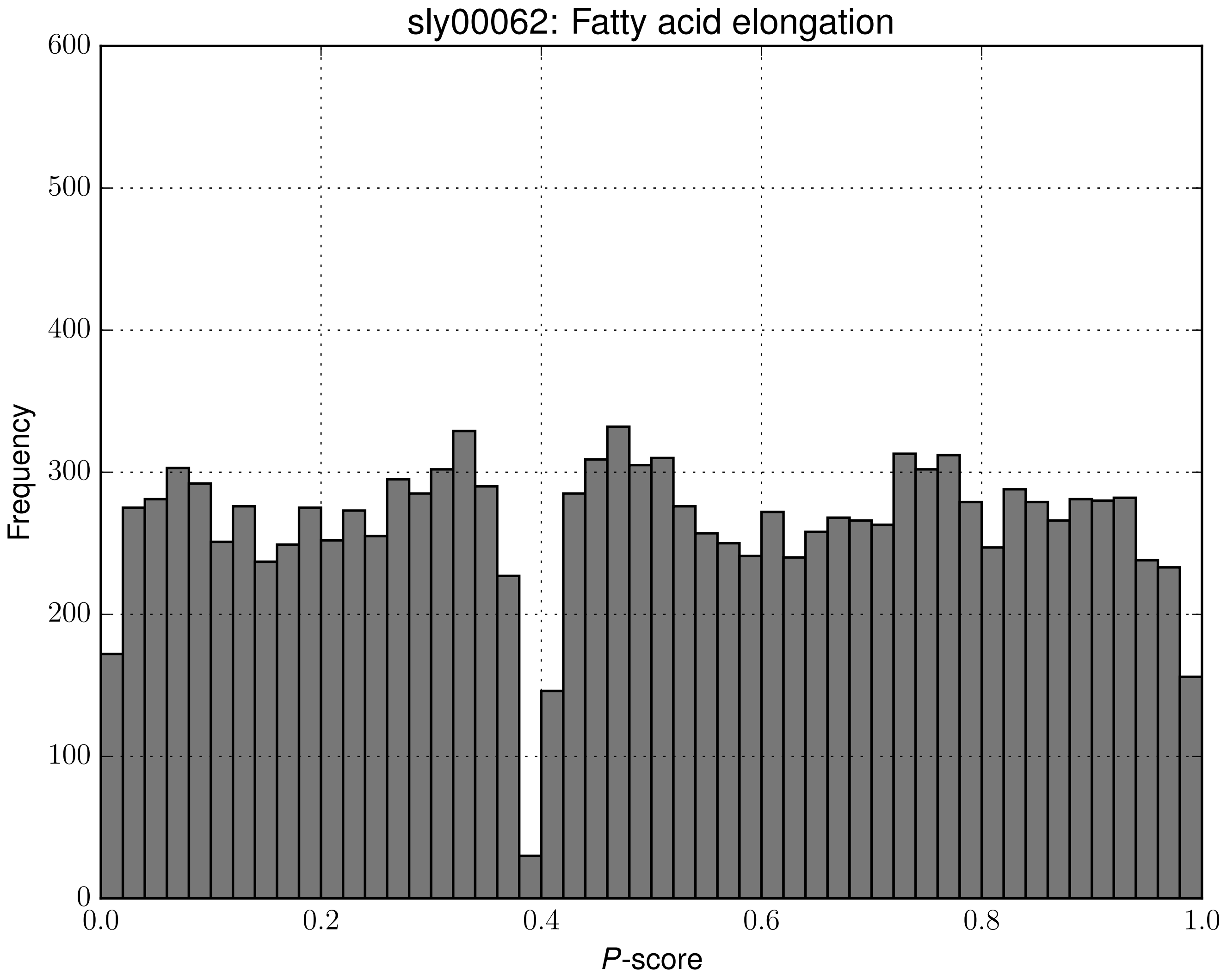

sly00062: Fatty acid elongation
600
500
400
300
200
100
0
Frequency
0.0
0.2
0.4
0.6
0.8
1.0
P-score

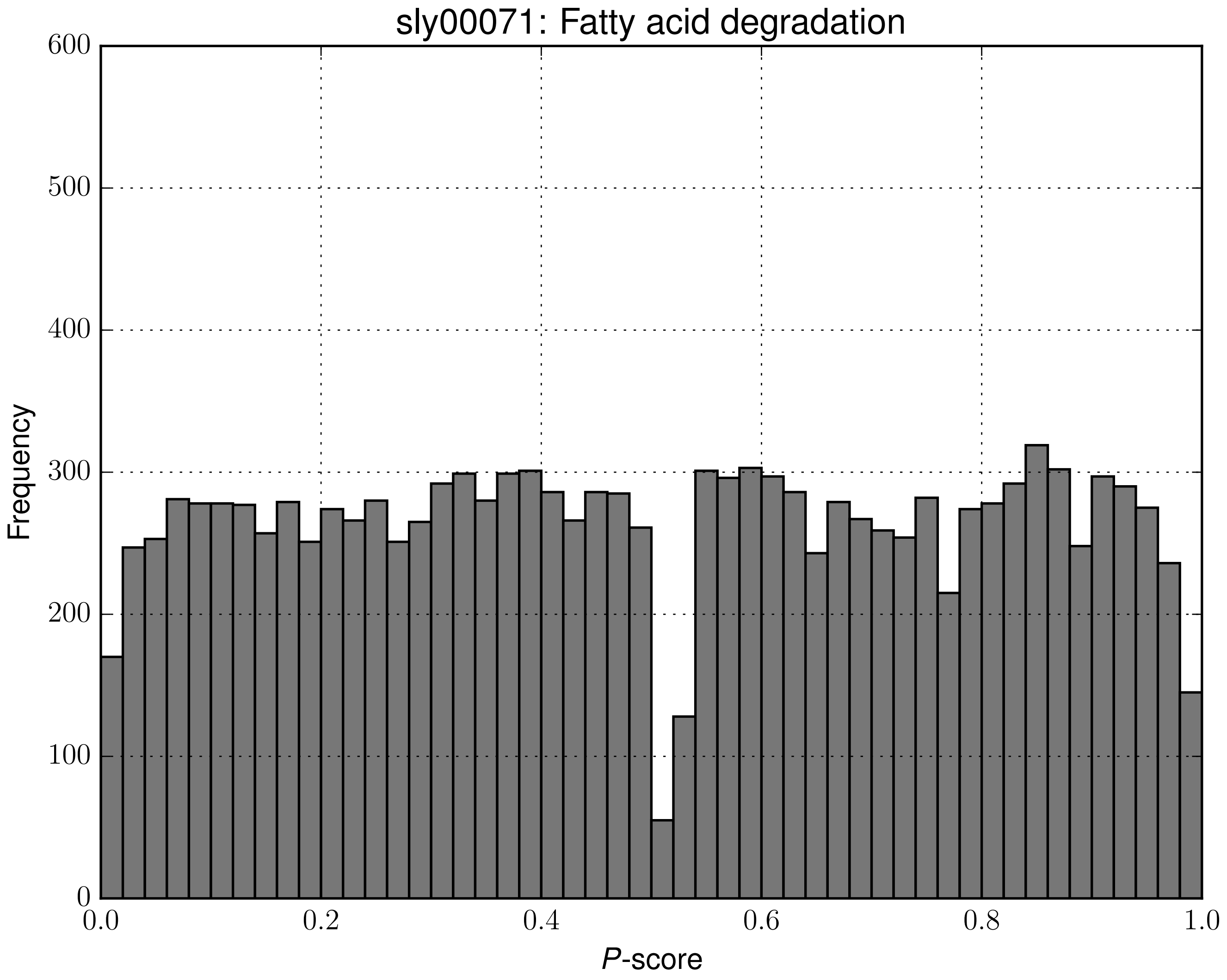

sly00071: Fatty acid degradation
Frequency
P-score
0
100
200
300
400
500
600
0.0
0.2
0.4
0.6
0.8
1.0

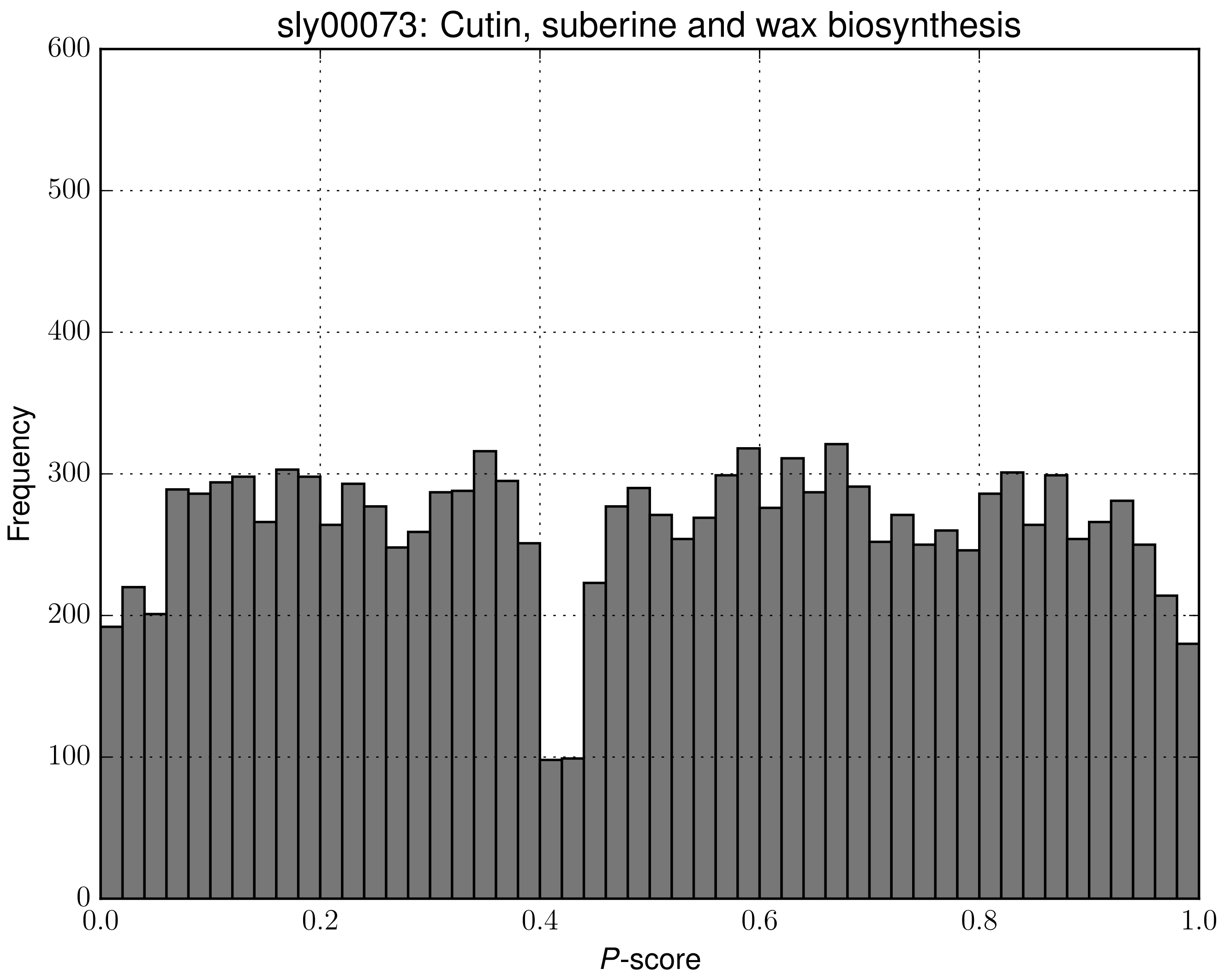
sly00073: Cutin, suberine and wax biosynthesis
Frequency
P-score
0
100
200
300
400
500
600
0.0
0.2
0.4
0.6
0.8
1.0

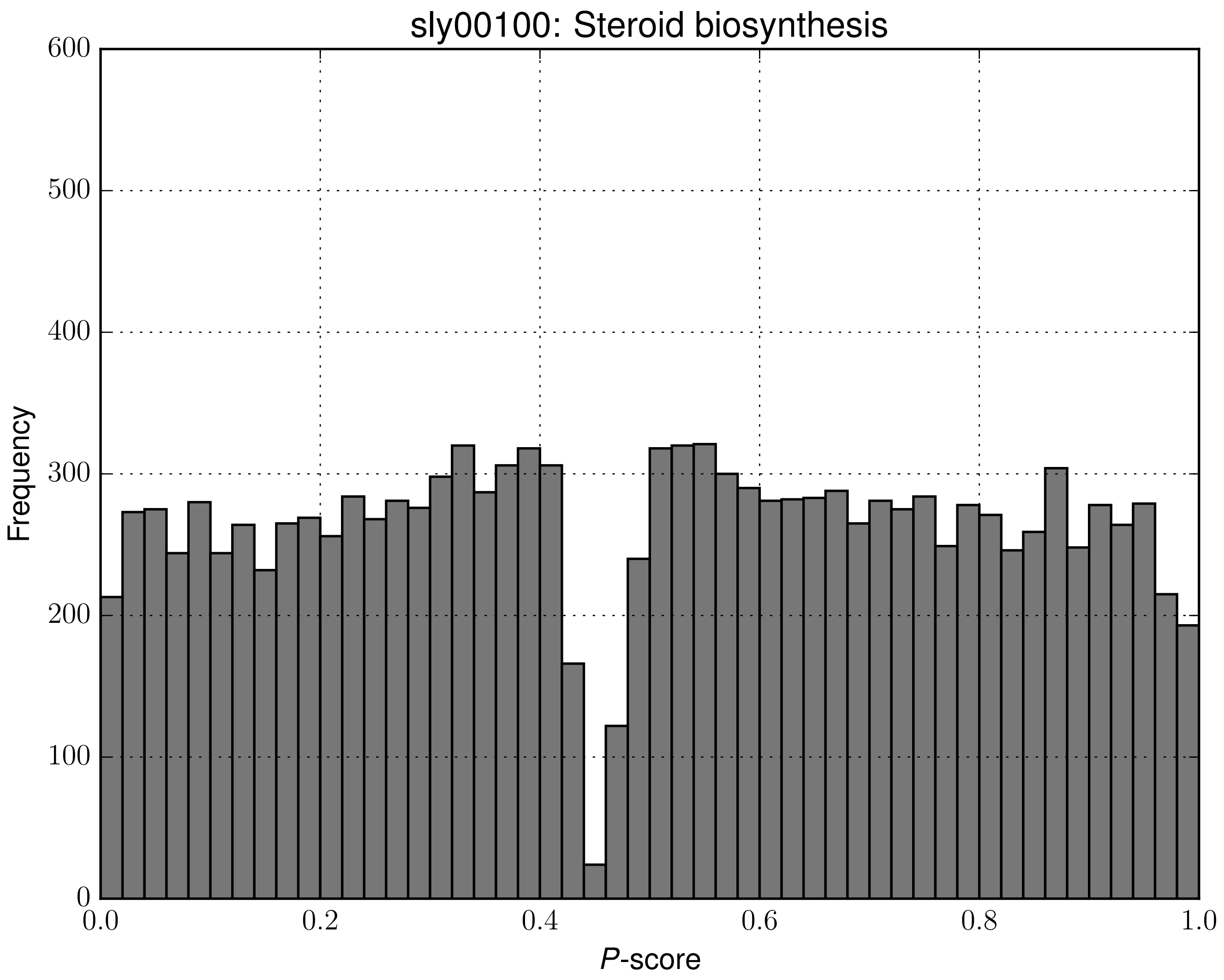
sly00100: Steroid biosynthesis
Frequency
P-score
0
100
200
300
400
500
600
0.0
0.2
0.4
0.6
0.8
1.0

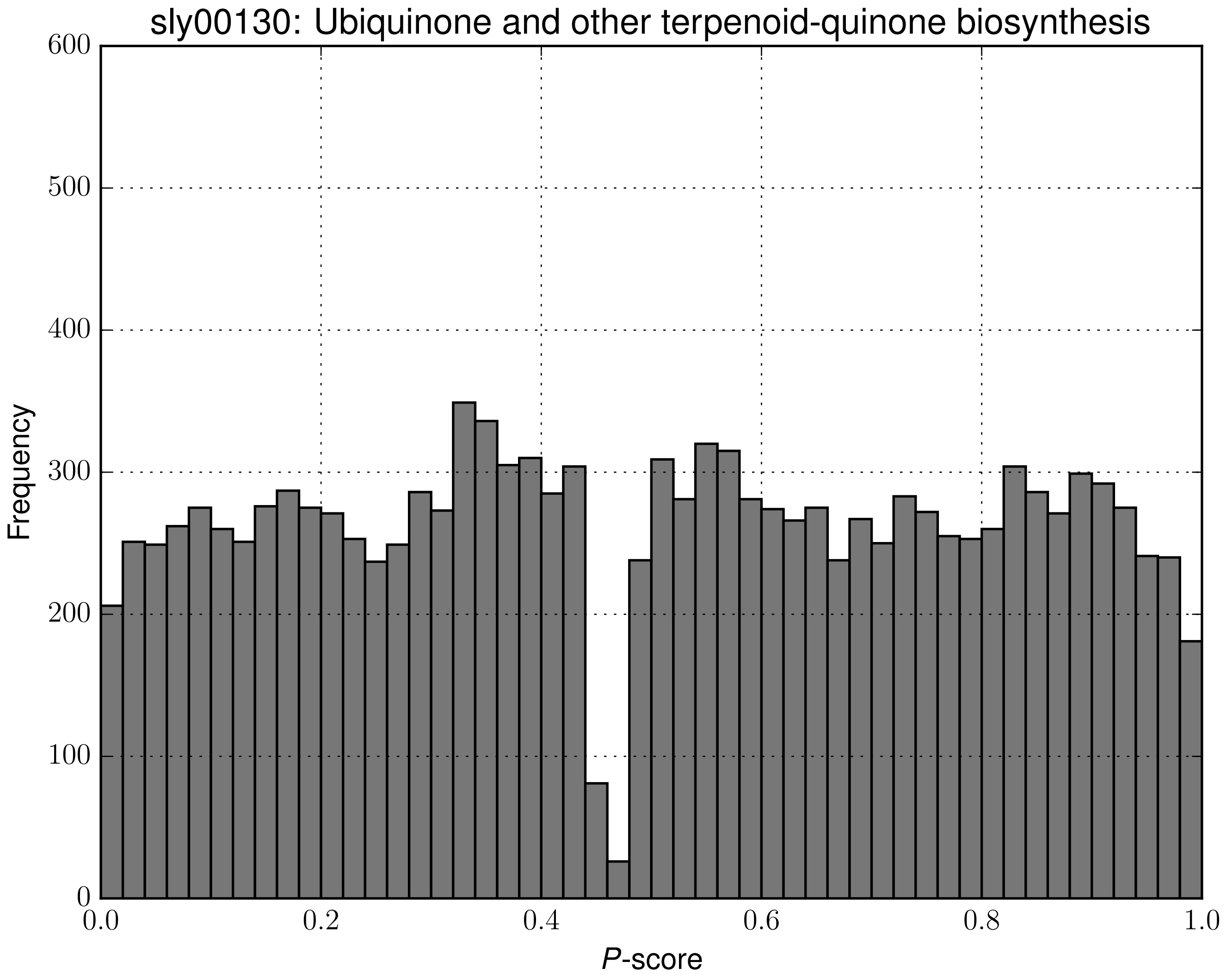
sly00130: Ubiquinone and other terpenoid-quinone biosynthesis
Frequency
P-score
0
100
200
300
400
500
600
0.0
0.2
0.4
0.6
0.8
1.0

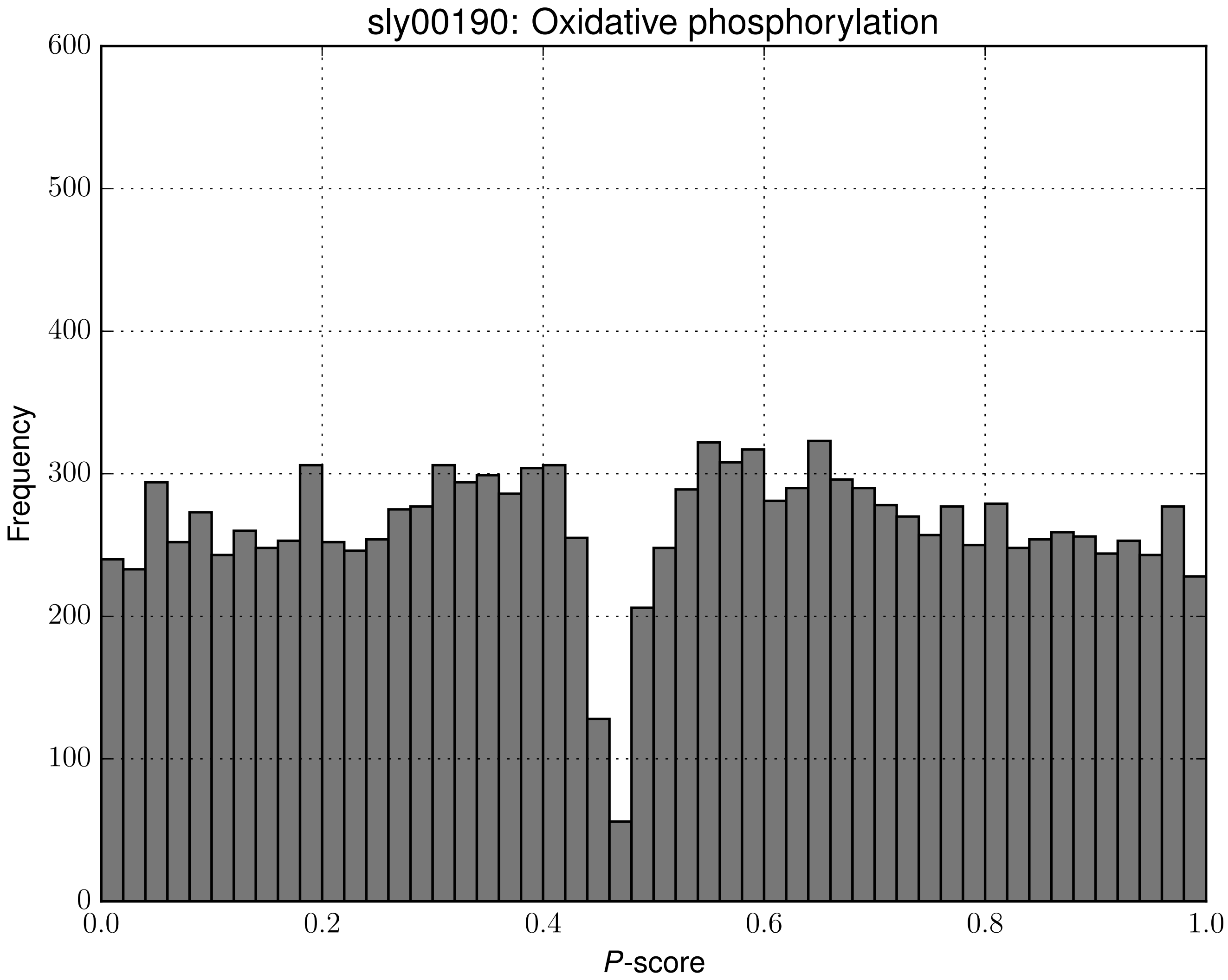
sly00190: Oxidative phosphorylation
Frequency
P-score
0
100
200
300
400
500
600
0.0
0.2
0.4
0.6
0.8
1.0

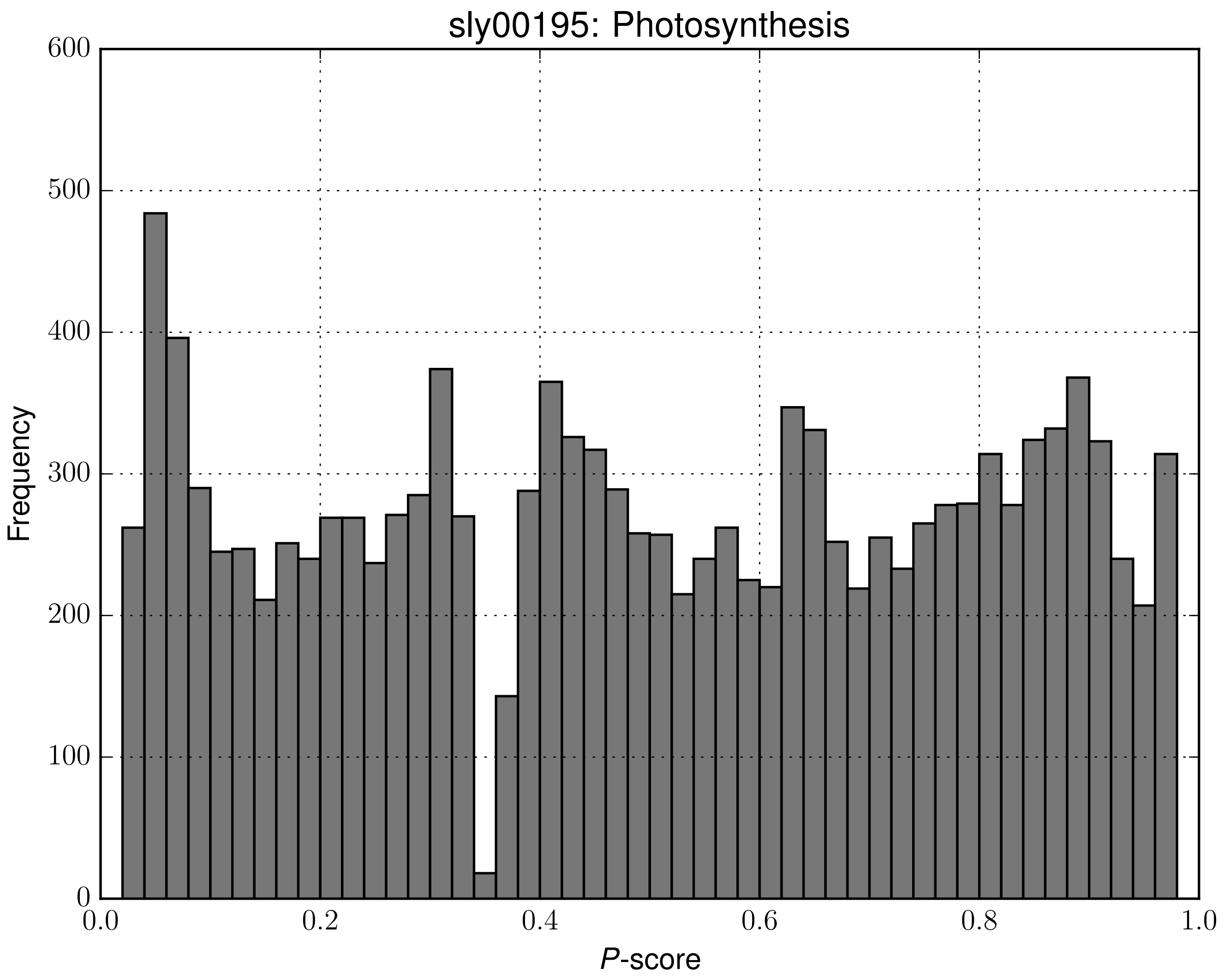
sly00195: Photosynthesis
Frequency
P-score
0
100
200
300
400
500
600
0.0
0.2
0.4
0.6
0.8
1.0

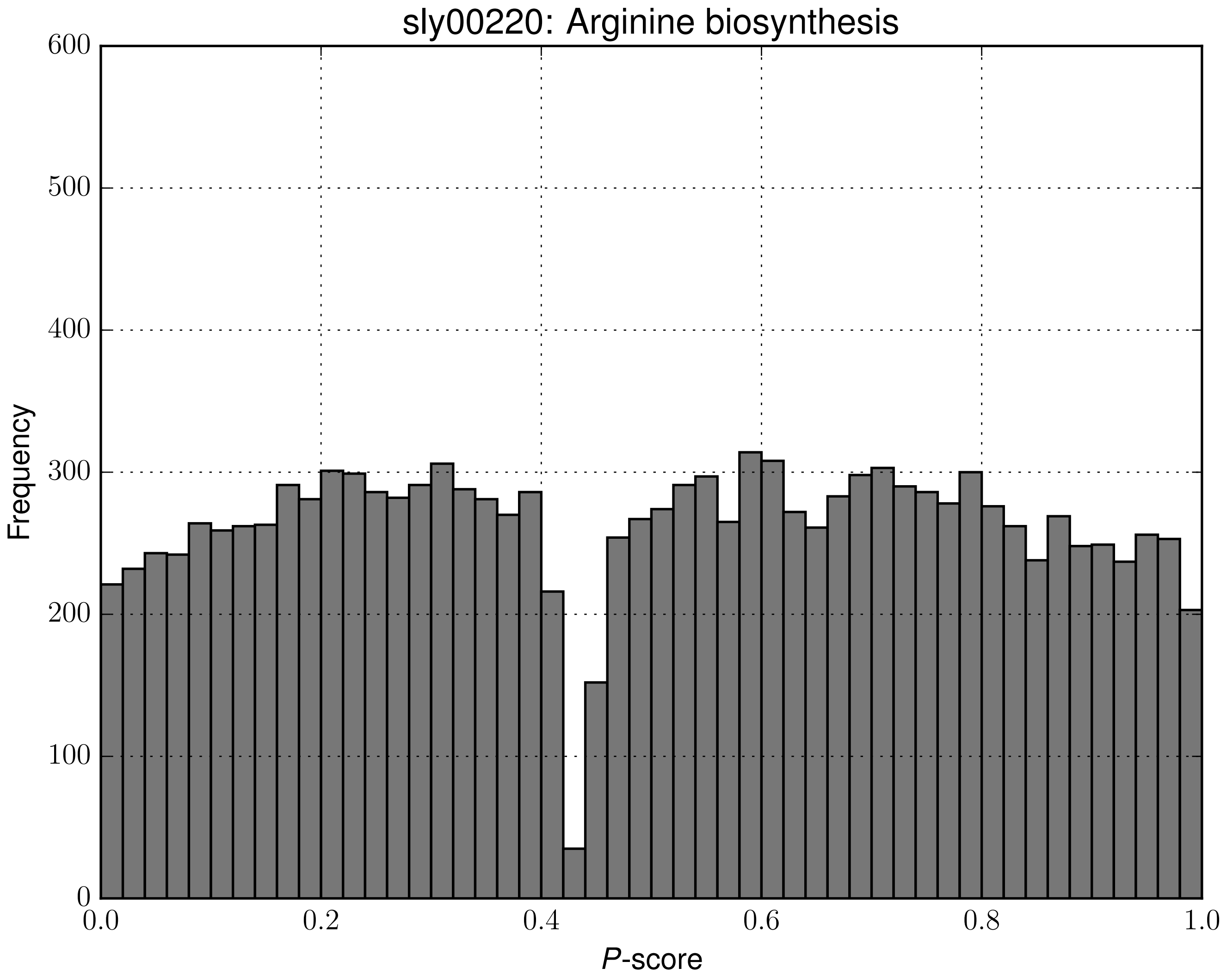

sly00220: Arginine biosynthesis
Frequency
P-score
0
100
200
300
400
500
600
0.0
0.2
0.4
0.6
0.8
1.0

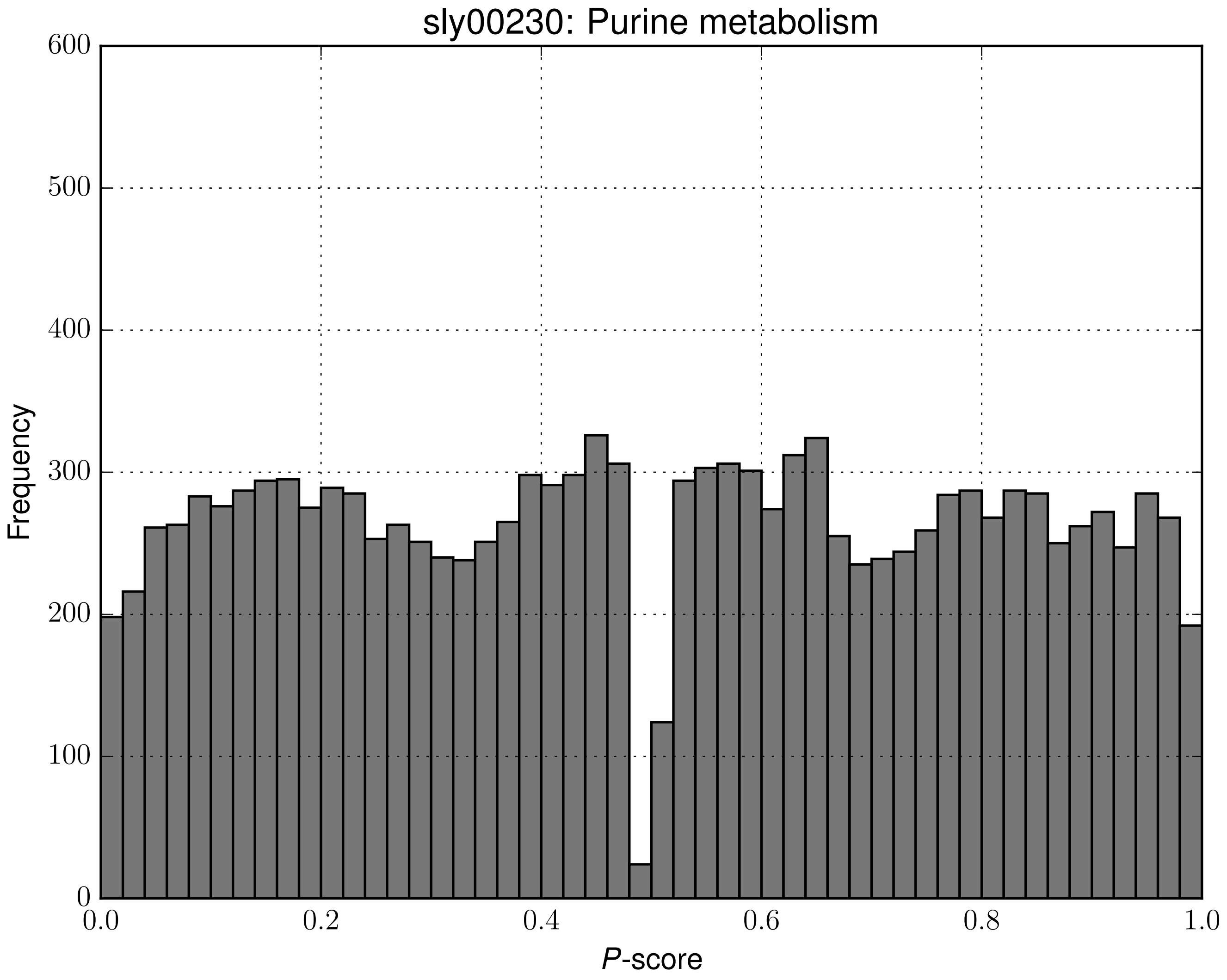
sly00230: Purine metabolism
Frequency
P-score
0
100
200
300
400
500
600
0.0
0.2
0.4
0.6
0.8
1.0

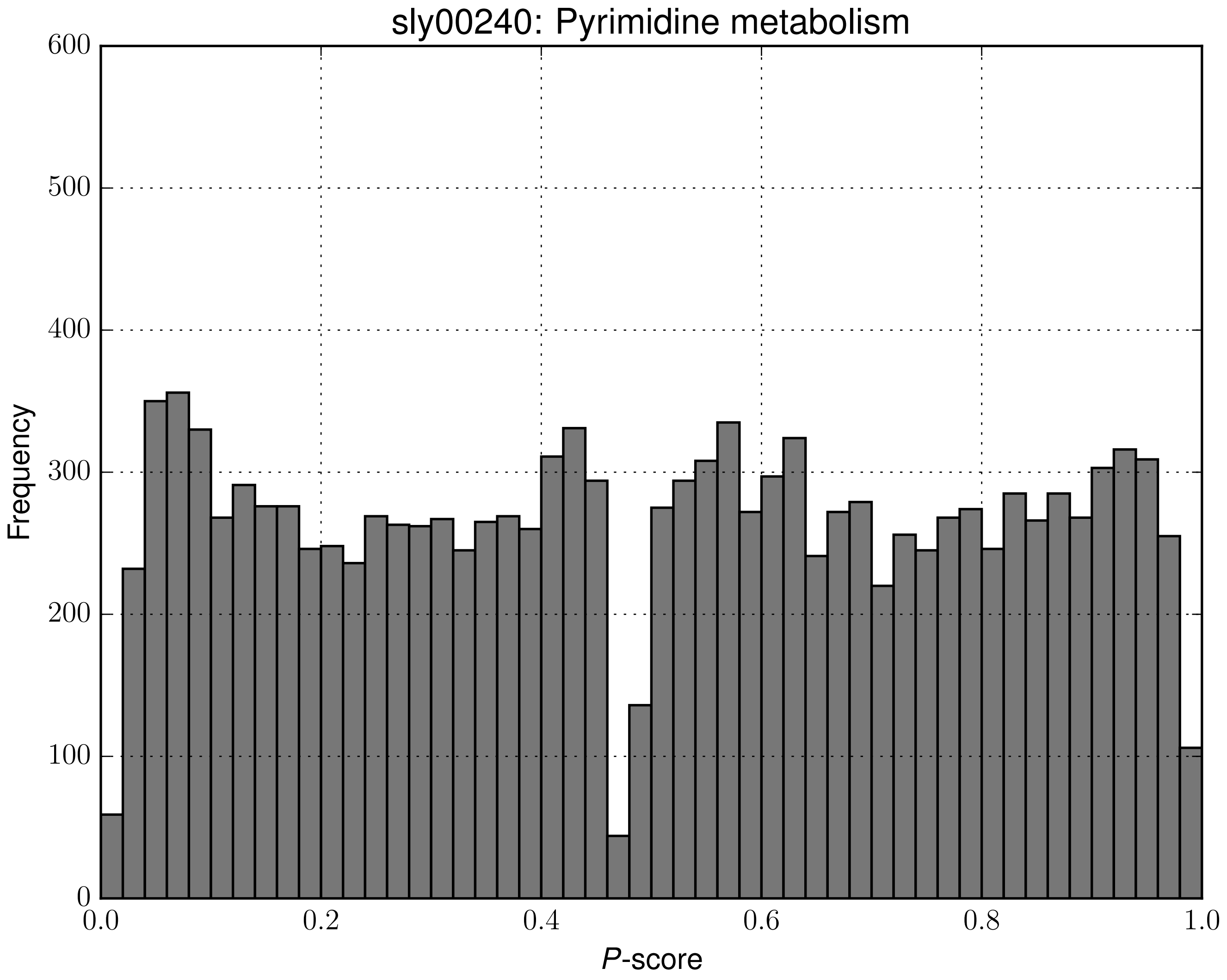
sly00240: Pyrimidine metabolism
Frequency
P-score
0
100
200
300
400
500
600
0.0
0.2
0.4
0.6
0.8
1.0

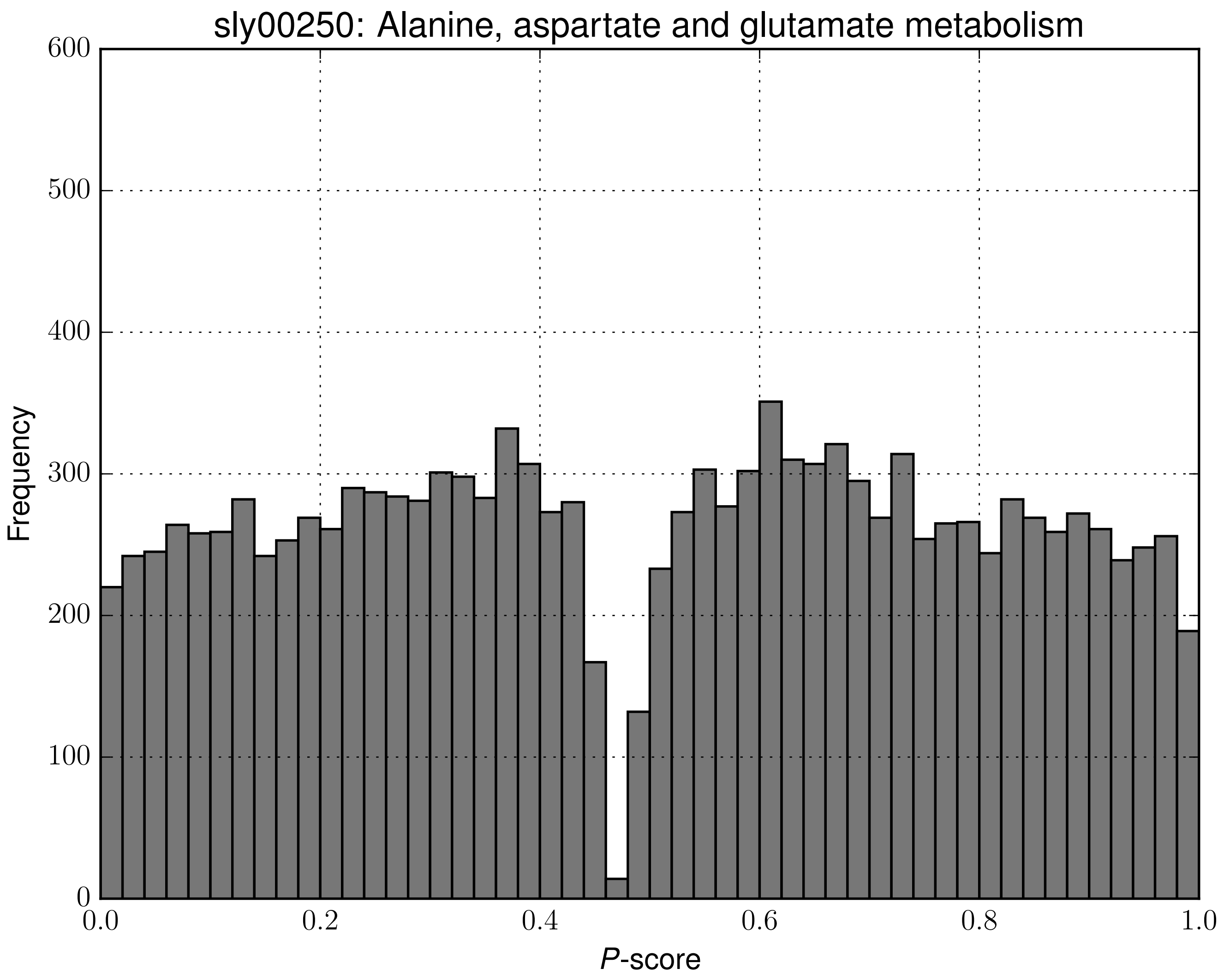
sly00250: Alanine, aspartate and glutamate metabolism
600
500
400
300
200
100
0
Frequency
0.0
0.2
0.4
0.6
0.8
1.0
P-score

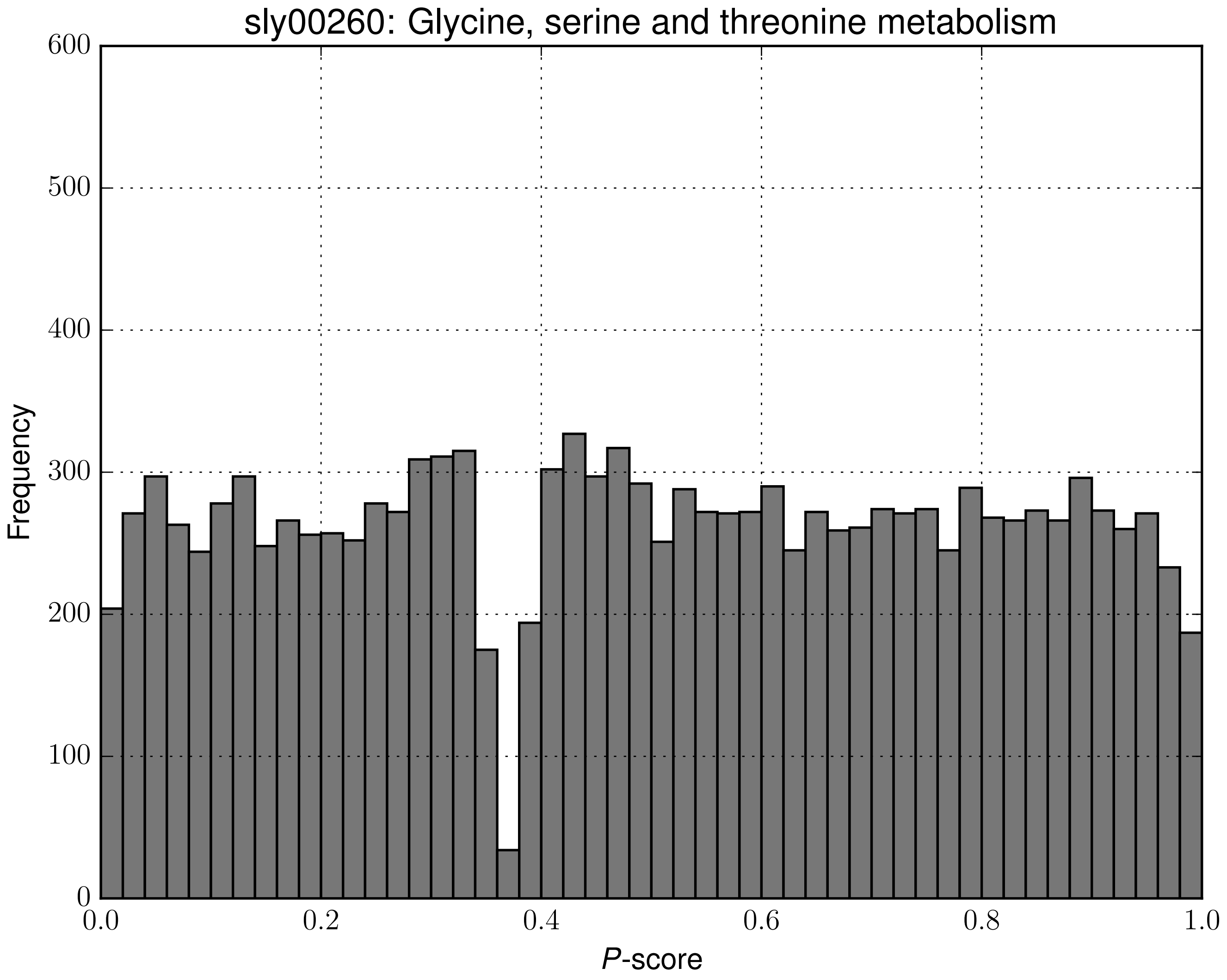
sly00260: Glycine, serine and threonine metabolism
Frequency
P-score
0
100
200
300
400
500
600
0.0
0.2
0.4
0.6
0.8
1.0

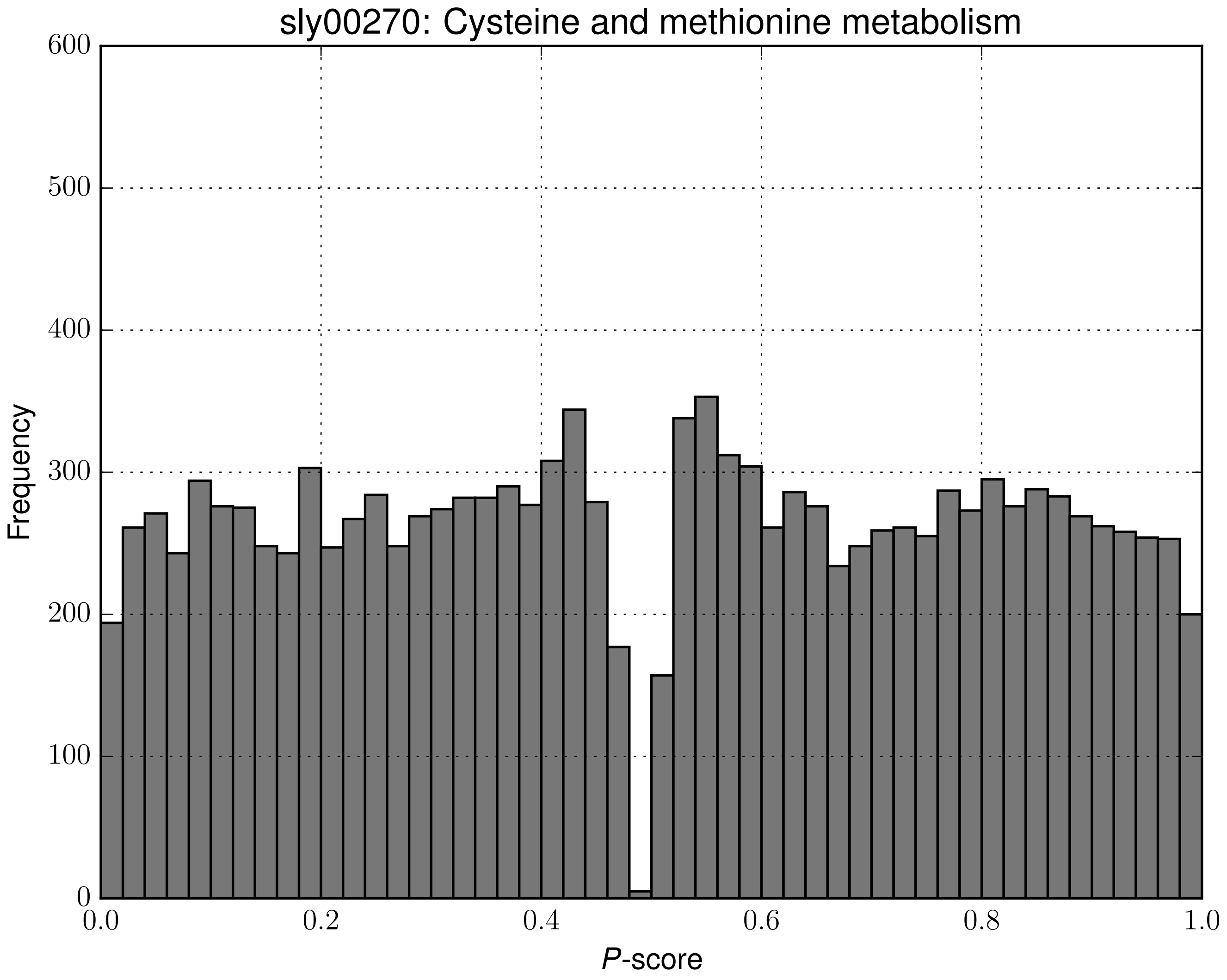
sly00270: Cysteine and methionine metabolism
Frequency
P-score
0
100
200
300
400
500
600
0.0
0.2
0.4
0.6
0.8
1.0

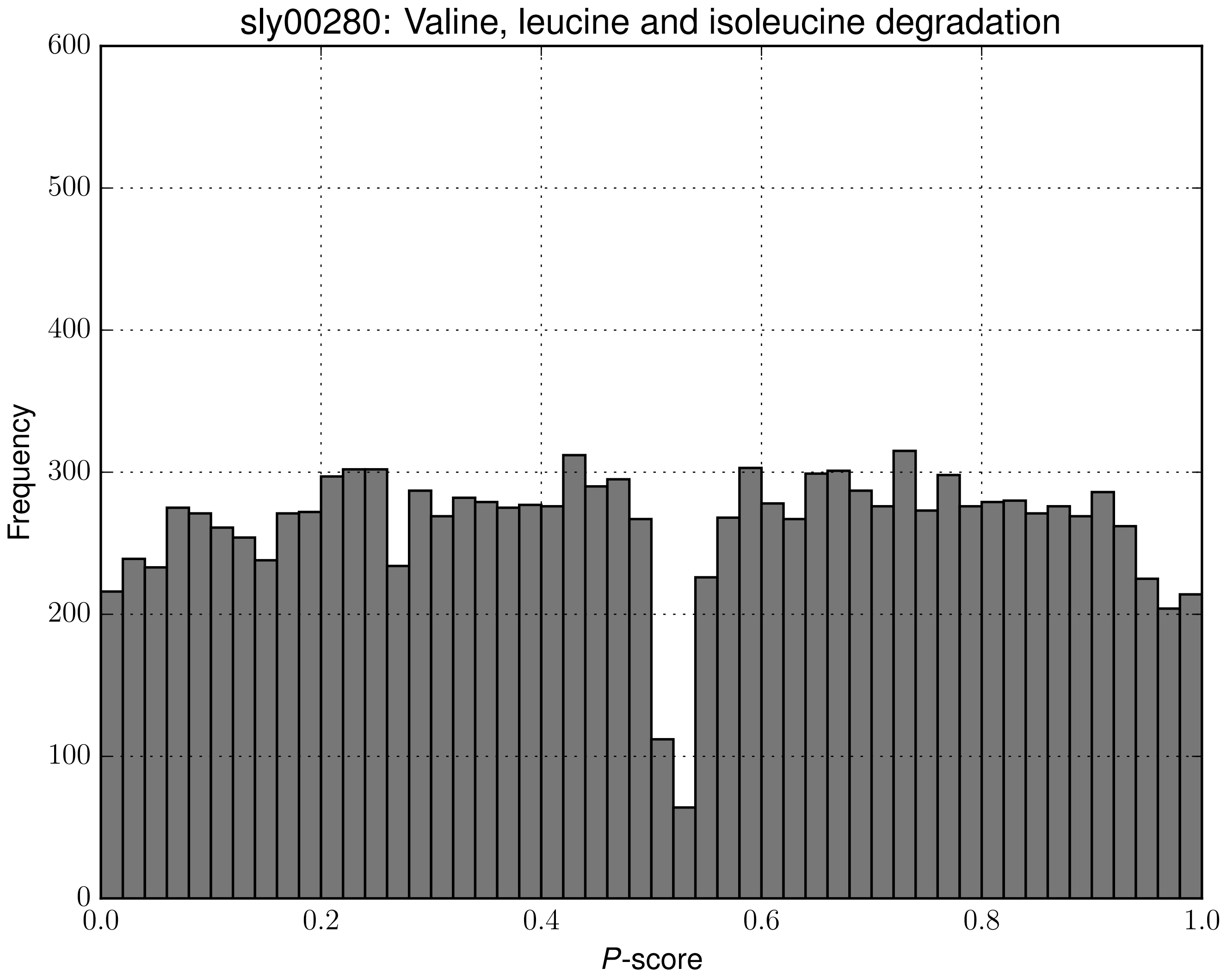
sly00280: Valine, leucine and isoleucine degradation
Frequency
P-score
0
100
200
300
400
500
600
0.0
0.2
0.4
0.6
0.8
1.0

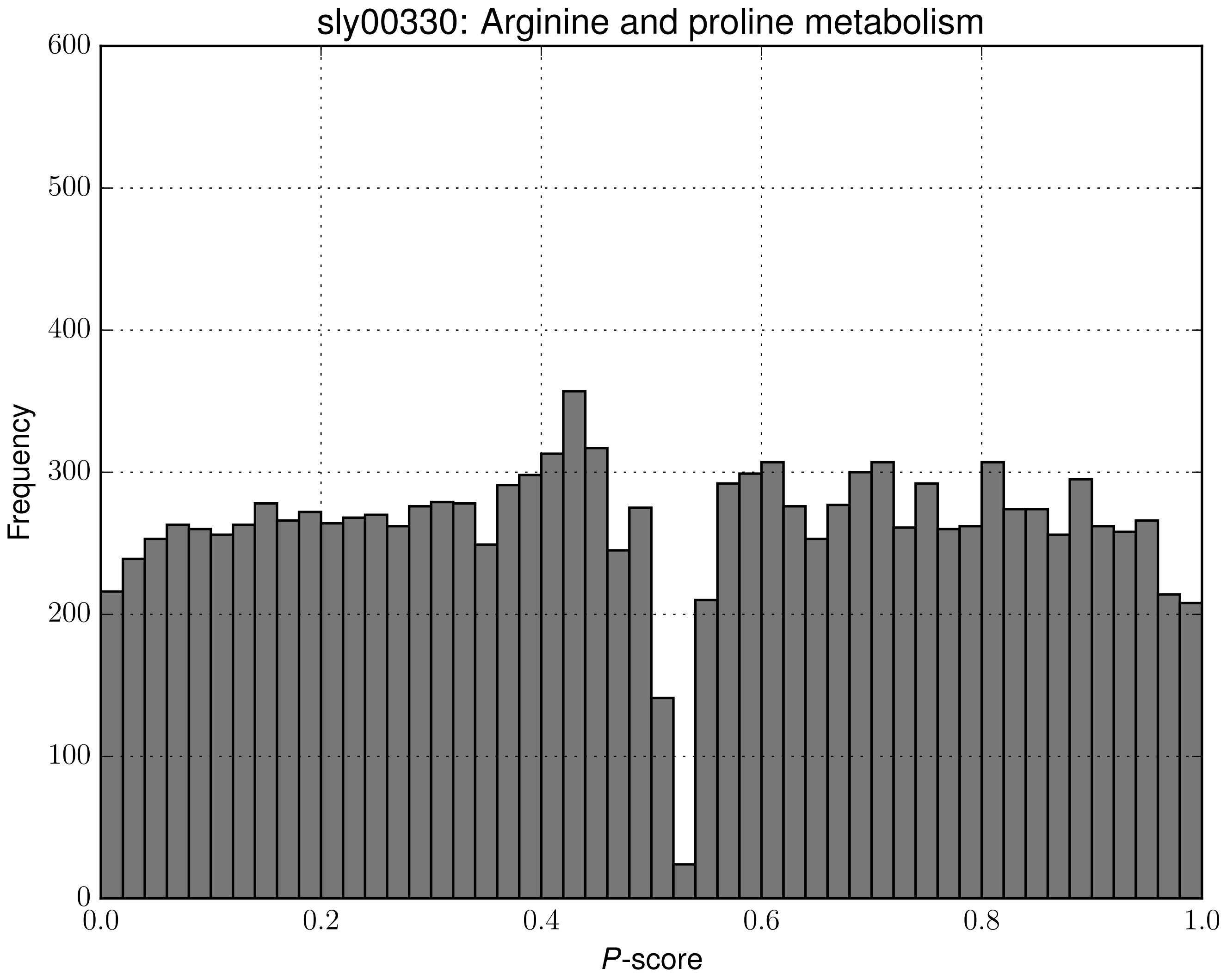
sly00330: Arginine and proline metabolism
Frequency
P-score
0
100
200
300
400
500
600
0.0
0.2
0.4
0.6
0.8
1.0

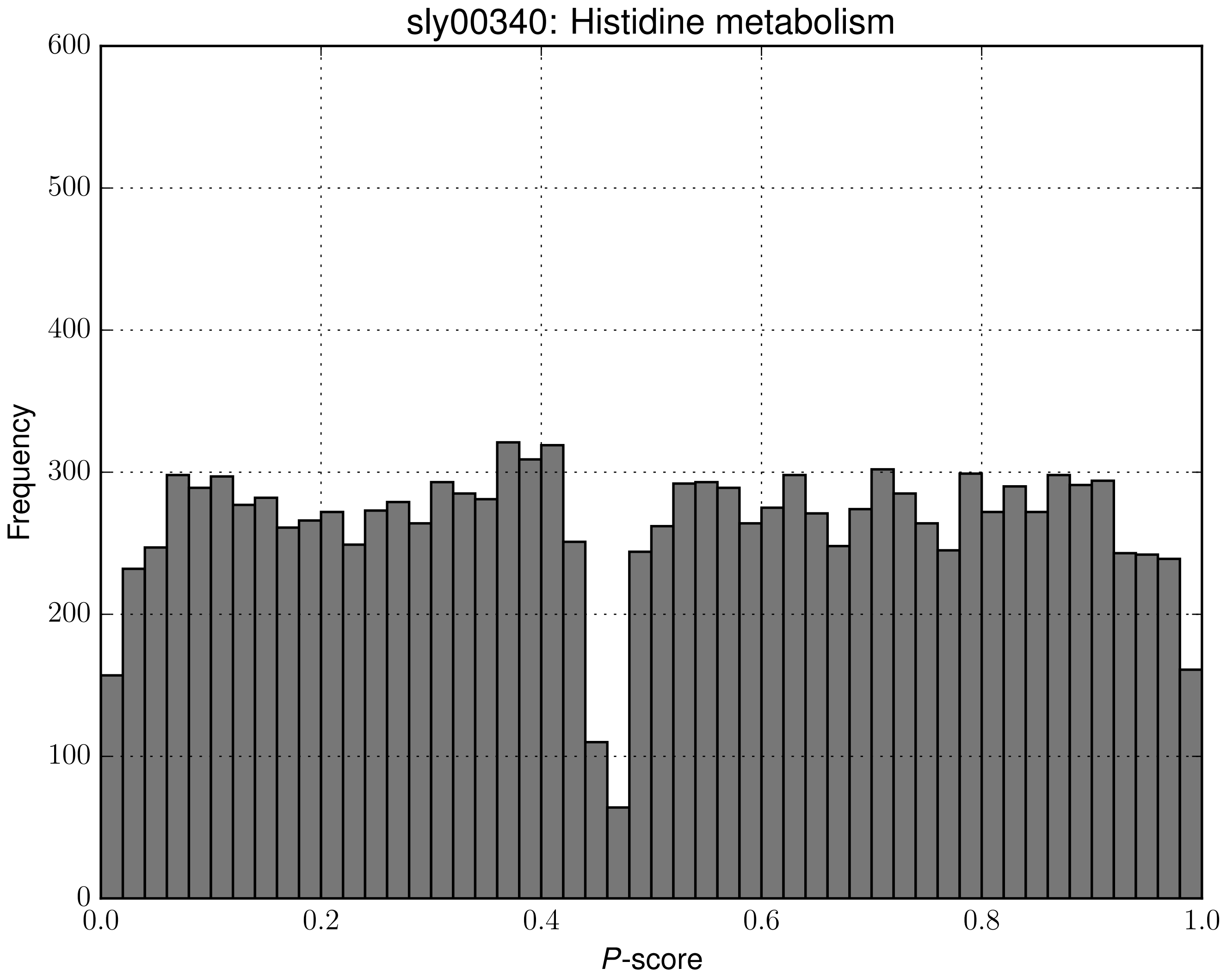
sly00340: Histidine metabolism
Frequency
P-score
0
100
200
300
400
500
600
0.0
0.2
0.4
0.6
0.8
1.0

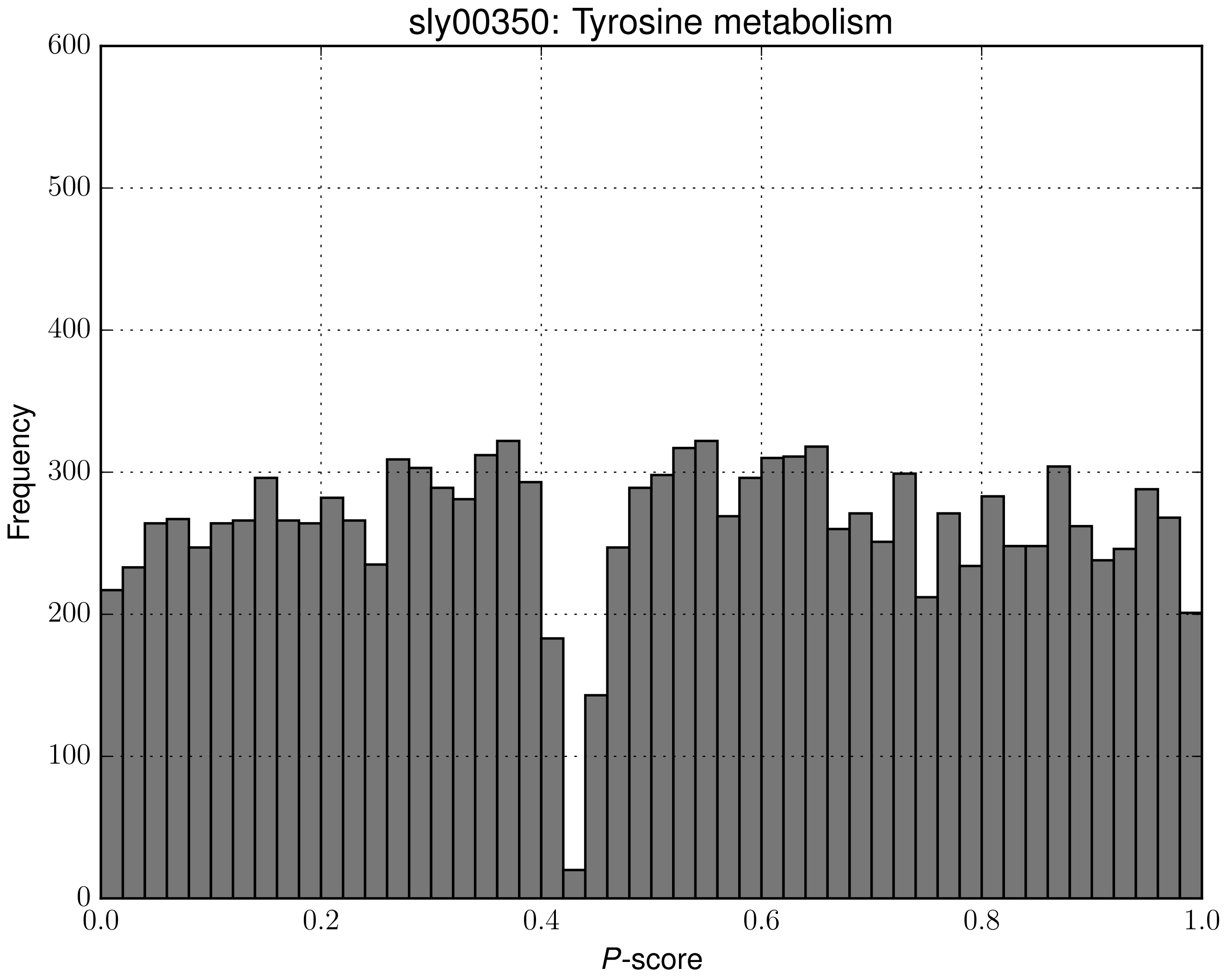
sly00350: Tyrosine metabolism
Frequency
P-score
0
100
200
300
400
500
600
0.0
0.2
0.4
0.6
0.8
1.0

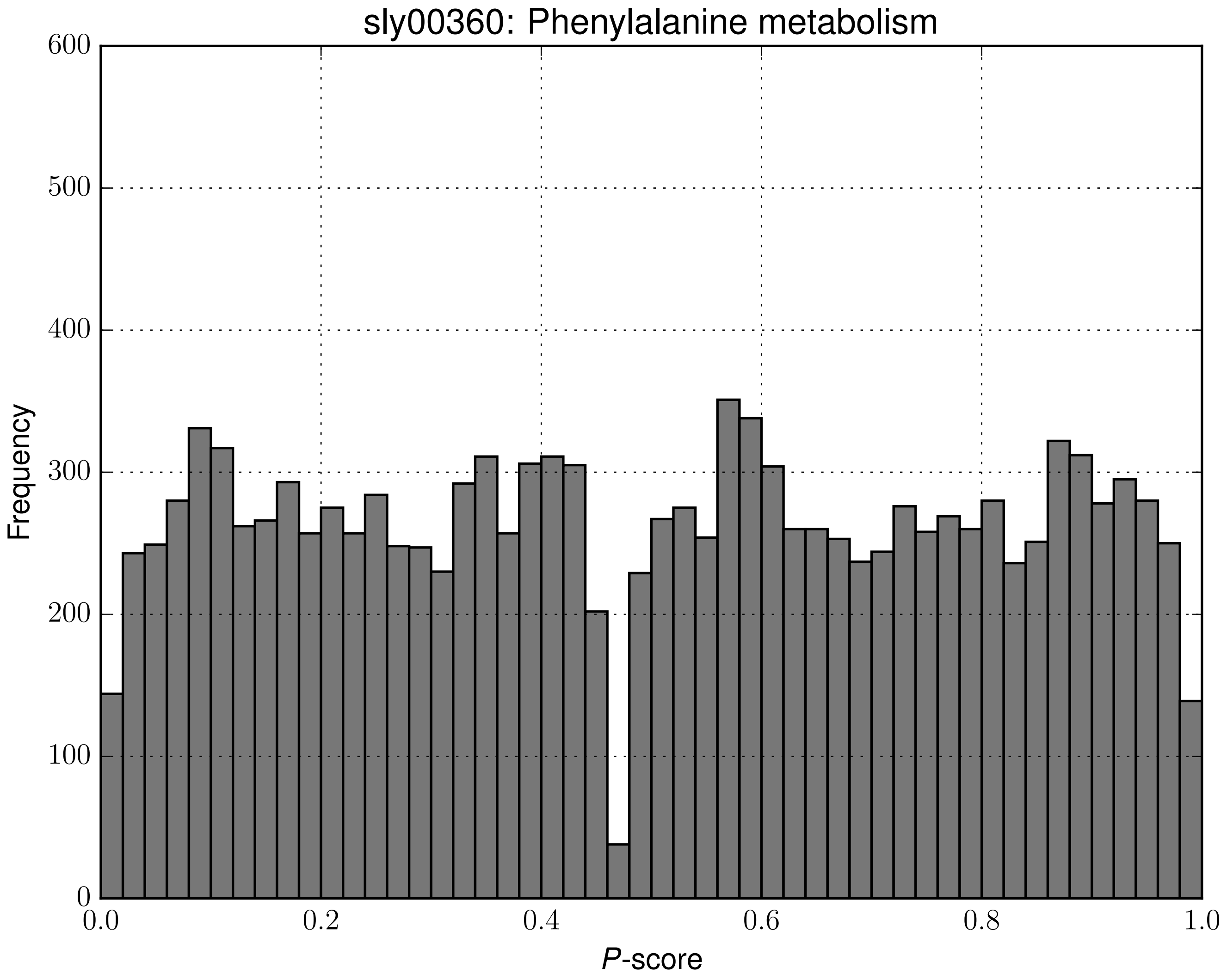

sly00360: Phenylalanine metabolism
Frequency
P-score
0
100
200
300
400
500
600
0.0
0.2
0.4
0.6
0.8
1.0

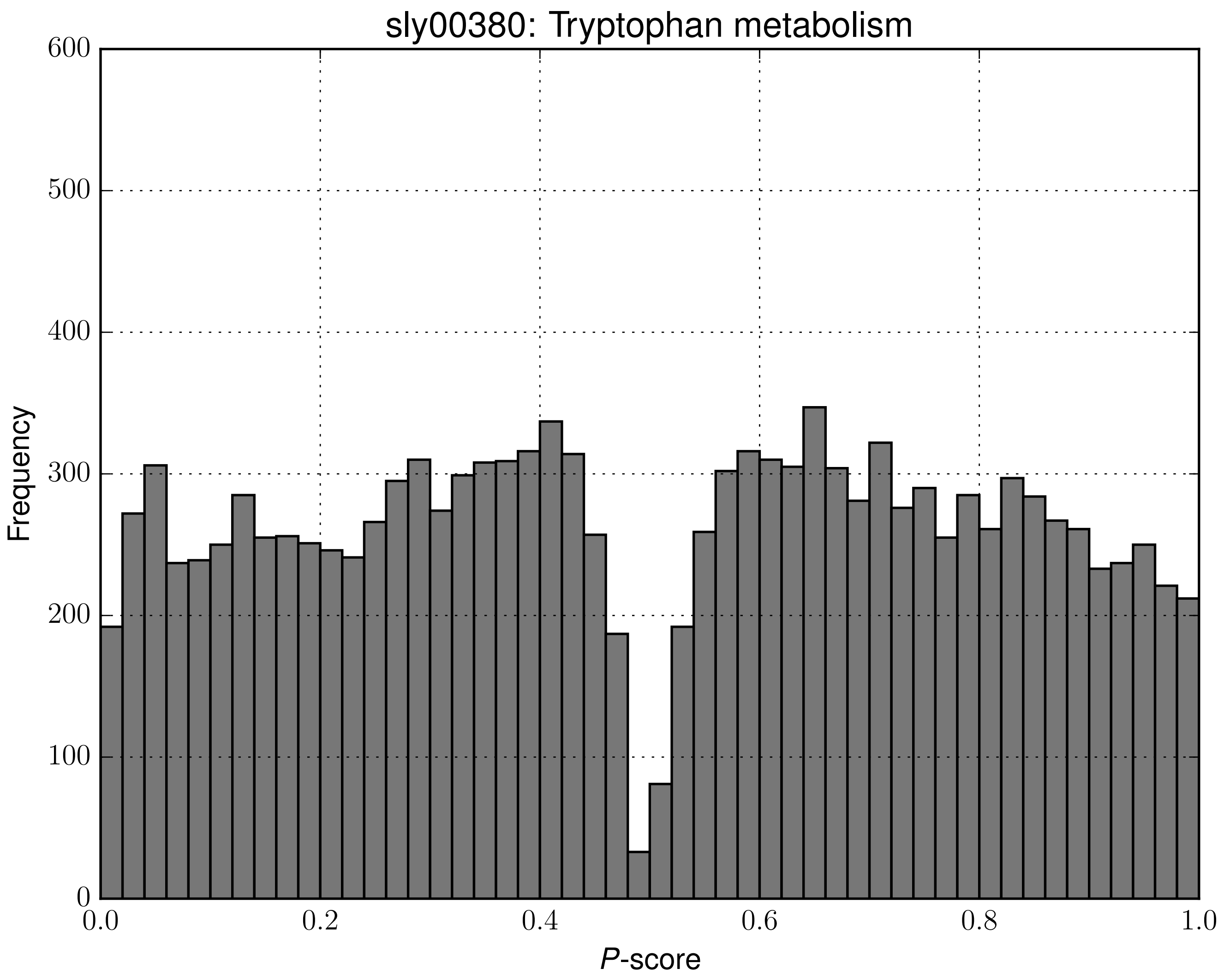

sly00380: Tryptophan metabolism
Frequency
P-score
0
100
200
300
400
500
600
0.0
0.2
0.4
0.6
0.8
1.0

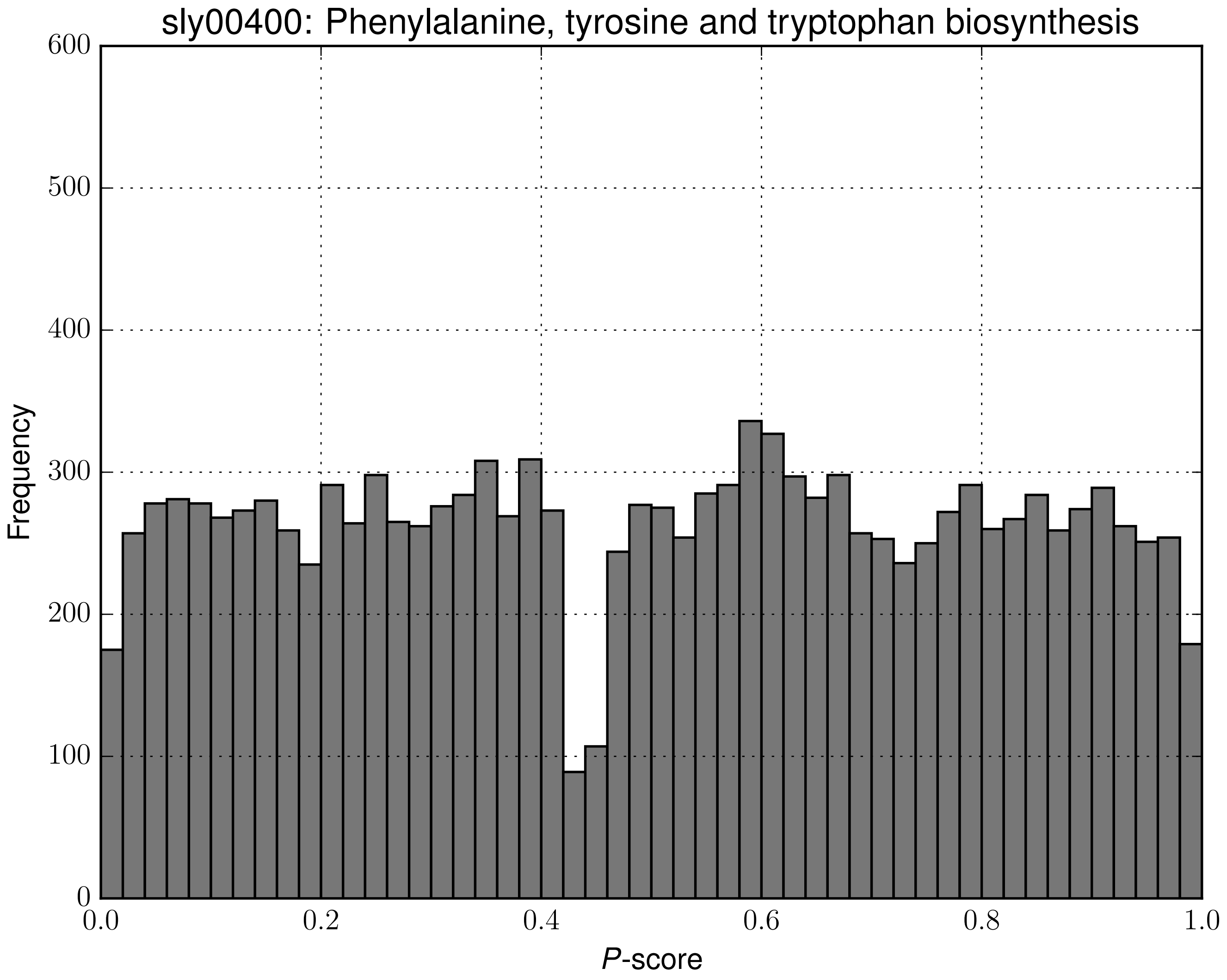
sly00400: Phenylalanine, tyrosine and tryptophan biosynthesis
Frequency
P-score
0
100
200
300
400
500
600
0.0
0.2
0.4
0.6
0.8
1.0

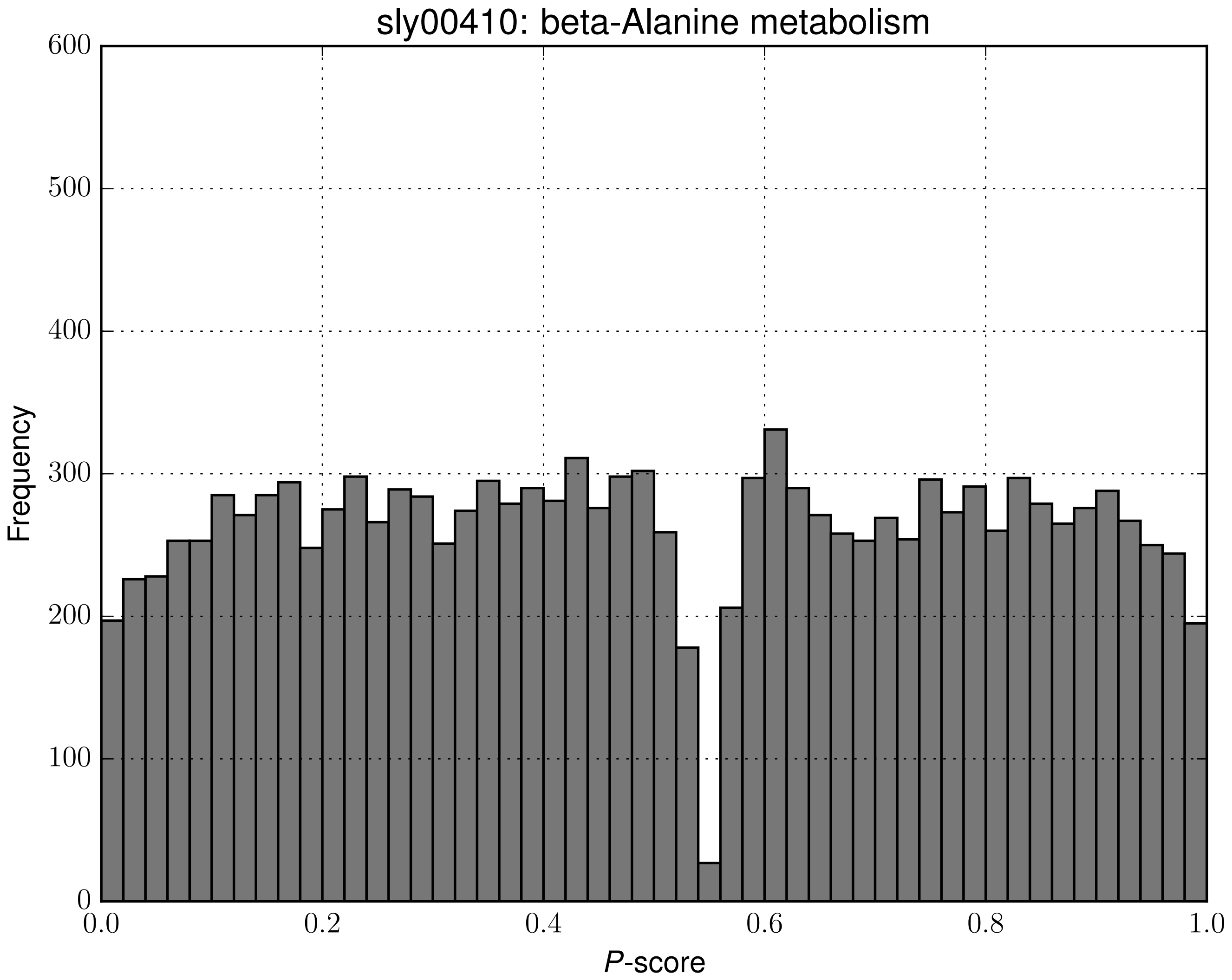

sly00410: beta-Alanine metabolism
Frequency
P-score
0
100
200
300
400
500
600
0.0
0.2
0.4
0.6
0.8
1.0

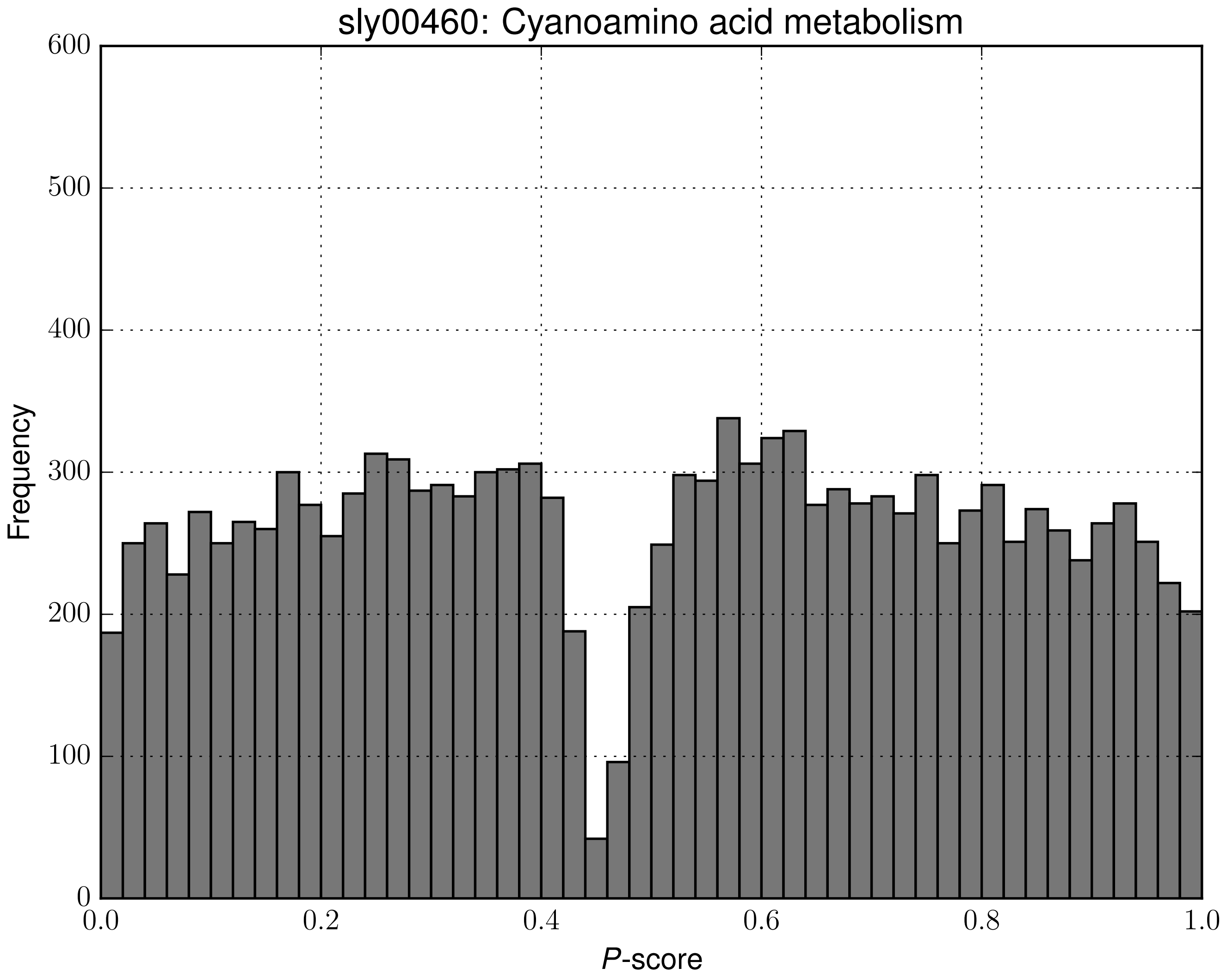

sly00460: Cyanoamino acid metabolism
600
500
400
300
200
100
0
0.0
0.2
0.4
0.6
0.8
1.0
Frequency
P-score

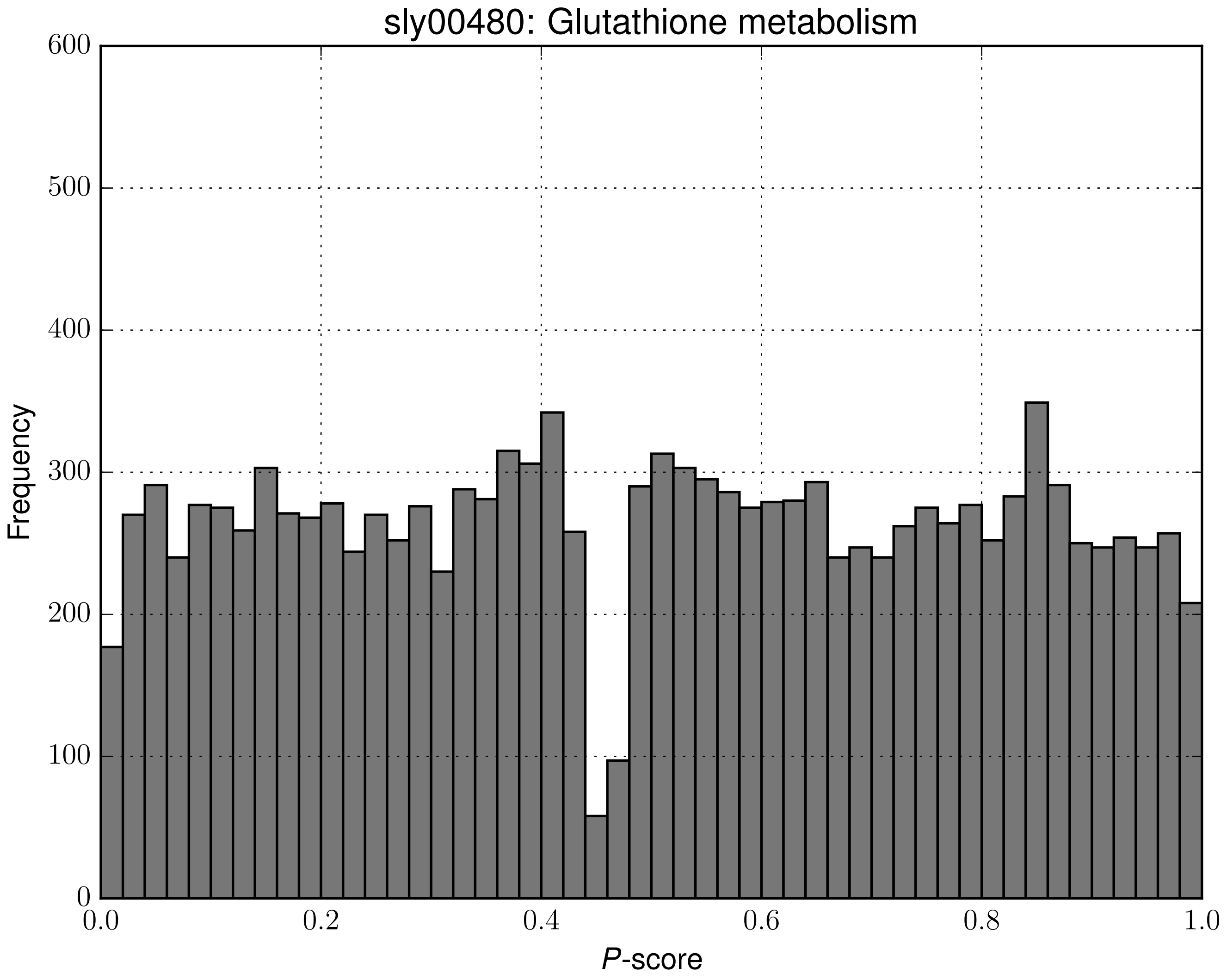
sly00480: Glutathione metabolism
Frequency
P-score
0
100
200
300
400
500
600
0.0
0.2
0.4
0.6
0.8
1.0

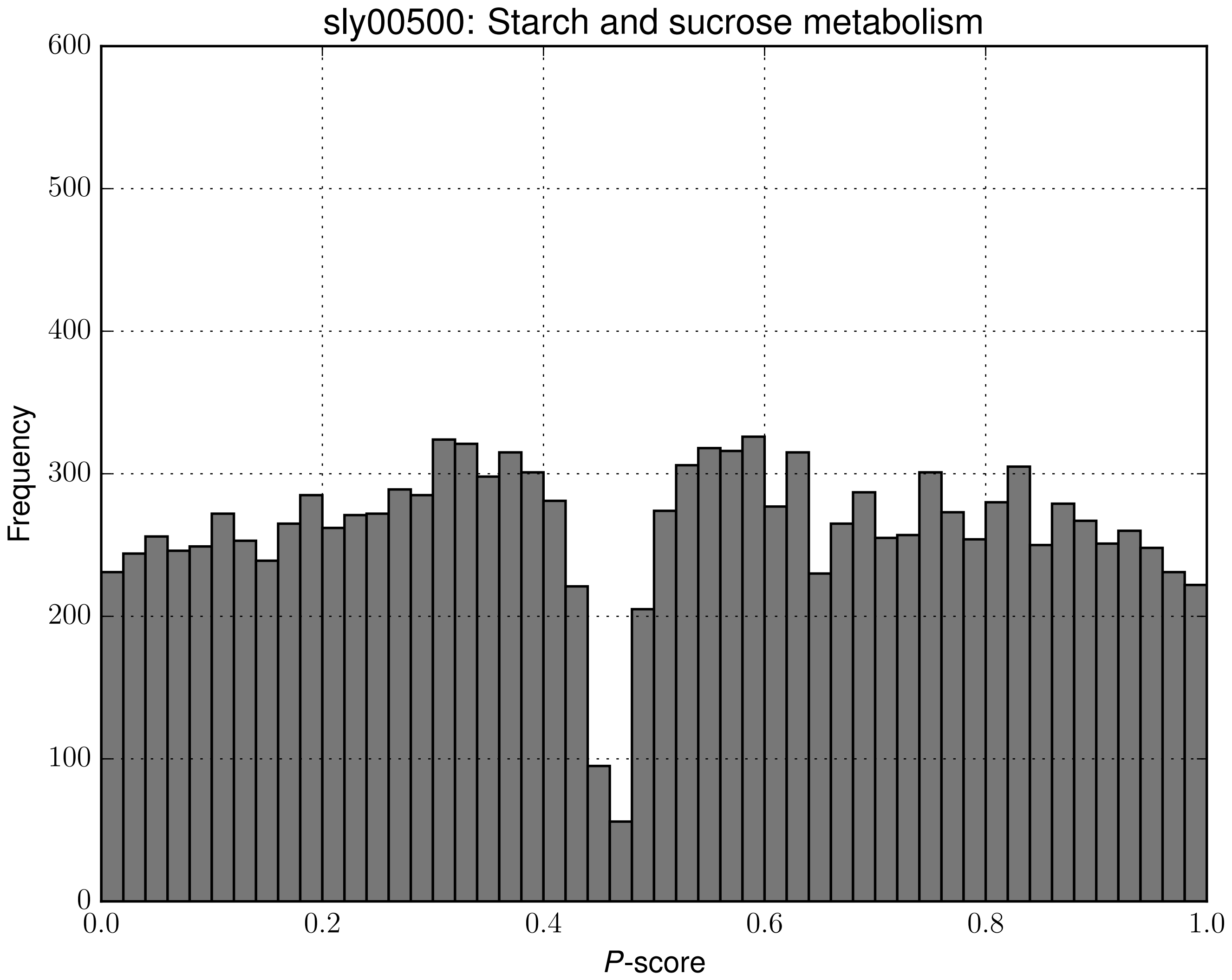

sly00500: Starch and sucrose metabolism
Frequency
P-score
0
100
200
300
400
500
600
0.0
0.2
0.4
0.6
0.8
1.0

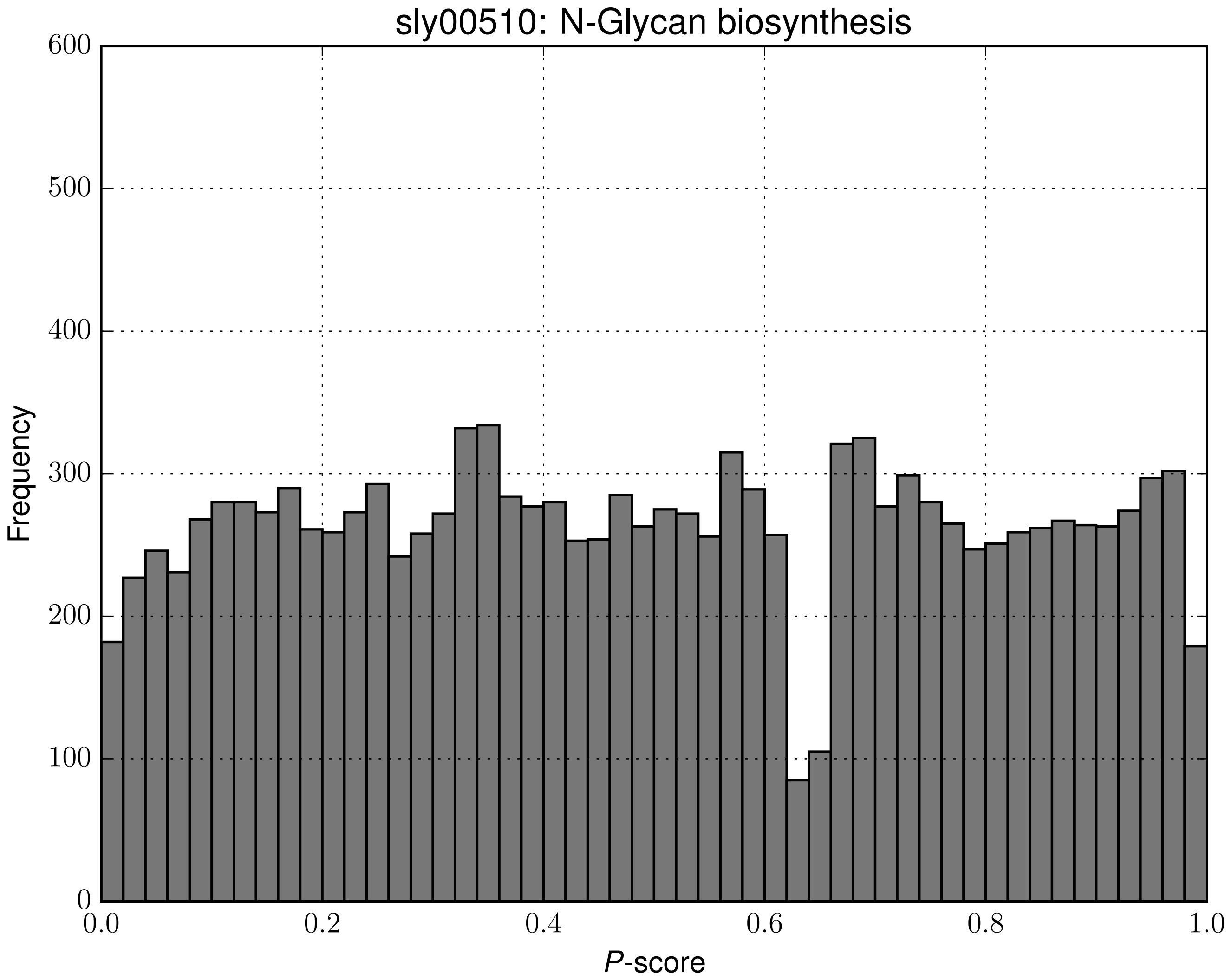
sly00510: N-Glycan biosynthesis
Frequency
P-score
0
100
200
300
400
500
600
0.0
0.2
0.4
0.6
0.8
1.0

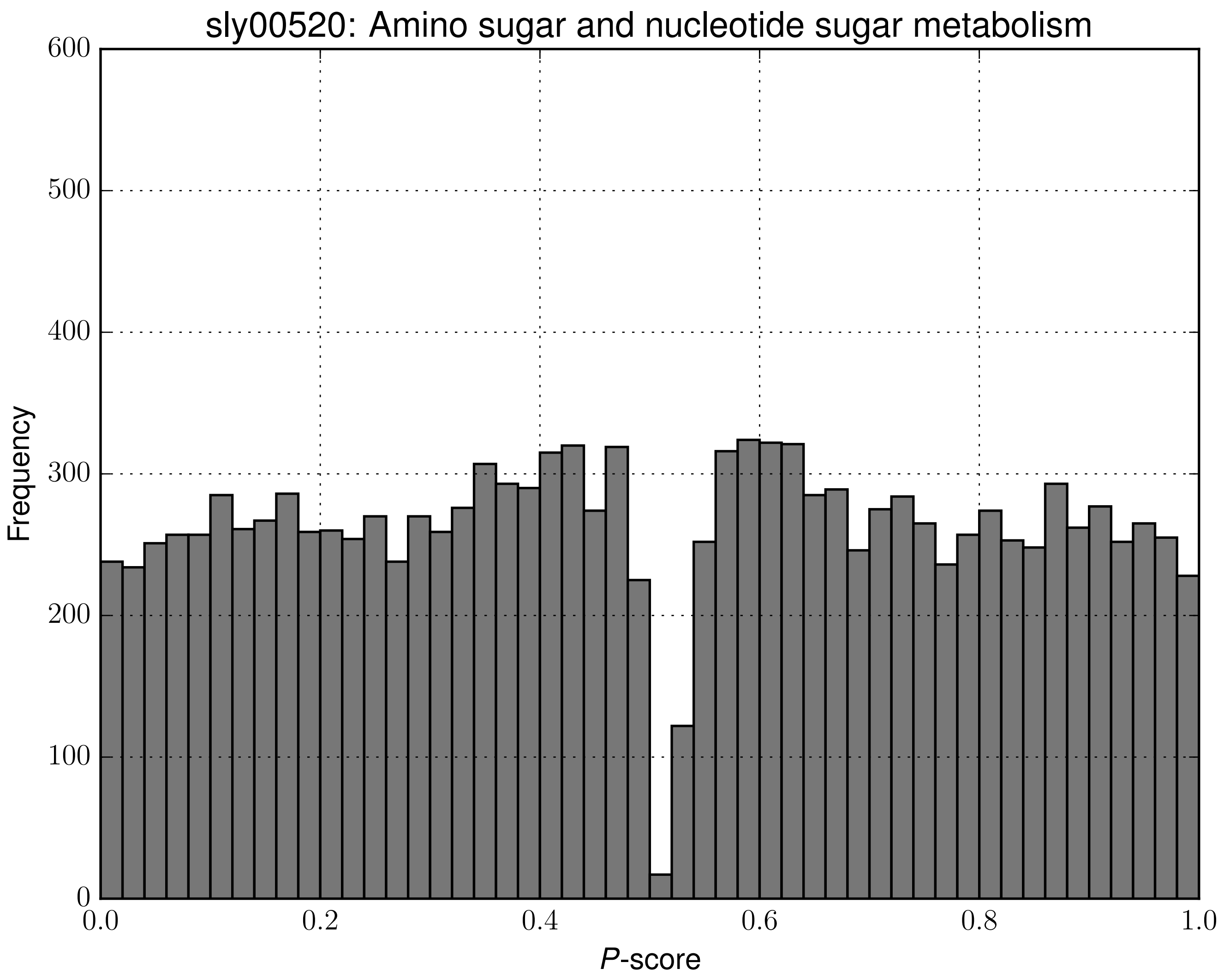
sly00520: Amino sugar and nucleotide sugar metabolism
Frequency
600
500
400
300
200
100
0
0.0
0.2
0.4
0.6
0.8
1.0
P-score

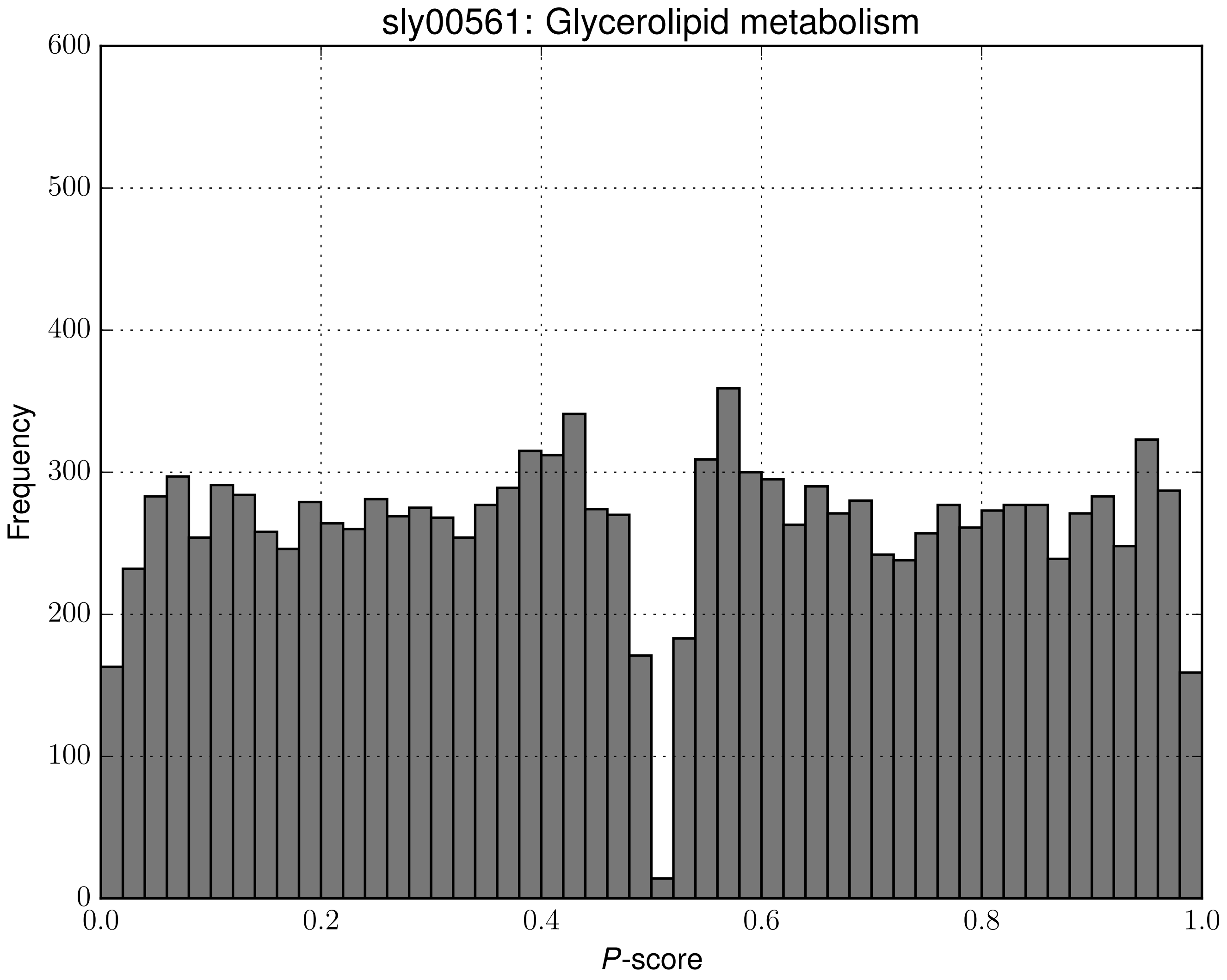
sly00561: Glycerolipid metabolism
Frequency
P-score
0
100
200
300
400
500
600
0.0
0.2
0.4
0.6
0.8
1.0

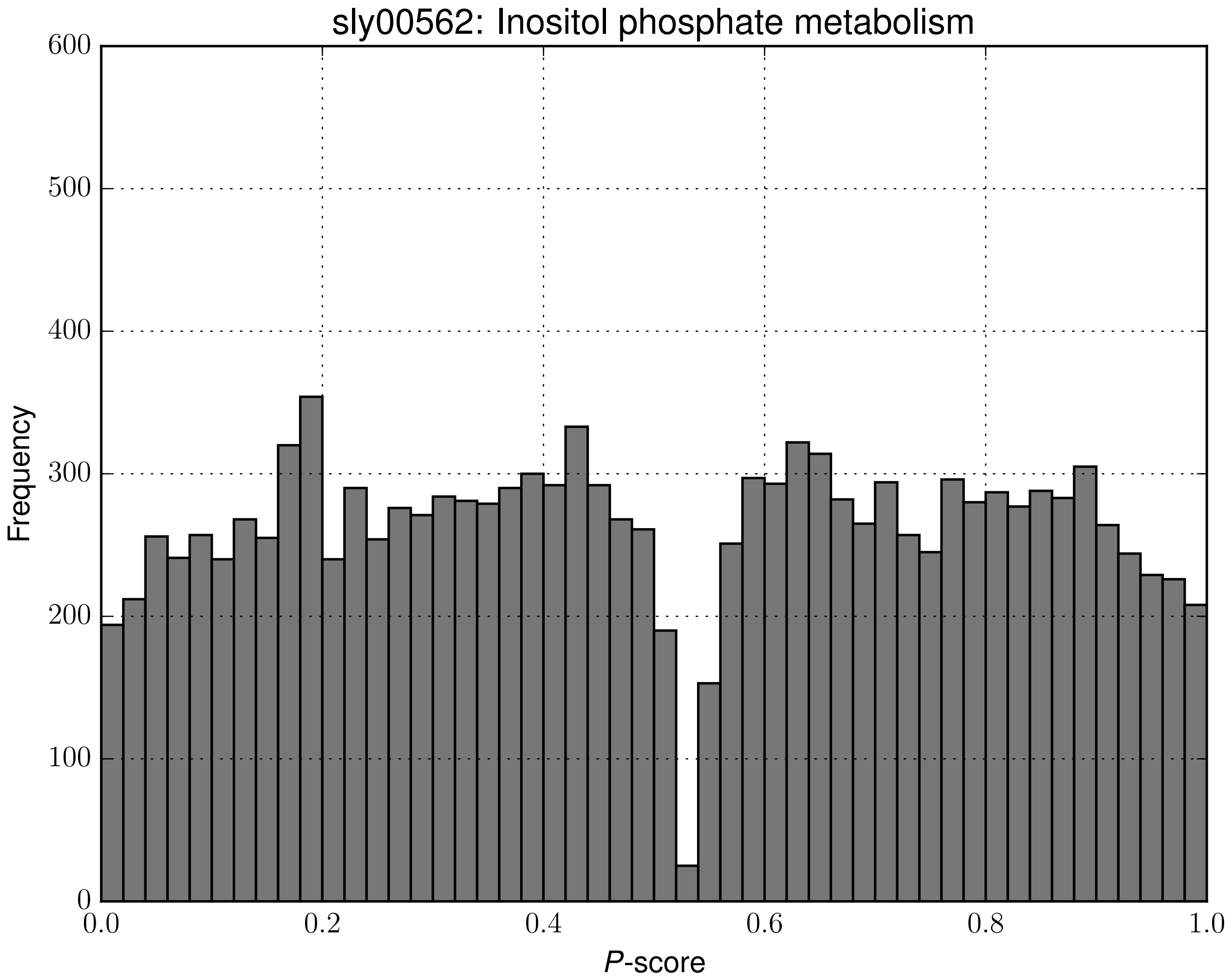
sly00562: Inositol phosphate metabolism
Frequency
P-score
0
100
200
300
400
500
600
0.0
0.2
0.4
0.6
0.8
1.0

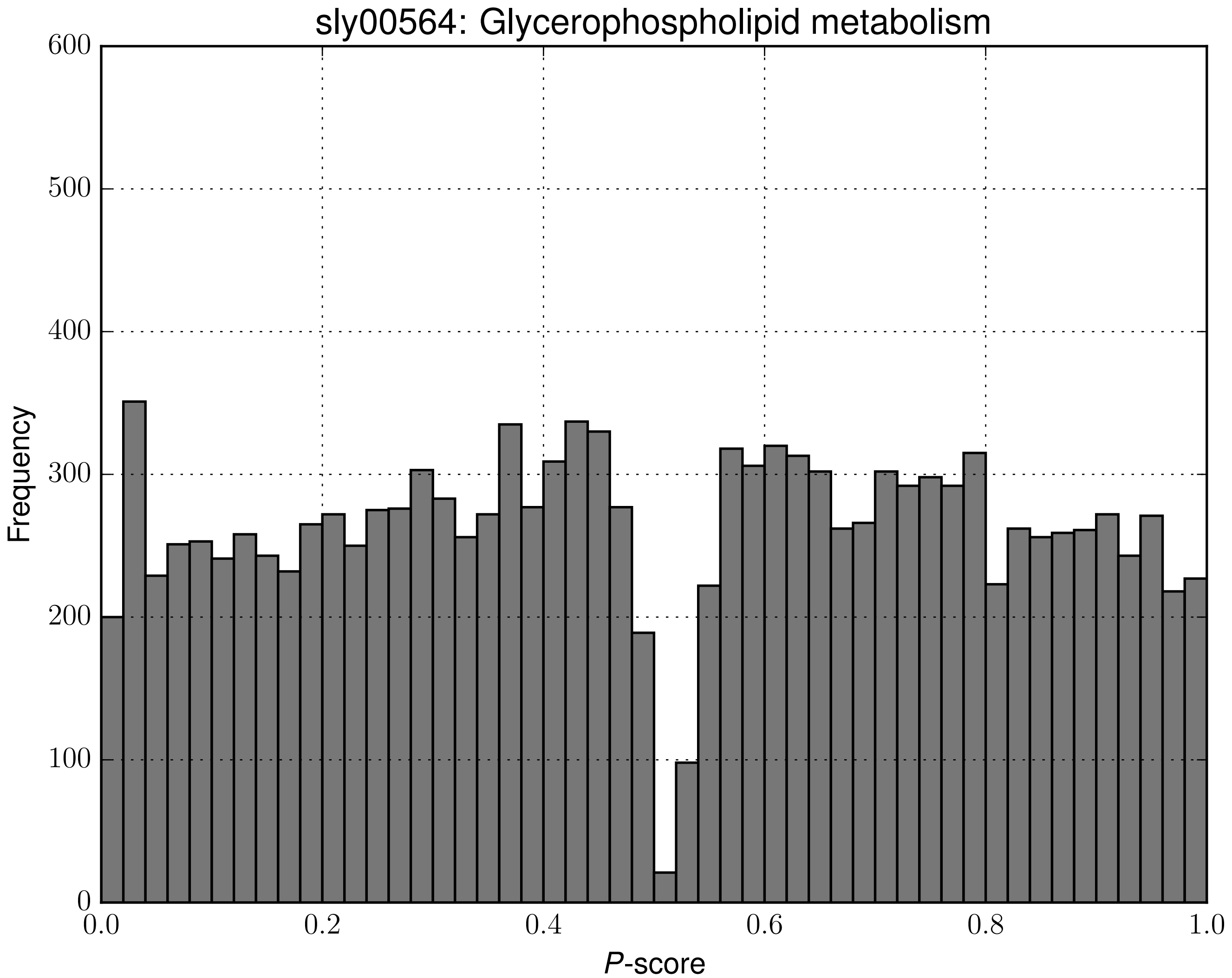
sly00564: Glycerophospholipid metabolism
600
500
400
300
200
100
0
Frequency
0.0
0.2
0.4
0.6
0.8
1.0
P-score

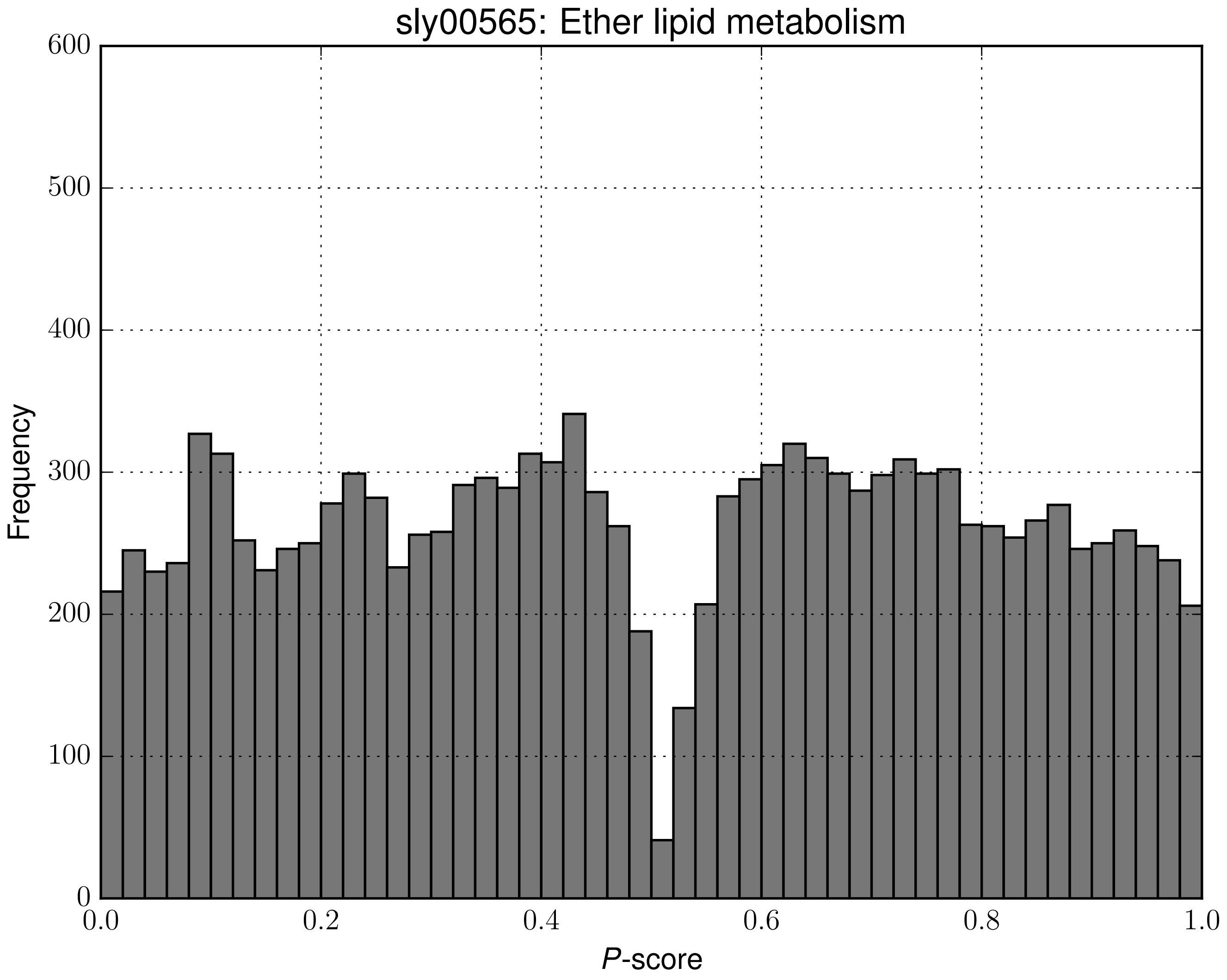
sly00565: Ether lipid metabolism
Frequency
P-score
0
100
200
300
400
500
600
0.0
0.2
0.4
0.6
0.8
1.0

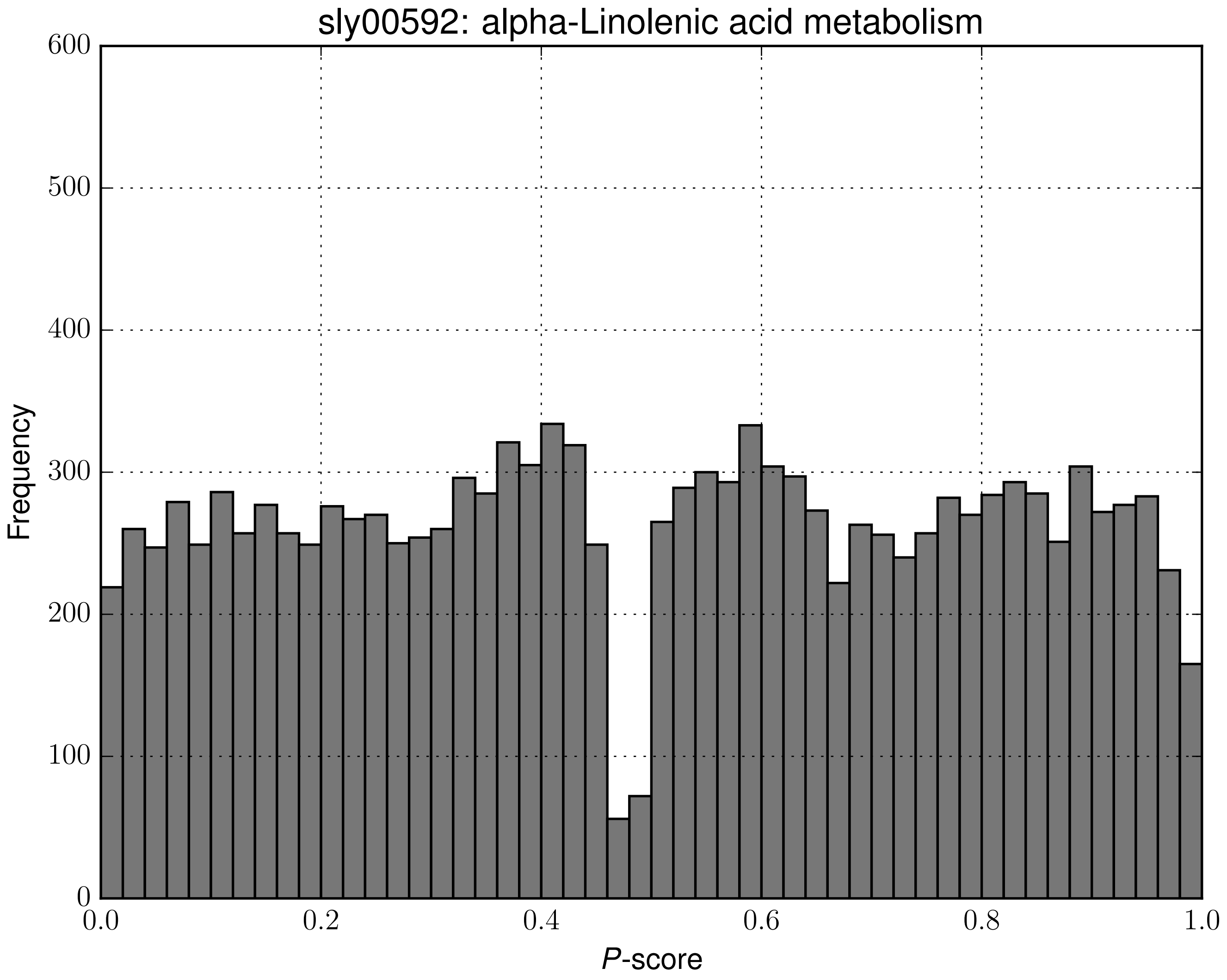

sly00592: alpha-Linolenic acid metabolism
Frequency
P-score
0
100
200
300
400
500
600
0.0
0.2
0.4
0.6
0.8
1.0

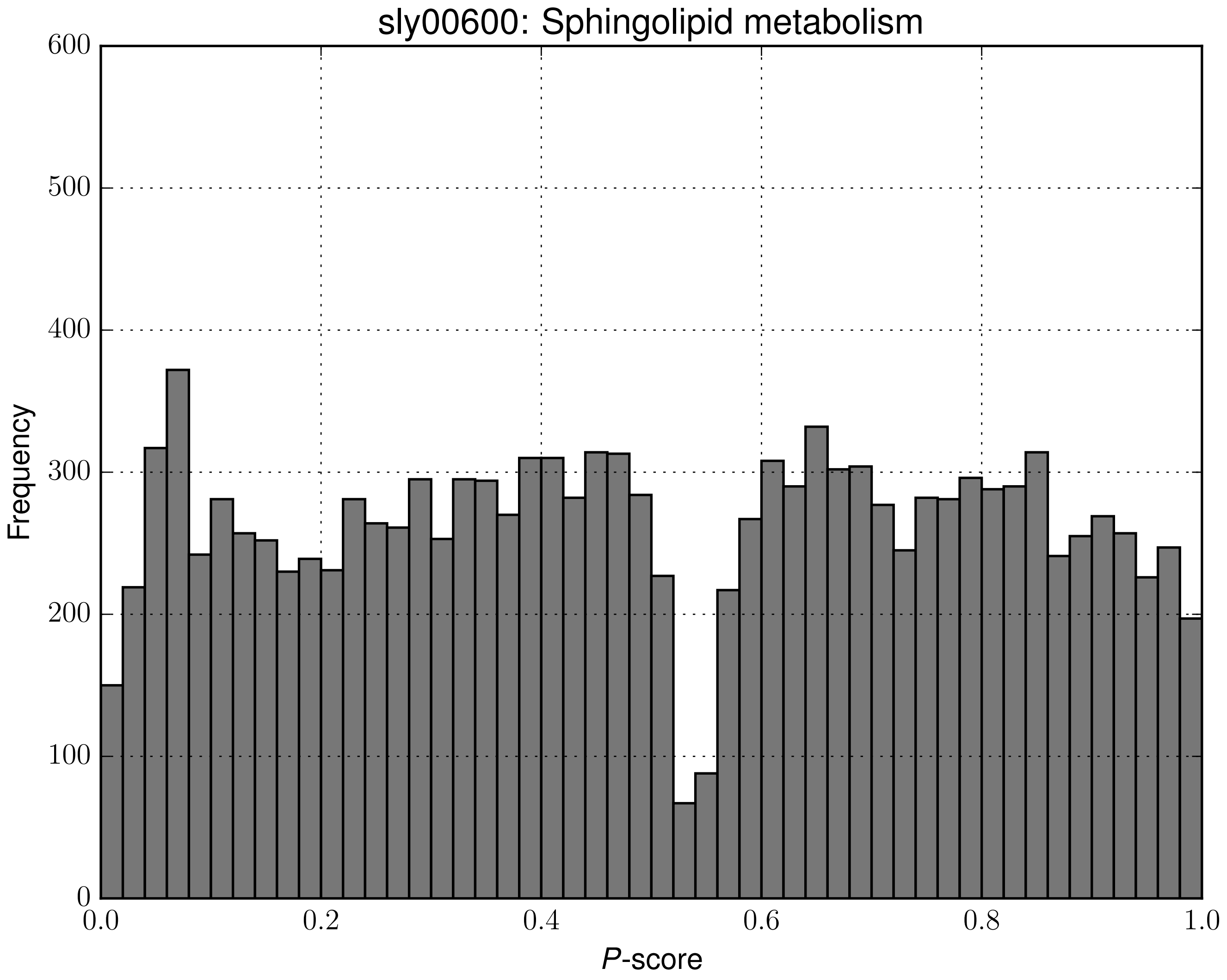

sly00600: Sphingolipid metabolism
600
500
400
300
200
100
0
Frequency
0.0
0.2
0.4
0.6
0.8
1.0
P-score

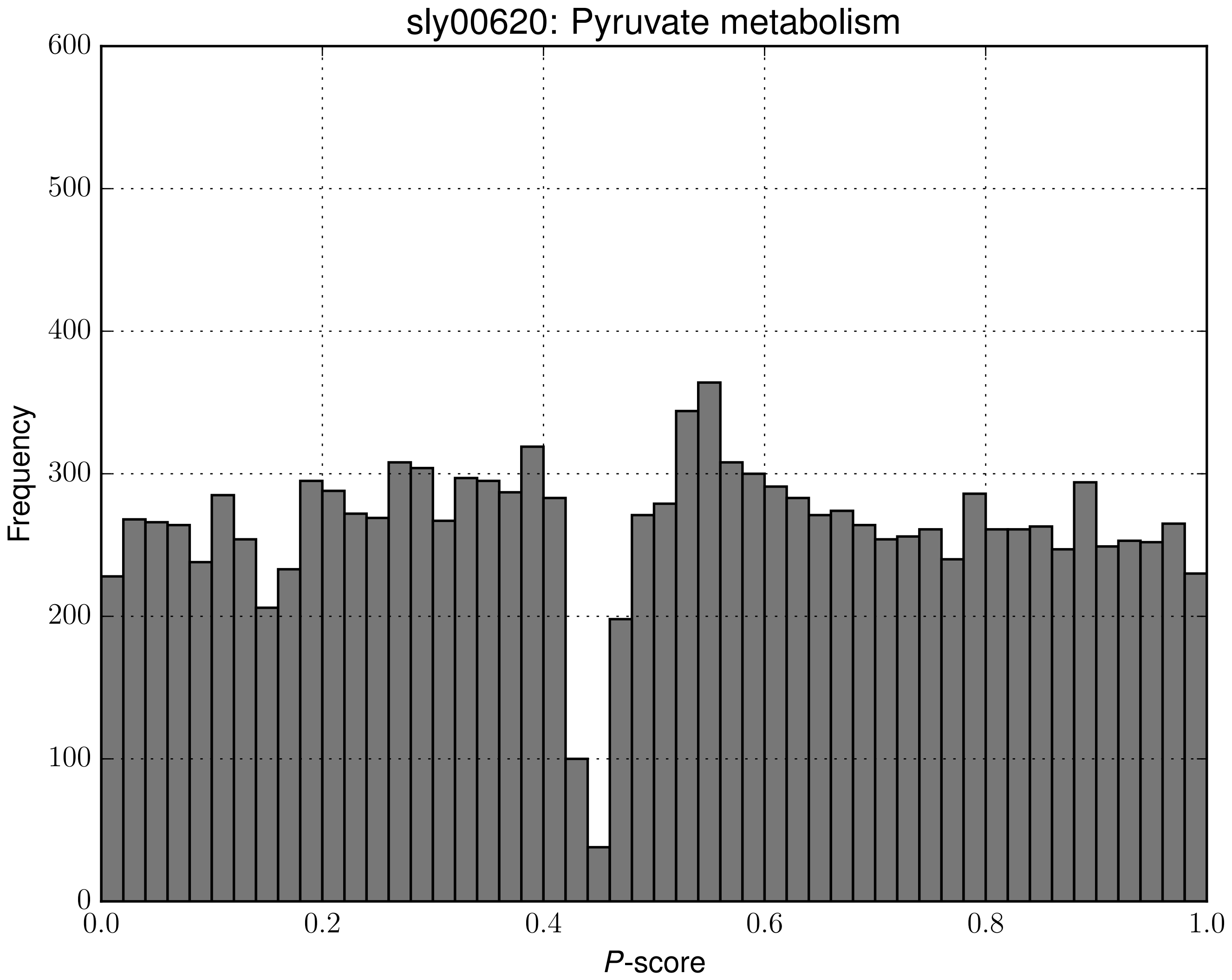

sly00620: Pyruvate metabolism
Frequency
P-score
0
100
200
300
400
500
600
0.0
0.2
0.4
0.6
0.8
1.0

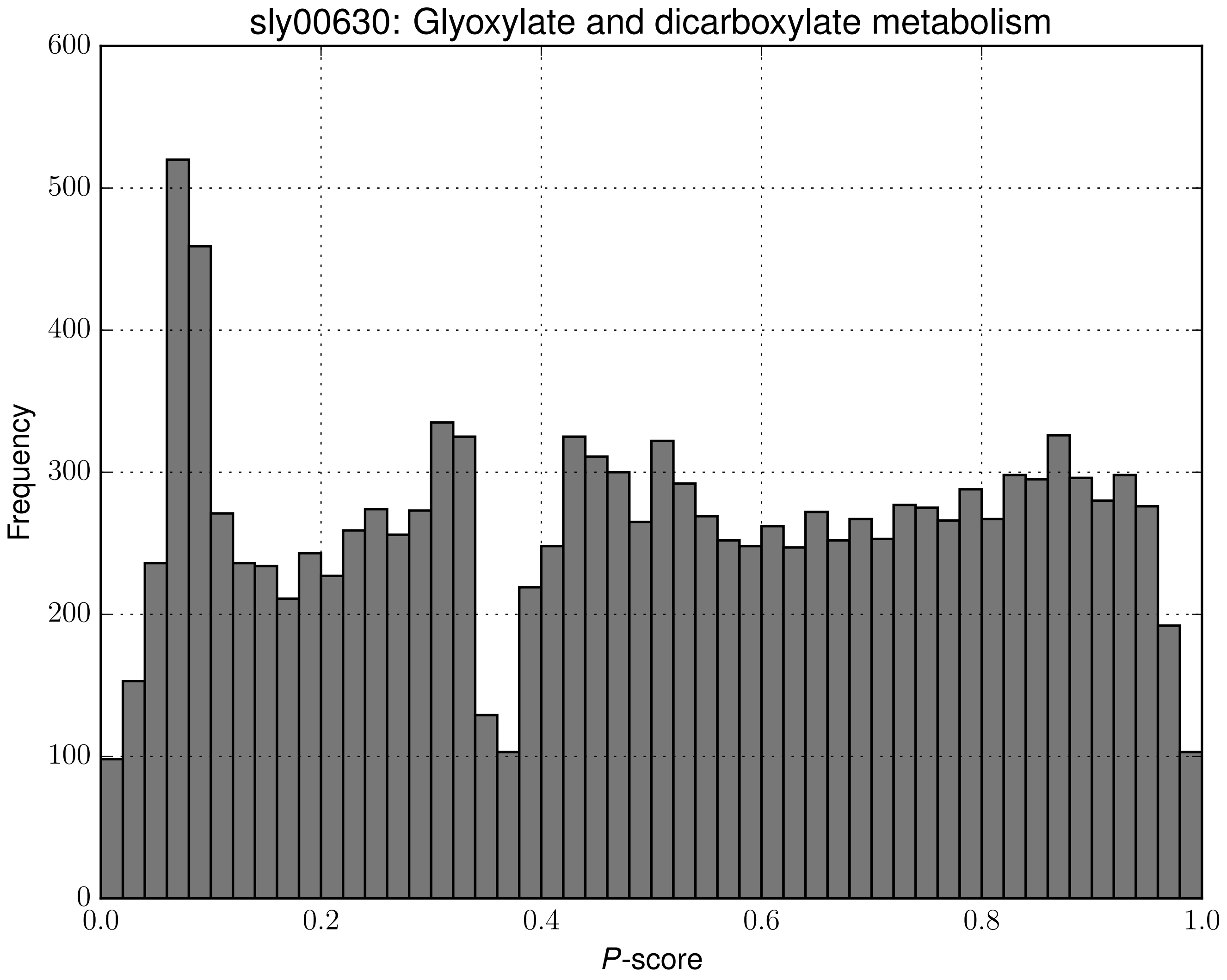
sly00630: Glyoxylate and dicarboxylate metabolism
Frequency
0
100
200
300
400
500
600
0.0
0.2
0.4
0.6
0.8
1.0
P-score

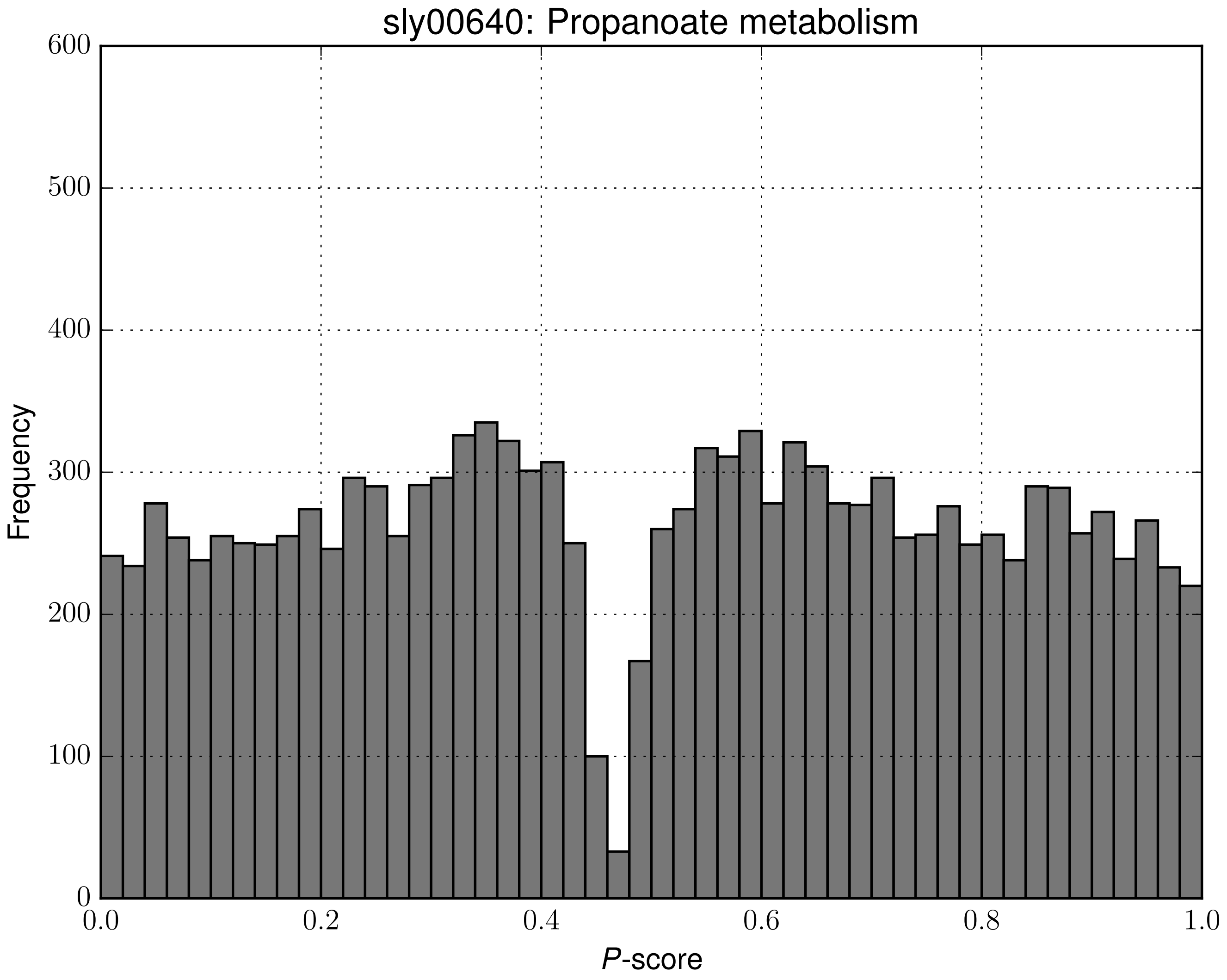
sly00640: Propanoate metabolism
Frequency
P-score
0
100
200
300
400
500
600
0.0
0.2
0.4
0.6
0.8
1.0

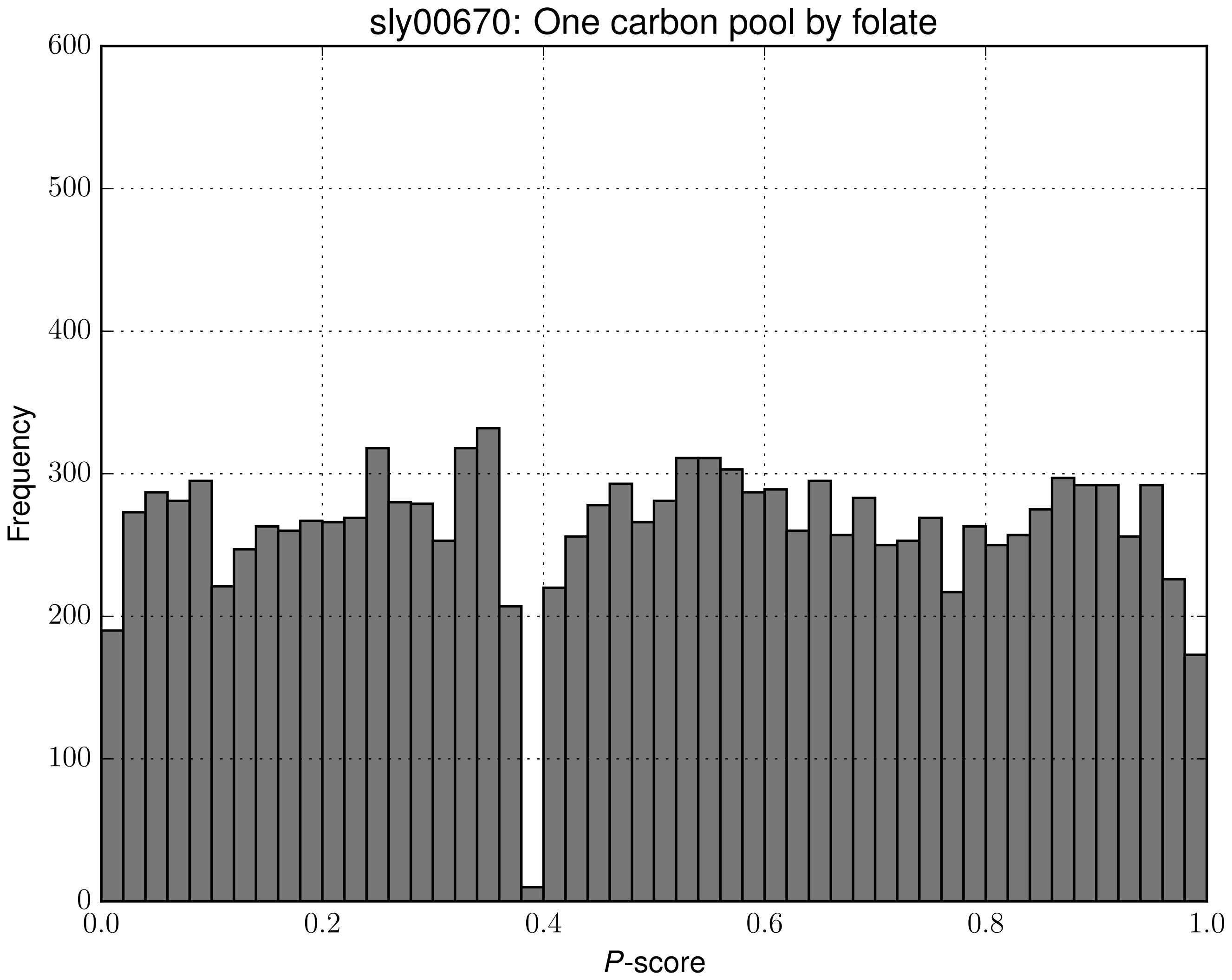
sly00670: One carbon pool by folate
Frequency
P-score
0
100
200
300
400
500
600
0.0
0.2
0.4
0.6
0.8
1.0

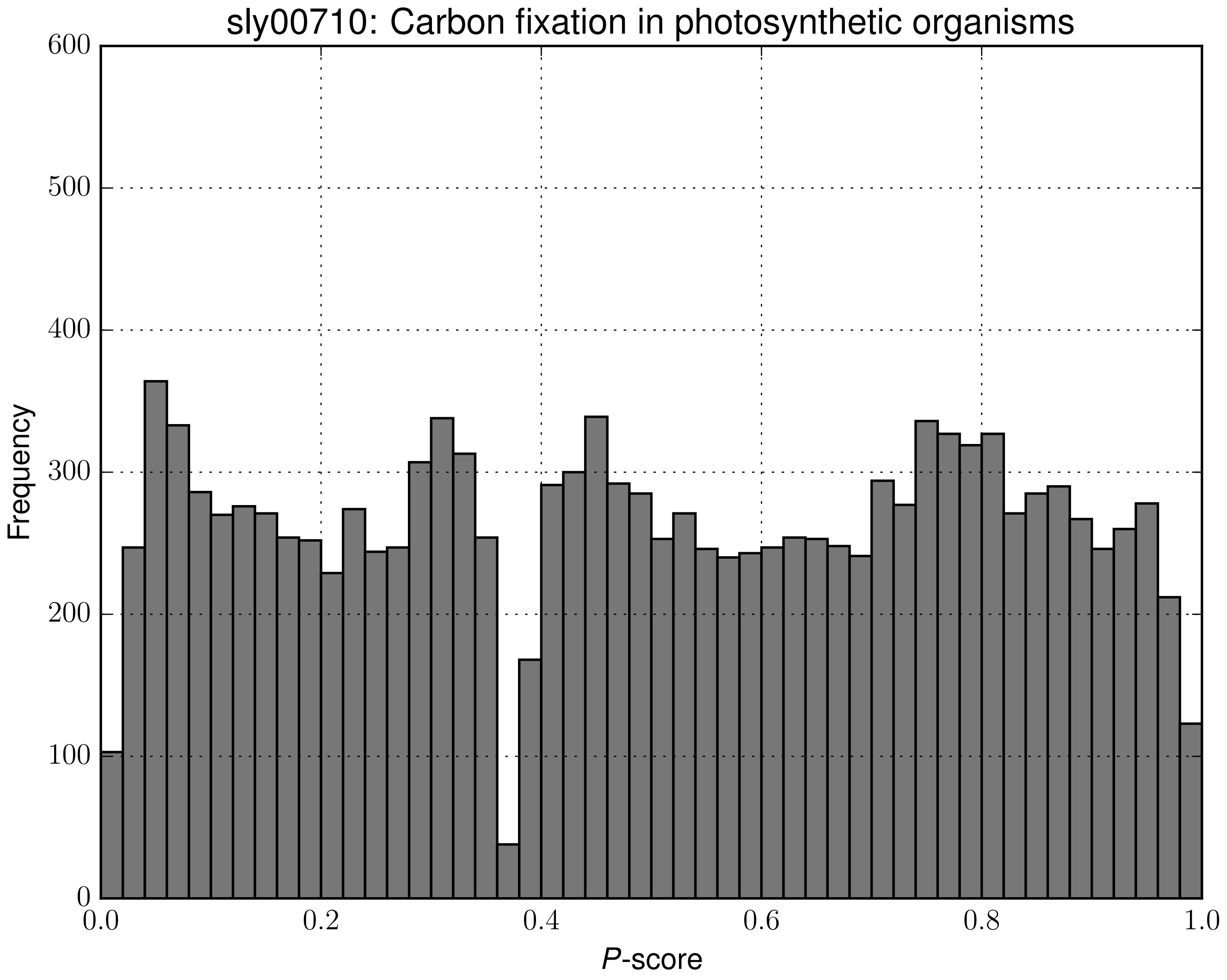
sly00710: Carbon fixation in photosynthetic organisms
Frequency
P-score
0
100
200
300
400
500
600
0.0
0.2
0.4
0.6
0.8
1.0

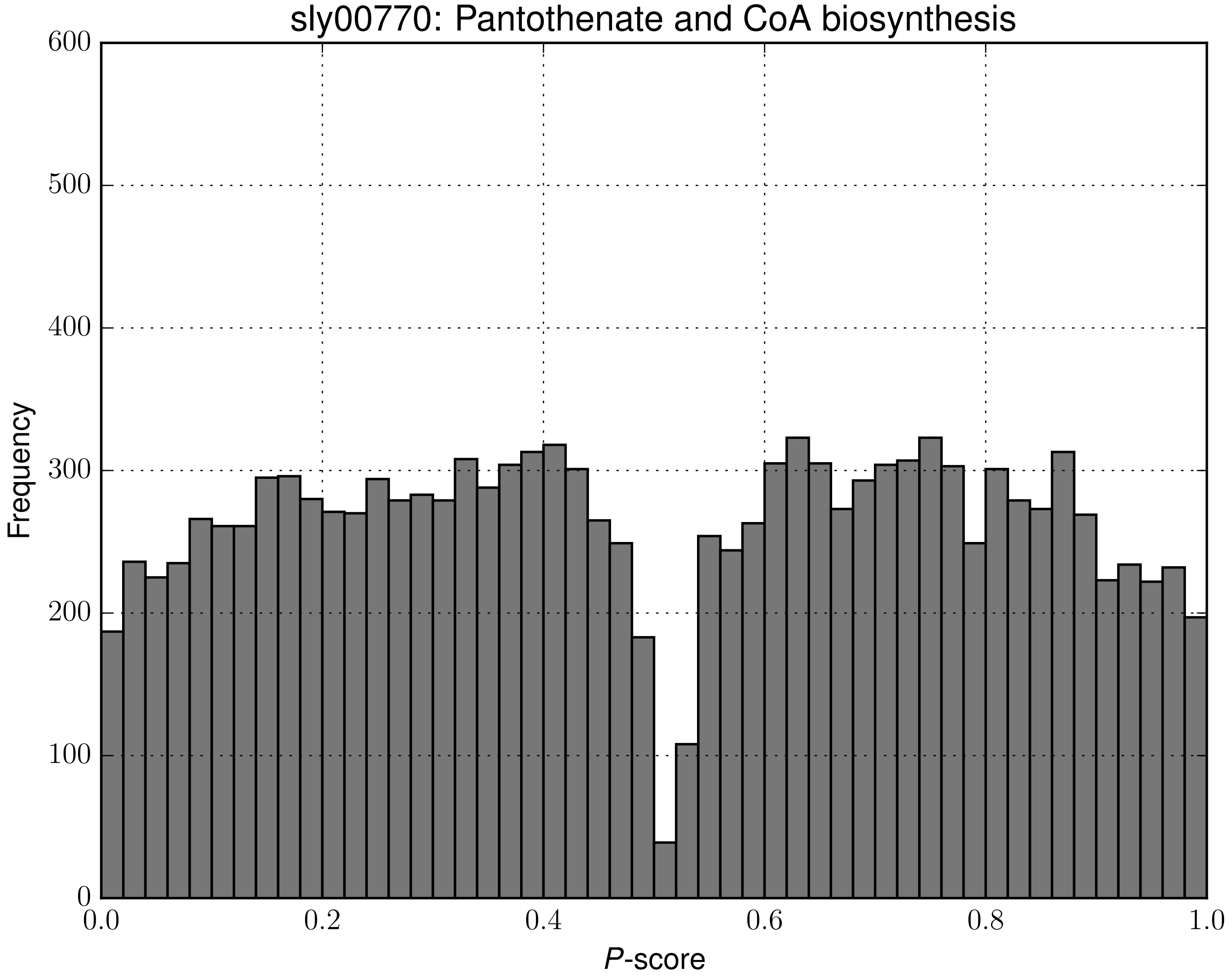
sly00770: Pantothenate and CoA biosynthesis
Frequency
P-score
0
100
200
300
400
500
600
0.0
0.2
0.4
0.6
0.8
1.0

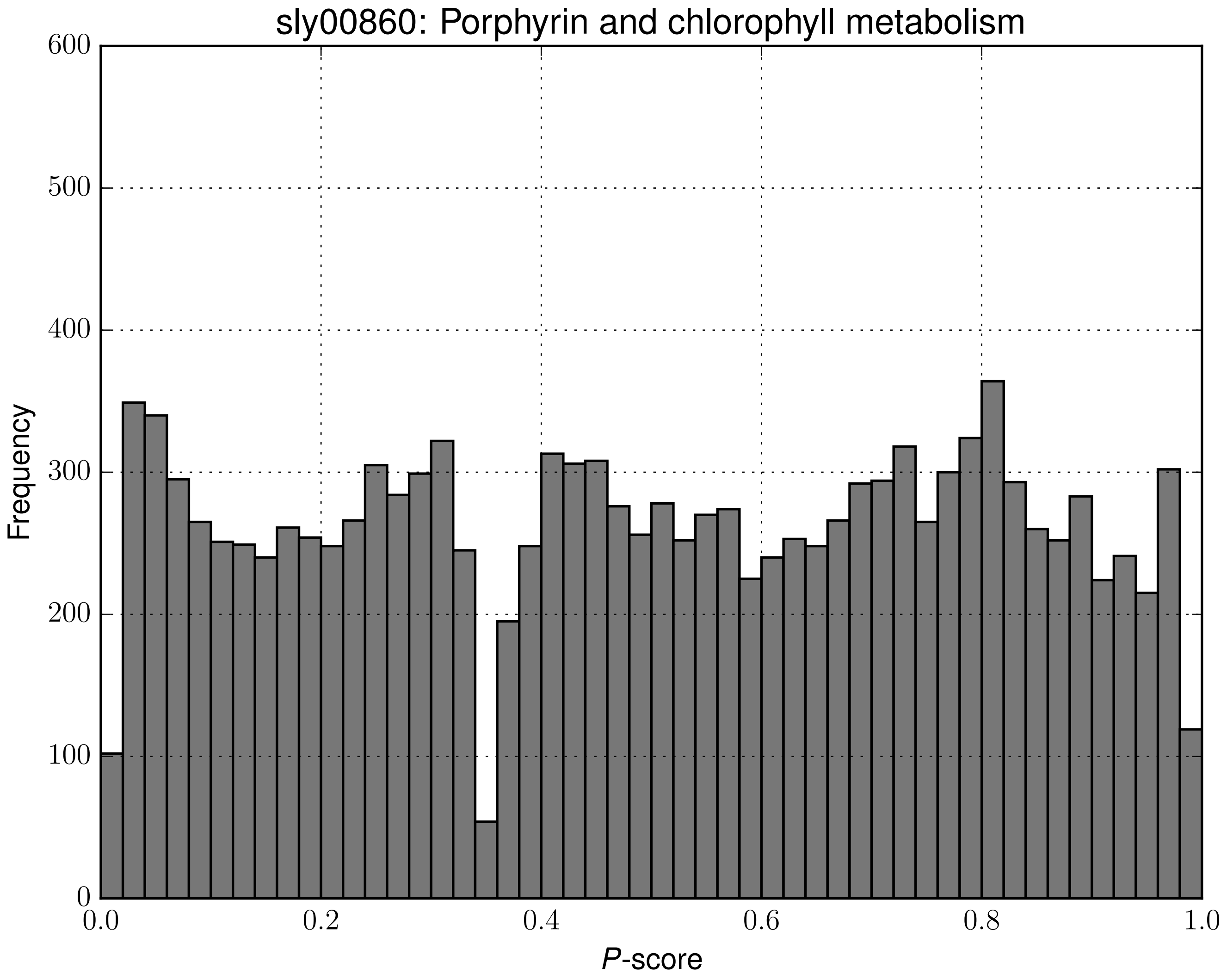
sly00860: Porphyrin and chlorophyll metabolism
Frequency
P-score
0
100
200
300
400
500
600
0.0
0.2
0.4
0.6
0.8
1.0

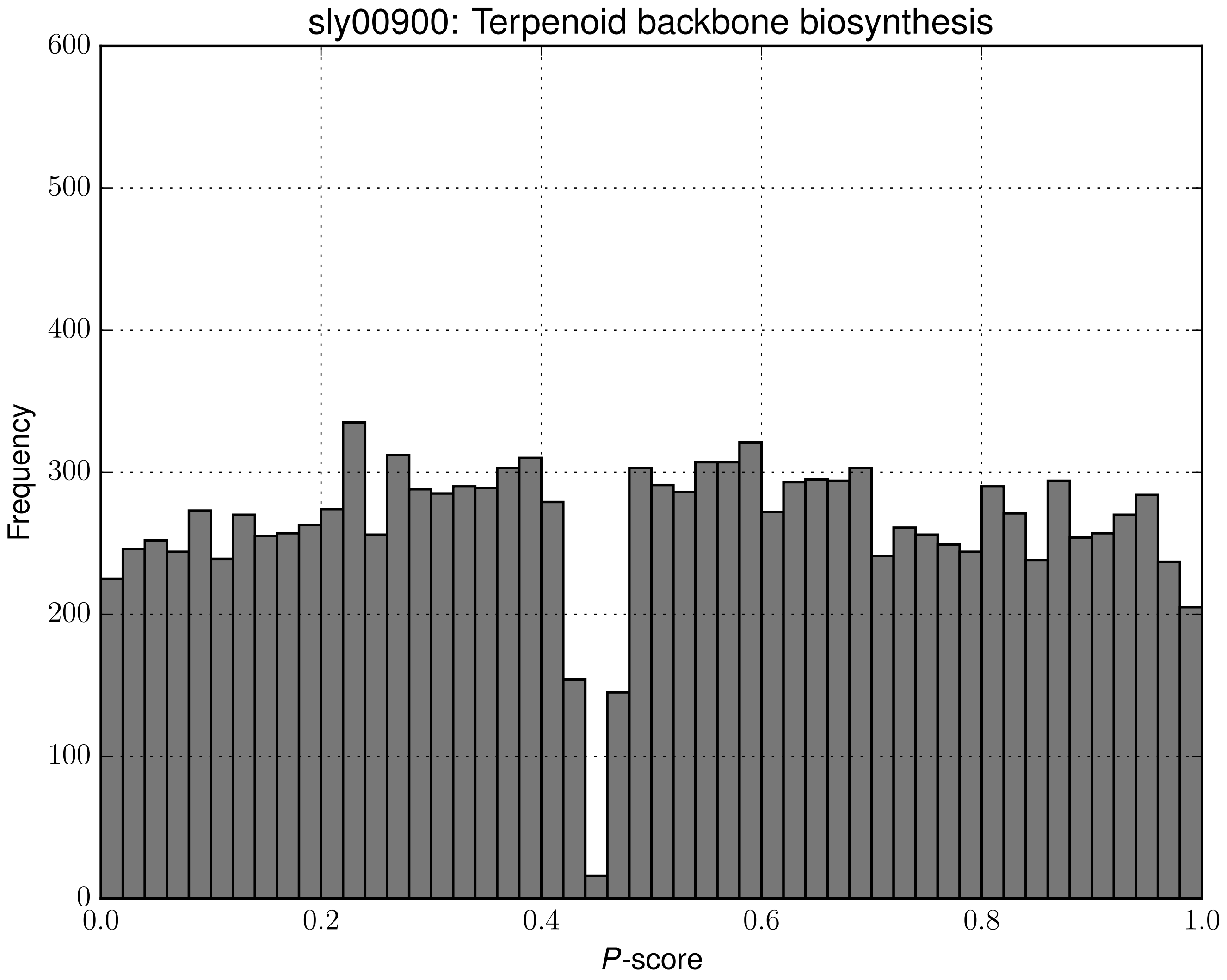
sly00900: Terpenoid backbone biosynthesis
Frequency
P-score
0
100
200
300
400
500
600
0.0
0.2
0.4
0.6
0.8
1.0

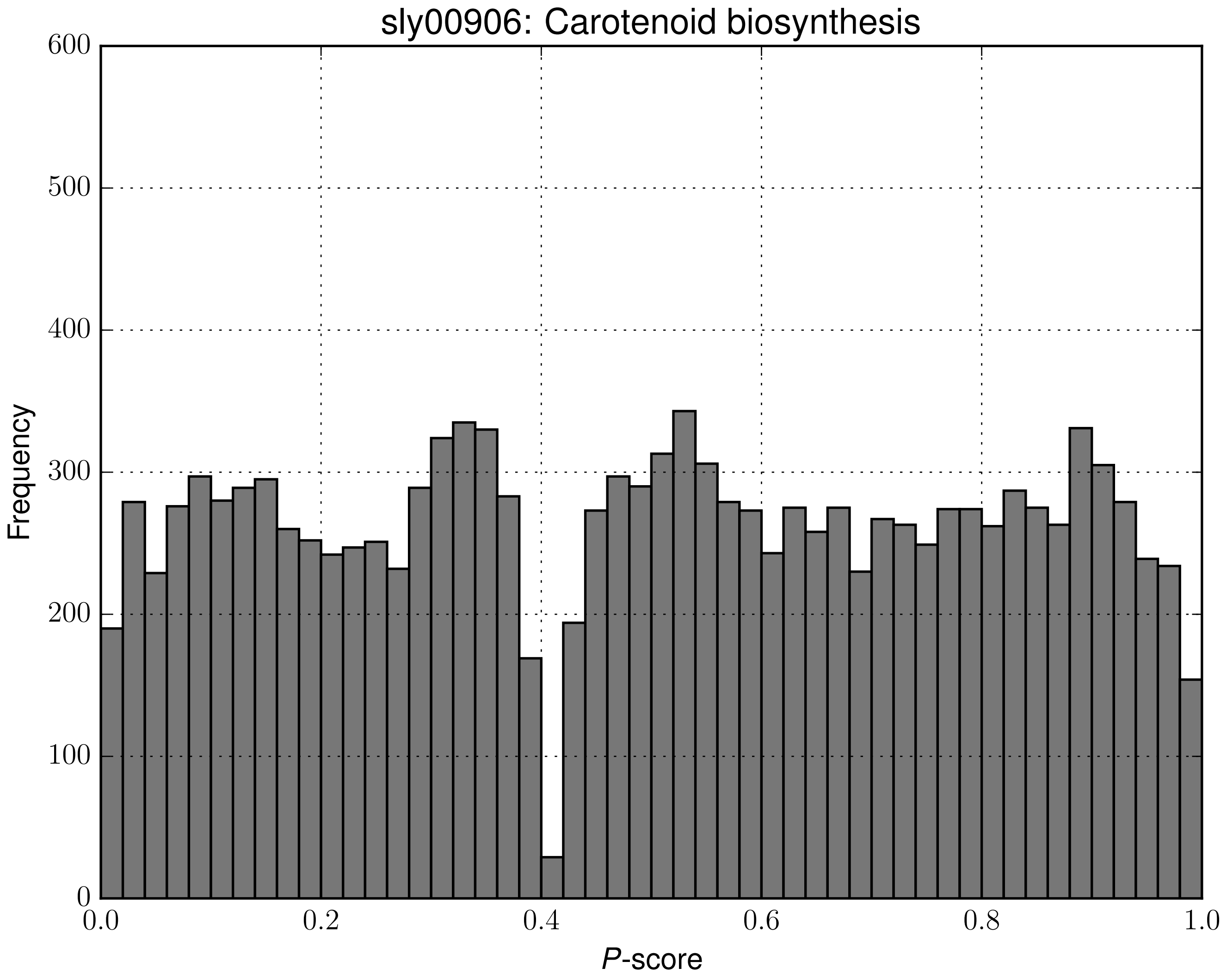
sly00906: Carotenoid biosynthesis
600
500
400
300
200
100
0
0.0
0.2
0.4
0.6
0.8
1.0
Frequency
P-score

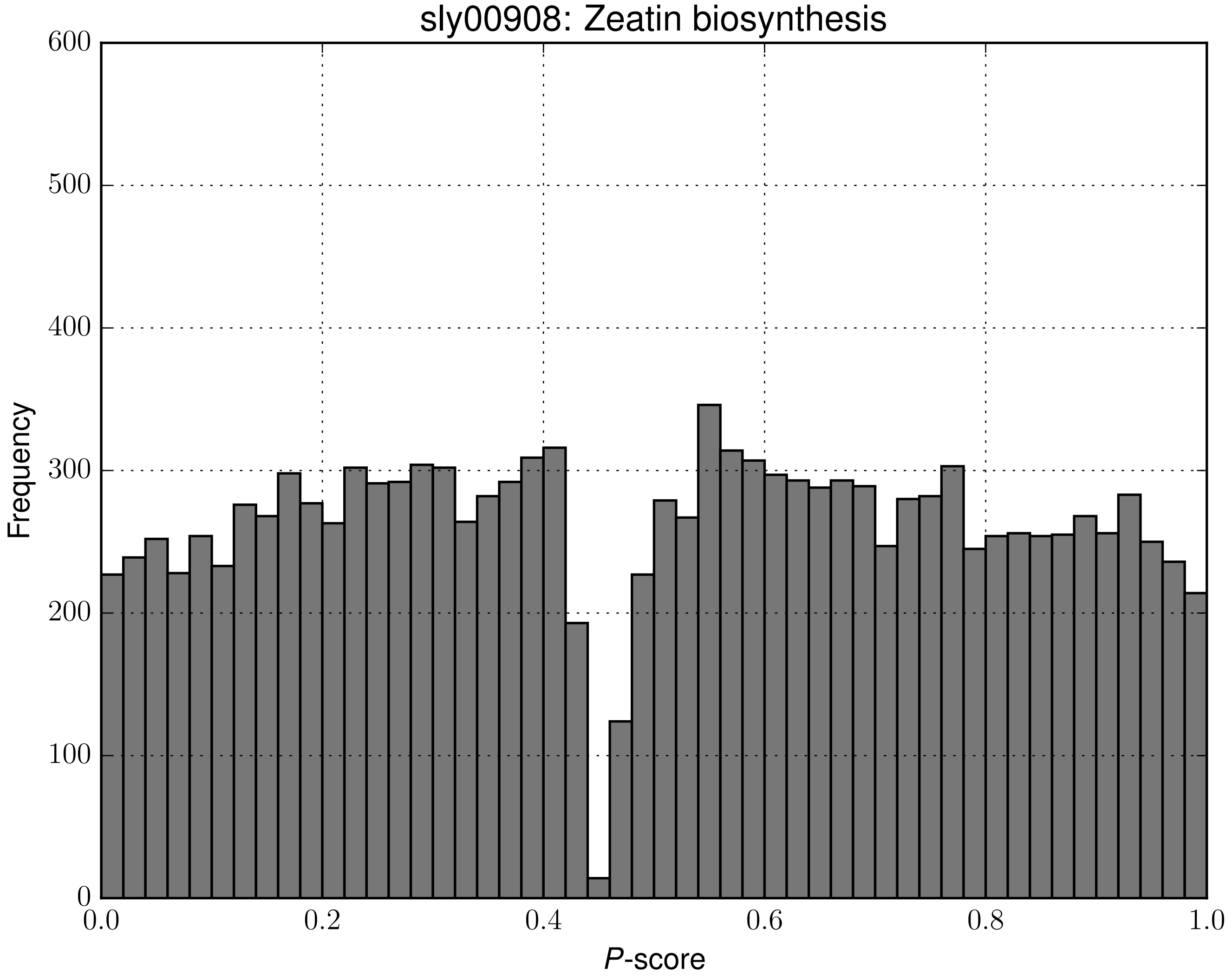
sly00908: Zeatin biosynthesis
Frequency
P-score
0
100
200
300
400
500
600
0.0
0.2
0.4
0.6
0.8
1.0

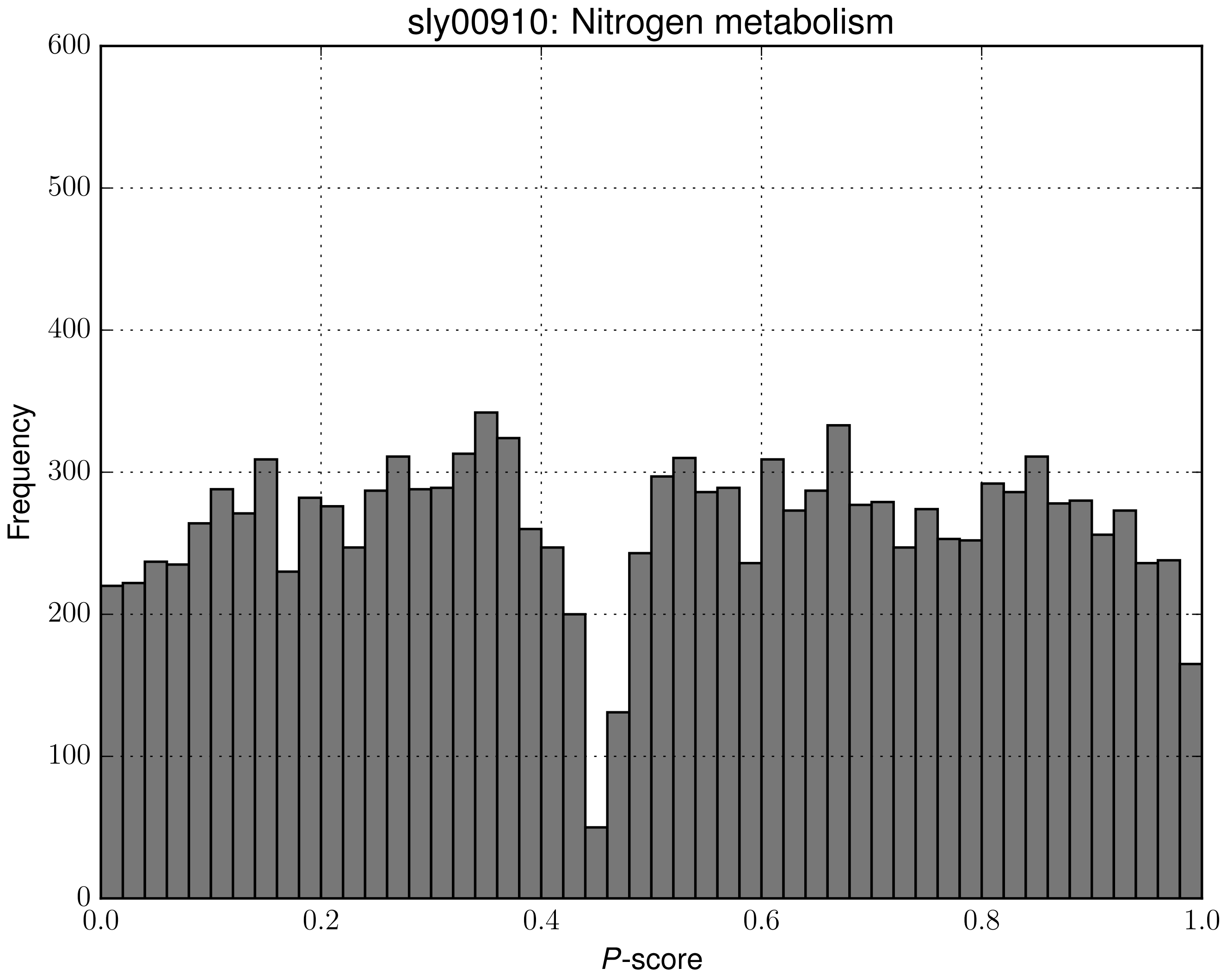
sly00910: Nitrogen metabolism
Frequency
P-score
0
100
200
300
400
500
600
0.0
0.2
0.4
0.6
0.8
1.0

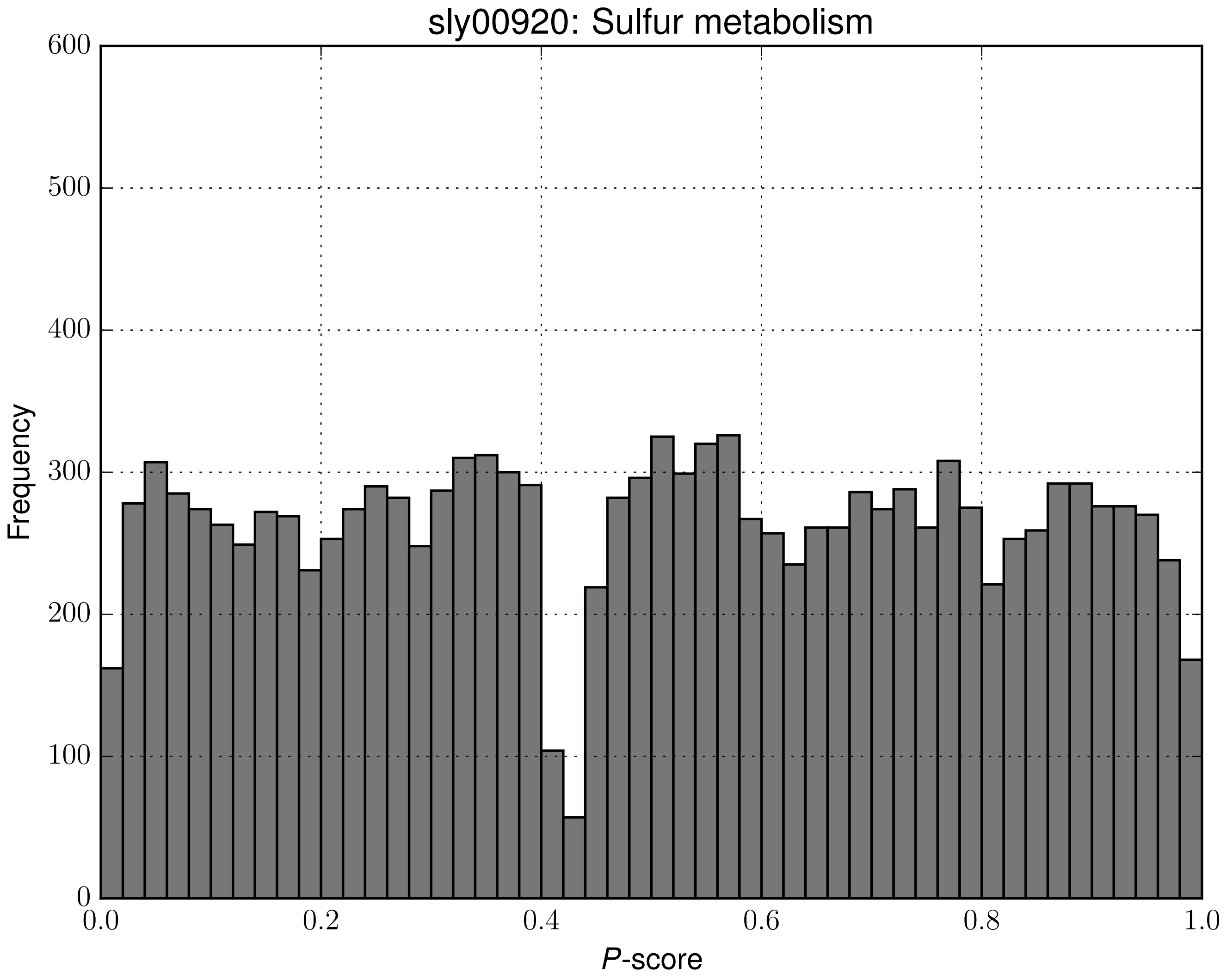

sly00920: Sulfur metabolism
600
500
400
300
200
100
0
Frequency
0.0
0.2
0.4
0.6
0.8
1.0
P-score

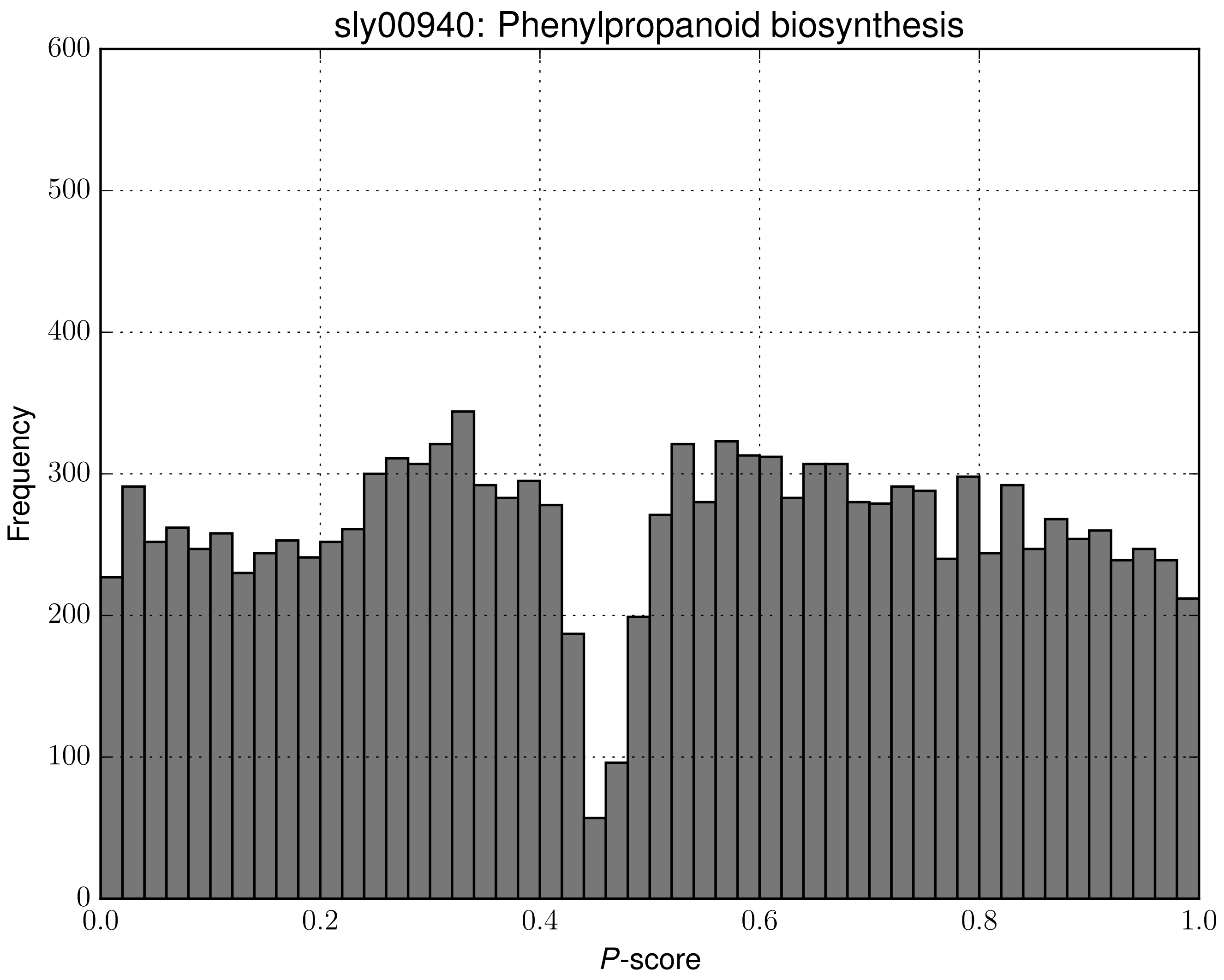

sly00940: Phenylpropanoid biosynthesis
600
500
400
300
200
100
0
0.0
0.2
0.4
0.6
0.8
1.0
Frequency
P-score

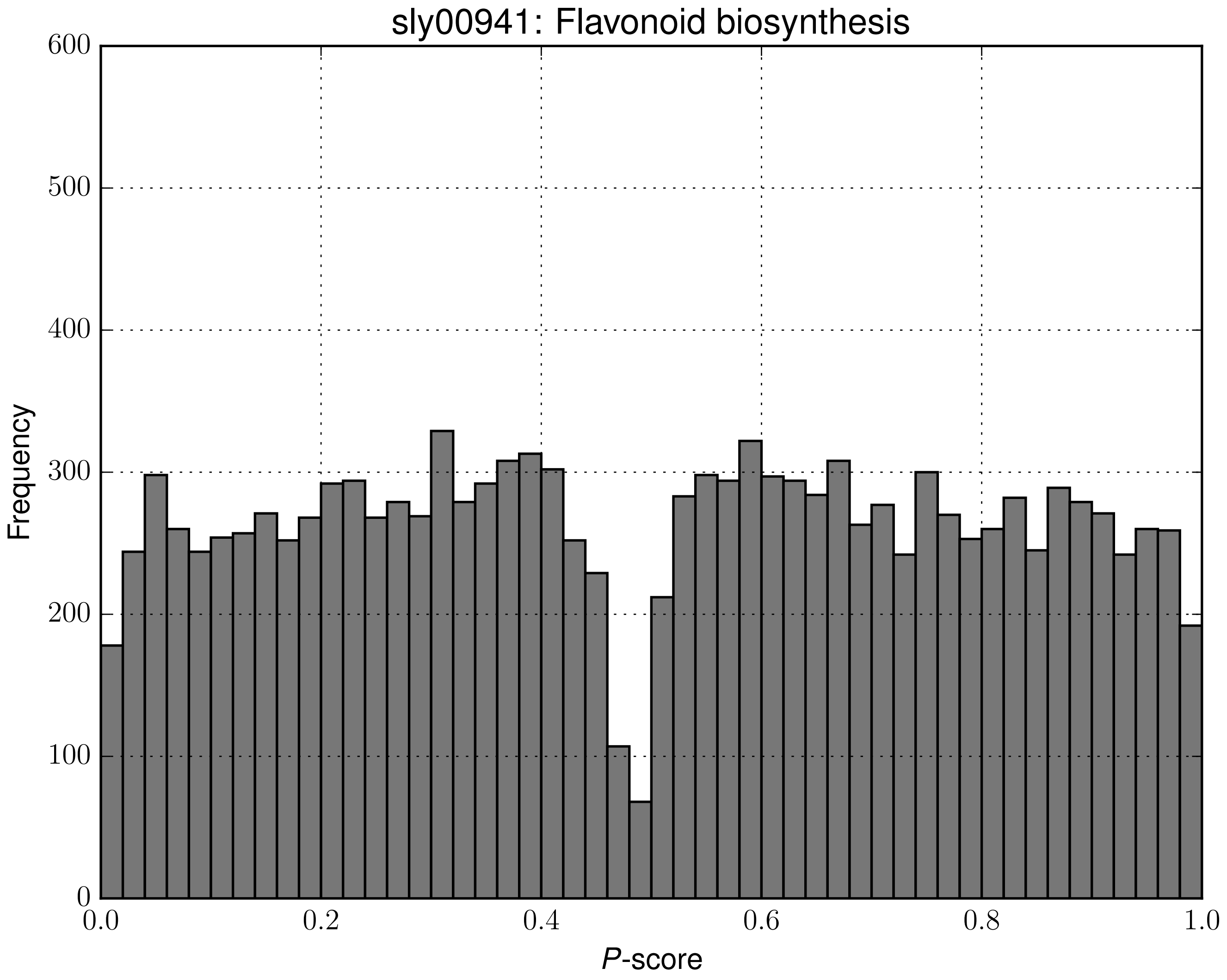
sly00941: Flavonoid biosynthesis
Frequency
P-score
0
100
200
300
400
500
600
0.0
0.2
0.4
0.6
0.8
1.0

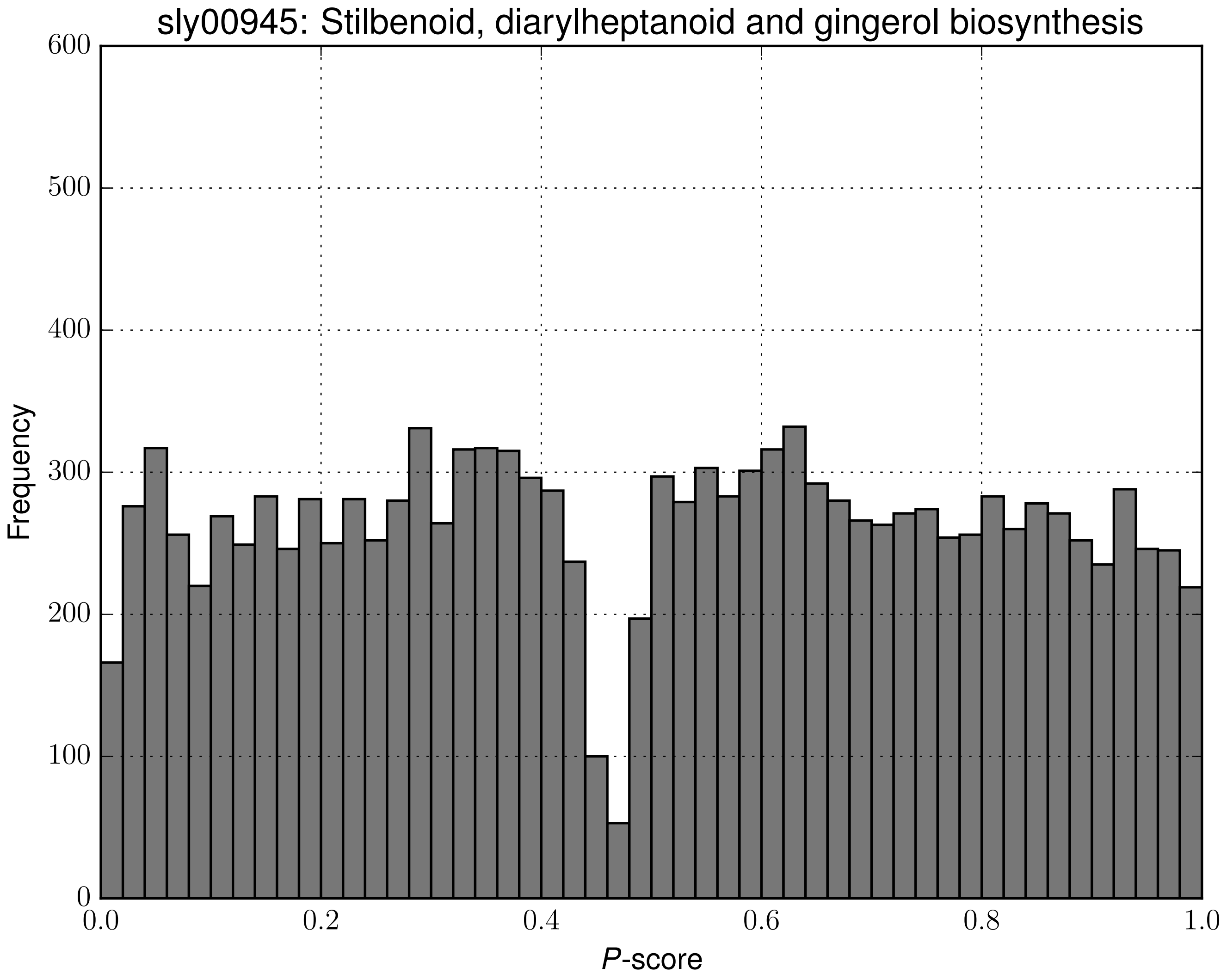
sly00945: Stilbenoid, diarylheptanoid and gingerol biosynthesis
Frequency
P-score
0
100
200
300
400
500
600
0.0
0.2
0.4
0.6
0.8
1.0

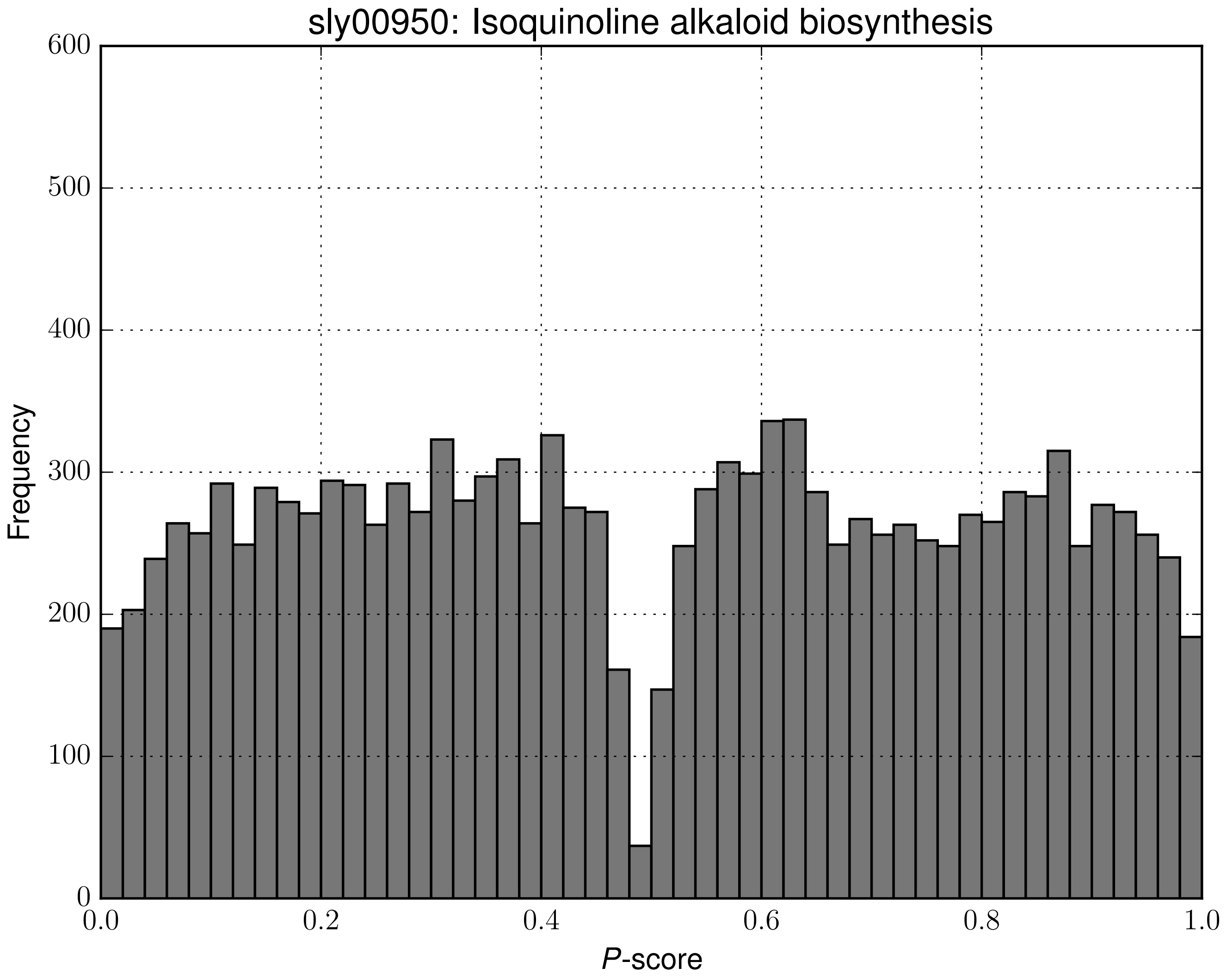
sly00950: Isoquinoline alkaloid biosynthesis
Frequency
P-score
0
100
200
300
400
500
600
0.0
0.2
0.4
0.6
0.8
1.0

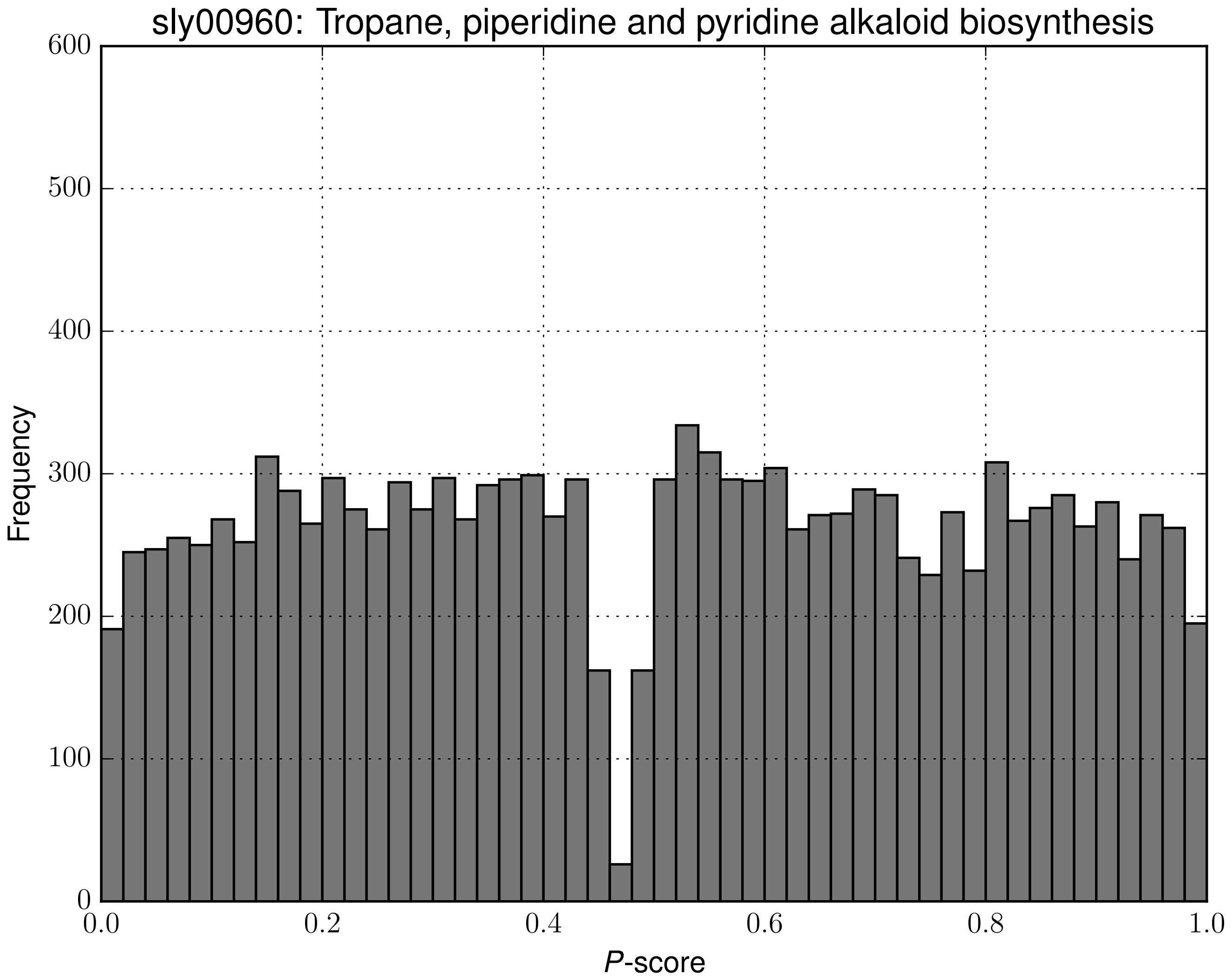
sly00960: Tropane, piperidine and pyridine alkaloid biosynthesis
600
500
400
300
200
100
0
Frequency
0.0
0.2
0.4
0.6
0.8
1.0
P-score

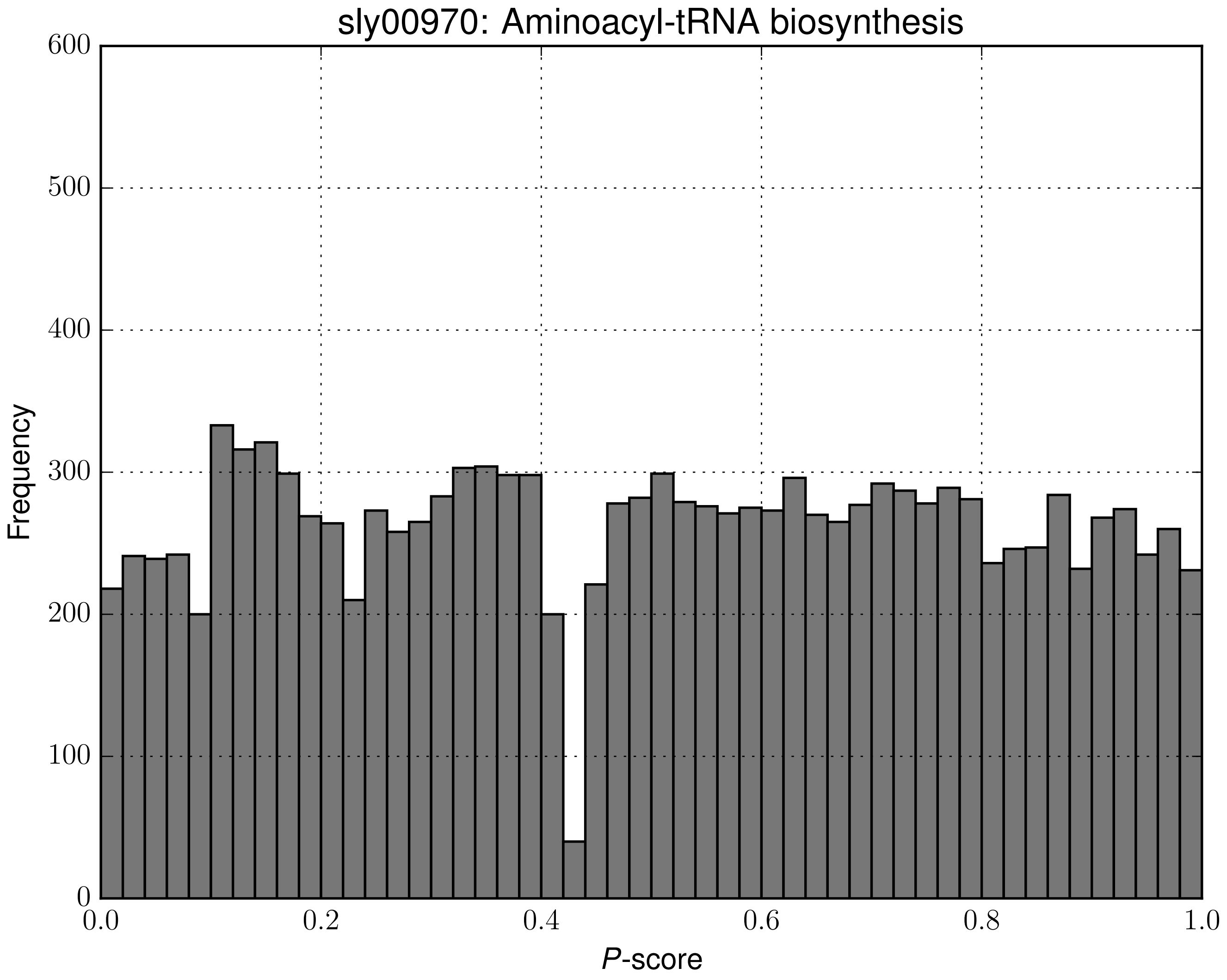
sly00970: Aminoacyl-tRNA biosynthesis
Frequency
P-score
0
100
200
300
400
500
600
0.0
0.2
0.4
0.6
0.8
1.0

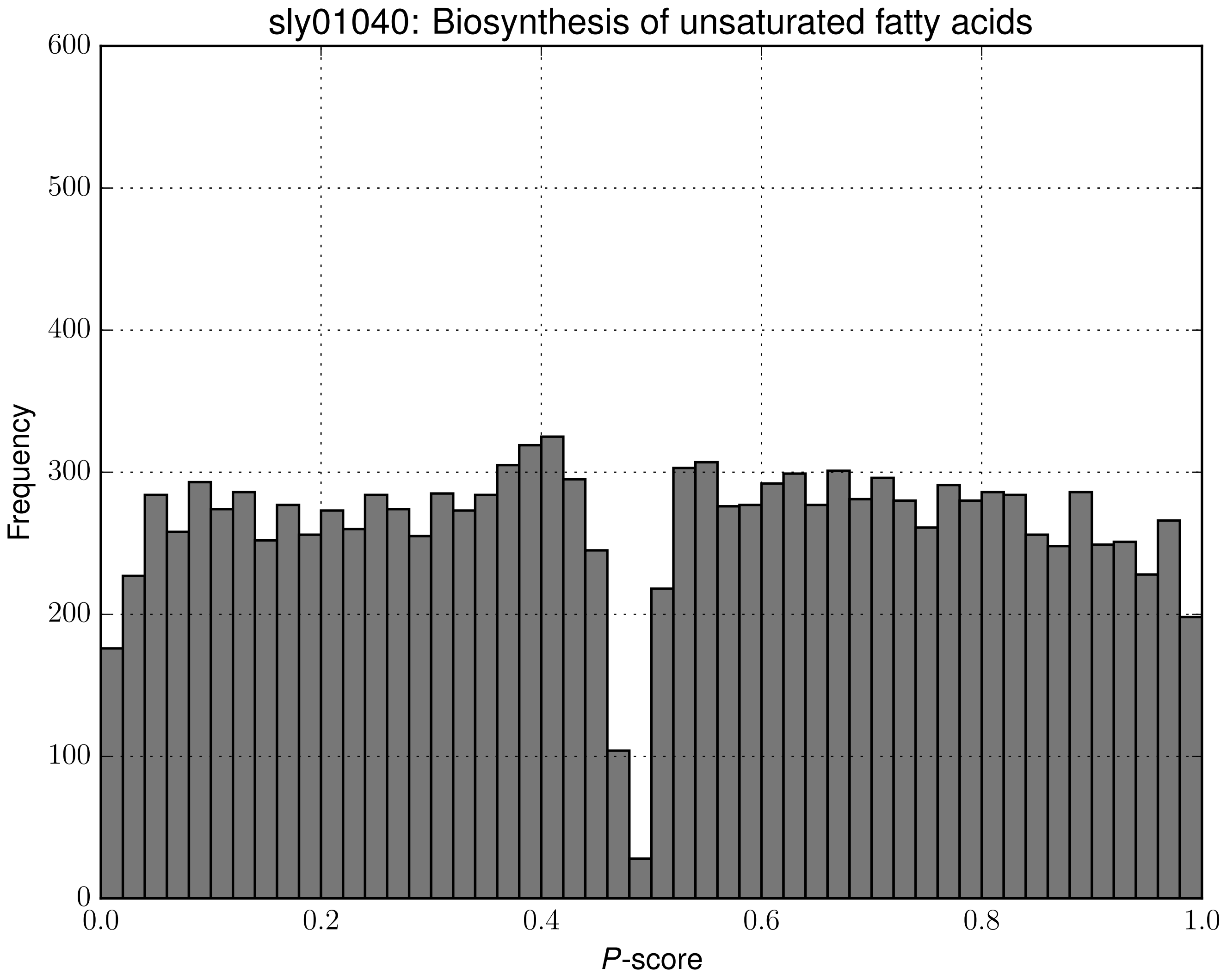
sly01040: Biosynthesis of unsaturated fatty acids
Frequency
P-score
0
100
200
300
400
500
600
0.0
0.2
0.4
0.6
0.8
1.0

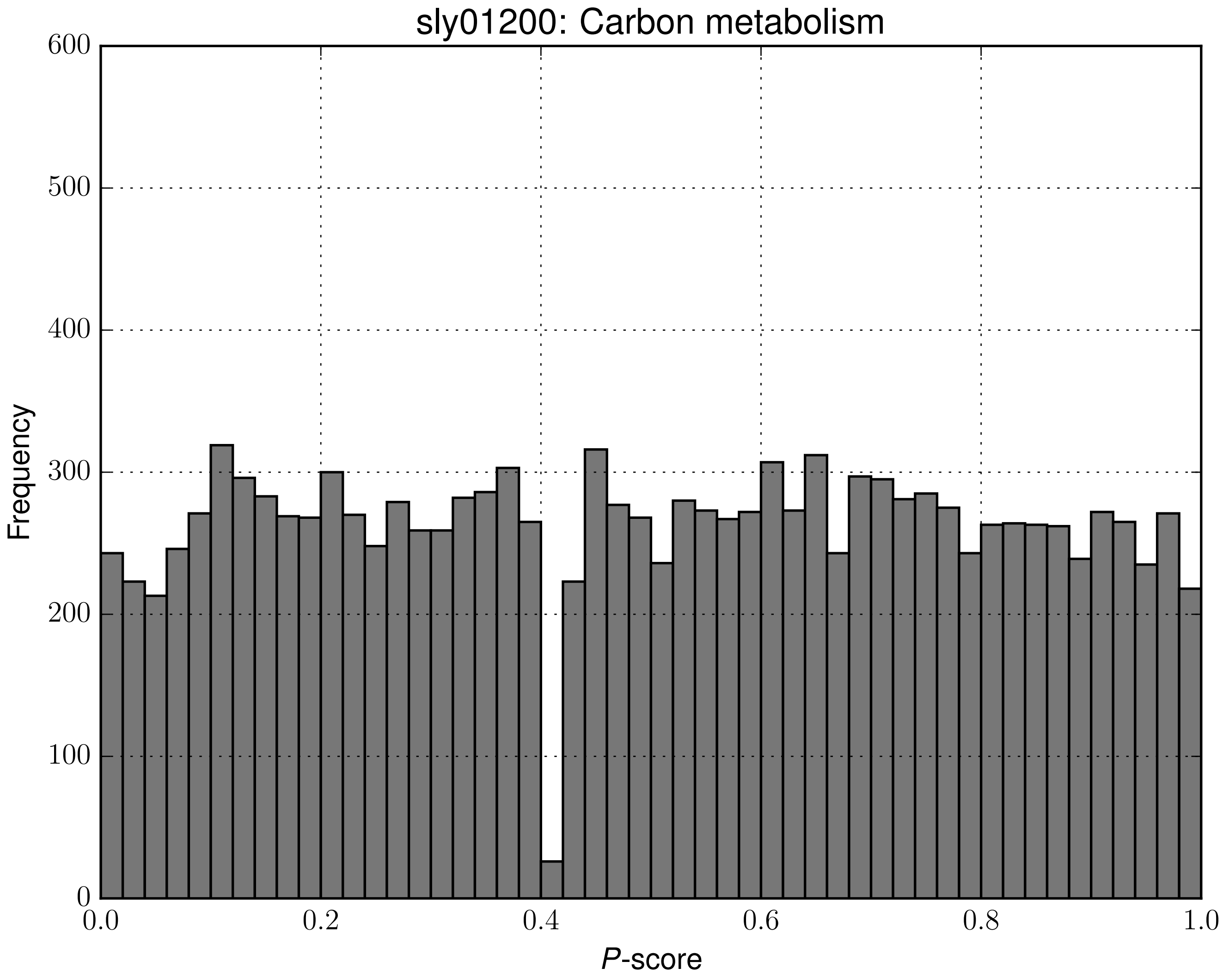
sly01200: Carbon metabolism
Frequency
P-score
0
100
200
300
400
500
600
0.0
0.2
0.4
0.6
0.8
1.0

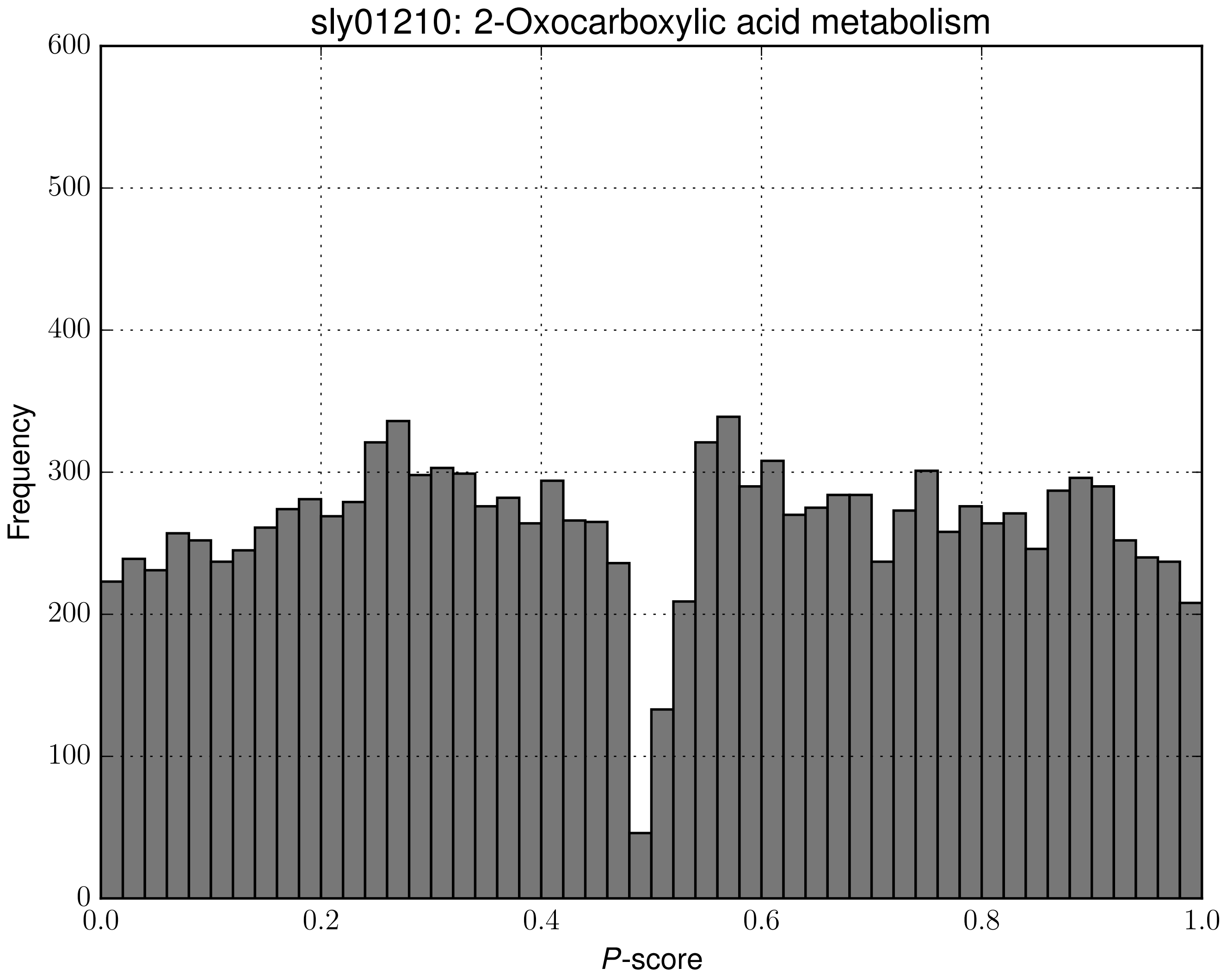
sly01210: 2-Oxocarboxylic acid metabolism
Frequency
600
500
400
300
200
100
0
0.0
0.2
0.4
0.6
0.8
1.0
P-score

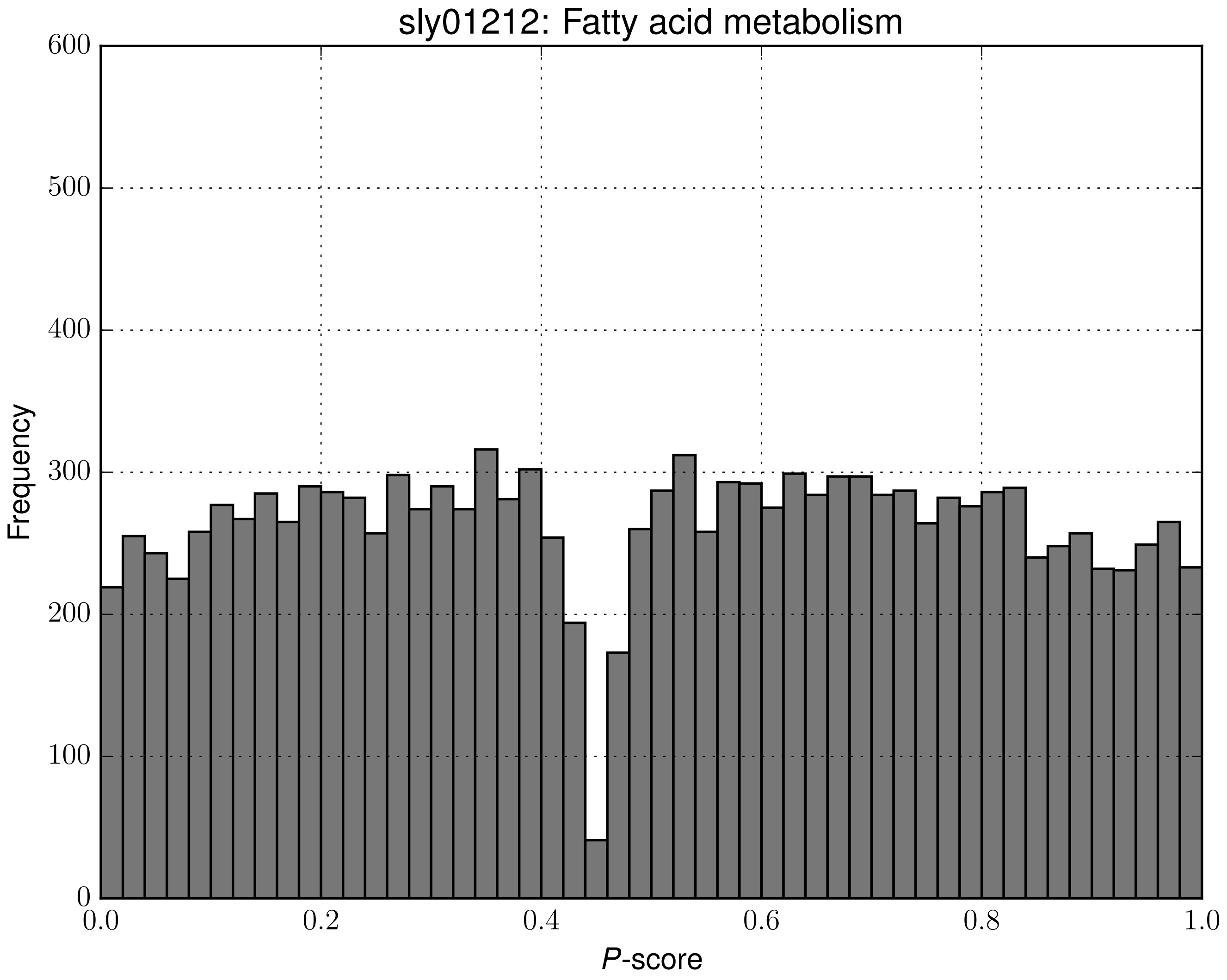
sly01212: Fatty acid metabolism
Frequency
600
500
400
300
200
100
0
0.0
0.2
0.4
0.6
0.8
1.0
P-score

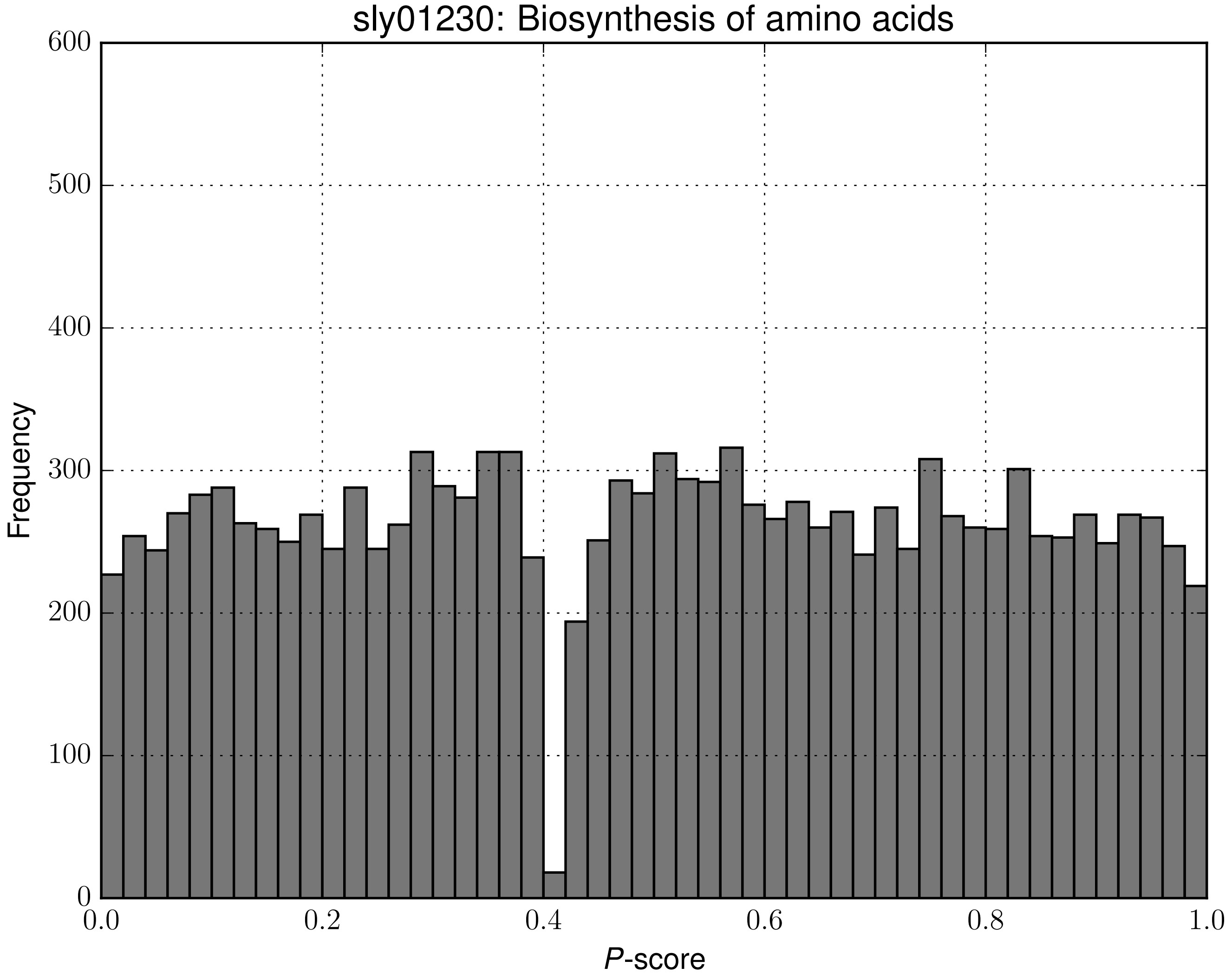
sly01230: Biosynthesis of amino acids
Frequency
P-score
0
100
200
300
400
500
600
0.0
0.2
0.4
0.6
0.8
1.0

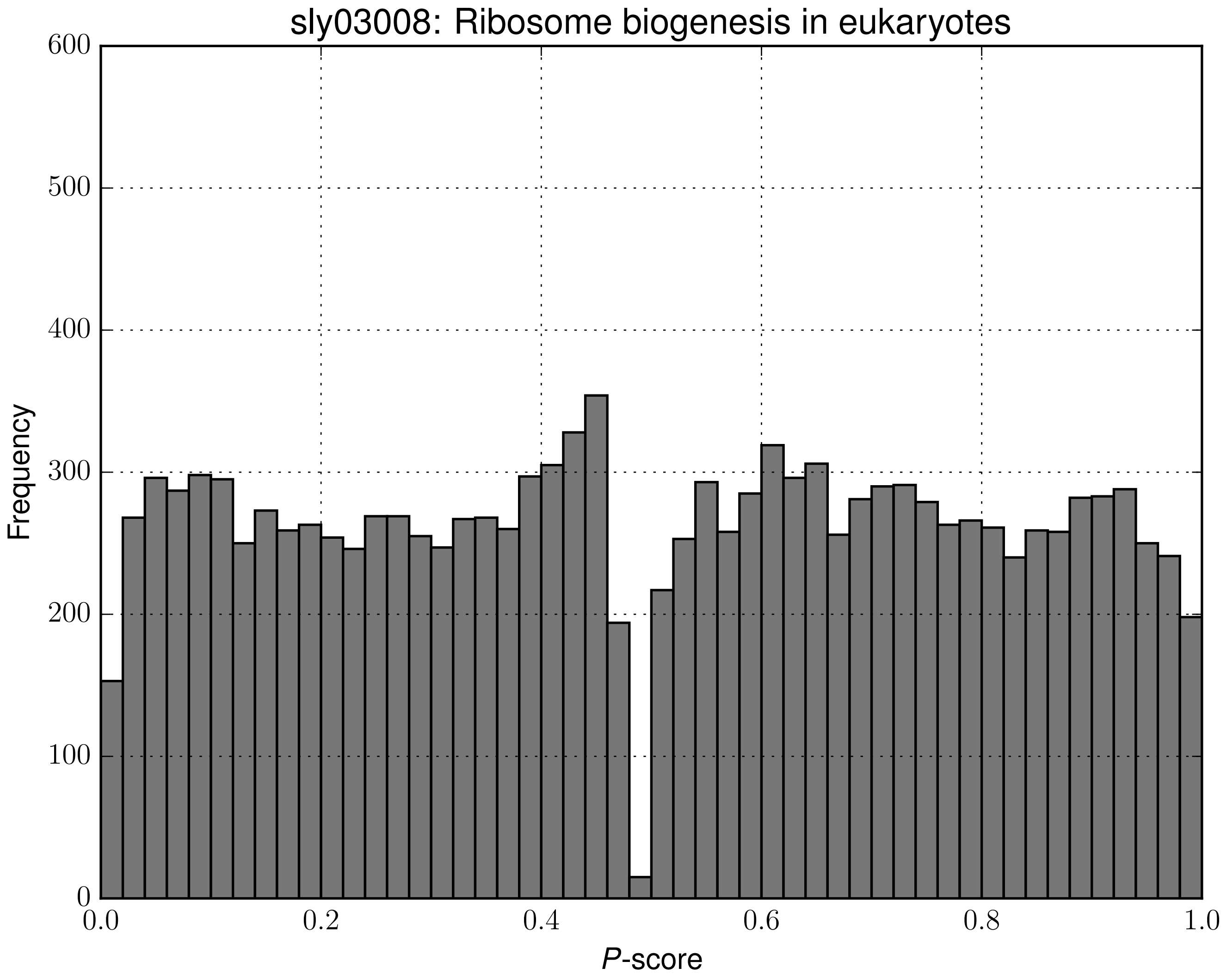
sly03008: Ribosome biogenesis in eukaryotes
Frequency
P-score
0
100
200
300
400
500
600
0.0
0.2
0.4
0.6
0.8
1.0

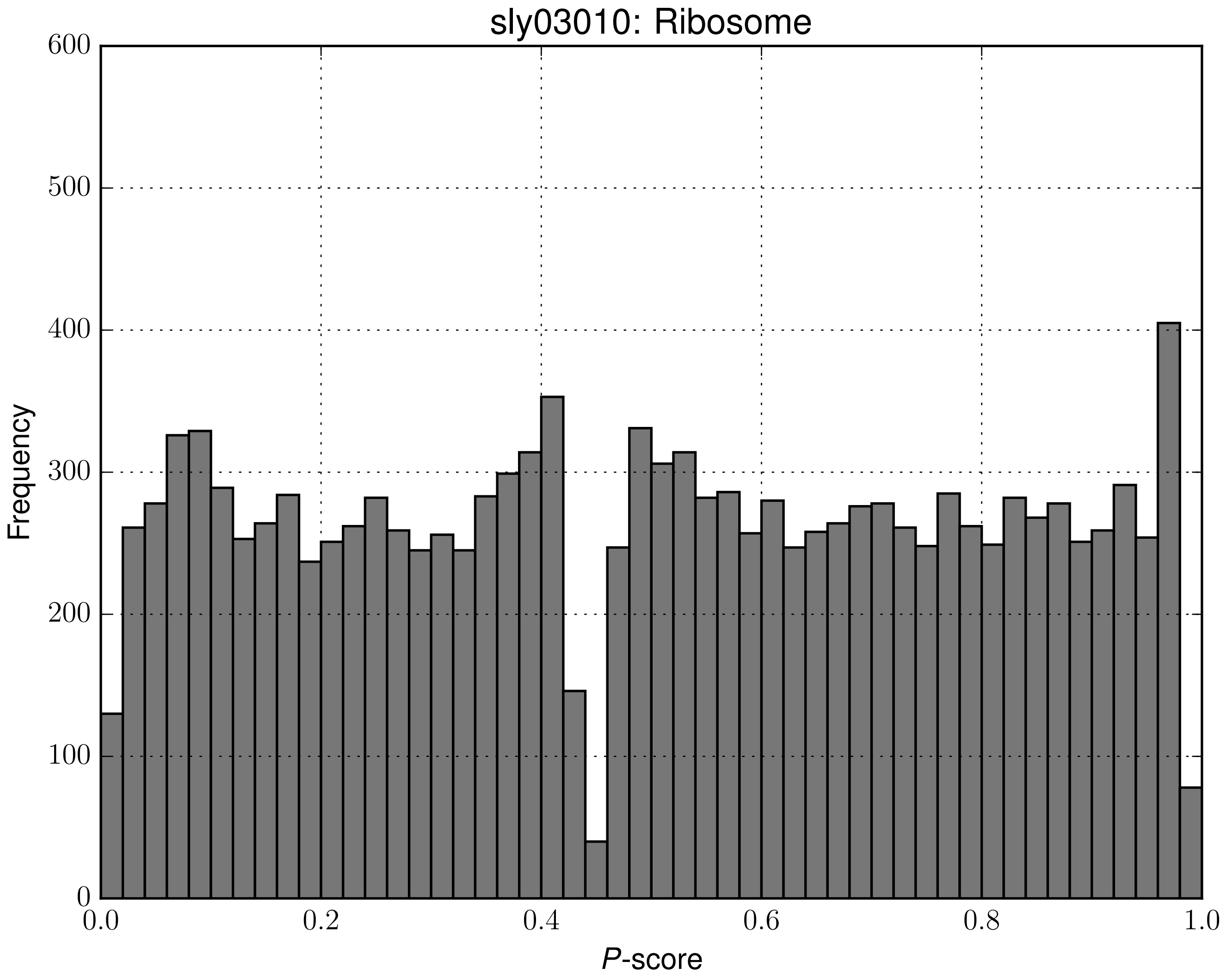

sly03010: Ribosome
Frequency
P-score
0
100
200
300
400
500
600
0.0
0.2
0.4
0.6
0.8
1.0

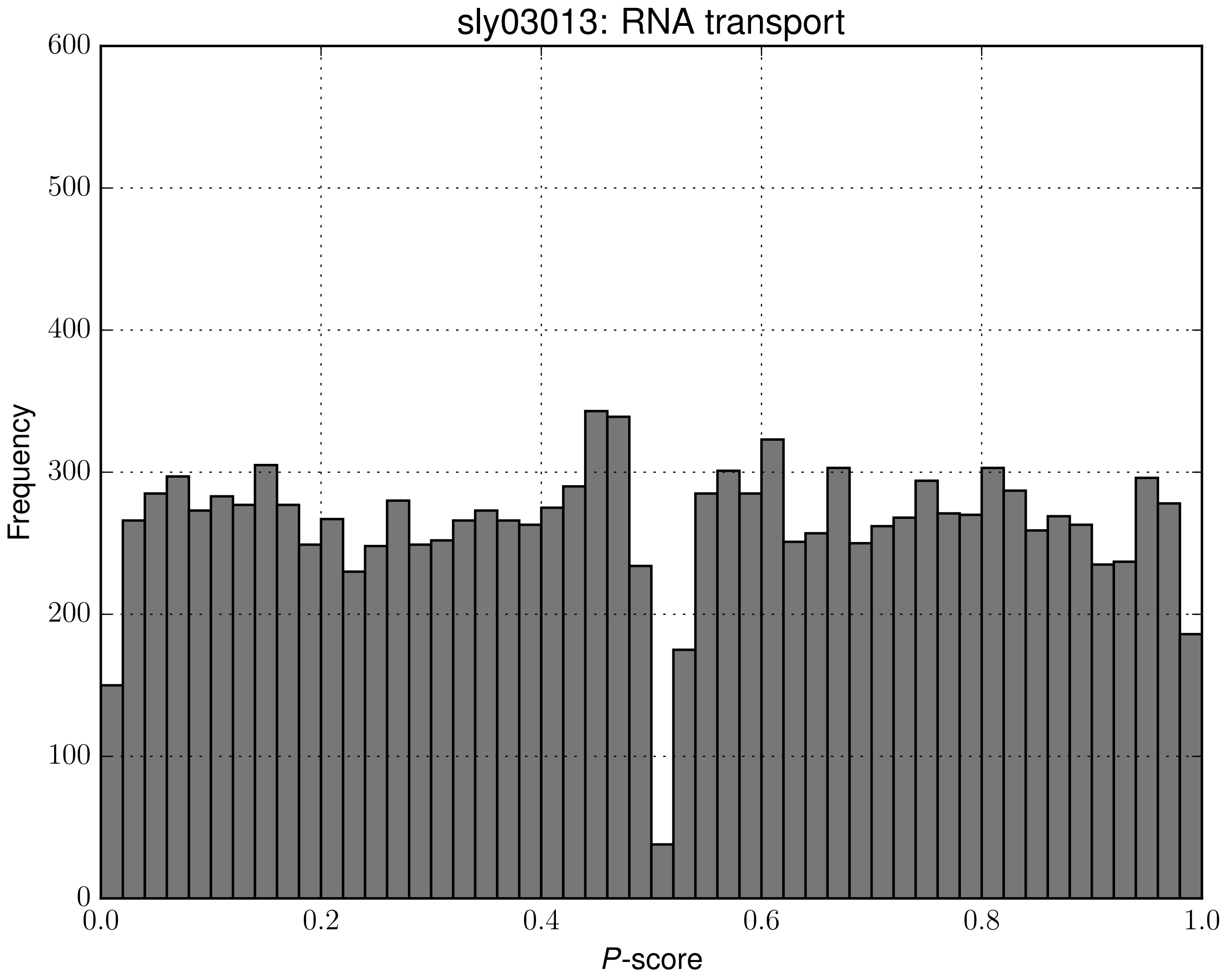
sly03013: RNA transport
Frequency
P-score
0
100
200
300
400
500
600
0.0
0.2
0.4
0.6
0.8
1.0

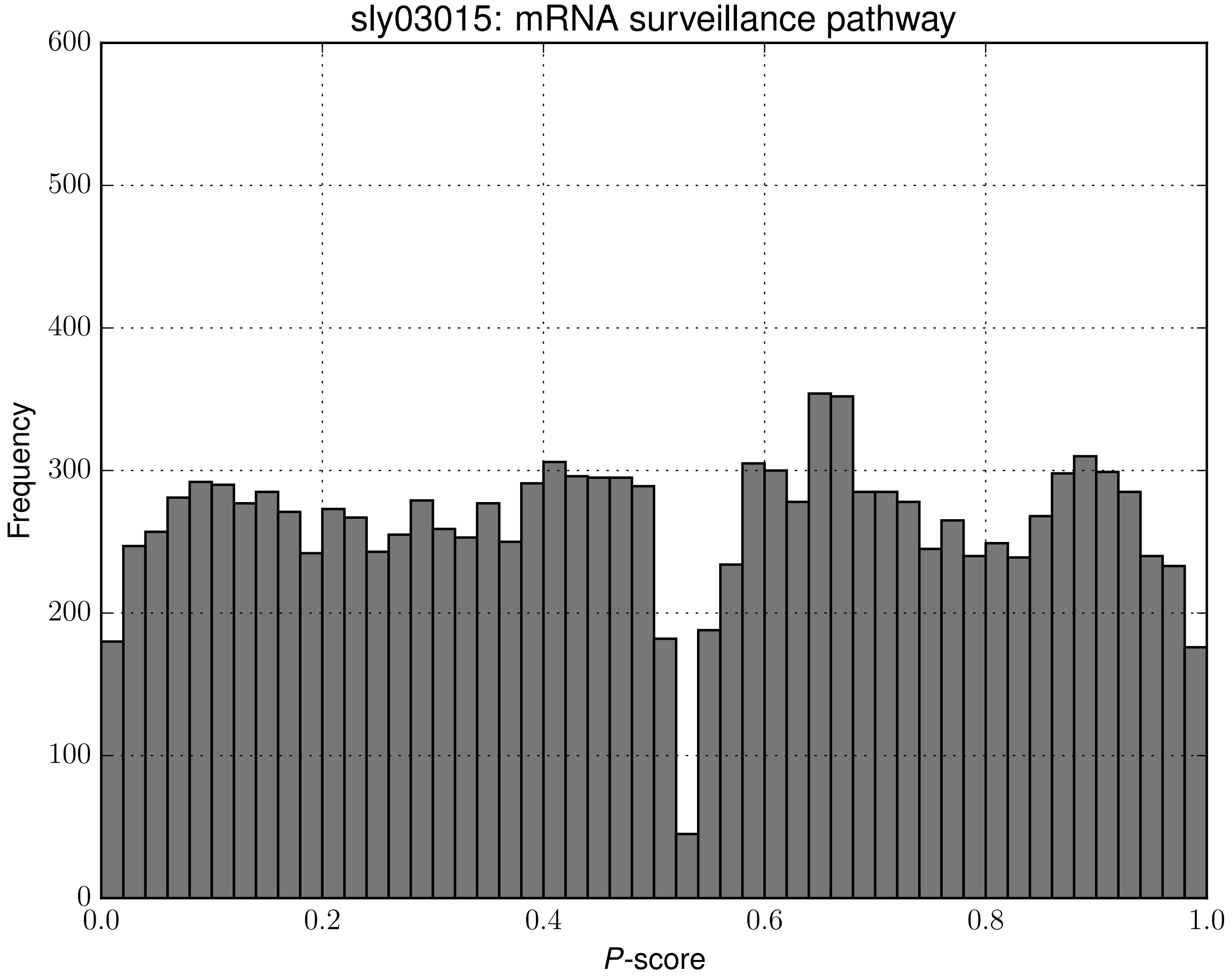
sly03015: mRNA surveillance pathway
Frequency
600
500
400
300
200
100
0
0.0
0.2
0.4
0.6
0.8
1.0
P-score

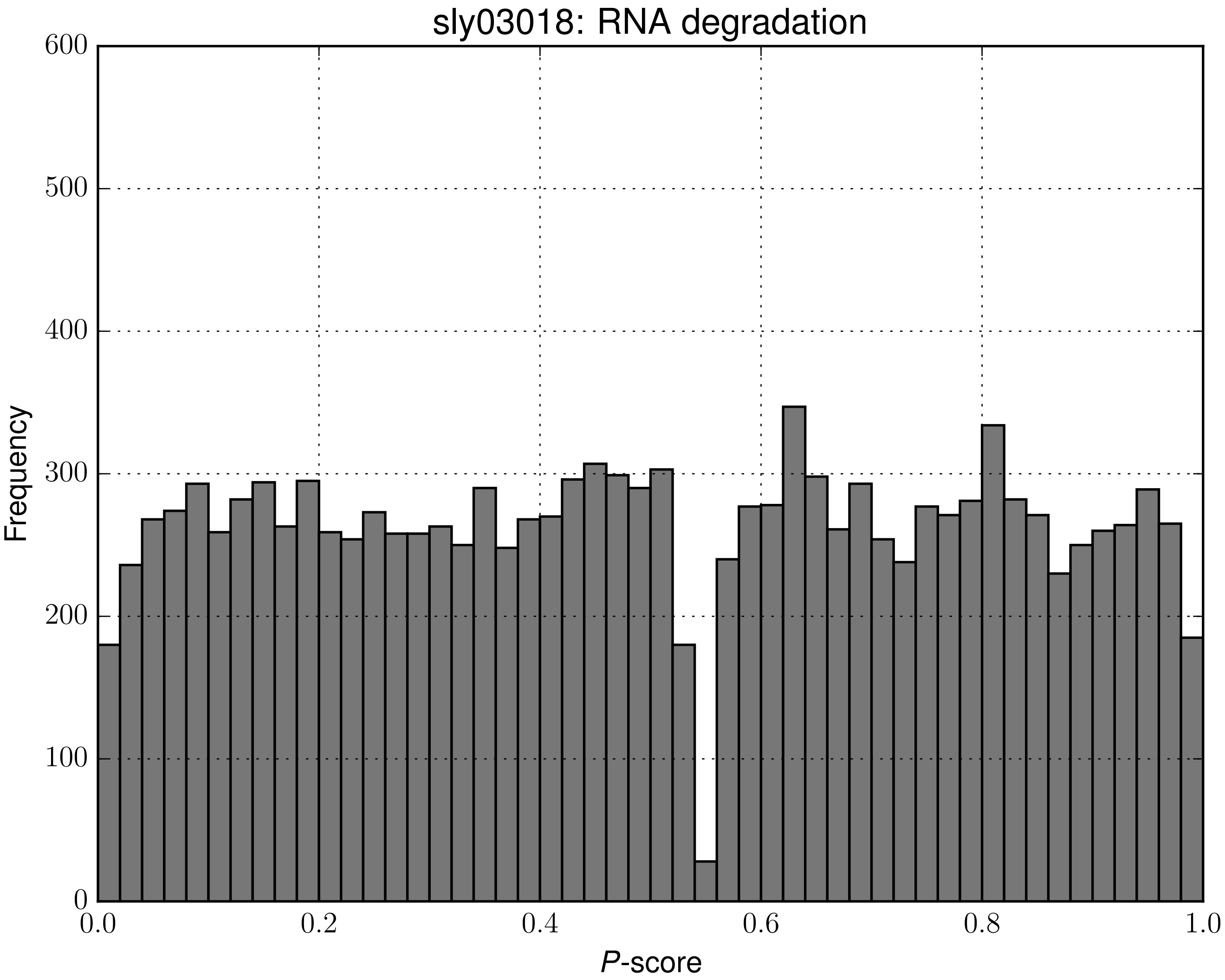
sly03018: RNA degradation
Frequency
0
100
200
300
400
500
600
0.0
0.2
0.4
0.6
0.8
1.0
P-score

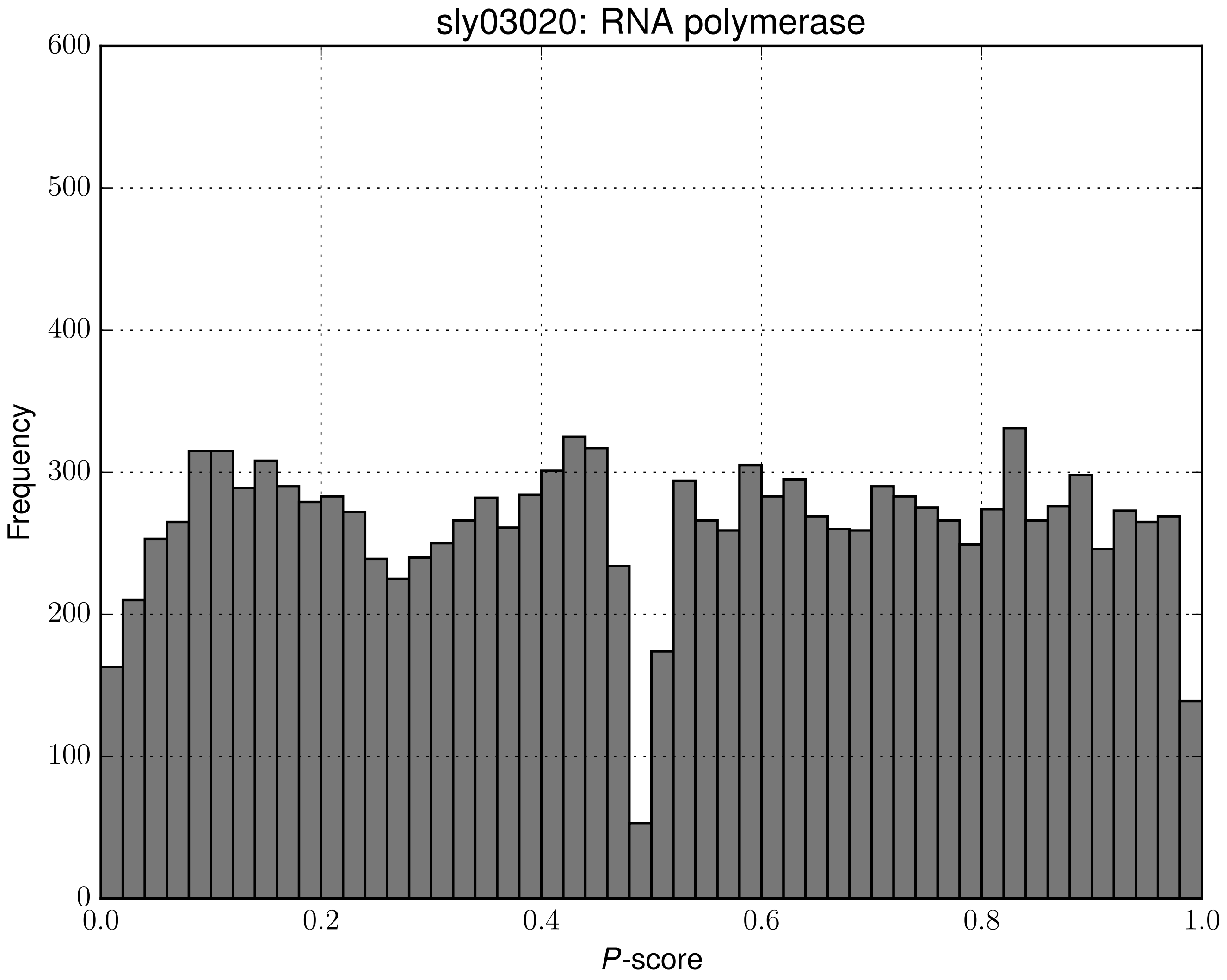

sly03020: RNA polymerase
Frequency
P-score
0
100
200
300
400
500
600
0.0
0.2
0.4
0.6
0.8
1.0

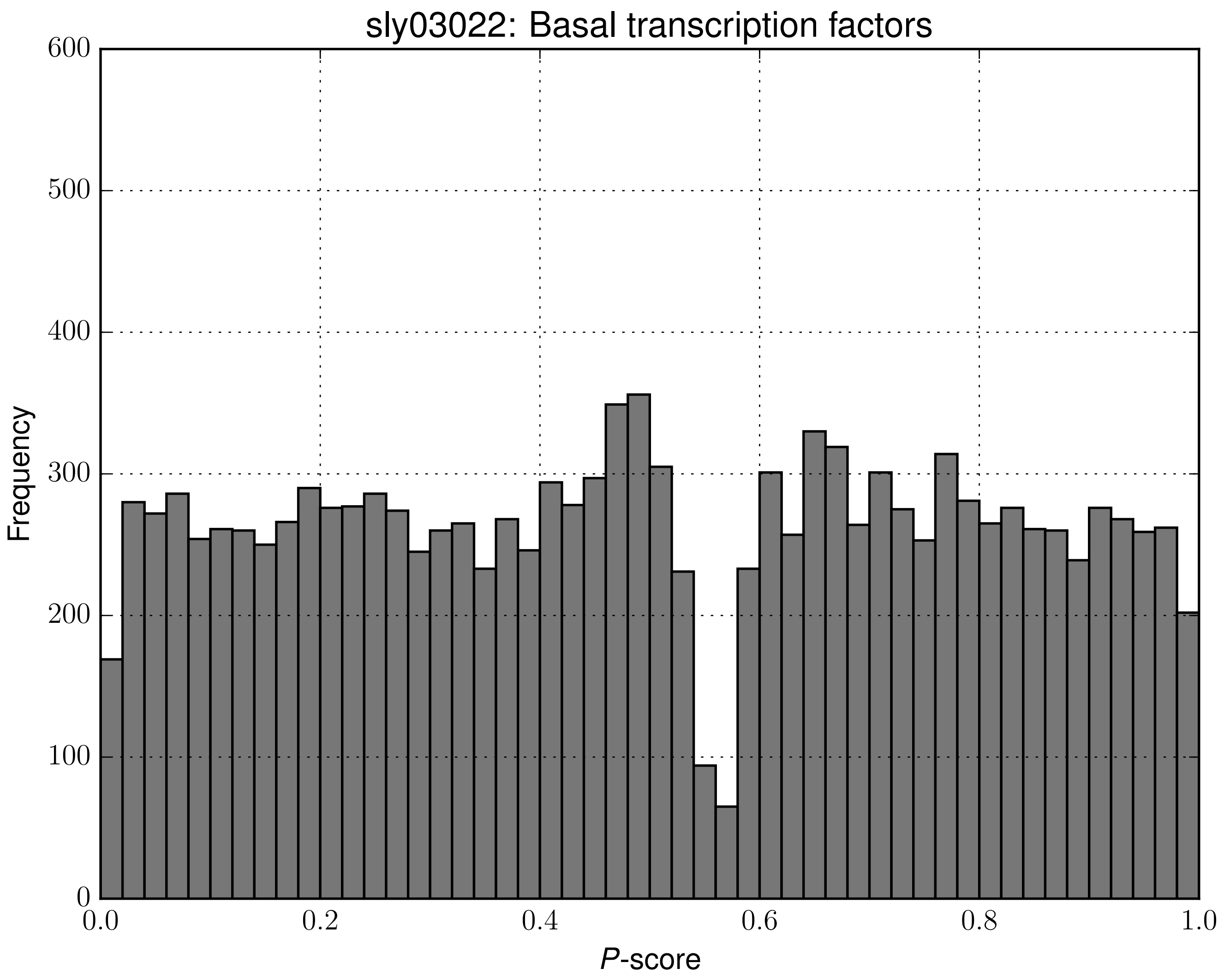
sly03022: Basal transcription factors
Frequency
P-score
0
100
200
300
400
500
600
0.0
0.2
0.4
0.6
0.8
1.0

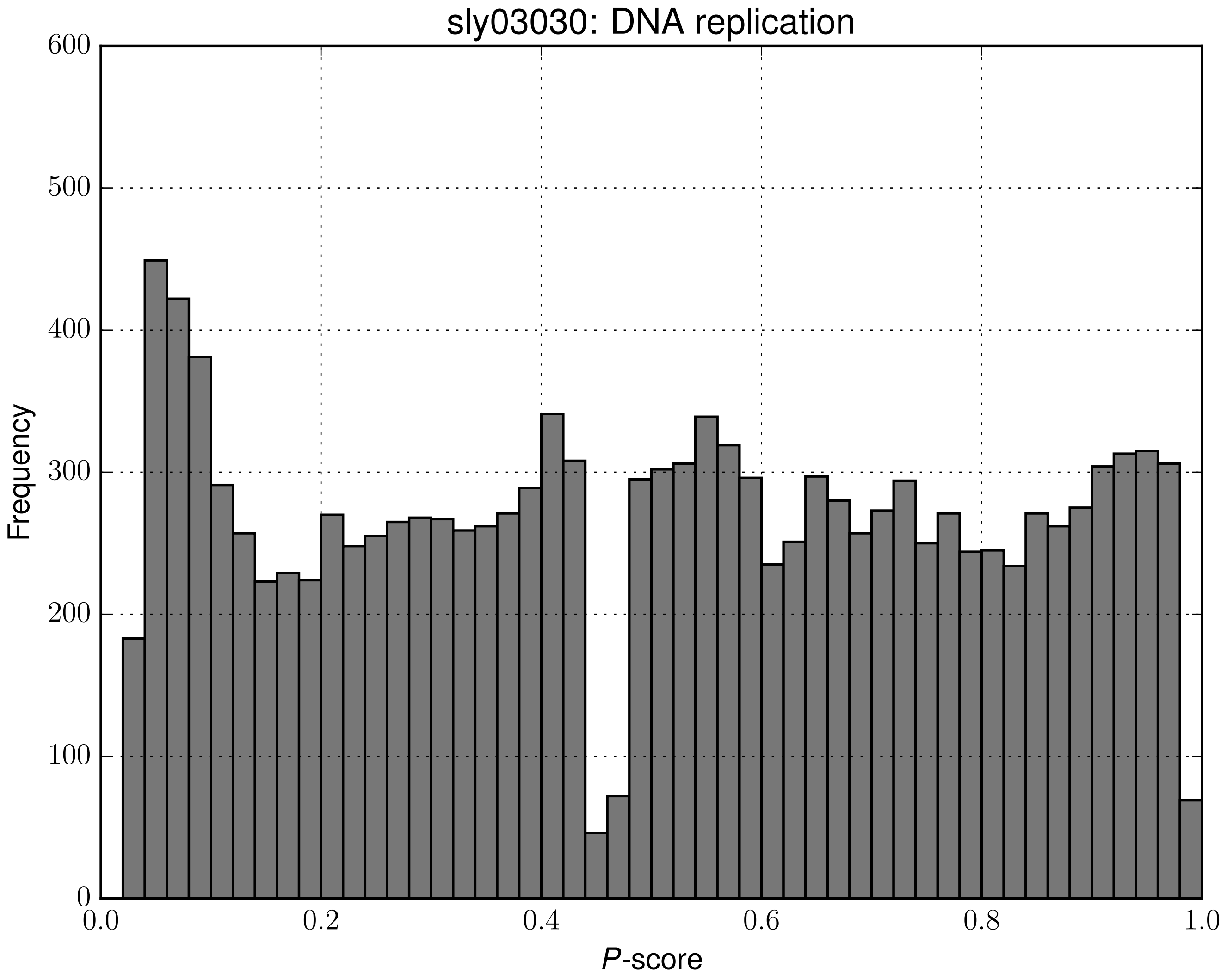

sly03030: DNA replication
Frequency
P-score
0
100
200
300
400
500
600
0.0
0.2
0.4
0.6
0.8
1.0

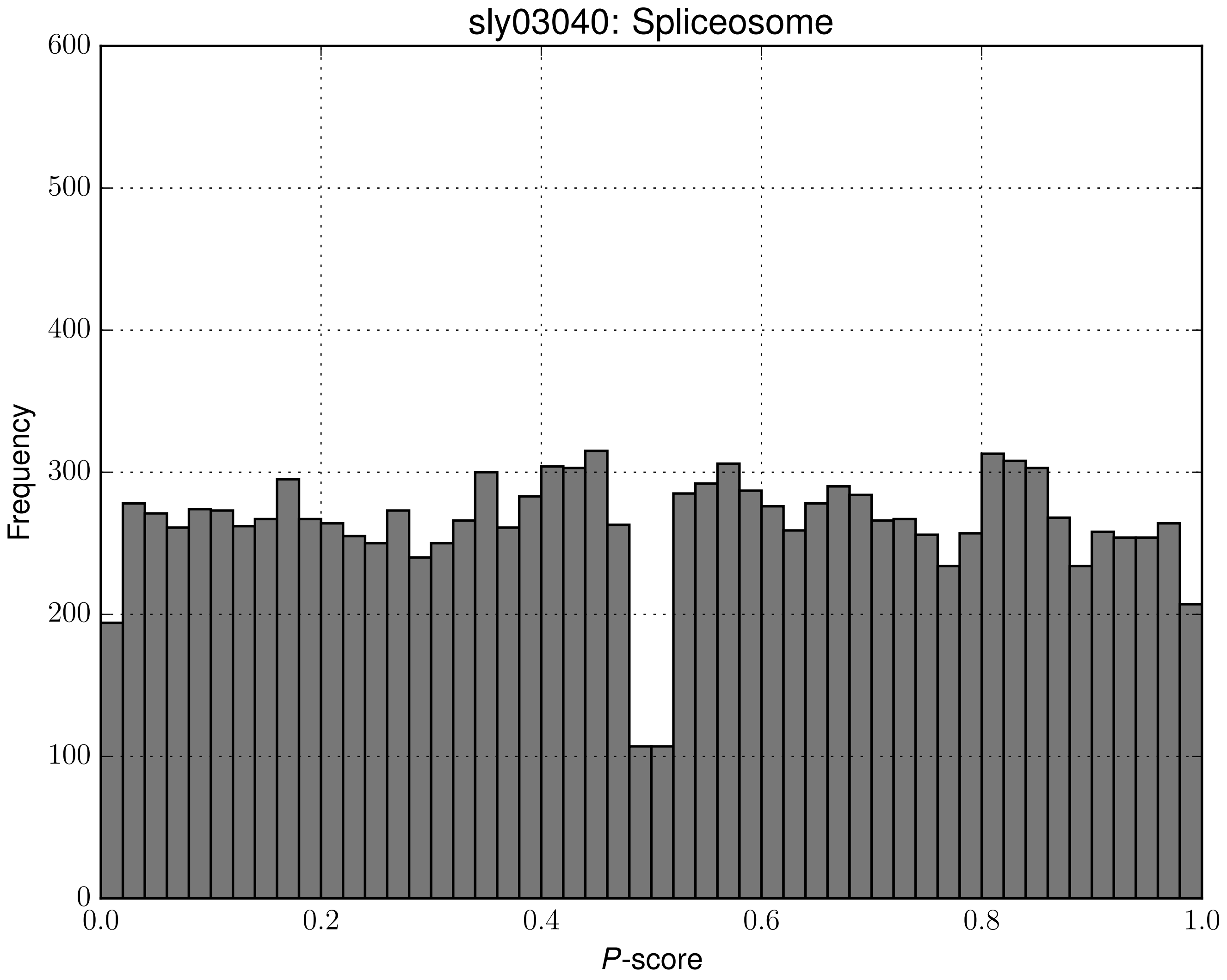
sly03040: Spliceosome
Frequency
P-score
0
100
200
300
400
500
600
0.0
0.2
0.4
0.6
0.8
1.0

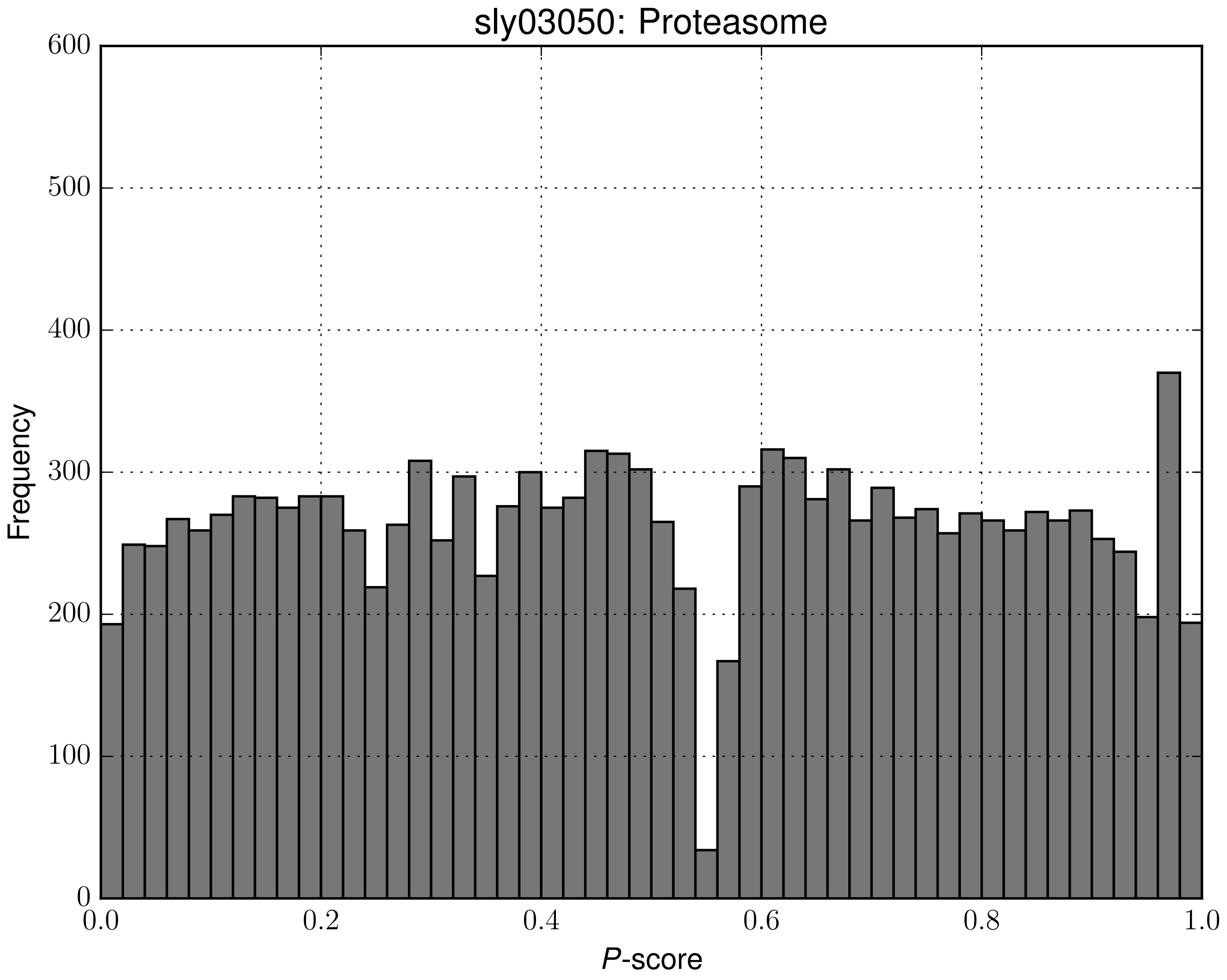
sly03050: Proteasome
Frequency
P-score
0
100
200
300
400
500
600
0.0
0.2
0.4
0.6
0.8
1.0

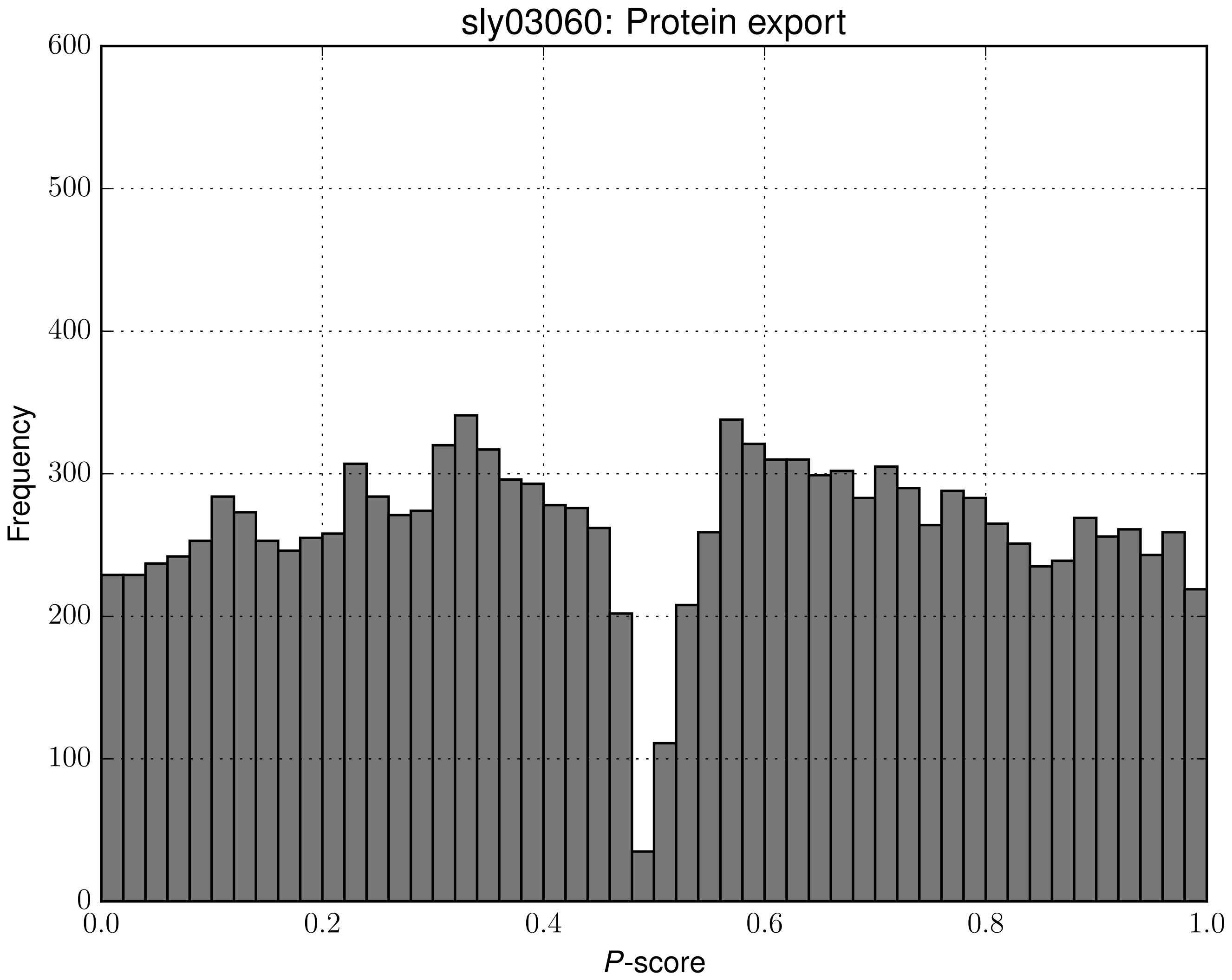
sly03060: Protein export
Frequency
P-score
0
100
200
300
400
500
600
0.0
0.2
0.4
0.6
0.8
1.0

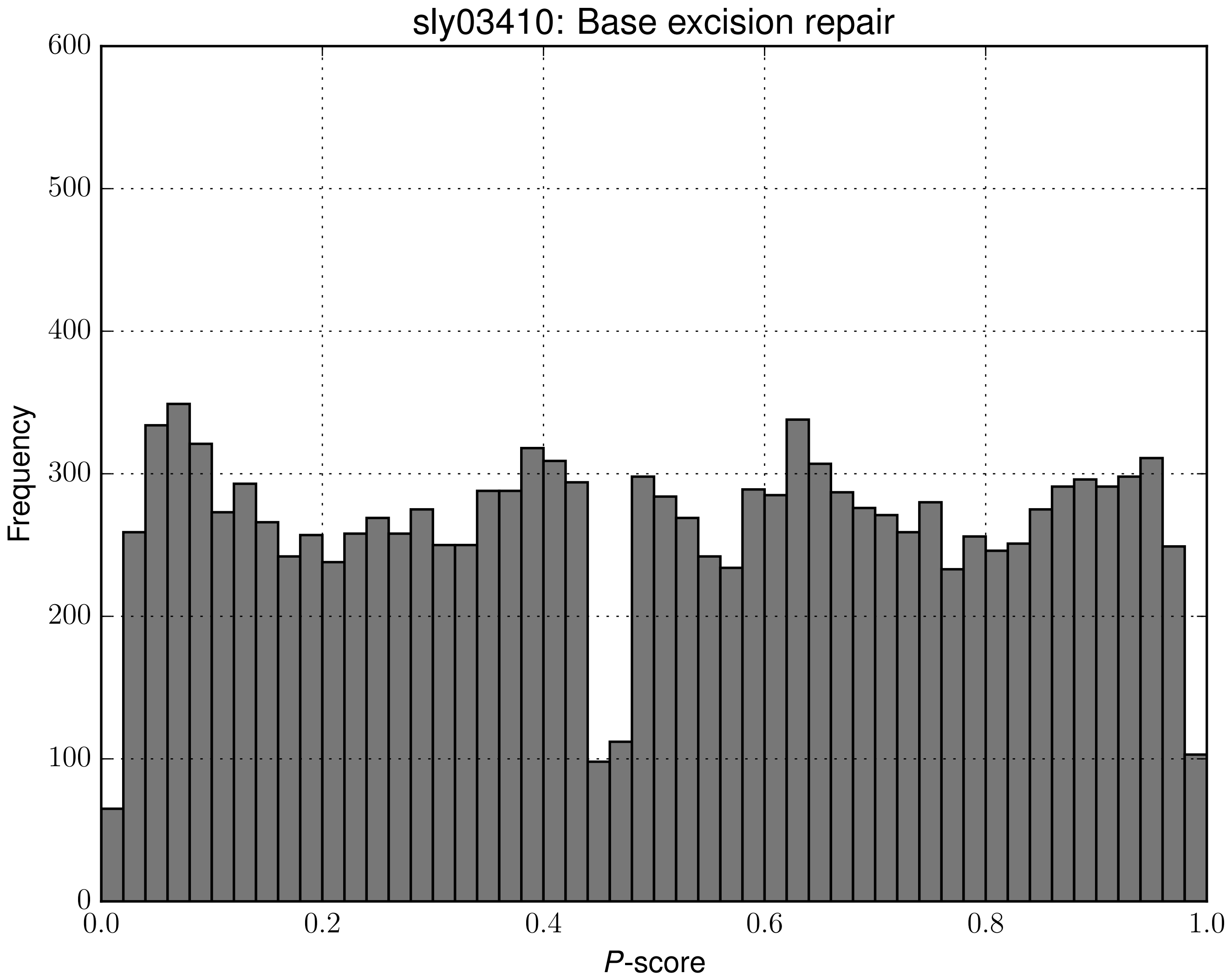

sly03410: Base excision repair
Frequency
P-score
0
100
200
300
400
500
600
0.0
0.2
0.4
0.6
0.8
1.0

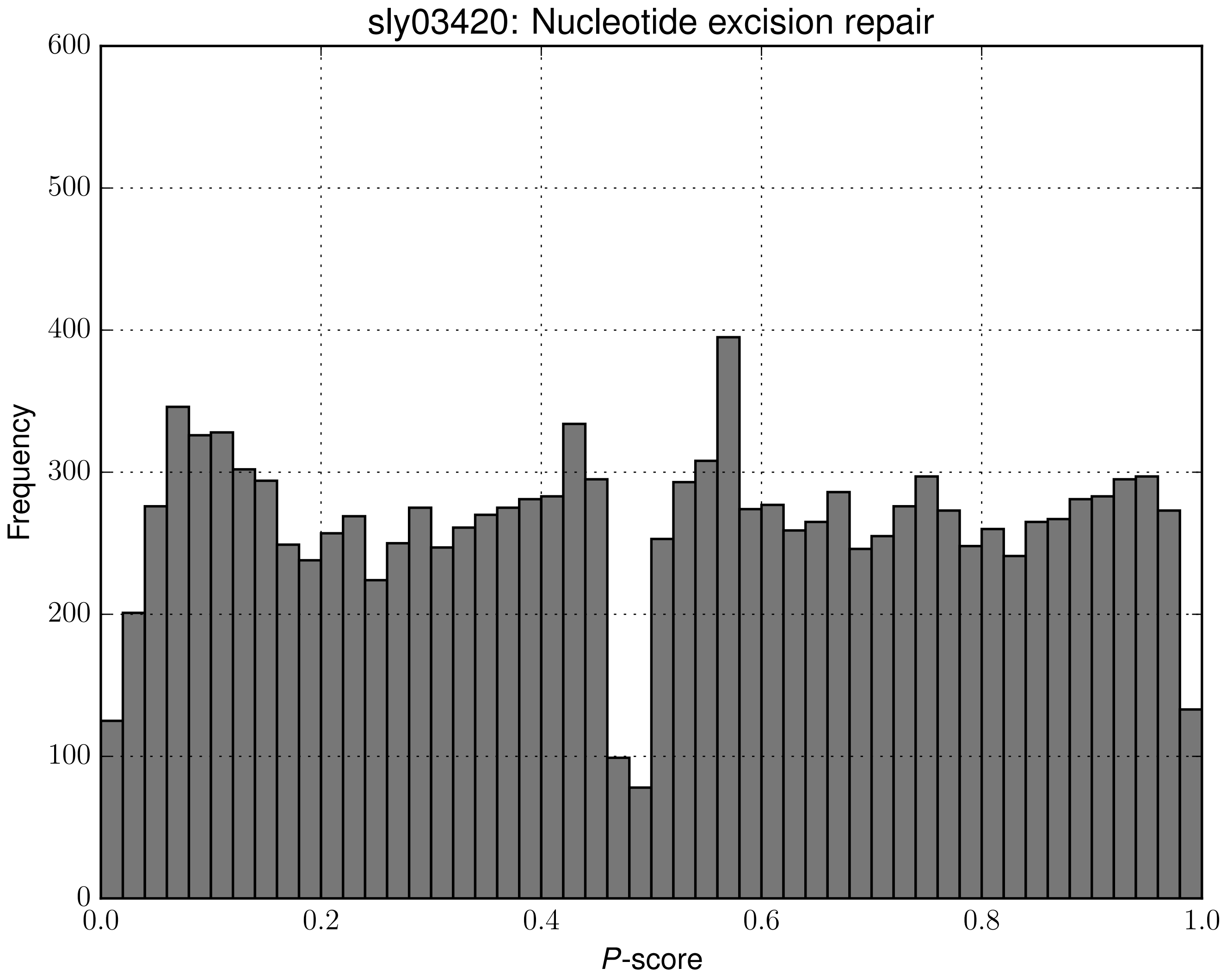
sly03420: Nucleotide excision repair
Frequency
P-score
0
100
200
300
400
500
600
0.0
0.2
0.4
0.6
0.8
1.0

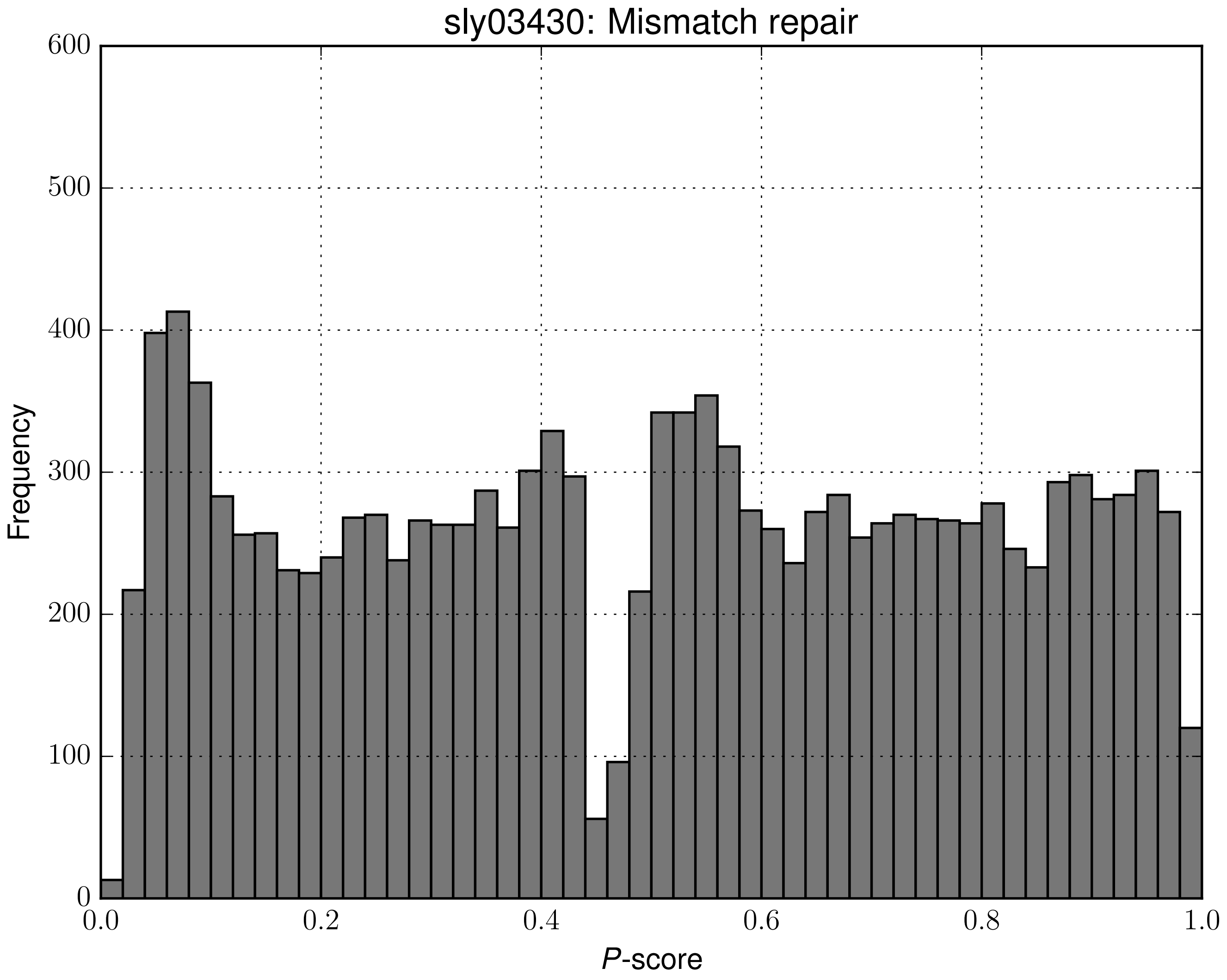
sly03430: Mismatch repair
Frequency
P-score
0
100
200
300
400
500
600
0.0
0.2
0.4
0.6
0.8
1.0

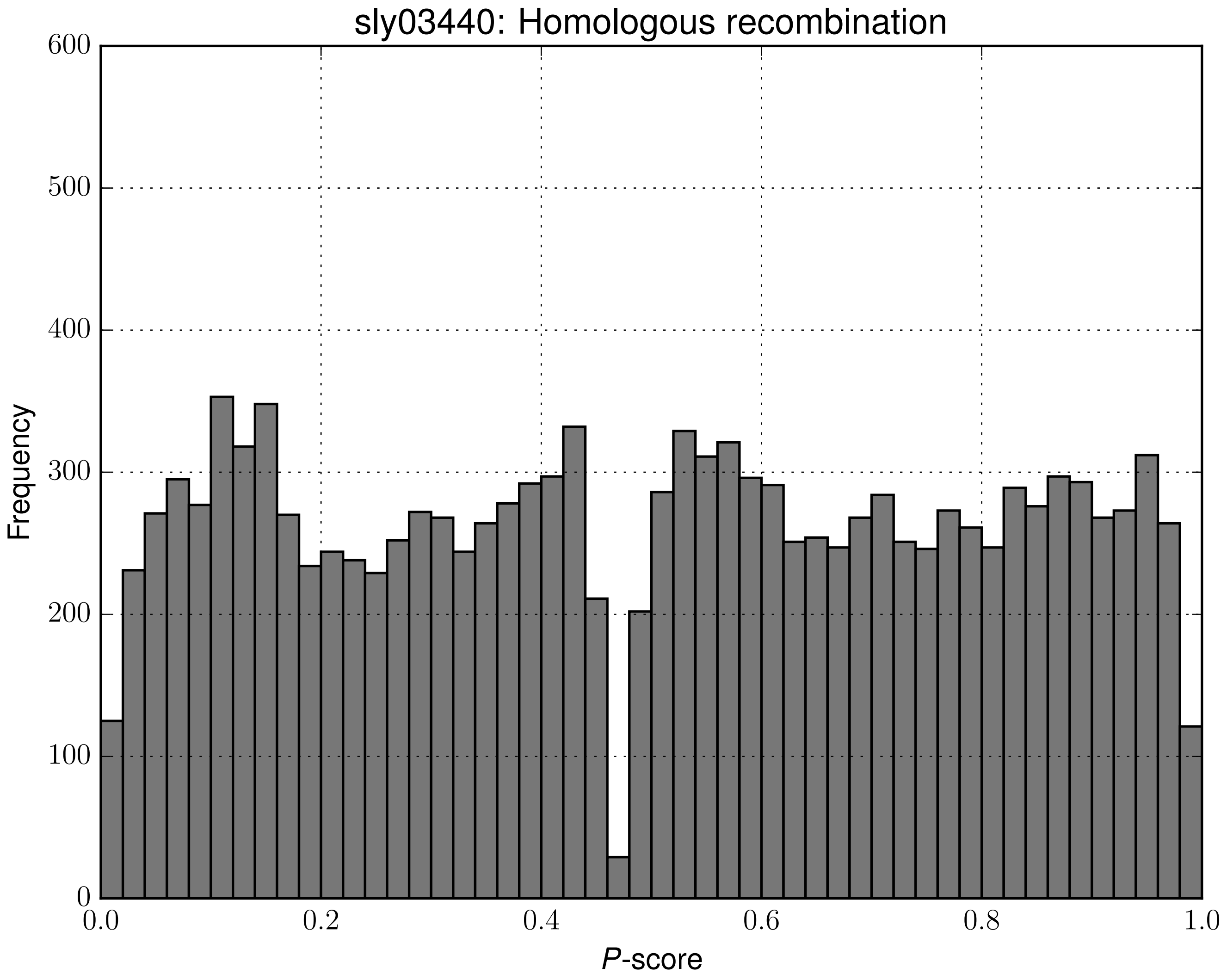
sly03440: Homologous recombination
Frequency
P-score
0
100
200
300
400
500
600
0.0
0.2
0.4
0.6
0.8
1.0

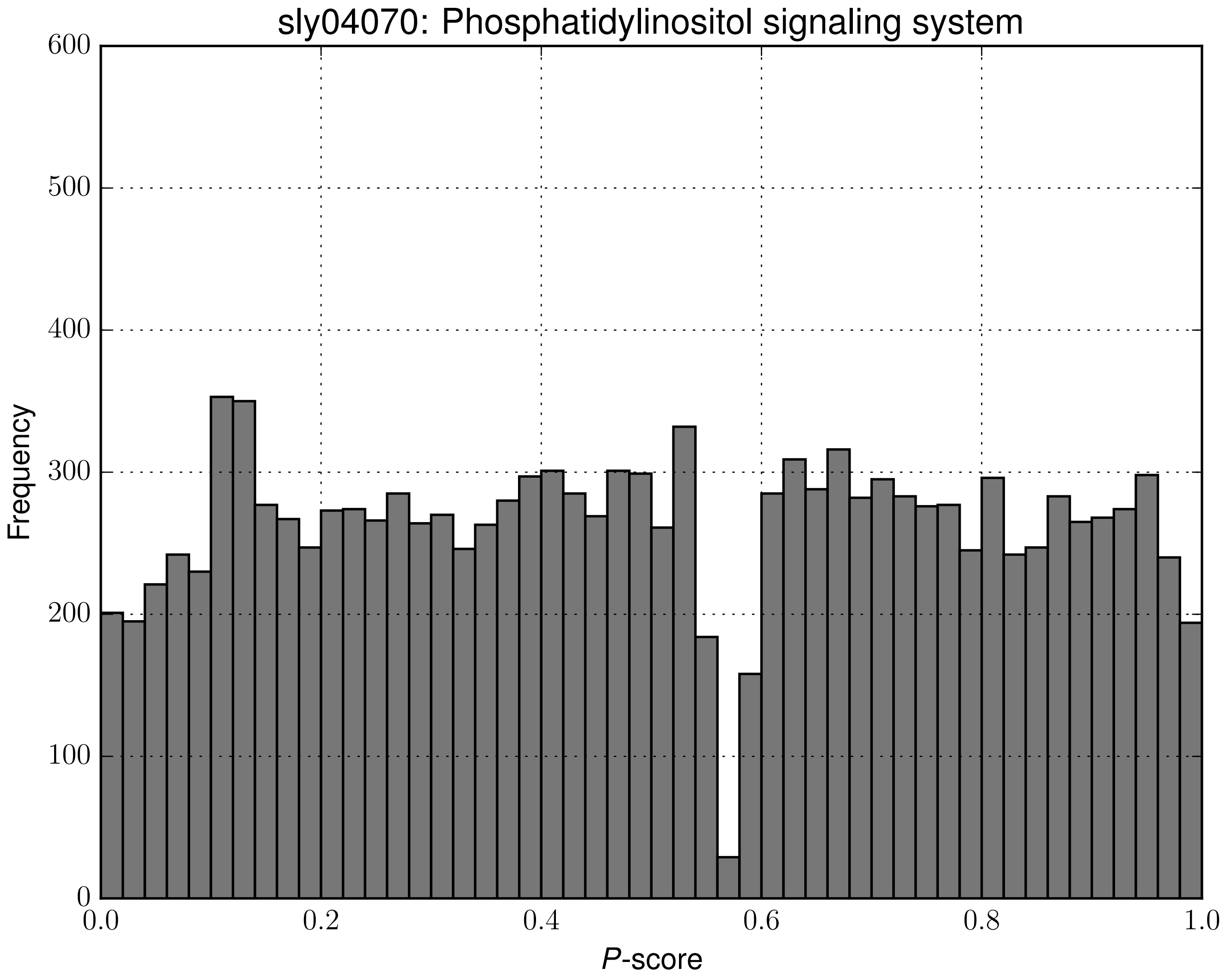

sly04070: Phosphatidylinositol signaling system
Frequency
P-score
0
100
200
300
400
500
600
0.0
0.2
0.4
0.6
0.8
1.0

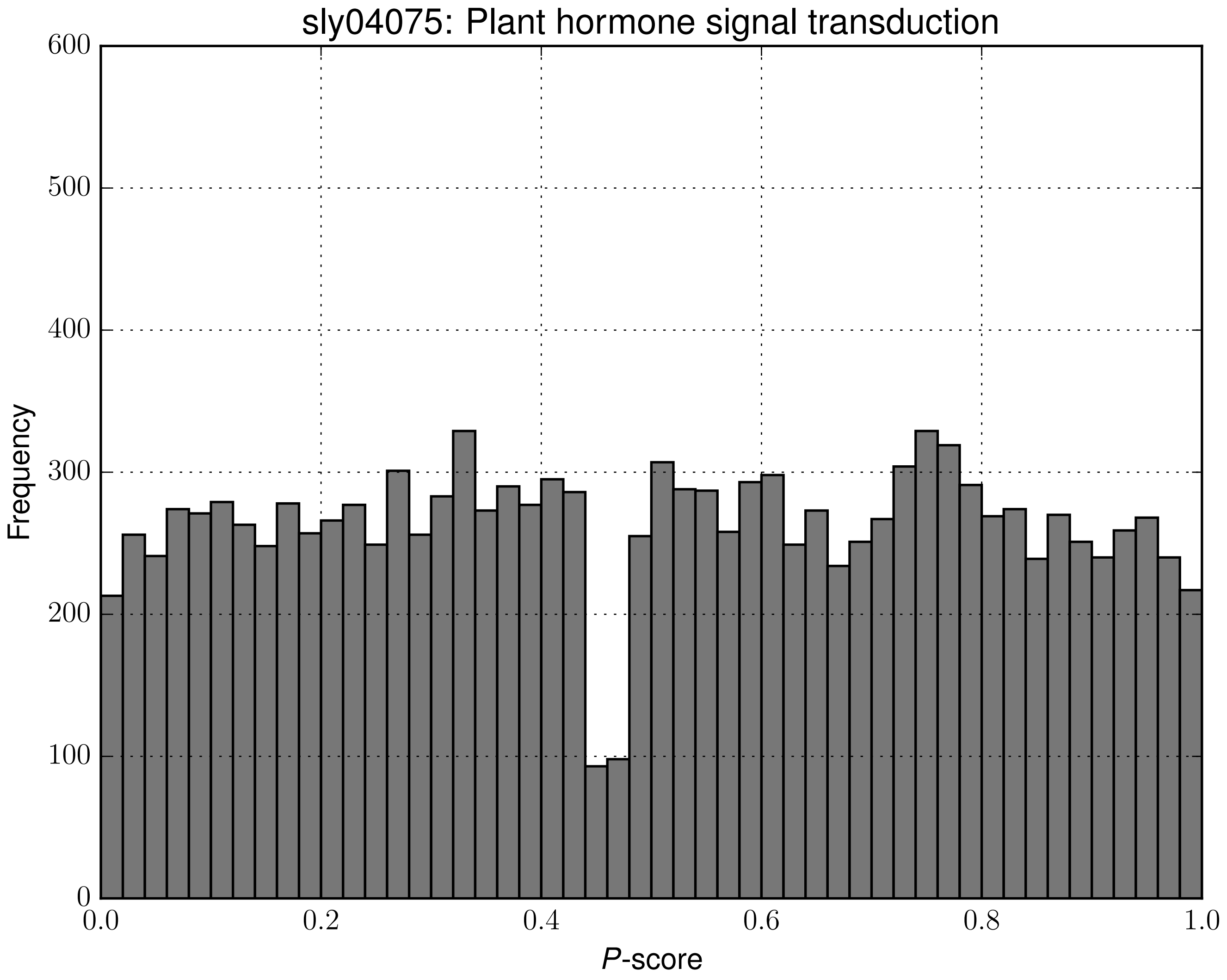
sly04075: Plant hormone signal transduction
Frequency
P-score
0
100
200
300
400
500
600
0.0
0.2
0.4
0.6
0.8
1.0

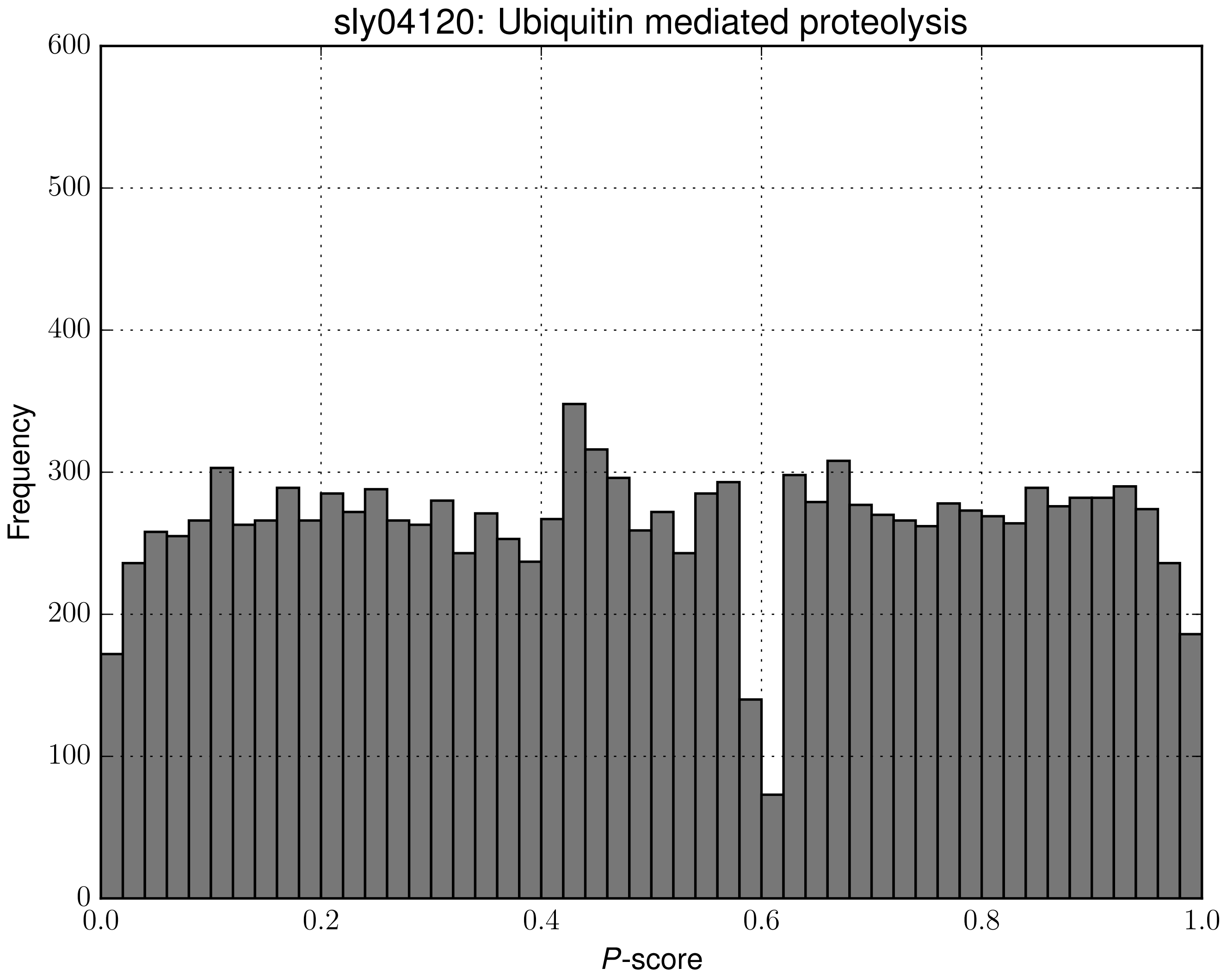

sly04120: Ubiquitin mediated proteolysis
Frequency
0
100
200
300
400
500
600
0.0
0.2
0.4
0.6
0.8
1.0
P-score

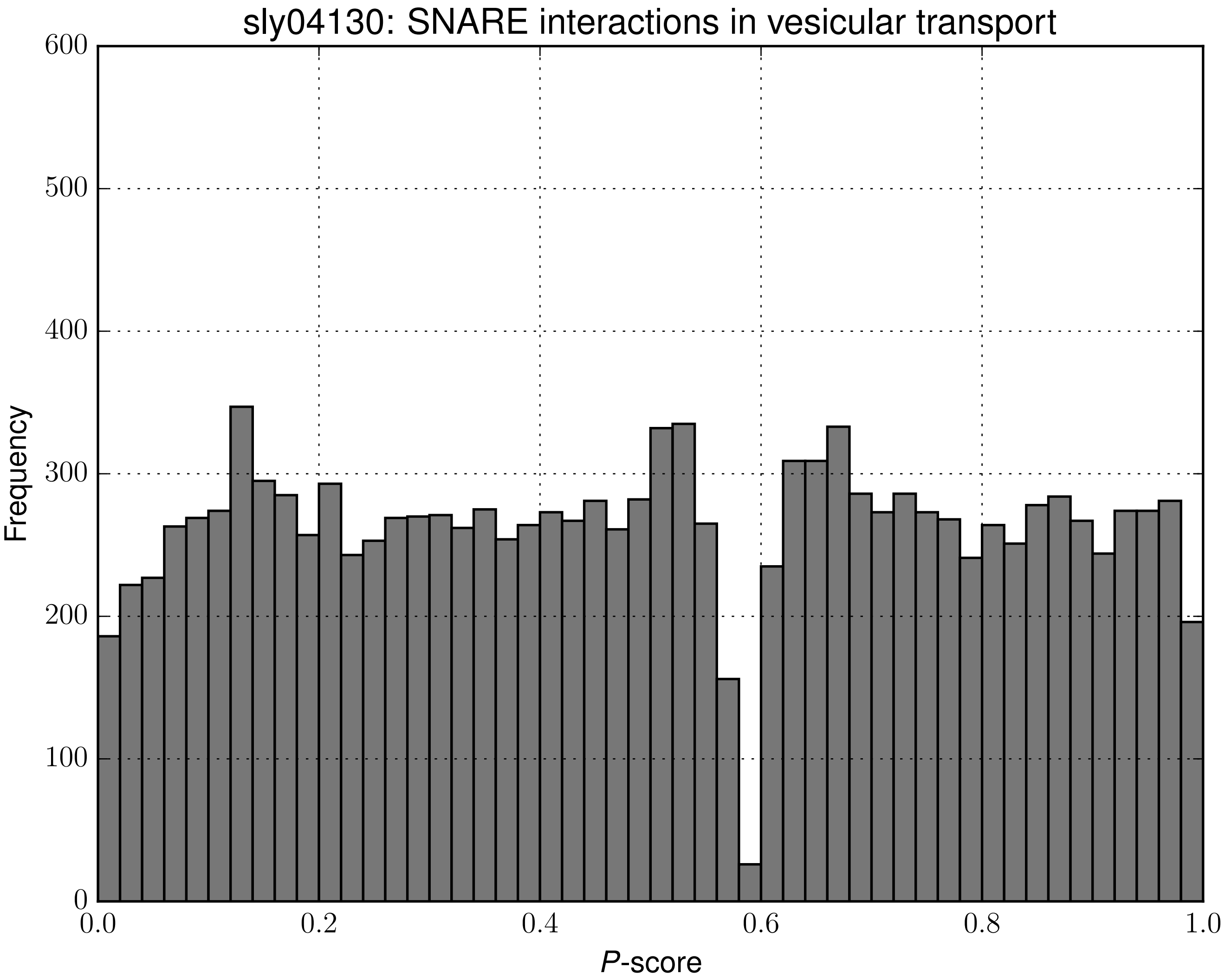

sly04130: SNARE interactions in vesicular transport
600
500
400
300
200
100
0
Frequency
0.0
0.2
0.4
0.6
0.8
1.0
P-score

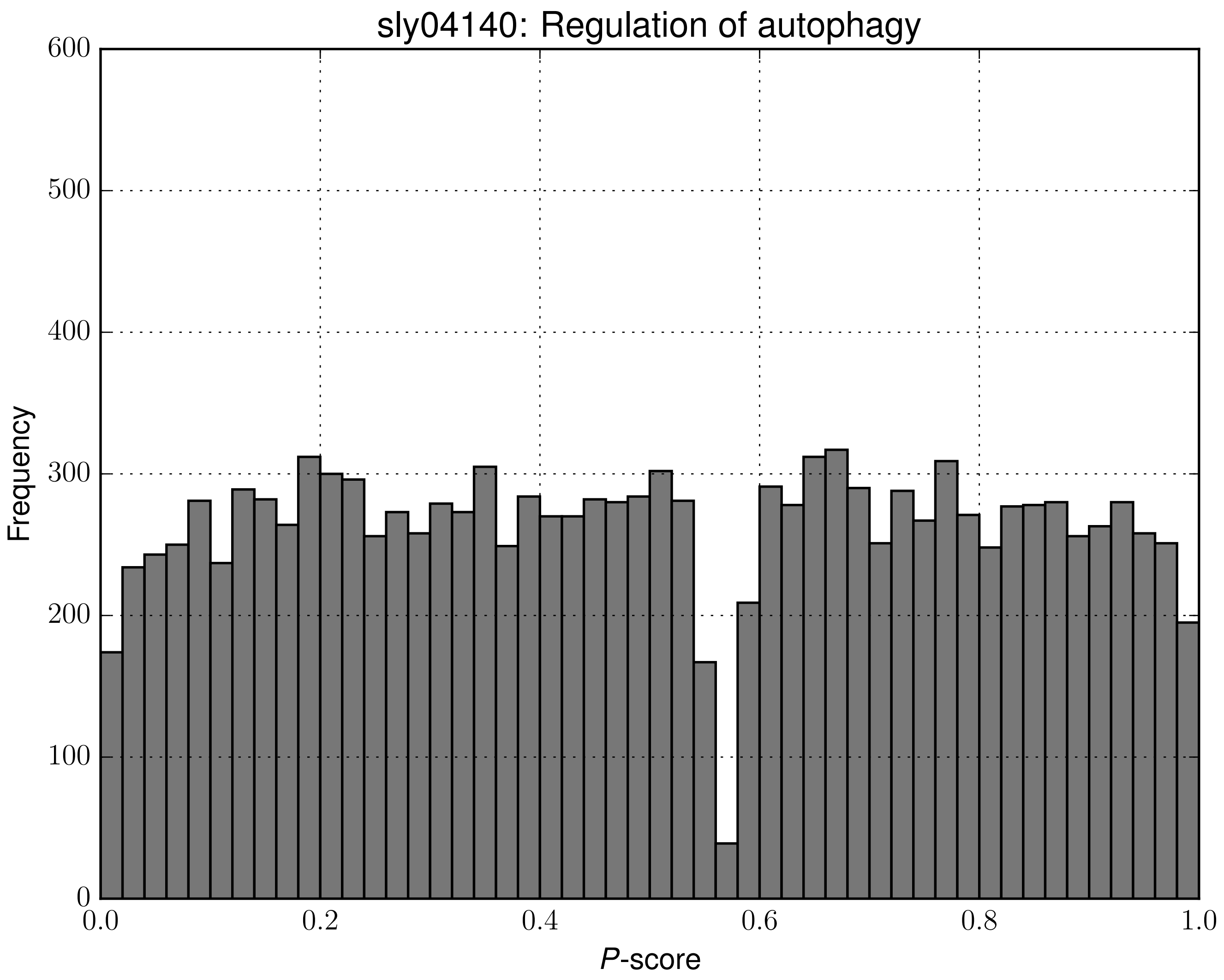

sly04140: Regulation of autophagy
Frequency
P-score
0
100
200
300
400
500
600
0.0
0.2
0.4
0.6
0.8
1.0

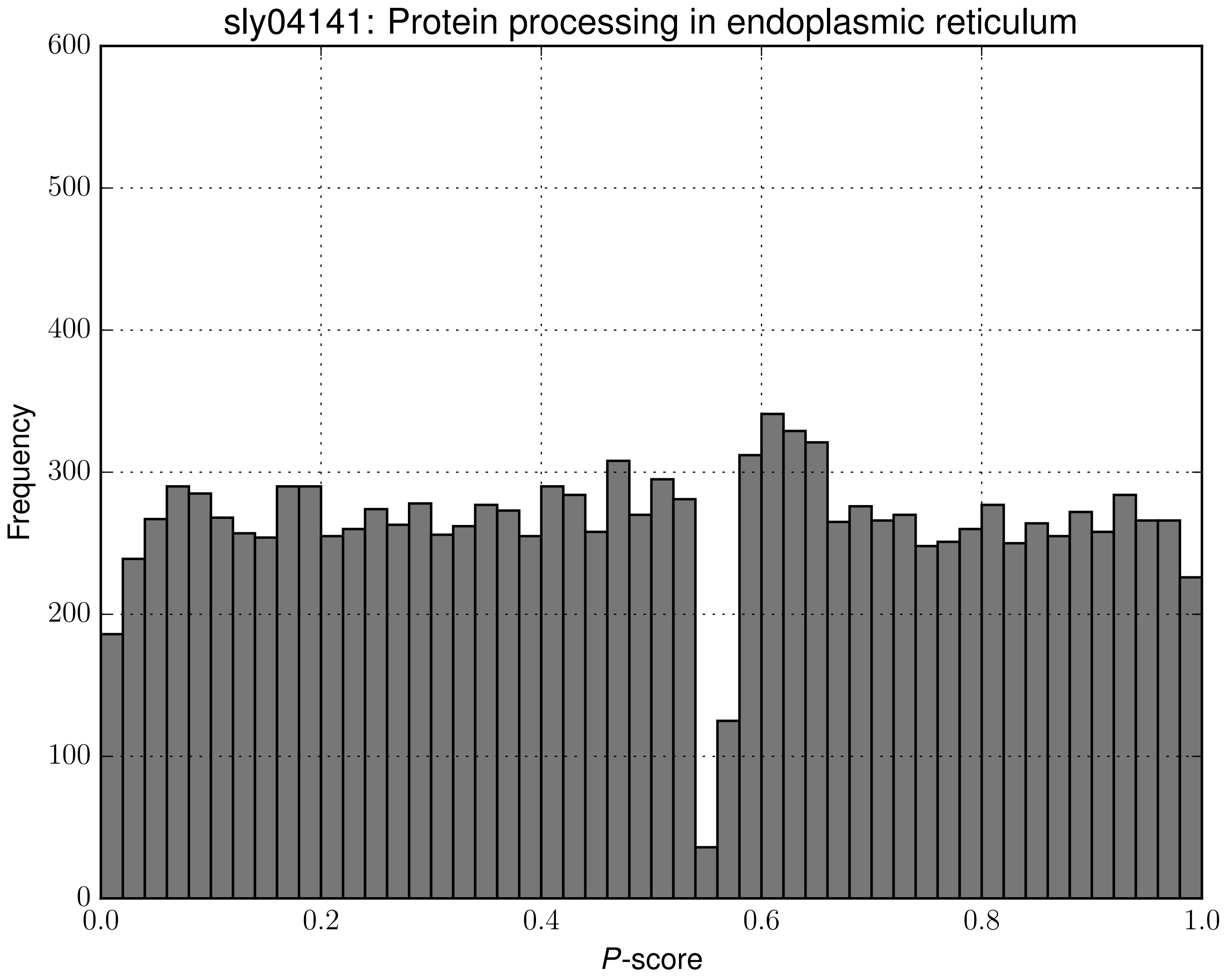
sly04141: Protein processing in endoplasmic reticulum
Frequency
P-score
0
100
200
300
400
500
600
0.0
0.2
0.4
0.6
0.8
1.0

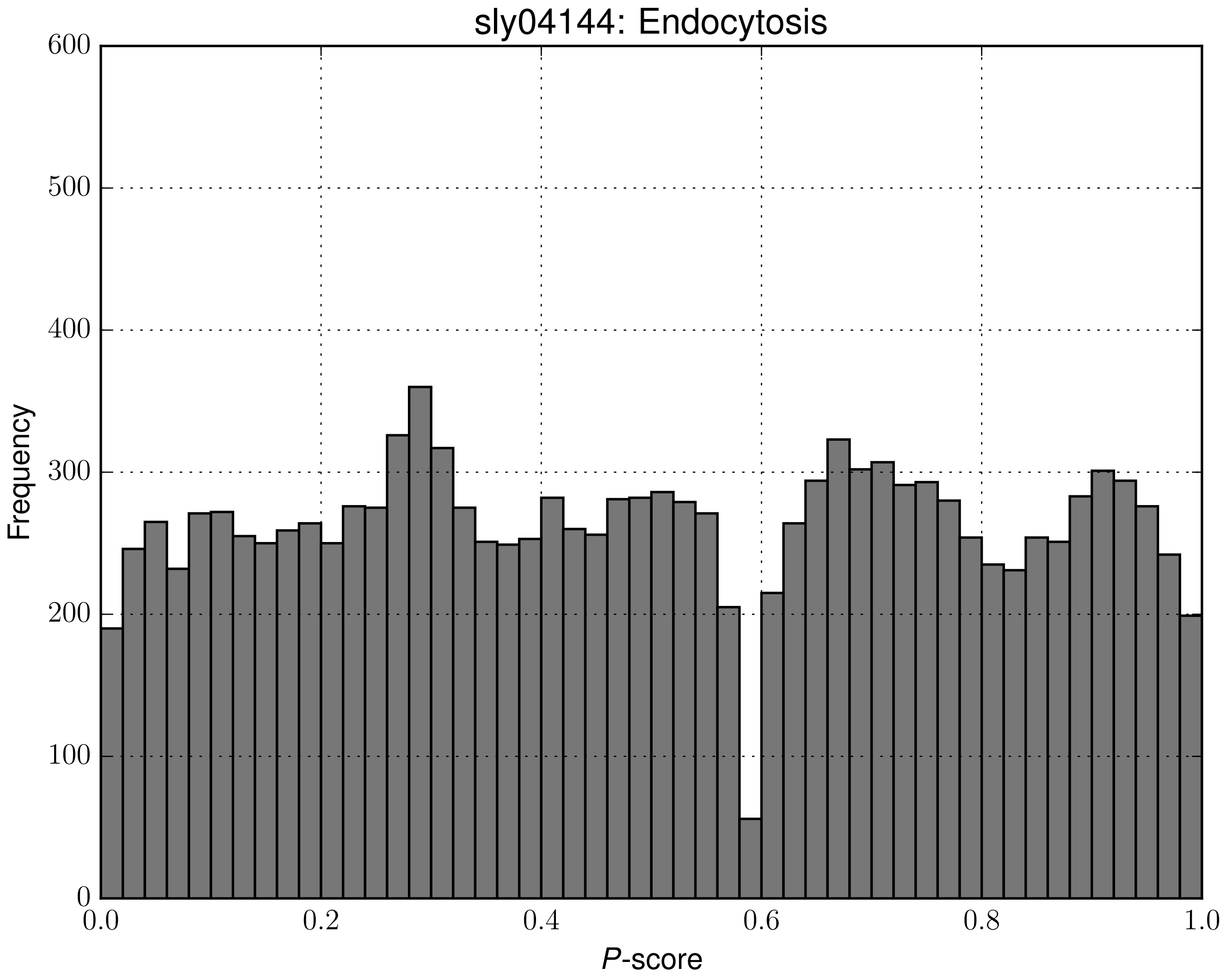
sly04144: Endocytosis
600
500
400
300
200
100
0
0.0
0.2
0.4
0.6
0.8
1.0
Frequency
P-score

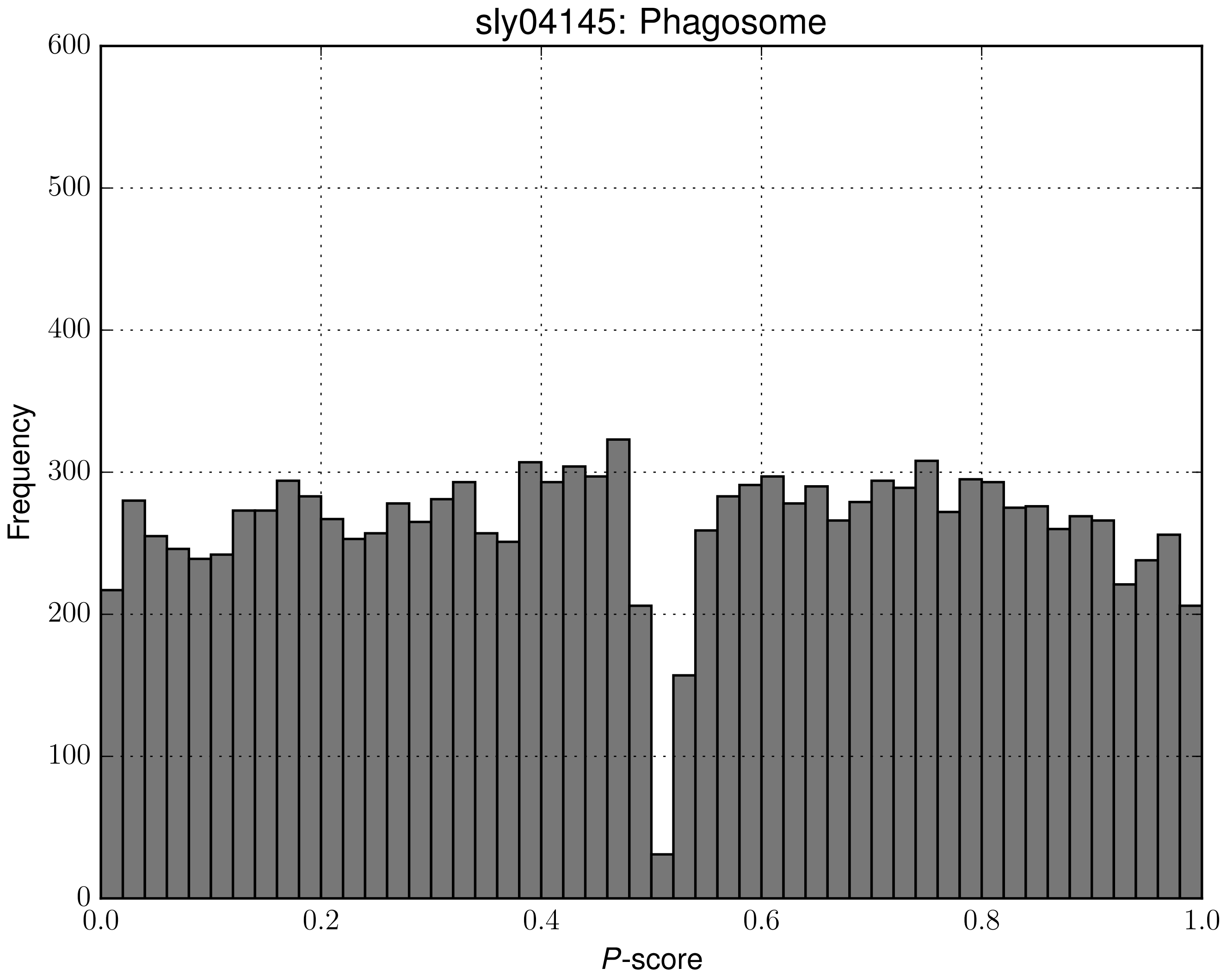
sly04145: Phagosome
Frequency
P-score
0
100
200
300
400
500
600
0.0
0.2
0.4
0.6
0.8
1.0

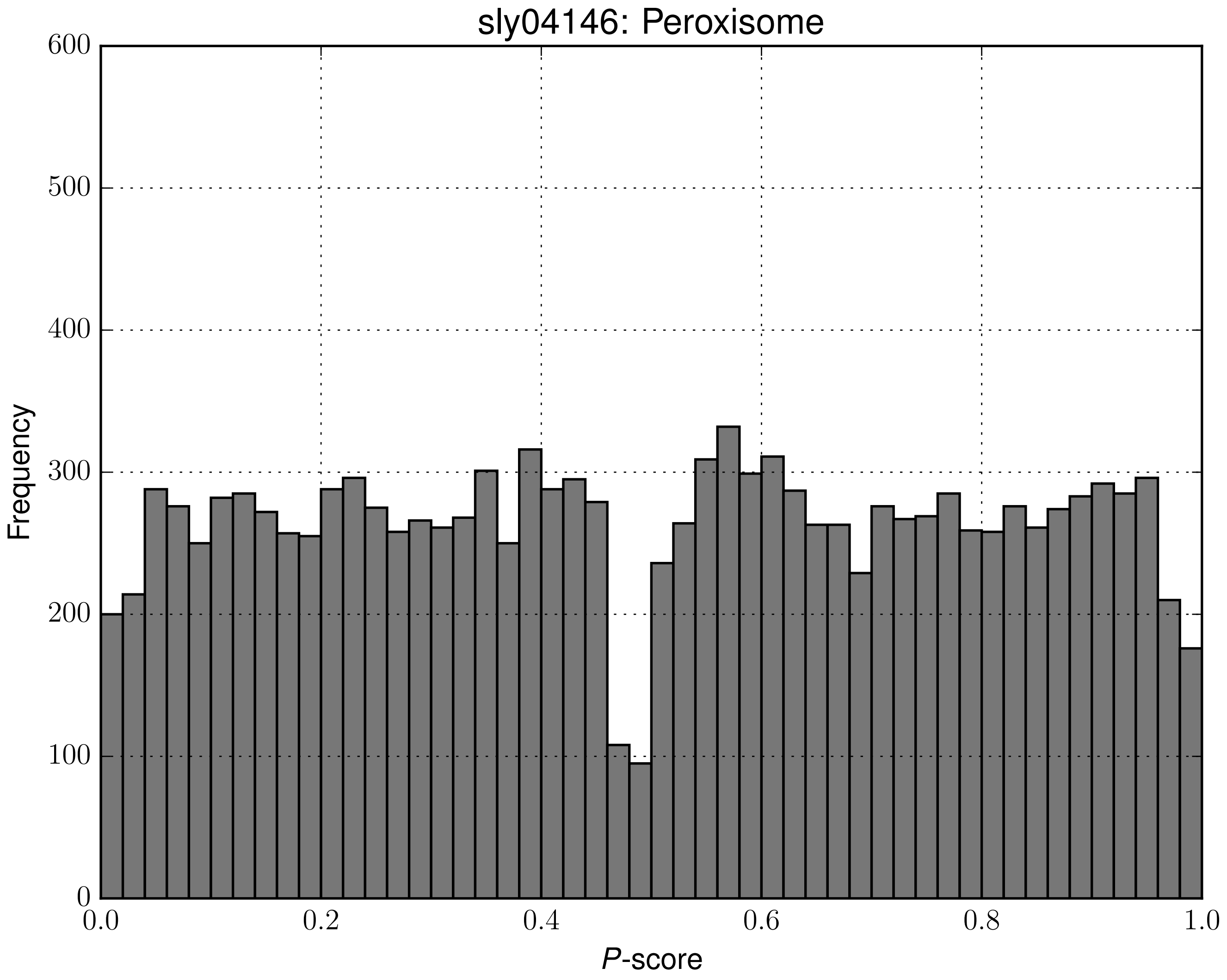
sly04146: Peroxisome
Frequency
P-score
0
100
200
300
400
500
600
0.0
0.2
0.4
0.6
0.8
1.0

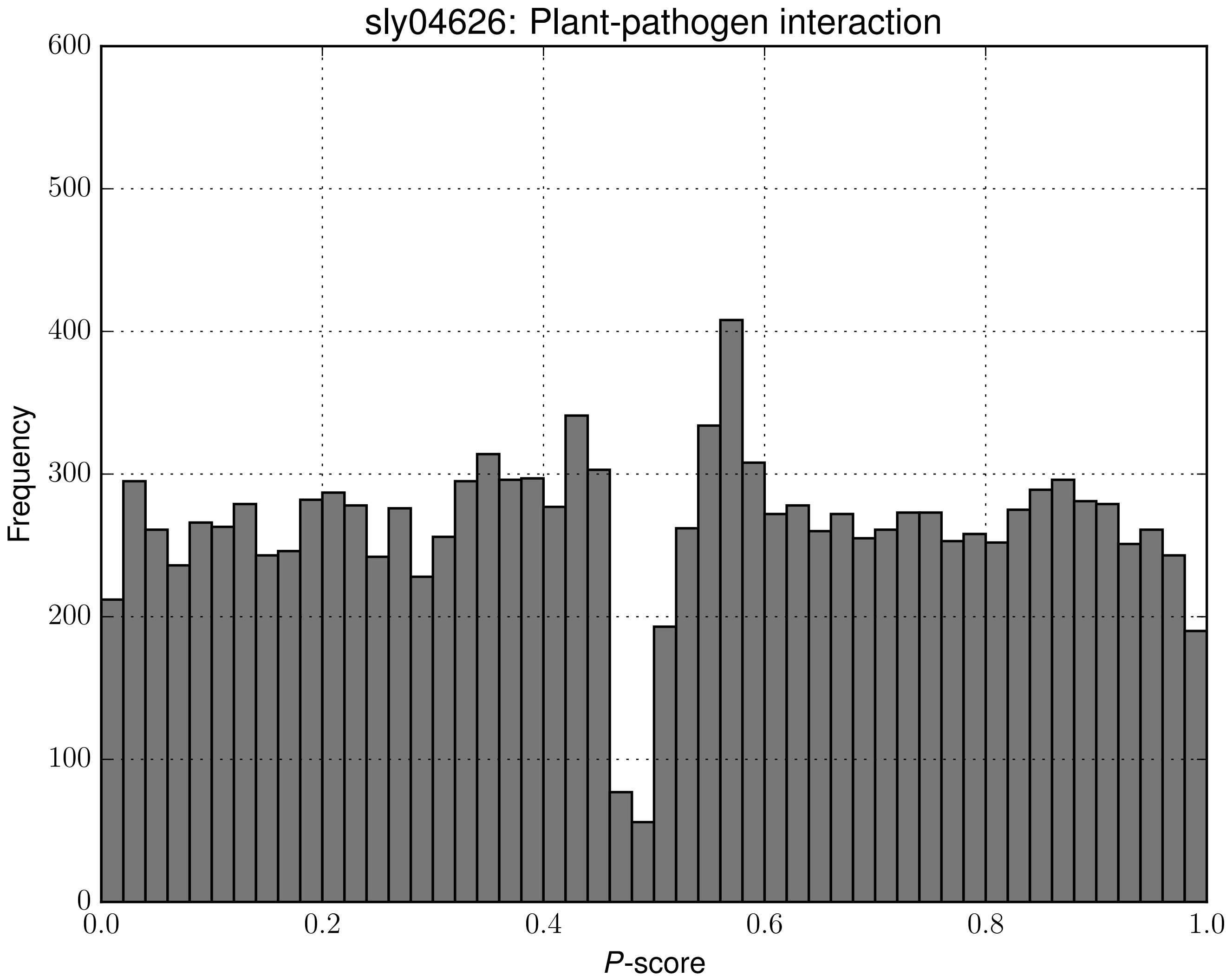
sly04626: Plant-pathogen interaction
Frequency
P-score
0
100
200
300
400
500
600
0.0
0.2
0.4
0.6
0.8
1.0

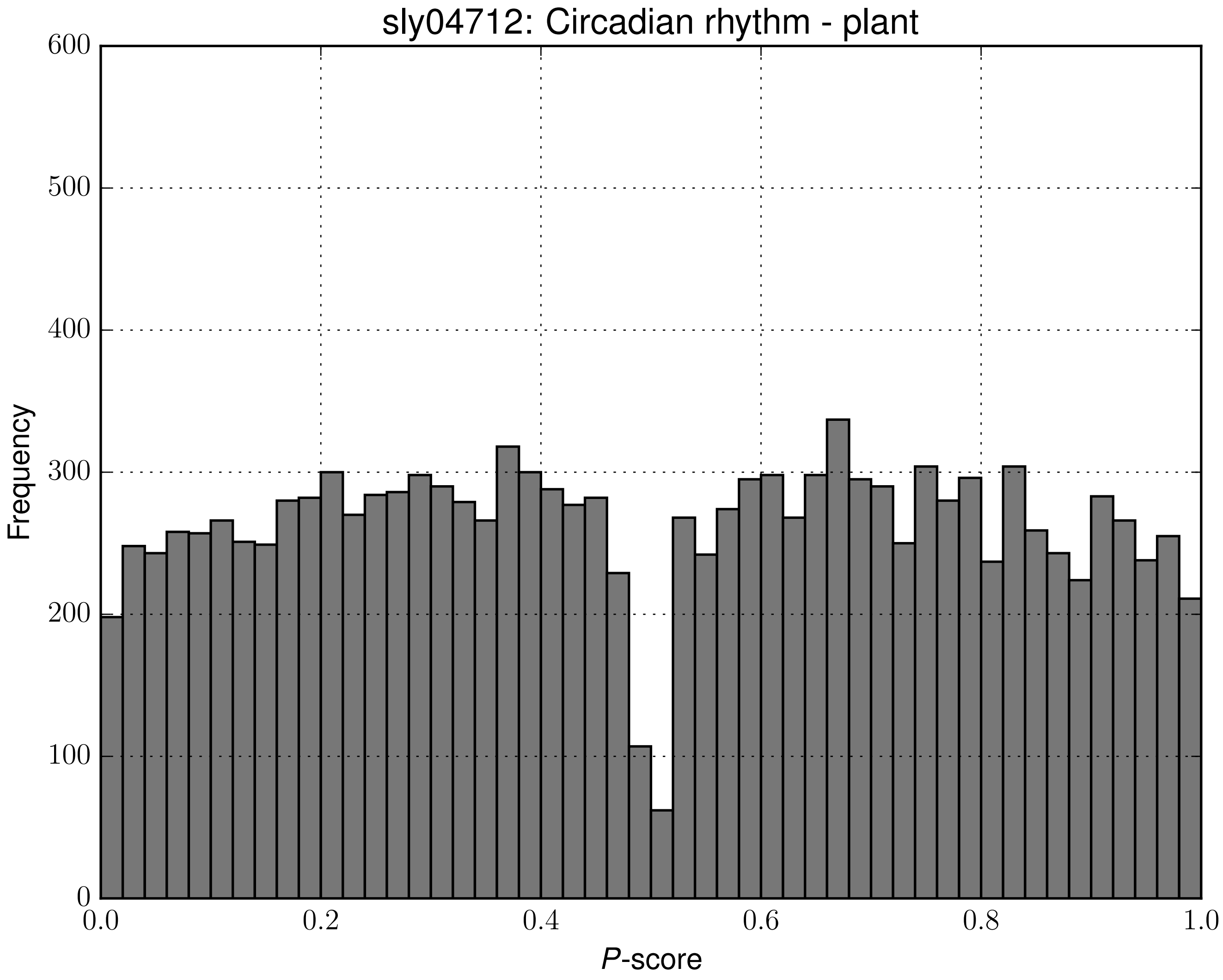
sly04712: Circadian rhythm - plant
Frequency
P-score
0
100
200
300
400
500
600
0.0
0.2
0.4
0.6
0.8
1.0
